# Transcriptomic analysis of the 12 major human breast cell types reveals mechanisms of cell and tissue function

**DOI:** 10.1371/journal.pbio.3002820

**Published:** 2024-11-05

**Authors:** Katelyn Del Toro, Rosalyn Sayaman, Kate Thi, Yamhilette Licon-Munoz, William Curtis Hines

**Affiliations:** 1 Department of Biochemistry and Molecular Biology, University of New Mexico School of Medicine, University of New Mexico, Albuquerque, New Mexico, United States of America; 2 Life Sciences Division, Lawrence Berkeley National Laboratory, Berkeley, California, United States of America; Consejo Nacional de Investigaciones Científicas y Técnicas: Consejo Nacional de Investigaciones Cientificas y Tecnicas, ARGENTINA

## Abstract

A fundamental question in biology, central to our understanding of cancer and other pathologies, is determining how different cell types coordinate to form and maintain tissues. Recognizing the distinct features and capabilities of the cells that compose these tissues is critical. Unfortunately, the complexity of tissues often hinders our ability to distinguish between neighboring cell types and, in turn, scrutinize their transcriptomes and generate reliable and tractable cell models for studying their inherently different biologies. We have recently introduced a novel method that permits the identification and purification of the 12 cell types that compose the human breast—nearly all of which could be reliably propagated in the laboratory. Here, we explore the nature of these cell types. We sequence mRNAs from each purified population and investigate transcriptional patterns that reveal their distinguishing features. We describe the differentially expressed genes and enriched biological pathways that capture the essence of each cell type, and we highlight transcripts that display intriguing expression patterns. These data, analytic tools, and transcriptional analyses form a rich resource whose exploration provides remarkable insights into the inner workings of the cell types composing the breast, thus furthering our understanding of the rules governing normal cell and tissue function.

## Abstract 2

Determining how different cell types coordinate to form and maintain tissues is crucial to understand cancer and other pathologies. This study presents a transcriptomic analysis of the twelve major cell types that make up the human breast, providing a resource to study cell-intrinsic characteristics and human breast tissue composition.

## Introduction

Understanding the biology of tissues and ultimately developing effective cancer therapies requires us to recognize the symphony of interactions in tissues that occur among its many different cell types—and the myriad of molecules they synthesize. Determining how cells communicate with each other and their microenvironments is critical, as is defining the roles of stromal cells in regulating epithelial cell behavior, shaping tissues, and promoting or preventing overt cancer [1–4]. To this puzzle, there are many pieces. Clarifying and appreciating the roles of each cell type requires a substantial foundation in our ability to identify and distinguish among these distinct tissue elements so we may learn how they fit together to form a functioning organ. Tumors are caricatures of normal tissues [5], yet the intricate cellular composition and arrangement of cells in many tissues, including the breast, have yet to be adequately dissected, leaving many unanswered questions about these cells’ unique and essential qualities.

**Fig 1:**
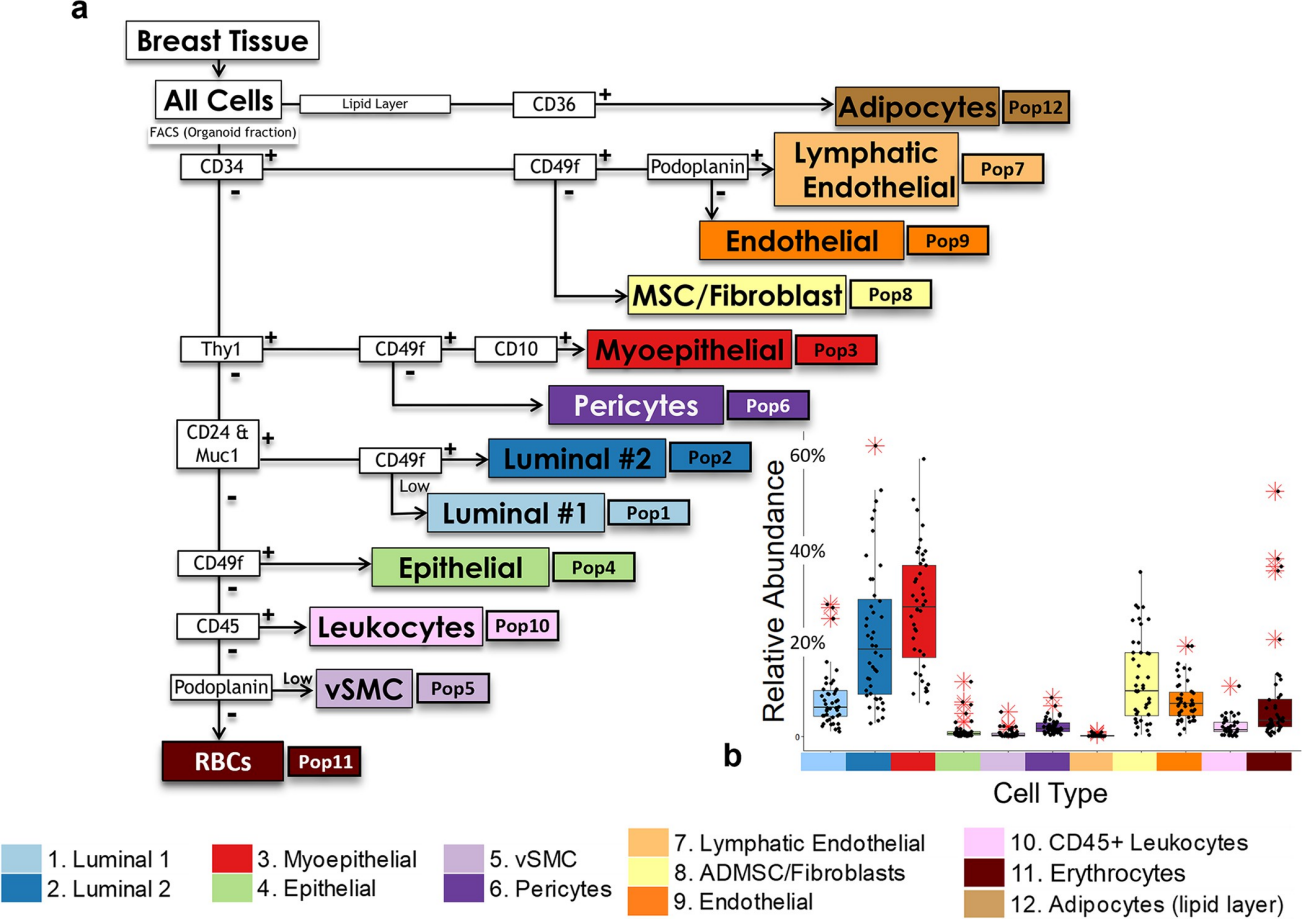
FACS gating strategy for comprehensive cell type isolation. (**a**) Prior to FACS isolation, breast tissues were enzymatically digested with collagenase. This was followed by low-speed centrifugation, which yielded an organoid pellet, supernatant, and an adipocyte-enriched lipid layer. RNA was isolated from the lipid layer (adipocytes, Pop12) and stored for later sequencing. The remaining cell types were FACS purified from the trypsin-dissociated organoid cell pellet, according to the outlined gating scheme. Each cell type was assigned a population number that corresponds to each population’s isolation strategy (Pops 1–12). (**b**) Relative abundance of each cell type from 30 different individuals (proportion of total cell counts, as measured by FACS analysis of 44 independent FACS experiments). Outliers (*) are defined as points falling (1.5 × IQR) above/below the 75th and 25th quartile. FACS, fluorescence-activated cell sorting.

The human dissections and inspiring observations made by Andreas Vesalius, William Harvey, and other Renaissance anatomist-physicians produced astonishing breakthroughs in medical science [6,7]. With a similar passion for anatomical dissection, we have recently applied modern tools of histology and cell and molecular biology to examine the fabric of the human breast—to learn its cellular composition and study its mechanistic underpinnings [8]. Using immunofluorescence and advanced flow cytometry, we developed methods to resolve, isolate, quantify, and culture each breast cell type, reconciling every cell in the tissue. The product of this labor was a fluorescence-activated cell sorting (FACS) strategy that used redundant markers and a rigorous gating strategy to identify the 12 distinct breast cell types (Fig 1A and Fig A in S1 Text). These populations included 2 separate luminal epithelial cell types, myoepithelial cells, adipocytes, leukocytes, pericytes, vascular smooth muscle cells, erythrocytes, adipose-derived mesenchymal stem cells (fibroblasts), lymphatic and vascular endothelial cells, and a newly identified rare epithelial cell population with unknown function. Although this FACS method resolved leukocytes as a single CD45^Pos^ fraction, the strategy is adaptable and can be further refined to scrutinize the array of leukocyte populations in the tissue. Primary cell models were generated from nearly every cell type—some being the first of their kind from this tissue. These are breast pericytes, lymphatic endothelial cells, and our newly defined epithelial cell population. Their isolation and culture add to the repertoire of available models and permit functional studies that were not previously possible [8].

Here, we ask the critical question of what defines each breast cell type—and seek to identify their individual features and functions. Unsurprisingly, hierarchical clustering of transcriptional profiles echoed FACS results by cleanly separating the 11 sequenced cell types—revealing remarkable correlations among biological replicates (of each cell type, isolated from random individuals). Principal component analysis (PCA) established genes that heavily contribute to cellular diversity. We used PCA-derived gene lists to identify the distinguishing features of epithelial, mesenchymal, endothelial, and leukocyte lineages—each being a group of cell types with similar origins and characteristics. These included well-recognized features, such as keratinization of epithelial cells, muscle contraction by mesenchymal cells, vasculogenesis by endothelial cells, and more. While these results validated our analytic approach, others revealed many processes we had not previously associated with a specific cell lineage.

Shifting to a more gene-centric approach, we explored the families of genes that contribute to cell type-specific functions by identifying the gene families that varied most across the spectrum of cell types. Many gene families (as a group) showed little to no change in expression (e.g., exportins), whereas others were extensively differentially expressed (e.g., laminins, integrins, Rho GTPase activating proteins, and more). This information highlights the types and properties of protein-encoding genes that most contribute to cell type differences.

Differences in gene expression and the biological processes in which these genes are involved were further solidified—and others identified—via comparisons between cell type pairs. We achieved this through the application of gene set enrichment and pathway analyses to reveal the “essence” of each cell type. We highlighted 6 comparisons that we believed to be of the highest interest as they reveal features of every major cell type and identify those distinguishing closely related cell types. Moreover, we have performed all 55 possible pairwise cell type comparisons and provided the results in a user-friendly analysis tool (Pathway Analyzer, Supplementary Data File 12 at https://osf.io/GFHC3/ [9]) and web application (Breast Cell Atlas: Transcript Finder [10]). These tools allow one to thoroughly engage with the data set and actively explore each cell type’s expressed genes and enriched biological pathways.

## Results and discussion

### Identification of cell lineages and analytic pipeline

**Fig 2:**
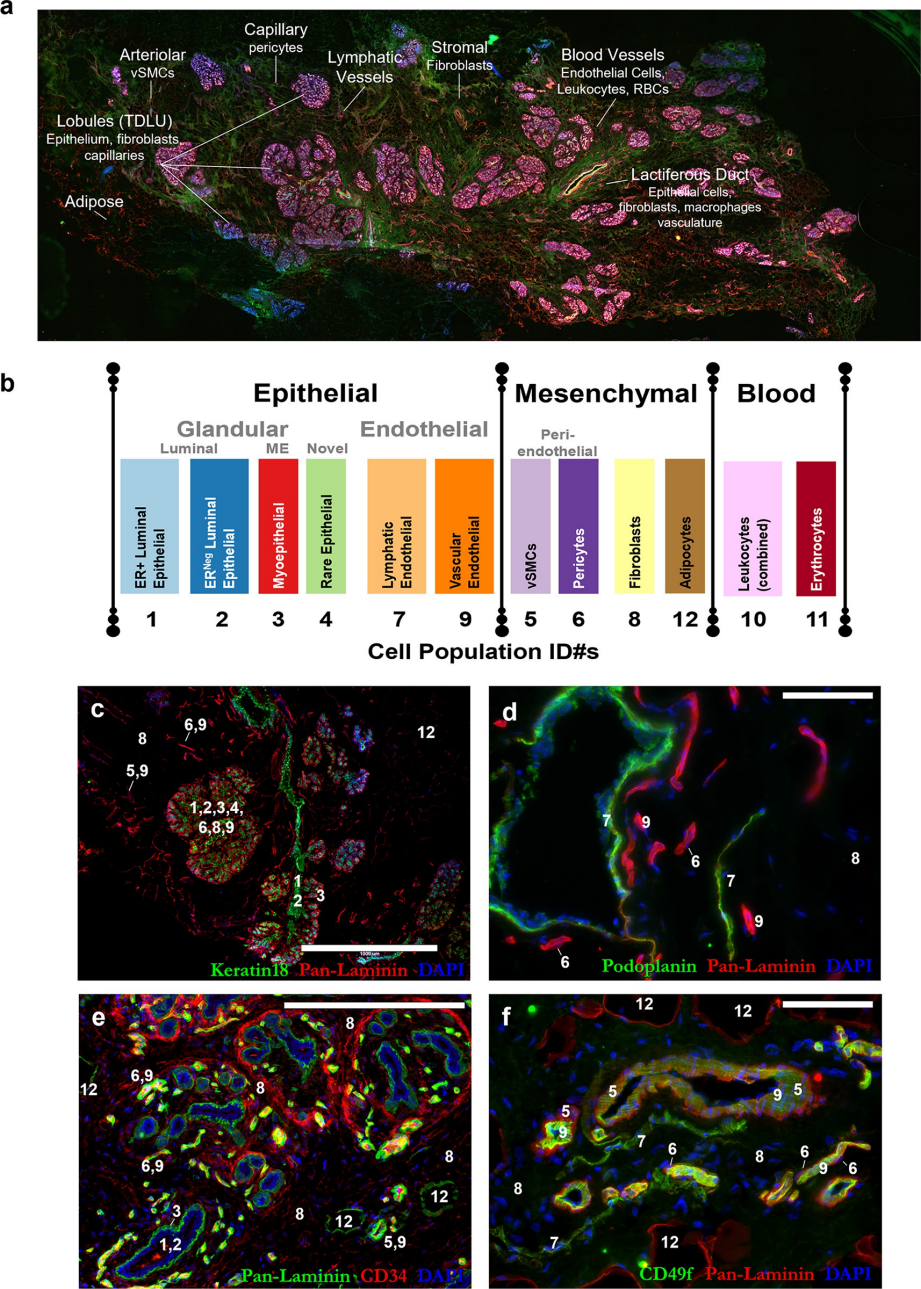
IHC staining of cell types in the breast. **(a)** Representative 4× scan of normal breast tissue (reduction mammoplasty tissue from 23-year-old female). The general locations of the 12 cell types are indicated by their FACS population identification numbers, Pops1-12. **(b)** Chart of the 12 breast cell types and their assigned colors (used throughout this article). Cell types are grouped by their common histological classification: epithelial, mesenchymal, and blood cell types. Epithelial types are further subdivided into glandular and endothelial types, whereas the mesenchymal perivascular types are indicated (pericytes and vascular smooth muscle cells). **(c–f)** Spatial position of the cell types in normal tissue immunostained with: **(c)** keratin 18 (green; luminal epithelial cells) and pan-laminin (red; epithelial and vascular basement membrane, and adipocytes). Staining reveals a collecting duct within several breast lobules (center) composed of epithelial (Pops 1–4), fibroblasts (Pop8), and capillaries that consist of pericytes (Pop6) and vascular endothelial cells (Pop9). Stromal adipocytes are also present (Pop12, upper-right). Scale = 1,000 μm. **(d)** Podoplanin (green) and pan-laminin (red) staining reveal podoplanin-staining lymphatic vessels (Pop7) and vascular blood vessels (Pop9). Laminin embedded pericytes (Pop6) are also discernible on the capillaries; Scale = 200 μm; **(e)** CD34 (red) and pan-laminin (green) staining reveals a cross-section of three-and-a-half breast lobules within a bed of CD34^Pos^ fibroblasts. Endothelial cells forming capillaries and larger blood vessels also stain for CD34, both inside and outside the lobular areas, e.g., larger vessels are visible at lower right, along with pan-laminin staining adipocytes (Pop12); scale = 200 μm. **(f)** CD49f (green) and pan-laminin (red) reveals a large blood vessel (center) surrounded by vascular smooth muscle cells (Pop5). Endothelial cells (Pop9) and pericytes (Pop6) of smaller capillaries are present, as are lymphatic vessels (Pop7) and adipocytes (Pop12); scale = 200 μm. FACS, fluorescence-activated cell sorting.

The breast is a compound tubuloalveolar gland whose subcutaneous tissue comprises, at a minimum, 12 major cell types. In a recent article, we describe the development and validation of an antibody panel and gating strategy capable of isolating these cell types [8]—cell populations we numbered from 1 to 12 (which refers to their respective FACS gate using the isolation strategy outlined in Fig 1A and Fig A in S1 Text). These cell populations are described as follows: ER^Pos^ (Pop1/Lum1) and ER^Neg^ (Pop2/Lum2) luminal epithelial cells, myoepithelial cells (Pop3), a rare epithelial cell population (Pop4), vascular smooth muscle cells (vSMCs, Pop5), pericytes (Pop6), lymphatic endothelial cells (Pop7), fibroblasts (Pop8), vascular endothelial cells (Pop9), CD45^Pos^ leukocytes (Pop10), erythrocytes (Pop11), and adipocytes (Pop12). These different cell types are often broadly classified as members of epithelial, endothelial, mesenchymal, or hematopoietic lineages (Fig 2B).

In the early days of this study, we had not yet completed identifying all populations revealed by FACS, and several remained a mystery even after finalizing our isolation strategy. Establishing their identities was particularly challenging. We were aided by several helpful clues, however. One was the distinct morphologies the cell types displayed when cultured. These observations allowed us to determine whether a particular population appeared epithelial-like, e.g., Pop4 epithelial cells, or presented with more mesenchymal-like characteristics, e.g., Pop6 pericytes. We also backtracked and stained tissues using the same antibody combinations used in FACS and hunted for cell types in the tissue that shared identical staining patterns. Ultimately, the transcriptome information from RNA-sequencing provided definitive evidence that solidified each cell type’s identity.

**Fig 3:**
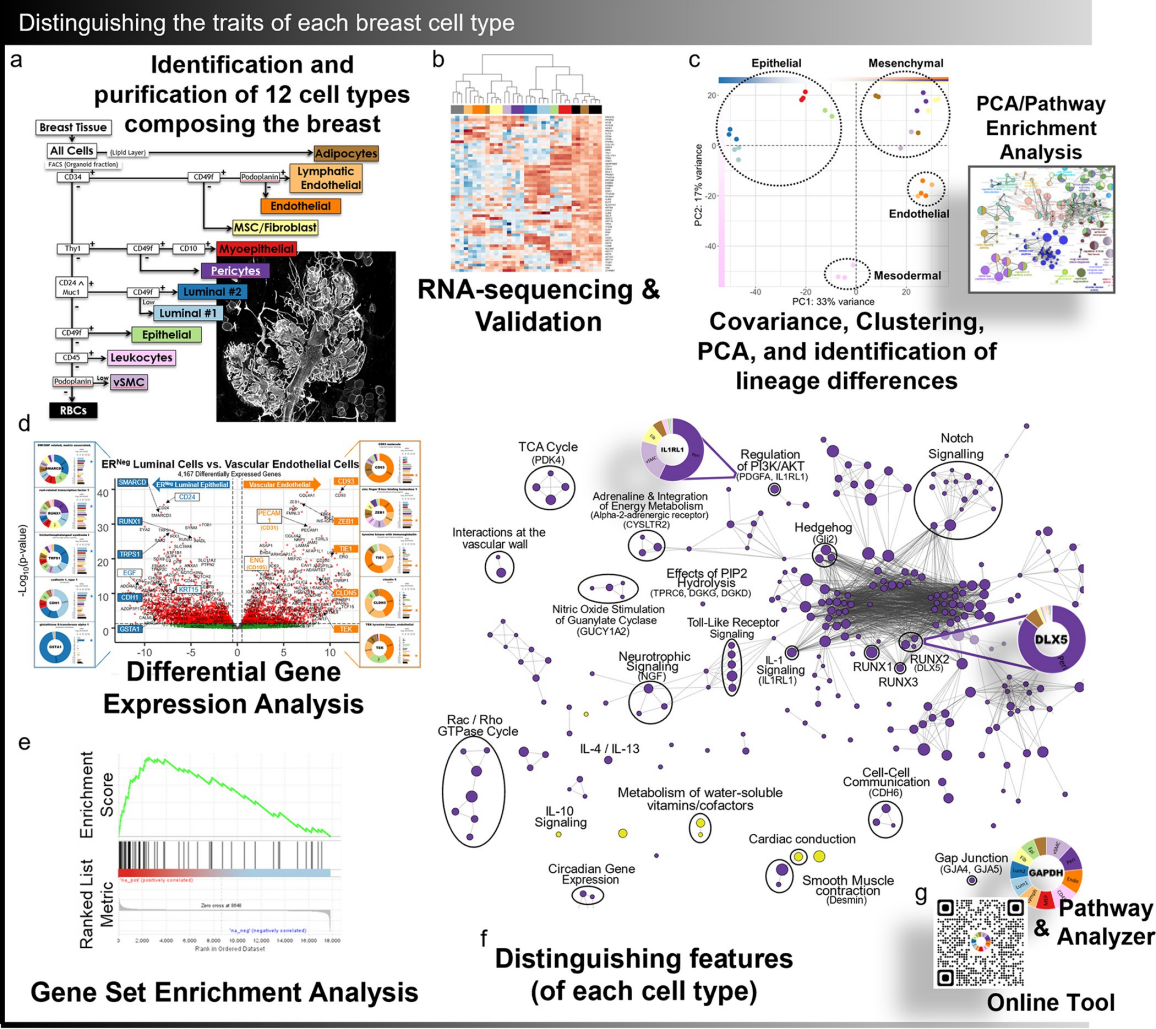
Distinguishing the traits of each breast cell type. Shown is a sketch of the RNA-sequencing workflow used for analyzing the 11 sequenced cell types. This began with **(a)** developing a FACS isolation method for separating each breast cell type (as detailed in Fig 1). This procedure was scaled up and cells were sorted and RNA-sequenced. Analysis of the RNA-sequencing data involved: **(b)** validation and exploration of cellular relationships by hierarchical clustering, **(c)** covariance matrix and quality control analyses, identification of unique cell markers, and PCA. PCA-derived gene lists were used to perform gene ontology enrichment analyses that revealed the shared characteristics of cell lineage. **(d)** Differential gene expression analysis was performed and used to identify gene families that varied most among breast cell types; pairwise comparisons identified transcripts whose levels varied between sample pairs. Differentially expressed genes between cell pairs were ranked and analyzed by **(e)** GSEA, leading to the identification of key pathways and processes that differ between cell types. GSEA results were further analyzed by **(f)** constructing and analyzing enrichment maps that identified the central biological themes and distinguishing features of each cell type. Analyzing the data required the development of software code and packages that are available for download: Pathway Analyzer (Supplementary Data File 12, Open Science Framework, https://osf.io/gfhc3/) and online Transcript Finder (http://breastcancerlab.com/breast-atlas). FACS, fluorescence-activated cell sorting; GSEA, gene set enrichment analysis; PCA, principal component analysis.

With their identities and histological locations now solved (Fig 2A–2F and Figs B–D in S1 Text) [8], we shifted our attention to exploring the individual biologies of these different cell types by diligently dissecting their transcriptomes through a comparative lens provided by the comprehensive collection of cell types we had isolated and sequenced. Our comprehensive analytic pipeline is described in Fig 3 and Fig E in S1 Text.

### mRNA sequencing and validation of FACS purified cell types

To investigate the unique biology of the breast cell types, we FACS isolated each population to sequence and analyze their transcriptomes. This process entailed processing and collagenase-treating breast tissues (reduction mammoplasty tissues free of proliferative disease), followed by low speed (<100 g) centrifugation. The first population collected was the lipid layer that had phase-separated under centrifugal force (Pop12 adipocytes). All other cell types were FACS-purified from the pelleted tissue “organoids” following their trypsinization to single-cell suspensions (Fig 1 and Fig A in S1 Text). While developing and testing our FACS antibody panel, we typically stained between 100,000 to 500,000 cells [8]. However, many more were needed for mRNA library construction and sequencing here, so we scaled up our operation by increasing the number of cells stained by roughly 1,000-fold.

**Fig 4:**
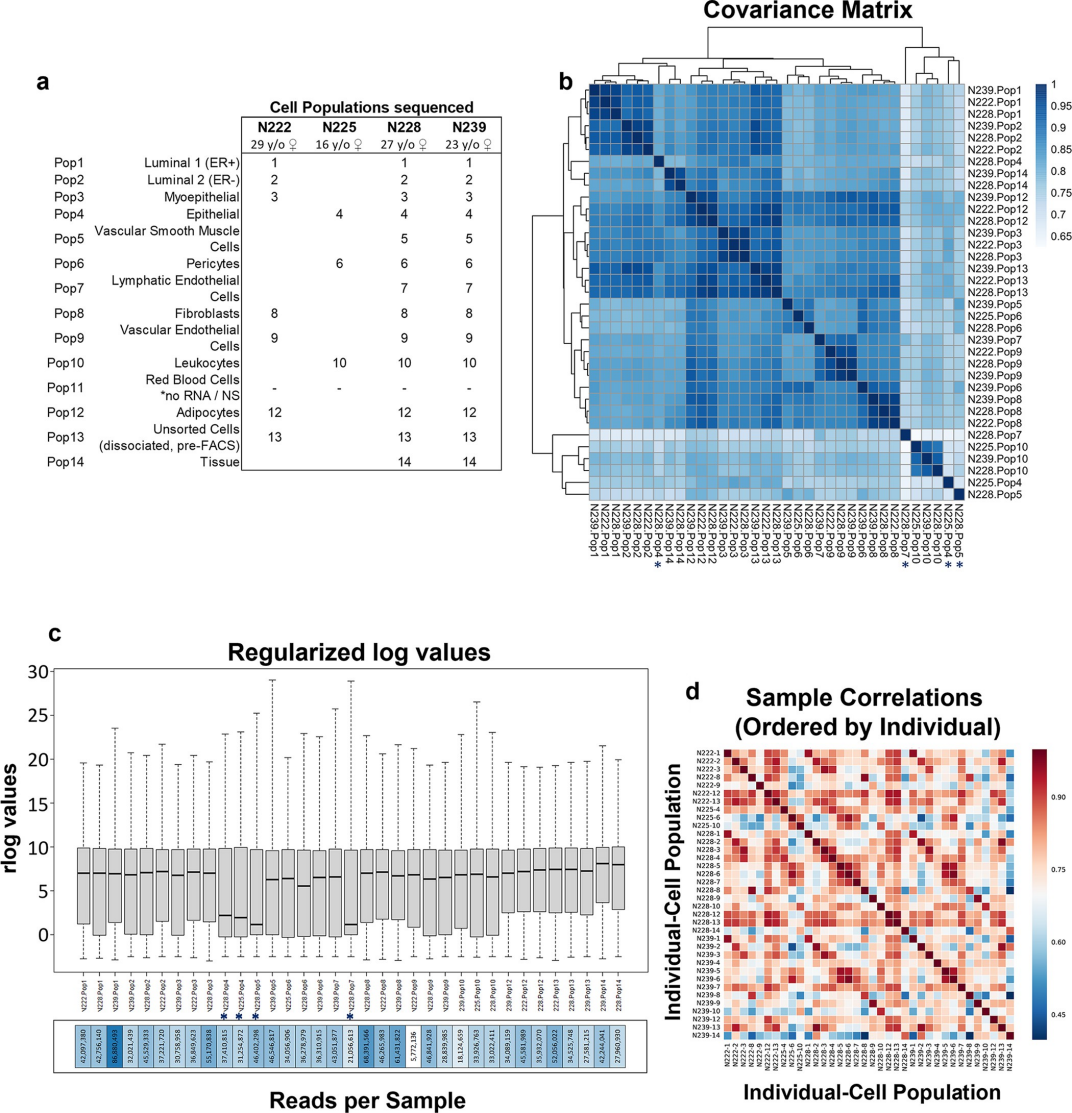
RNA-seq quality assessment. **(a**) Sample metadata for the mRNA-sequenced cell populations. **(b)** Covariance matrix displaying the correlation of gene expression for all pairwise combinations of samples. Samples N228-Pop4, N225-Pop4, N228-Pop5, N228-Pop7 have been flagged for lower correlations between replicates (*). These cell populations are the least abundant types in the breast and are among those that produced the lowest FACS cell yields **(c)** distribution of regularized log transcript levels in each sample. **(d)** Sample correlation matrix demonstrates the absence of sample/batch effects. (Supplementary Data File 13 at 10.17605/OSF.IO/GFHC3.) FACS, fluorescence-activated cell sorting.

FACS purifying cells for RNA-sequencing was performed on freshly collected tissues. This process required relatively large amounts of tissue (>30 grams) and sort times (14 to 18 continuous hours for each tissue) to produce cell yields that could support deep RNA-sequencing. In addition to the above lipid layer, the sorting provided 11 FACS-purified cell populations from each specimen. This exercise yielded hundreds of thousands to millions of cells for the more abundant cell types (Fig E in S1 Text and S1 data). In contrast, yields were much lower for the rarer types, which produced a range of only 24,000 to 130,000 cells. After cell purification, the RNA isolated from each sample correlated with cell counts, and in pilot experiments, we found it essential to isolate RNA immediately following FACS to preserve its integrity. In 2 independent sequencing runs, we sequenced 36 samples representing 11 of the 12 identified breast cell types (red blood cells did not produce measurable RNA and thus were not sequenced). All cell populations were isolated from 4 random individuals (aged 16, 23, 27, and 29 years/old), and nearly all were sequenced in triplicate (from 3 of the 4 individuals, S1 Data). In only 2 instances did we not have enough RNA of sufficient quality to sequence in triplicate—and both were rare populations with low FACS yields (Pop5 vascular smooth muscle cells and Pop7 lymphatic endothelial cells; Figs 1B and 4A). We also isolated and sequenced RNAs from snap-frozen tissues and unsorted cell suspensions, which we used as mixed cell controls. After sequencing mRNAs from the samples and aligning the FASTQ sequencing data to the human genome, we calculated the average mRNA counts across all samples to be 39.5 million paired-end reads—all with Phred [11] quality scores of 25 or more, indicating the sequencing was of high depth and quality. We found these early sequencing results encouraging and moved forward with quality control analyses.

Nearly all sequenced samples demonstrated excellent read counts. However, covariance analysis, assessment of Cook’s distances, and distribution of regularized log values [12] identified 5 samples with reduced transcript complexity and lower correlations. All were the rarer cell types, i.e., Pop4 epithelial cells, Pop5 vSMCs, and lymphatic endothelial cells (Fig 4B and 4C). Nevertheless, we determined that only Pop4 epithelial cells from individual N239 needed to be removed from critical downstream analyses. We retained the 4 other low-complexity samples because they clustered well with their cell type counterparts (hierarchical clustering and PCA) and provided meaningful insights for many transcripts (Fig 4B and S2 Data). These samples were: Pop4 epithelial cells (from individuals N225 and N228), Pop5 vSMCs (from N228), and Pop7 lymphatic endothelial cells (from individual N228). Although outlier genes existed in these samples, concordance between replicates was high for most others. Despite these minor issues, the sequencing data across the board was impressively robust. We did not detect sequencing artifacts, such as correlations with sequencing run or individuals from which the cells were derived, as all samples clustered tightly based on their inherent cell type (Fig 4B and 4D). The substantial number of reads detected per sample, the highly correlated replicates, and the large number of unique transcripts detected in these specimens (21,559) indicated that the data set was rich and of excellent quality. Indeed, these data were immediately helpful in determining the identities of several obscure cell populations.

**Fig 5:**
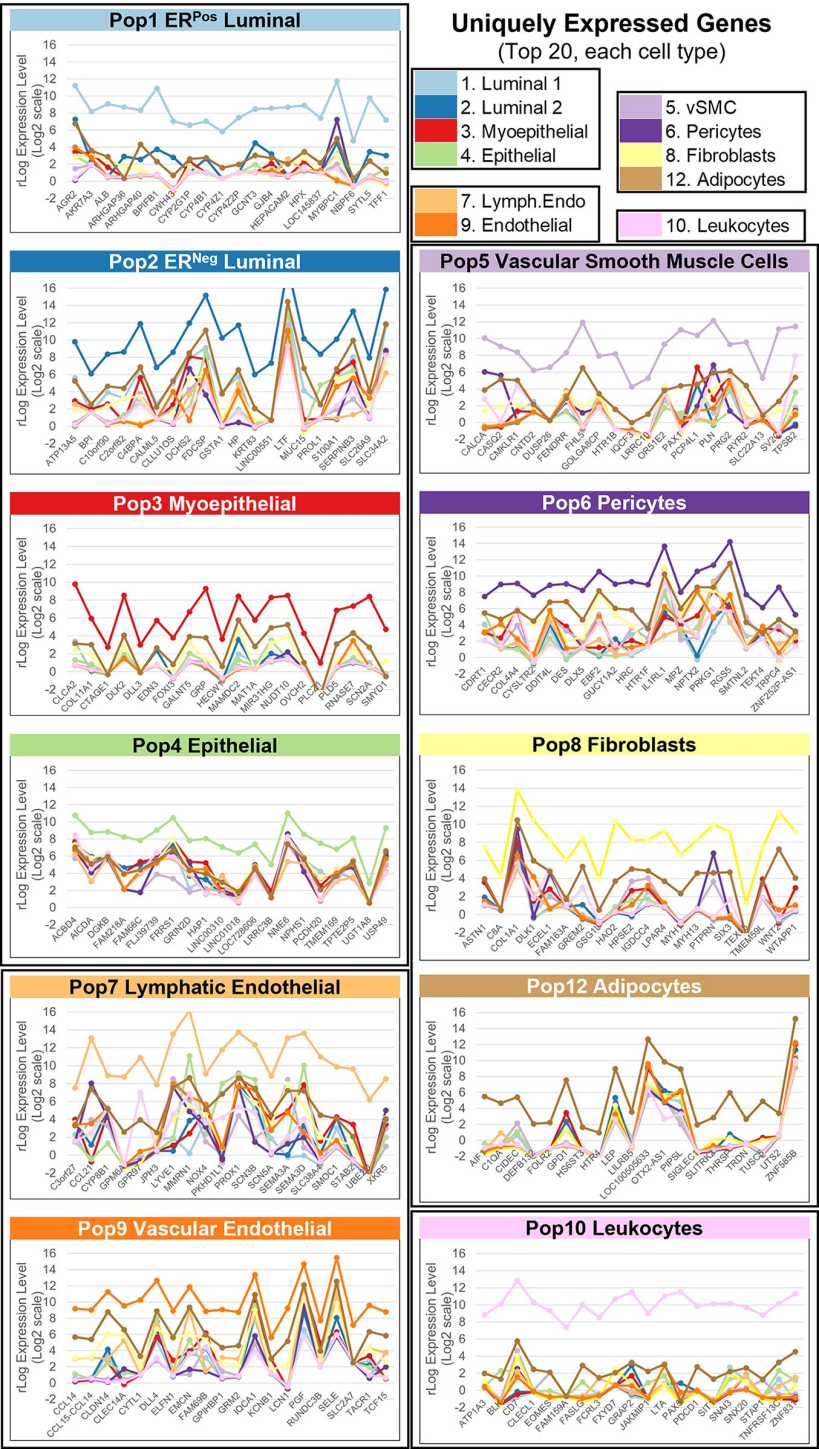
Uniquely expressed genes. A sliding threshold (fold-change) was used to identify the top 20 most uniquely expressed genes in each of the 11 sequenced cell types. Notable genes included: **(a)** Albumin (ALB) and connexin 30.3 (GJB4) in ER-positive luminal cells, **(b)** lactotransferrin (LTF) in ER-negative luminal cells, **(c)** alpha-1 chain of collagen XI (COL11A1) and notch ligand delta-like 3 (DLL3) in myoepithelial cells, **(d)** protocadherin 20 (PCDH20) in Pop4 epithelial cells, **(e)** lymphatic vessel endothelial hyaluronan receptor 1 (LYVE1) and prospero homeobox 1 (PROX1) in lymphatic endothelial cells, **(f)** endothelial selectin (SELE) and delta-like canonical notch ligand 4 (DLL4) in vascular endothelial cells, **(g)** calcitonin-related polypeptide alpha (CALCA) and 5-hydroxytryptamine receptor 1b (HTR1B) in vascular smooth muscle cells, **(h)** desmin (DES) and 5-hydroxytryptamine receptor 1f (HTR1F) in pericytes, **(i)** alpha-1 chain of collagen I (COL1A1) and wnt ligand family member 2 (WNT2) in fibroblasts, **(j)** the lipid-binding protein “cell death inducing dffa-like effector c” (CIDEC) and glycerol-3-phosphate dehydrogenase 1 (GPD1) in adipocytes, and **(k)** CD7 and fas ligand (FASLG) in leukocytes. (Supplementary Data File 13 at 10.17605/OSF.IO/GFHC3).

Before we had sequenced the mRNAs from the cell types, we questioned the identities of several FACS populations [8]. Most notably were populations #4, 5, and 6, which we ultimately identified as a rare epithelial cell type, vascular smooth muscle cells, and pericytes. RNA-sequencing provided conclusive evidence that helped identify these cell types by revealing their relationships to other populations and supplying information on expressed markers. After identifying these 3 populations, we explored all remaining cell types. Reassuringly, transcript levels of genes encoding cell surface markers used to sort each cell type tracked nicely with their respective FACS-staining levels (Fig 1A, Fig F-A and F-B in S1 Text). And GAPDH, often used as a uniformly expressed internal control gene, was indeed expressed equally among the cell types, as were the transcripts of other widely used internal control genes (Fig F6 in S1 Text and S2 Data). In addition, analysis of hundreds of other genes, such as those uniquely expressed by each cell type or those used as cell type markers, i.e., genes encoding CD antigens, validated and helped us build confidence in our data set (Fig 5 and Fig G in S1 Text). To facilitate the exploration of these RNA-sequencing data, we have created a publicly available tool for visualizing transcript levels in each cell type (http://breastcancerlab.com/breast-atlas/).

### Identification of cell type-specific transcriptional patterns

**Fig 6:**
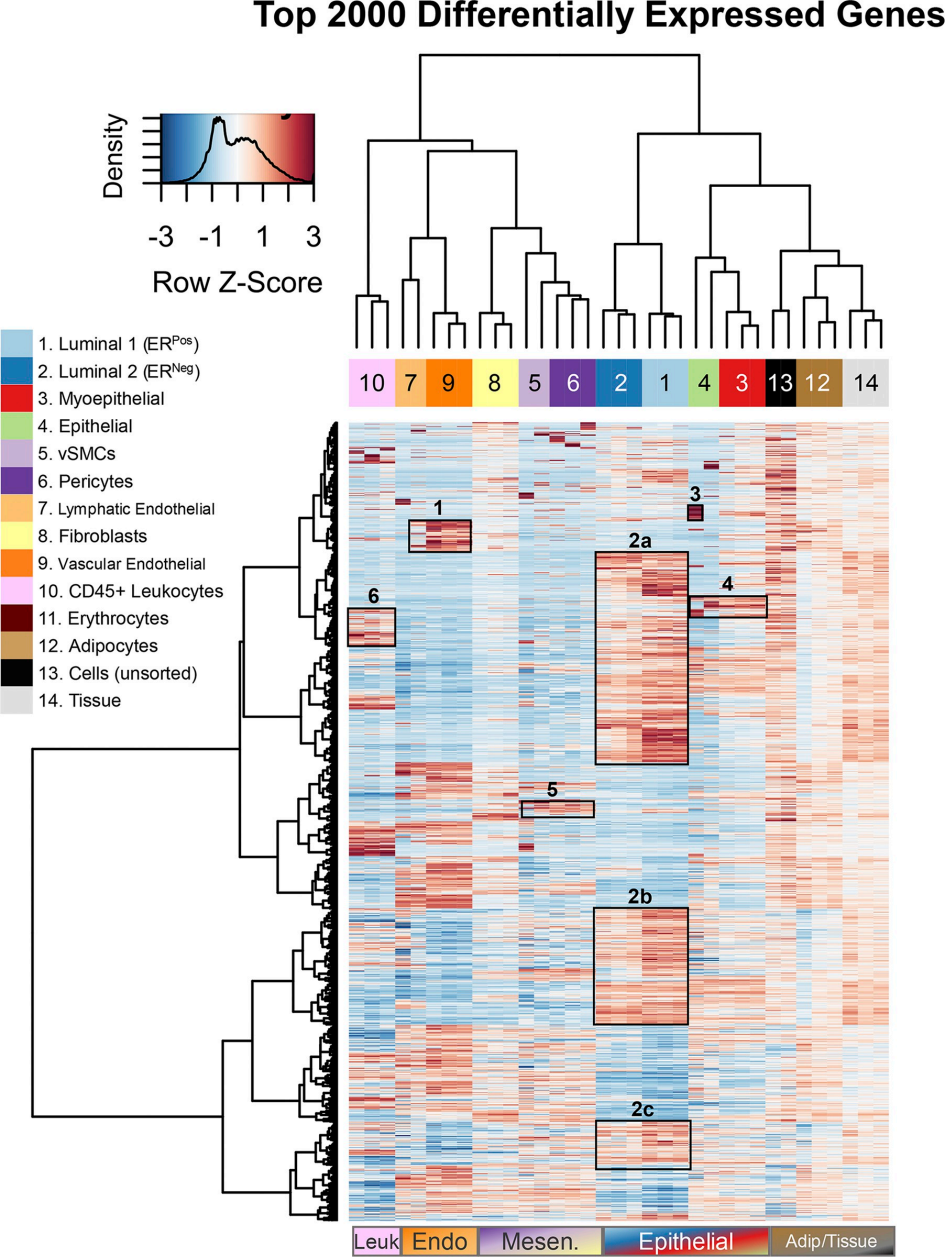
Transcript levels of top 2,000 most differentially expressed genes. Heatmap with dendrogram of the top 2,000 most differentially expressed genes across all FACS sorted populations, as well as unsorted cells and matched breast tissues (mixed cell controls). Clustering was performed using Euclidean distance measures and Ward clustering method on variance stabilized expression values (DESeq2, VST). Adjusted *p*-values for these 2,000 differentially expressed genes ranged between 4.9e-97 to 0.0017. All 2,000 genes symbols can be viewed in S5 Data (when the data is ordered by adj. *p*-value) or Supplementary Data File 13 at 10.17605/OSF.IO/GFHC3. FACS, fluorescence-activated cell sorting; VST, variance stabilized transformation.

One of our early goals was to identify the distinct transcriptional patterns of the different breast cell types. To address this question and further explore the dataset, we clustered the samples’ transcriptomes using a standard Euclidean distance metric. Hierarchical clustering recapitulated the distinct nature of the different cell types and mixed cell controls (Fig 6). Clustering was robust, especially for the 2 luminal cell types, myoepithelial cells, pericytes, fibroblasts, vascular endothelial cells, and adipocyte samples (all of which displayed approximately unbiased (AU) *p-*values >0.95 when evaluated by multiscale bootstrap resampling [13]). Notably, biological replicates of each cell type clustered tightly together, and similar cell types formed adjacent clusters. These related pairs included: (i) vascular and lymphatic endothelial cells (FACS Pops 7&9); (ii) vSMCs and pericytes (Pops 5&6); (iii) ER^Pos^ and ER^Neg^ luminal epithelial cells (Pops 1&2); and (iv) myoepithelial cells and related Pop4 epithelial cells (Pops3&4). As expected, fibroblasts (Pop8) clustered with the other mesenchymal cell types (vSMCs and pericytes)—albeit at a greater distance—reflecting their inherently distinct features. In contrast, the most distant samples that stood far from the others were the CD45^Pos^ leukocytes (Pop10)—a separation that we anticipated, given their unique biology. The clustering confirmed that the 11 sorted cell types were indeed distinct and captured the underlying differences—and similarities—in their gene expression patterns.

Several features emerged after closely examining the hierarchical clustering of genes. Gene expression trends, in particular, were apparent across populations, and many clusters contained genes encoding prominent cell markers, which helped us identify or confirm each cell type’s identity (Fig 6). For example, p-selectin (SELP) and HOXD1 were genes in a cluster that differentiated endothelial cells (Fig 6, box 1). Epithelial cell adhesion molecule (EPCAM), zona occludens 3 (TJP3), and epidermal growth factor (EGF) were among the many genes in a cluster highly expressed by luminal epithelial cells (Fig 6, boxes 2a, 2b, 2c). Calmodulin-like 3 (CALML3) and G-protein-coupled receptor 87 (GPR87) were highly expressed by Pop4 epithelial and myoepithelial cells, along with the chloride channel regulator CLCA2, which is regulated by the myoepithelial lineage marker p63 and is involved in basal cell adhesion (Fig 6, boxes 3 and 4). The perivascular cell cluster contained angiopoietin 1 (ANGPT1) and connexin 40 (GJA5), which are essential for blood vessel development and maintenance (Fig 6, box 5). Finally, the leukocyte cluster contained the zeta chain of the T-cell receptor (CD247), the delta subunit of the T-cell Receptor complex (CD3D), along with the beta subunit of the IL2 receptor (IL2RB), among other leukocyte-associated genes (Fig 6, box 6). The clustering thus demonstrated high agreement among the biological replicates and identified clusters of shared genes that provided substantial evidence confirming the identity of each cell population (or pointing to its identity).

The hierarchical clustering used to compare cell types was also notably stable. Whereas Fig 6 shows the clustering of the 2,000 most differentially expressed genes, the pattern did not significantly change when we repeated the analysis with different inclusive thresholds, such as when we used the top 100, 200, 500, 1,000, or 3,000 genes that were most differentially expressed. Although the cellular relationships that emerged from these analyses were largely confirmatory, they helped us identify and characterize several populations early on. For example, before we had ascertained the identity of FACS Pop5 as vSMCs, its close clustering with related Pop6 pericytes here provided a critical clue [8]. The clustering also revealed similarities between the rare Pop4 epithelial cells and myoepithelial cells. However, it also showed that Pop4’s expression of some genes more resembled those found in luminal epithelial cells that distinguished this cell population from myoepithelial cells [8]. The molecular patterns that emerged provided information on each population and objective evidence of the relatedness among cell types. To explore these relationships—and features fundamental to each cell type—we needed to decipher their transcriptomes in finer detail.

### Distinguishing the features of cell lineage

Differentiated cells are often classified by their developmental history and the lineage from which they are derived. The different breast cells, for example, are often described as being mesenchymal (fibroblasts, pericytes, vSMC, and adipocytes), hematopoietic/blood (leukocytes, erythrocytes), endothelial (vascular and lymphatic), or epithelial derived (ER^Pos^ and ER^Neg^ luminal, myoepithelial, and Pop4 epithelial, Fig 2B). Although our primary goal was to identify the qualities that make each cell type unique, our sequencing of every breast cell type presented an unprecedented opportunity to explore the broad attributes of cell lineage through the comparative lens afforded by our data set’s comprehensive nature. We accomplished this through principal component and pathway analysis, as this approach allowed us to analyze the samples en masse—and not focus on any particular pairwise comparison prematurely. As a result, these analyses produced an astonishing amount of data. Unfortunately, we can present only a fraction of it here. However, we have provided additional tools and tables to aid the exploration of all data and results presented herein (Figs A–G in S1 Text, Supplementary Data Files 12 and 13 at 10.17605/OSF.IO/GFHC3 [9], and the Breast Cell Atlas: Transcript Finder App [10]). Below are our analyses of the first 4 principal components that revealed the distinguishing features of the 4 broad cell lineages composing breast tissues.

**Fig 7:**
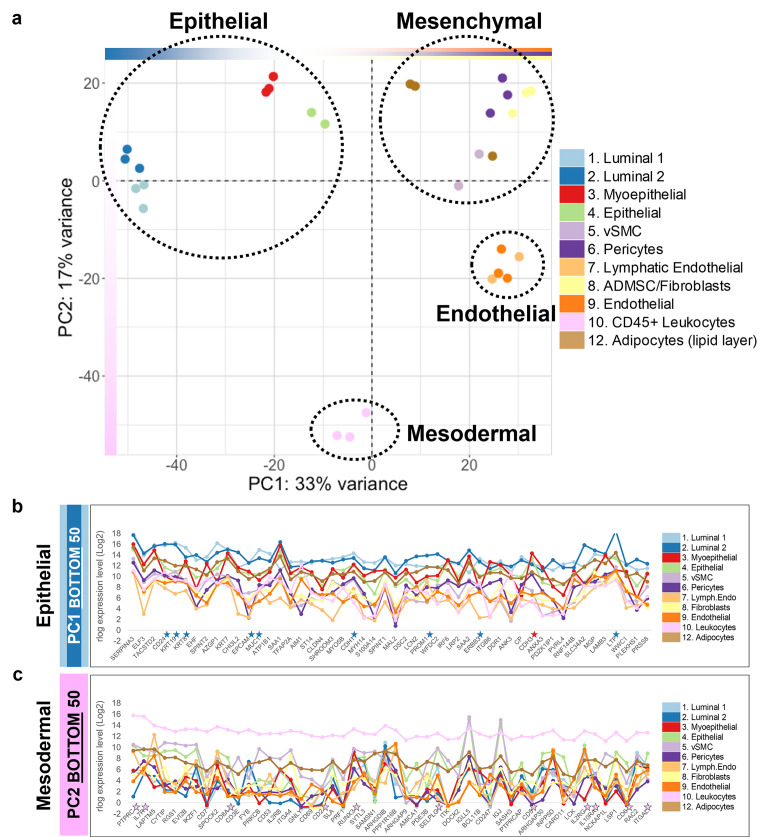
Principal component analysis. **(a)** Projection of breast cell types on principal components 1 and 2 (PC1, PC2). PC1 separates the luminal ER^Pos^ and ER^Neg^, Pop4 epithelial, and myoepithelial cell types (epithelial lineages) from the mesenchymal (fibroblasts, adipocytes, pericytes, and vSMCs) and endothelial (lymphatic and vascular) lineages. PC2 separates the CD45^Pos^ leukocytes (mesodermal lineage) from all other cell types. The cell types composing each of the epithelial, mesenchymal, endothelial, and mesodermal cell lineages are indicated (dashed circles). Data is available in S3 Data. **(b)** Median transcript levels of the 50 loading factors (genes) that contribute to the negative end of PC1 (bottom end of the list, rlog/log2 scale; Supplementary Data File 13 at 10.17605/OSF.IO/GFHC3). **(c)** Median mRNA expression of the 50 loading factors (genes) contributing to the negative end of PC2 (bottom end of the list) that separates the leukocyte samples (rlog, log2 scale; Supplementary Data File 13 at 10.17605/OSF.IO/GFHC3). Stars (★) denote commonly used markers for luminal cells (blue), myoepithelial cells (red), and leukocytes (pink). PC, principal component.

**Fig 8:**
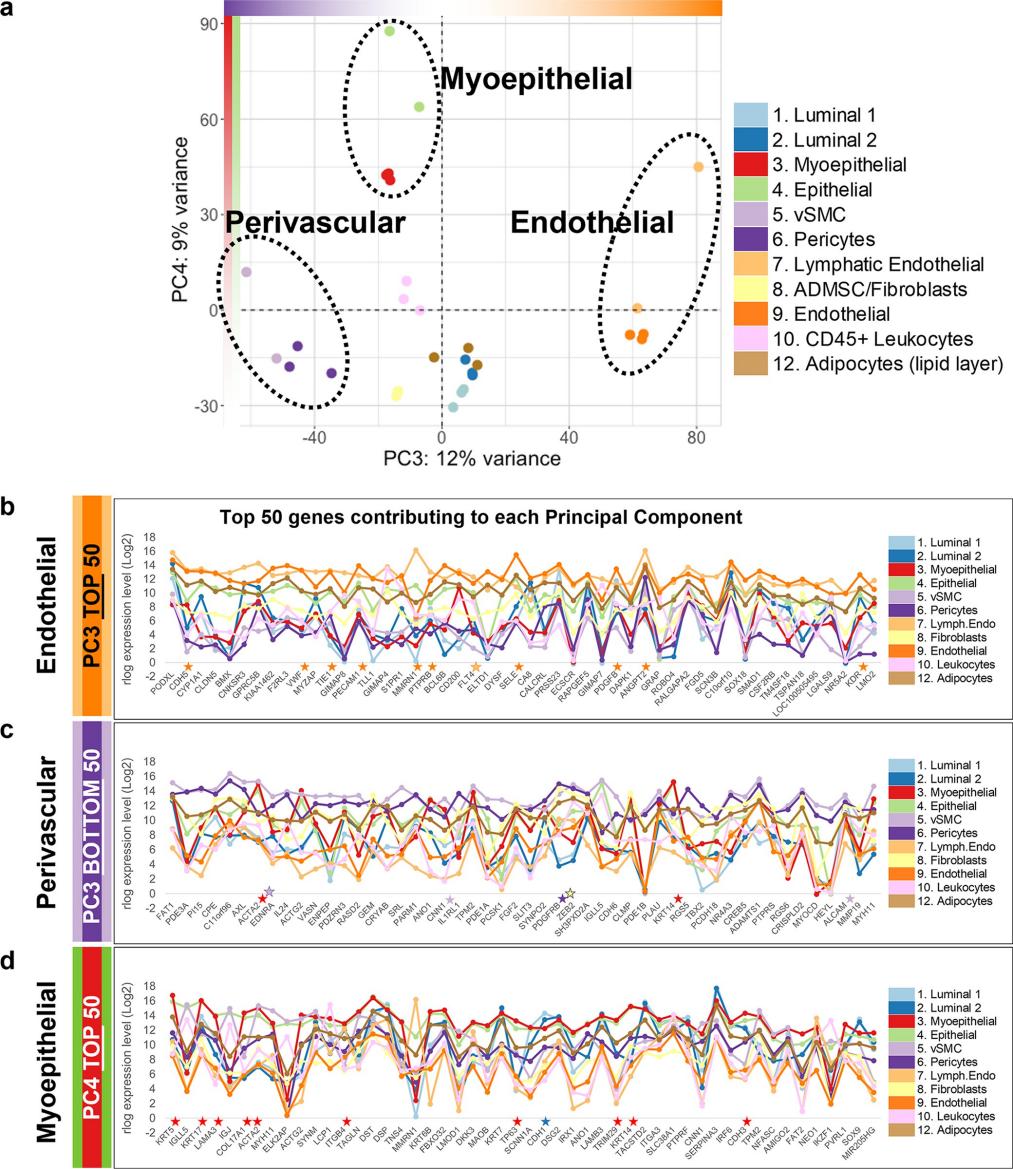
PCA plot of PC3 and PC4. **(a)** PCA projection of breast cell types on principal components 3 and 4 (PC3, PC4). PC3 separates the endothelial (most positive) and perivascular (most negative) cell types, whereas PC4 separates the myoepithelial and myoepithelial-related cell types (myoepithelial and Pop4 epithelial cells). Data can be found in S3 Data. Median mRNA expression of the 50 loading factors contributing to the **(b)** positive end of PC3 (top of the list; rlog, log2 scale; Supplementary Data File 13 at 10.17605/OSF.IO/GFHC3), **(c)** negative end of PC3 (bottom of the list; rlog, log2 scale; Supplementary Data File 13 at 10.17605/OSF.IO/GFHC3), and **(d)** the positive end of PC4 (top of the list; rlog, log2 scale; Supplementary Data File 13 at 10.17605/OSF.IO/GFHC3). Stars (★) denote common markers of endothelial cells (orange), myoepithelial cell (red), fibroblasts (yellow), pericytes (purple), and luminal cells (blue). PCA, principal component analysis.

PCA of the transcripts from all cell types showed gene expression variances were distributed across multiple principal components (PCs), with the bulk of the variation (71%) being captured by the first 4 components (Fig H in S1 Text). The projection of principal component 1 (PC1) and principal component 2 (PC2) markedly separated the 4 cell lineages in 2D space. Genes contributing to PC1 were primarily responsible for distinguishing the mesenchymal and epithelial cell types, as these lineages projected at PC1’s opposing ends (Fig 7A). Interestingly, endothelial cells—which derive from mesenchyme but are classically regarded as a specialized epithelial cell type [14,15]—projected as an independent group that aligned more closely with the mesenchymal cell types along PC1. In contrast, the variance explained by genes composing the second principal component (PC2) emerged from leukocytes, as these cells formed a single group at its distal end, far apart from the other cell types (Fig 7A). A similar analysis of principal components 3 and 4 resolved the endothelial cells and 2 subtypes of the mesenchymal and epithelial lineages, i.e., perivascular cells and myoepithelial/Pop4 epithelial cells (Fig 8A).

To gain further insights into the distinguishing attributes shared by members of each cell lineage, we examined the genes impacting their PCA groupings, i.e., the loading factors with the highest influence on each principal component (S3 Data). We then used these unique gene lists to probe functionally grouped Gene Ontology [16] and Reactome [17] pathway annotation networks [18].

#### Epithelial lineage. ER^Pos^ and ER^Neg^ Luminal cells, myoepithelial cells, Pop4 epithelial cells

**Fig 9:**
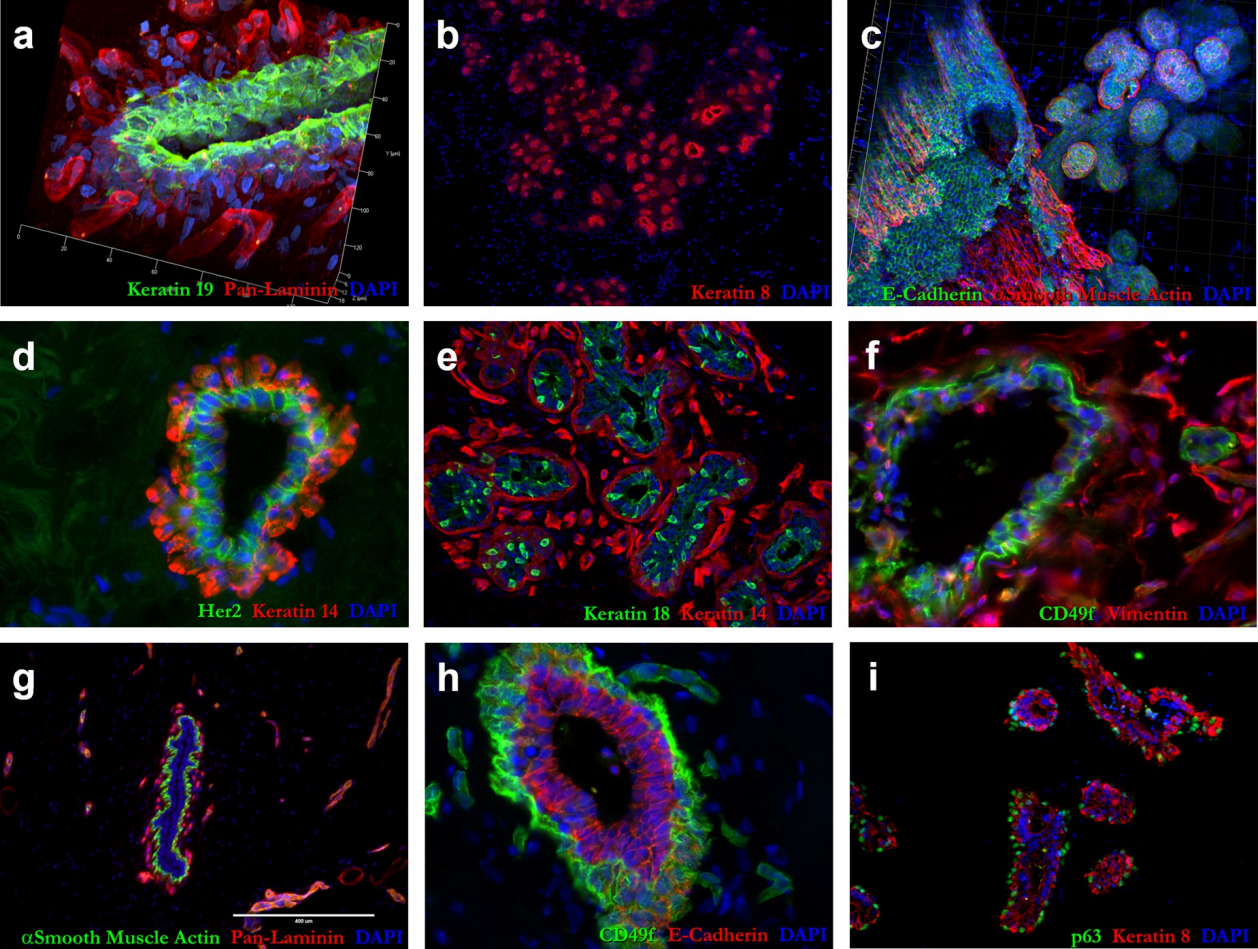
IHC validation of PCA markers. Tissue immunostaining of normal human breast tissue with markers contained in the principal components. **(a)** Keratin 19 (green) and pan-laminin (red); **(b)** keratin 8 (red); **(c)** e-cadherin (green) and alpha smooth muscle actin (red); **(d)** Her2 (green) and keratin 14 red); **(e)** CD49f (green) and vimentin (red); **(f)** alpha smooth muscle actin (green) and pan-laminin (red); **(g)** CD49f (green) and e-cadherin (red); and **(h)** p63 (green) and keratin 8 (red). Loading factors at the negative end of PC1 (epithelial end) included: keratin 19/Krt19 (#5 on list), keratin 8/KRT8 (#6), E-cadherin/CDH1 (#22), Her2/ERBB2 (#85), and keratin 18/KRT18 (#138), and CD49f/ITGA6 (#5574). Loading factors at the positive end of PC1^No Endo^ (mesenchymal end) included: smooth muscle actin/ACTA2 (#155 on the list), and Vimentin/VIM (#192). Loading factors at the negative end of PC3 (perivascular end) included: smooth muscle actin/ACTA2 (#7 on the list). Loading factors at the positive end of PC3 (endothelial end) included: CD49f/ITGA6 (#344). Loading factors at the positive end of PC4 (myoepithelial end) included: smooth muscle actin/ACTA2 (#7 on the list), P63/TP63 (#25), and CD49f/ITGA6 (#68). PCA, principal component analysis.

We first analyzed the cluster defined by the epithelial lineages: the ER^Pos^ and ER^Neg^ luminal myoepithelial and Pop4 epithelial cell types (Fig 2B). Based on their positioning on PC1, we expected the genes with the highest contributions to PC1 to reflect those that distinguish the epithelial and mesenchymal lineages, and in particular, highlight genes associated with luminal epithelial cells—as the ER^Pos^ and ER^Neg^ luminal cells projected the farthest along on PC1. Analysis of gene expression in each cell type showed this was indeed the case (Fig 7B). Many of PC1’s top genes (at the negative epithelial end) were recognizable, as they are often used as markers of the epithelial lineage. They included keratins 6b, 8, 18, and 19, P- and E- cadherins, claudin 4, and Her2 (Figs 7B and 9A–9E and S3 Data). Other familiar genes included integrin beta 6, the transcription factors ELF3, EHF, and TFAP2A; peptidase inhibitors SERPINA3 and SPINT2; serine protease ST14, cytoskeletal and associated proteins SHROOM3, MYO5D, and MYH14; the calcium-binding protein S100A14, among others (S3 Data). These genes were among the 200 extracted from the negative epithelial end of PC1—a gene list we used to identify the biological pathways enriched within the epithelial cell lineage.

**Fig 10:**
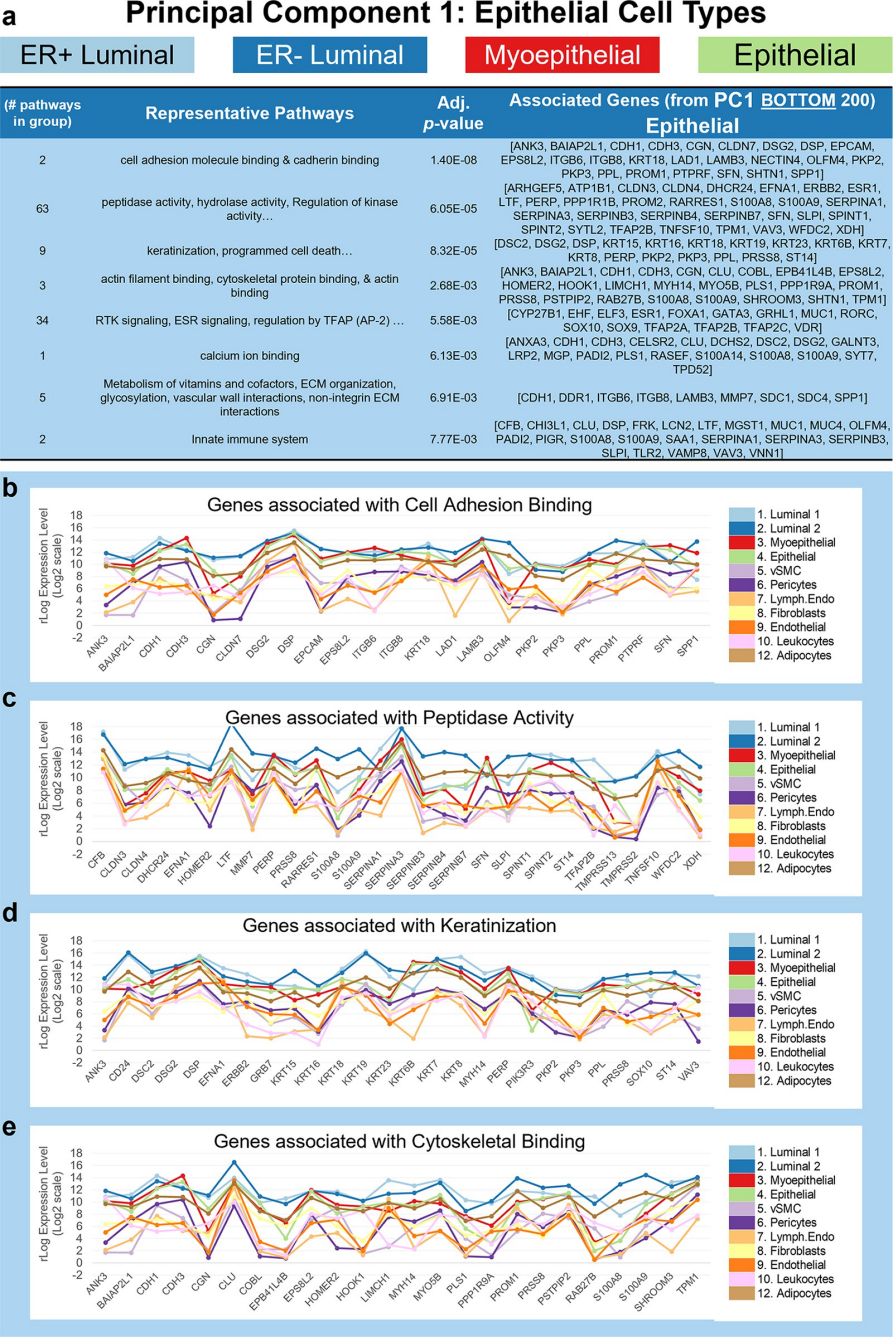
Pathway analysis of epithelial genes (PC1). **(a)** Grouped GO/Reactome terms overrepresented within the gene list 200 genes contributing to the negative/epithelial end of PC1 (bottom of the list) are ordered by adjusted *p*-value. Grouping was done by the similarity of terms to evaluate broad differences in cell class. Associated genes for each group are listed in the fourth column. Median mRNA expression (rlog, log2 scale) for PC1 genes associated with select pathways including **(b)** cell adhesion and binding, **(c)** peptidase activity, **(d)** keratinization, and **(e)** cytoskeletal binding. (The values for each graph can be found in Supplementary Data File 13 at 10.17605/OSF.IO/GFHC3.) PC, principal component.

Using the epithelial gene set, we performed a two-sided hypergeometric (enrichment/depletion) test to determine if particular molecular functions or biological pathways were disproportionately represented (Gene Ontology [19], Reactome Pathway database [17]). We found many were, and we aggregated the results to form a functionally organized pathway network, which grouped similar biological processes based on their shared genes (ClueGO [18]). The 124 enriched gene sets aggregated into 24 distinct groups related to cell adhesion, peptidase activity, keratinization, estrogen signaling, AP-2 transcription factor regulation, mucins, and innate immune system factors (Fig 10A and S4 Data: PC1 Epi tab). The most significant group (*p*-value = 1.4 × 10^−8^) contained the cell adhesion molecule binding and cadherin binding Gene Ontology terms. These gene sets represent different levels of the same ontology hierarchy and are highly correlated. For example, they both contain components of adherens junctions, i.e., epithelial- and placental-cadherins (CDH1 and CDH3), cingulin (CGN), and 348 other genes. By comparison, the cell adhesion gene set contains an additional 196 genes related to other aspects of cellular adhesion, such as desmosome-associated desmoplakin (DSP) and desmoglein 2 (DSG2, S3 Data, sorted by PC1). The algorithm’s ability to cluster overlapping gene sets like these, and assemble them into related clusters, allowed us to extract the common biological themes obscured within the volume and complexity of the data. Reassuringly, when we examined the expression of the genes in these pathways, we found they were indeed elevated in the 4 epithelial cell types (Fig 10B–10E and S4 Data: PC1 Epi tab). These enriched pathways and themes describe general features of the epithelial lineage. After exploring the remaining cell lineages’ shared attributes (defined by each PC), we will revisit these cells and explore their individual traits.

#### Leukocytes. CD45^Pos^ Cells

The second principal component separated the leukocytes (CD45^Pos^ FACS population), and analysis of PC2’s loading factors revealed many well-known leukocyte markers (Fig 7A and 7C). For example, “protein tyrosine phosphatase receptor type C” (PTPRC), the gene encoding the pan-leukocyte marker CD45—which we used to FACS isolate the leukocytes—was notably the first gene on the list. Other top genes included the CD antigens: CD2, CD3E, CD6, CD7, CD8A, and others. Also present was the transcription factor RUNX3, which is critical for T-cell differentiation, as was selectin P ligand (SELPLG), which is crucial for leukocyte trafficking. Using the same technique described above (for PC1 and the epithelial cells), we extracted the top genes from the leukocyte-defining end of PC2 and performed pathway enrichment analysis. We identified 453 enriched gene sets that clustered into 62 distinct thematic groups (S4 Data: PC2 Leukocyte tab). Representative pathways from the 10 most significant groups were: adaptive immune response, activation of immune response, leukocyte homeostasis, T-cell activation, leukocyte chemotaxis, phagocytosis, IL-6 production, acute inflammatory response, regulation of GTPase activity, mast cell degranulation, and monocyte differentiation (*p-*values ranged between 6.07 × 10^−46^–9.53 × 10^−11^, Fig J in S1 Text). Although most leukocytes in the breast are macrophages [8], the sequenced mRNAs from the CD45^Pos^ FACS gate represent a diverse population of white blood cells. Therefore, finding a broad range of enriched pathways attributable to different leukocyte subpopulations was expected. When we compared the expression of enriched genes in each cell type, we found they were indeed highly expressed by the Pop10 leukocytes (Fig J, panels B–D in S1 Text).

#### Mesenchymal lineage. vSMCs, pericytes, fibroblasts, adipocytes

Similar gene expression patterns within the 4 mesenchymal cell types positioned this cell group toward the positive end of PC1’s axis in the 2D projection of PC1 and PC2 (Fig 7A). However, endothelial cells were also located in this region, indicating they and mesenchymal cells share the distinct expression of many genes. The relatively similar projection of endothelial and mesenchymal cells along PC1 prevented extraction of loading factors that could be solely attributable to either cell class. As we were primarily interested in identifying the defining characteristics of mesenchymal cells at this stage, we digitally removed the endothelial cell data and repeated the PCA. The new PCA projection resolved the mesenchymal cells, situating them at the positive end of the first principal component in this ancillary graph (PC1^No ENDO^, Fig I, panel A, in S1 Text). Exploring the loading factors of this new principal component, we confirmed that these genes were indeed highly expressed by the mesenchymal cells. For example, vimentin (VIM), an intermediate filament protein and classical mesenchymal marker (Fig 9F), was near the top of the list, at position 192 (out of 21,560 genes, Fig I, panel B, in S1 Text). The top loading factor was the non-selective receptor for endothelins (EDNRB). Endothelins are potent vasoactive peptides, and EDNRB expression was notably highest in the perivascular cell types (vSMC and pericytes)—these cells surround blood vessels and regulate vessel diameter by influencing vasoconstriction and dilation. Genes highly contributing to PC1 included many collagen family members (e.g., COL4A1, COL1A2, COL6A3, COL4A2, COL3A1, COL15A1, COL5A3, and others). Also present were ADAM metalloproteinases, the transcription factors ZEB1and ZEB2, platelet-derived growth factor receptor beta (PDGFRB), insulin-like growth factor II, laminin subunit alpha-4 (LAMA4), interleukin 24 (IL24), chondroitin sulfate proteoglycan 4 (CSPG4), along with many other notable genes (Fig I in S1 Text and S3 Data: PC1^NoEndo^). To understand how these genes collectively contribute to mesenchymal cells, we performed pathway analysis using the loading factors from the mesenchymal-defining end of PC1^No ENDO^.

**Fig 11:**
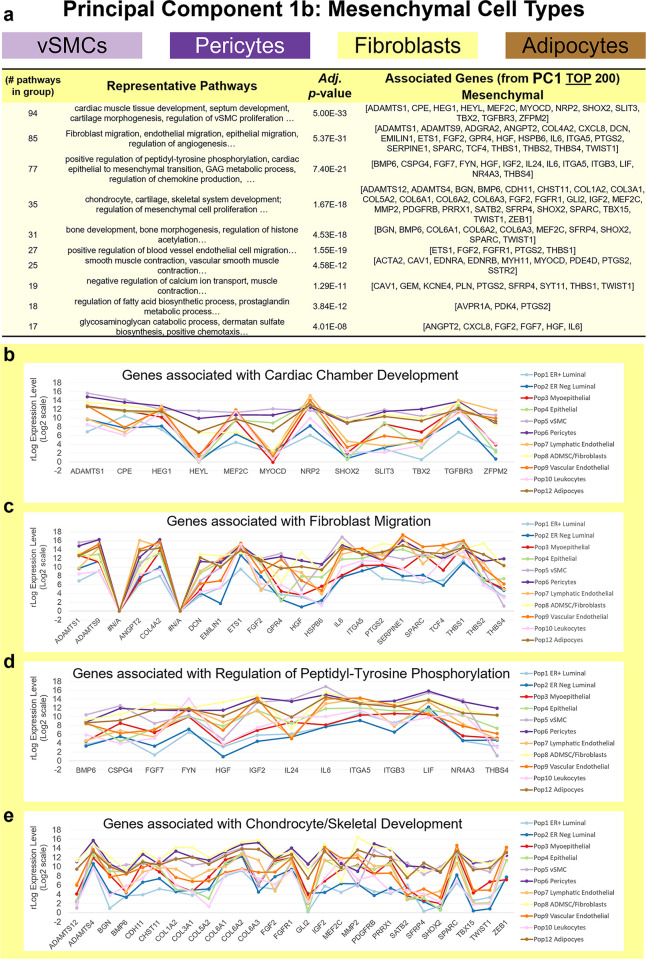
Pathway analysis of mesenchymal genes (PC1^No Endo^). **(a)** Grouped GO/Reactome terms overrepresented within the gene list 200 genes contributing to the positive/mesenchymal end of PC1^NoEndo^, ordered by adjusted *p*-value. Median mRNA expression (rlog, log2 scale) for PC1^NoEndo^ genes associated with select pathways, including **(b)** cardiac tissue development, **(c)** fibroblast migration, **(d)** regulation of peptidyl-tyrosine phosphorylation, and **(e)** chondrocyte/skeletal development. (The values for each graph can be found in Supplementary Data File 13 at 10.17605/OSF.IO/GFHC3.) PC, principal component.

Pathway enrichment using 200 of PC1’s most critical mesenchymal genes identified 337 enriched gene sets from the Reactome and Gene Ontology databases (*p*-values <0.05). These pathways assembled into a network of 96 distinct thematic groups (S4 Data: PC1 Mesenchymal tab). The most significant group (*p*-value = 5 × 10^−33^) contained 94 different aggregated gene sets/pathways that dealt mainly with: cardiac development (chamber, muscle, septum), cartilage morphogenesis, smooth muscle proliferation, bone development, and chondrocyte differentiation—pathways that are consistent with our primary cultures showing the isolated breast fibroblasts have multi-lineage differentiation potential [8]. The defining genes in this group included TBX2 and HEYL transcription factors, the type III TGFβ receptor TGFBR3, fibroblast growth factor FGF2, ADAM metallopeptidase with thrombospondin type 1 motif 1a and 9 (ADAMTS1, ADAMTS9), PDGFRB, and others (Fig 11A). Representative pathways from the other top significant groups included: Fibroblast migration, GAG metabolic process, chondrocyte development, bone development, smooth muscle contraction, negative regulation of calcium ion transport, regulation of fatty acid biosynthetic process, and dermatan sulfate biosynthesis, with *p-*values ranging between 5.00 × 10^−33^ to 4.01 × 10^−08^ (Fig 11A and S4 Data: PC1 Mesenchymal tab).

While the 4 mesenchymal cell types highly expressed genes in the enriched pathways, transcript levels were not always uniform across these cell types (Fig 11B–11D). For example, in the cardiac chamber development gene set, expression of the pathway’s genes was consistently higher in the perivascular cell types. The heart is composed of mesenchymal cardiac muscle cells and is a highly vascularized tissue that requires perivascular support, which may explain the frequent enrichment of cardiac-related pathways within the mesenchymal gene set (Fig 11—cardiac chamber development). The enrichment with these and other tissue-related gene sets (cartilage, bone, muscle, and adipose) indicates a deeply rooted bond among mesenchymal cells and common dependence on a core set of genes. Later, we will use specific pairwise comparisons to explore the distinguishing features of the different mesenchymal cell types.

#### Endothelial lineage. Lymphatic and vascular endothelial cells

To identify the genes and processes responsible for shaping the unique characteristics of endothelial cells, we explored the third and fourth principal components. These components resolved the vascular and lymphatic endothelial cells as a distinct group near the positive end of PC3 (Fig 8A). Many genes contributing to the endothelial end of PC3 were familiar, such as vascular-endothelial cadherin (CDH5)—a prominent endothelial adhesion molecule (Fig 8B). We also observed von Willebrand factor (VWF); the angiopoietin regulator tie1 (TIE1); the endothelial marker CD31 (PECAM1); endothelial selectin (SELE); angiopoietin 2 (ANGPT2); as well as vascular endothelial growth factor receptors KDR and FLT4, and many other notable genes. The top gene on the list was “podocalyxin like” (PODXL), which binds leukocyte selectin and is vital to lymphocyte-endothelial adhesion. Also present was Claudin 5, which is crucial for endothelial cell junctions. Finding so many genes with known importance to endothelial cells corroborated these genes’ notable contributions to endothelial cells and showed they are characteristics of both vascular and lymphatic cell types. At the same time, the analysis identified other influential genes that are perhaps less appreciated or whose importance to endothelial cell function are not as well characterized (listed in Fig 9 and S3 Data). To explore the biological contribution of these genes to endothelial cell biology, we performed pathway enrichment analysis.

**Fig 12:**
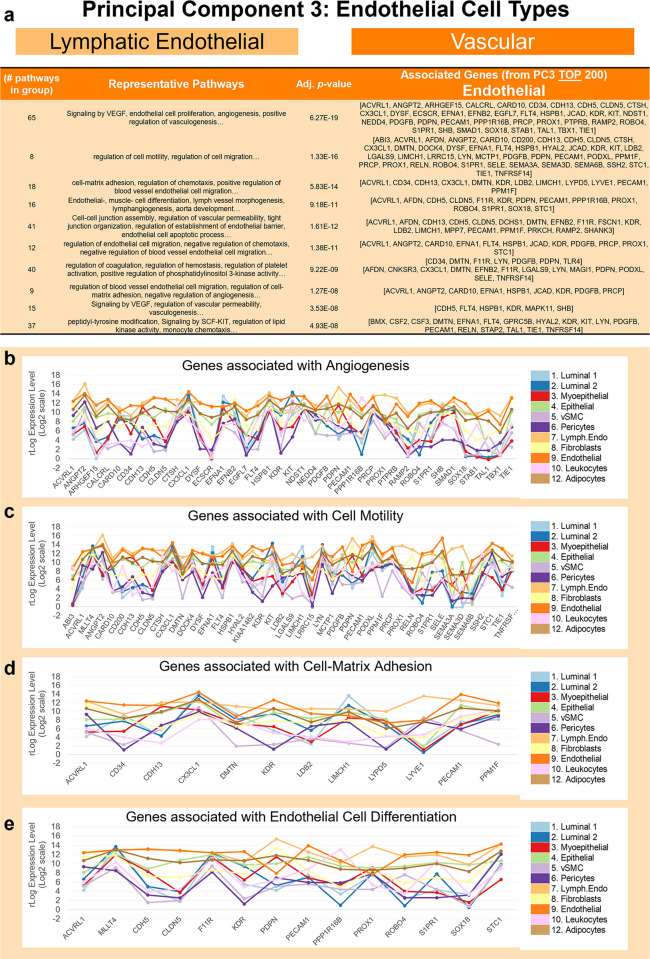
Pathway analysis of endothelial genes (PC3). **(a)** Grouped GO/Reactome terms associated with the 200 genes contributing to the positive end of PC3 (top of list), ordered by adjusted *p*-value. Median mRNA expression (rlog, log2 scale) for PC3 genes associated with select pathways including **(b)** angiogenesis, **(c)** cell motility, **(d)** cell-matrix adhesion, and **(e)** endothelial cell differentiation. (The values for each graph can be found in Supplementary Data File 13 at 10.17605/OSF.IO/GFHC3.) PC, principal component.

Hypergeometric analysis of the endothelial genes from PC3 identified 650 enriched biological pathways (*p-*values <0.05, S4 Data: PC3 Endothelial tab). These pathways assembled into 61 groups based on the genes they shared. Not surprisingly, most groups pertained to blood vessel and circulatory system biology (Fig 12A). The most significant group (*p-*value = 6.27 × 10^−19^) involved cell motility and chemotaxis and contained ameboidal-type cell migration, angiogenesis, and signaling by VEGF gene sets (Fig 12A and 12B). As expected, PC3-identified genes within these enriched pathways were highly and differentially expressed by the lymphatic and endothelial cell types (Fig 12B–12D). Representative pathways from these groups included: cell-matrix adhesion, endothelial- and muscle-cell differentiation, lymph vessel morphogenesis, regulation of establishment of endothelial barrier, regulation of coagulation, vasculogenesis, and monocyte chemotaxis. Many notable genes were present in these pathways, including podoplanin (PDPN)—a marker we used to sort lymphatic endothelial cells—ephrins A1 and B2 (EFNA1 and EFNB2), activin a receptor like type 1 (ACVRL1), platelet-derived growth factor subunit b (PDGFB), along with the aforementioned cadherin 5, claudin 5 and the VEGF receptors (KDR and FLT4). Finding vascular-related pathways enriched in the endothelial data set was not surprising, but identifying these pathways was reassuring, as it validated the approach and indicated these processes were occurring in both lymphatic and vascular endothelial cell types found in the breast.

Outside of the anticipated pathways, enrichment analysis of the endothelial genes also uncovered: Signaling by SCF-KIT (stem cell factor/c-kit), adherens junction organization, regulation of peptidyl-tyrosine phosphorylation (JAK/STAT), semaphorin receptor binding, interleukin-6 secretion (and IL-10, IL-12 cytokines), aminoglycan biosynthetic process, hyaluronic acid binding, along with roughly 500 other enriched processes and pathways (*p-*values ranging between 1.64E-06 and 2.08E-02; S4 Data: PC3 Endothelial tab). Enrichment of “Signaling by SCF-KIT” pathway was driven by elevated endothelial expression of the kit tyrosine kinase receptor (KIT), the GRB2-Related Adapter Protein (GRAP), and LYN Proto-Oncogene (LYN). Notably, Kit’s ligand, stem cell factor (KITLG), was differentially expressed between endothelial cell types, as vascular endothelial cells expressed it at a much higher level (40-fold higher) than lymphatic endothelial cells (Fig K in S1 Text).

Endothelial semaphorin expression was intriguing. Semaphorins are a large family of signaling ligands that exist as either secreted or membrane-associated proteins which provide movement and guidance cues [20]. The Semaphorin receptor-binding pathway was enriched predominantly because of endothelial expression of semaphorin 3a, 3d, and 6b (SEMA3A, SEMA3D, SEMA6B, *p-*value = 1.47 × 10^−3^; Fig L and S4 Data: PC3 Endothelial tab). The class 3 semaphorins are the only semaphorins in humans that are secreted, and we found that both SEMA3A and SEMA3D were expressed rather exclusively by lymphatic endothelial cells (Fig L, panel A, in S1 Text). Interestingly, the co-receptor for sema3 proteins, neuropilin1 and 2 (NRP1 and NRP2), which bind VEGF family members, were highly expressed by the endothelial cell types (*p-*values of 3.13 × 10^−27^ and 1.23 × 10^−53^, respectively), with vascular endothelial cells (and pericytes) differentially expressing the highest levels of NRP1. In contrast, the highest levels of NRP2 occurred in lymphatic endothelial cells, which was 2-fold higher than in their vascular counterparts (Fig L, panels B and C, in S1 Text). Others have implicated NRP2 as a critical factor in lymphangiogenesis [21,22], and our results are consistent with it fulfilling such a role for breast lymphatic cells.

The above analysis has identified many features shared between lymphatic and vascular endothelial cells. Later, we will directly compare these 2 endothelial cell types and explore their individual traits.

#### Perivascular sub-lineage. vSMC and Pericytes

After analyzing the endothelial cells, we were left with 2 other cell groups to explore that were resolved by PC3 and PC4. Perivascular cells were the first of these (Fig 8A). This mesenchymal cell subclass includes pericytes and vSMCs, and the group projected toward the negative end of the third principal component, opposite to the endothelial cells. Like fibroblasts and adipocytes, perivascular cells are mesenchymal in nature; but perform critical functions to the blood vessels they envelop. PC3’s loading factors (at the perivascular end) included genes with known pericyte and vSMC significance (Fig 8C), such as smooth muscle actin (ACTA2, Fig 9G), smooth muscle calponin (CNN1), glutamyl aminopeptidase (ENPEP), regulator of G-protein signaling 5 (RGS5), and PDGFRB. At the top of the list was the atypical cadherin FAT1, along with phosphodiesterase 3A (PDE3A), peptidase inhibitor 15 (PI15), and carboxypeptidase E (CPE, Fig 8C). Both perivascular cell types differentially expressed AXL receptor tyrosine kinase, which was sixth on the list and, in addition to its other physiological functions, serves as a receptor/co-receptor for many viruses, including Ebola, Lassa, Marburg [23], SARS-CoV-2 [24], and Zika viruses [25]—which may help explain the vessel involvement often observed in infected individuals. The perivascular cells also notably and differentially expressed members of the Notch and Hedgehog signaling pathways, such as NOTCH3, HEYL, GLI2, and GLI3, suggesting these cell types fulfill fundamental roles during tissue morphogenesis and maintenance. As before, we used the top 200 genes from the defining principal component for pathway enrichment analysis.

**Fig 13:**
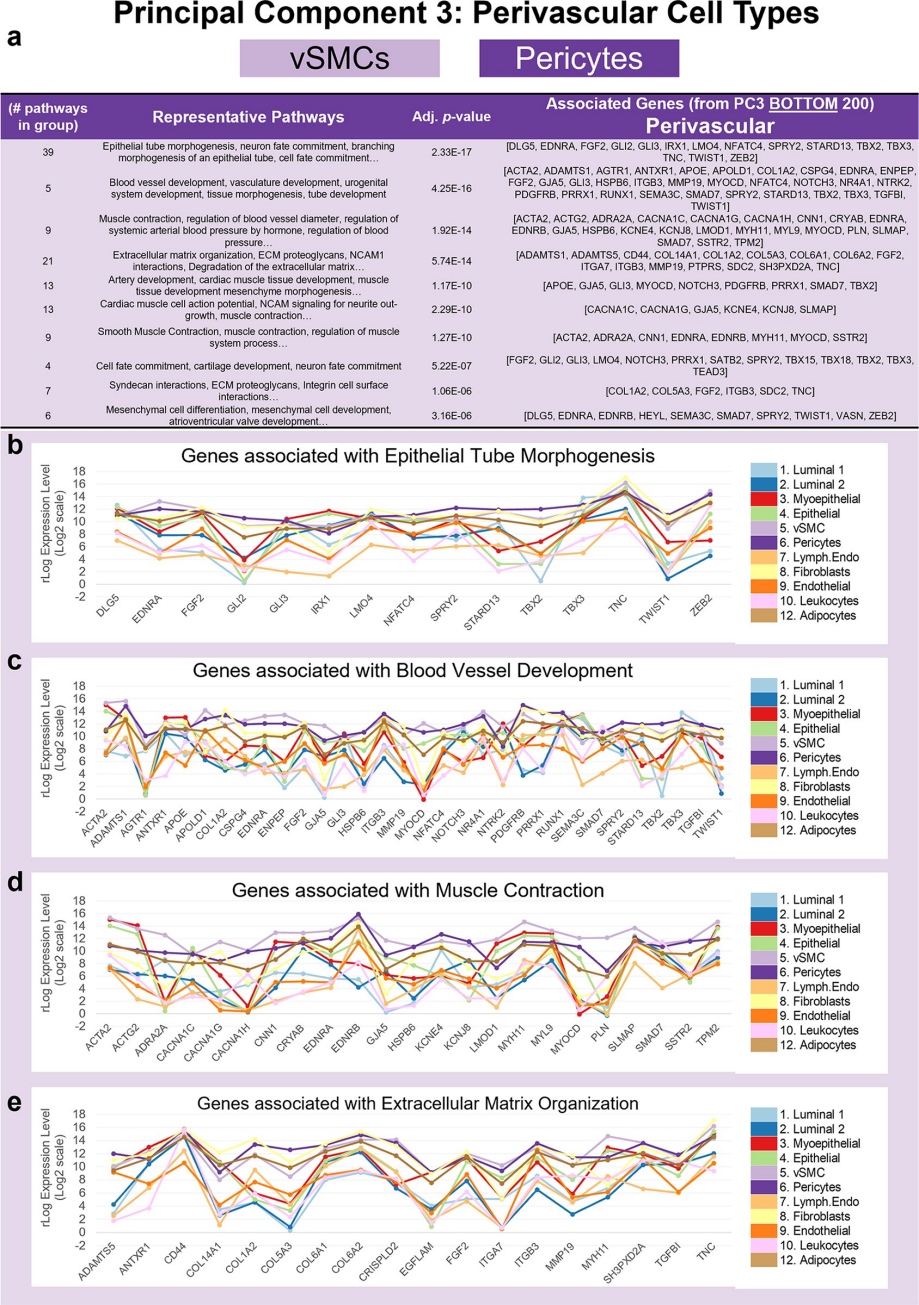
Pathway analysis of perivascular genes (PC3). **(a)** Grouped GO/Reactome terms associated with the 200 genes contributing to the negative end of PC3 (bottom of the list), ordered by adjusted *p*-value. Median mRNA expression (rlog, log2 scale) for PC3 genes associated with select pathways, including **(b)** epithelial tube morphogenesis, **(c)** blood vessel development, **(d)** muscle contraction, and **(e)** extracellular matrix organization. (The values for each graph can be found in Supplementary Data File 13 at 10.17605/OSF.IO/GFHC3.) PC, principal component.

Hypergeometric analysis of the perivascular genes identified 456 enriched processes and pathways from the Gene Ontology and Reactome databases (*p-*values <0.05, S4 Data: PC3 Perivascular tab). These aggregated into 24 distinct groups (Fig 13A). Many groups reflected vascular-related biology in line with known perivascular cell functions. Among these were regulation of blood vessel diameter, regulation of systemic arterial blood pressure by hormone, and smooth muscle contraction. Enriched pathways from other significant groups included: Epithelial tube morphogenesis, blood vessel- and vascular-development, extracellular matrix (ECM) proteoglycans, artery- and cardiac tissue-development, cell fate commitment, syndecan interactions, and mesenchymal cell differentiation (Fig 13B–13E). These analyses identify characteristic processes of both perivascular cell types.

#### Myoepithelial sub-lineage. Myoepithelial cells and Pop4 epithelial cells

The final cell group separated by PC3 and PC4 was an epithelial subclass comprised of myoepithelial cells and related Pop4 epithelial cells (a rare cell type we identified during FACS panel development [8]). The fourth principal component resolved these cells, and when we investigated PC4’s loading factors, we found that the most significant gene was none other than keratin 5 (KRT5). Finding keratin 5 was reassuring because it has a long and storied history as a marker of basal epithelial cells [26–28]. Another top gene was alpha-6 integrin (ITGA6/CD49f), which we anticipated because CD49f formed the backbone of our FACS panel—and myoepithelial cells consistently exhibited the highest CD49f staining intensities (Figs 1A, 9F, and 9H). Among the many genes contributing significantly to PC4 (Fig 8D), included the well-known myoepithelial markers: keratins 14 (KRT14, Fig 8D) and 17 (KRT17), laminin subunit alpha-3 (LAMA3), the alpha-1 chain of collagen type XVII (COL17A1), smooth muscle actin (ACTA2, Fig 9G), p63 (TP63, Fig 9I), E-cadherin (CDH1, Fig 9C and 9H), P-cadherin (CDH3), the transcriptional regulatory factor TRIM29, and integrin subunit beta 4 (ITGB4, Fig 8D and S3 Data). Many of these myoepithelial genes notably contribute to the molecular cluster defining the basal-like breast cancer subtype—which provided the rationale for its “basal-like” designation [29].

**Fig 14:**
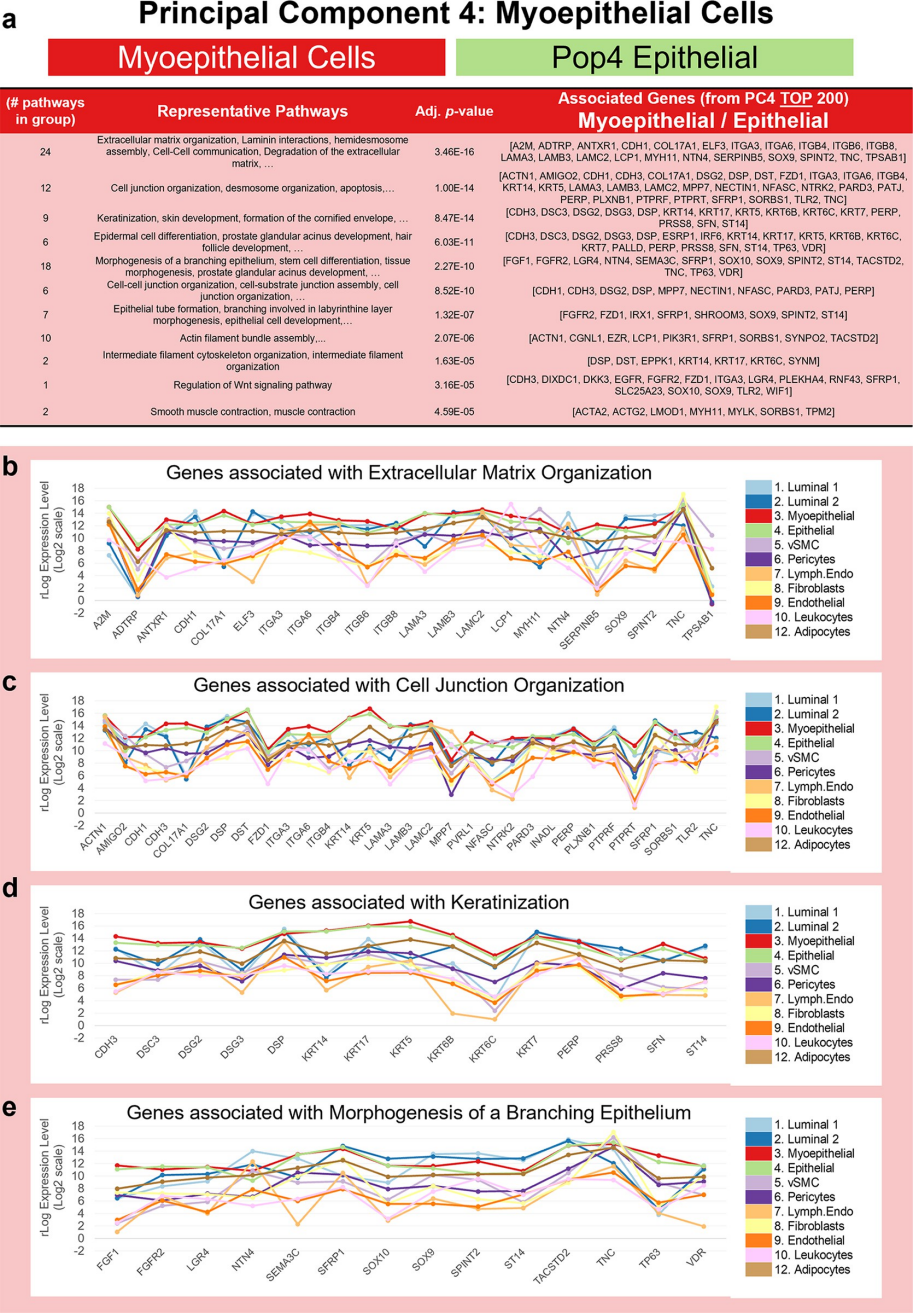
Pathway analysis of myoepithelial-related genes (PC4). **(a)** Grouped GO/Reactome terms associated with the 200 genes contributing to the positive end of PC4 (top of the list), ordered by adjusted p-value. Median mRNA expression (rlog, log2 scale) for PC4 genes associated with select pathways, including **(b)** extracellular matrix organization, **(c)** cell junction organization, **(d)** keratinization, and **(e)** morphogenesis of branching epithelium. (The values for each graph can be found in Supplementary Data File 13 at 10.17605/OSF.IO/GFHC3.) PC, principal component.

As with the preceding cell lineages, we extracted the top 200 loading factors from the defining end of the principal component (PC4, in this case) and used the gene list to explore potentially enriched pathways and processes. Pathway enrichment identified 333 gene sets, which aggregated into 24 groups of related biological functions (S4 Data: PC4 Myoepithelial (MEP) tab). Processes related to the basement membrane, extracellular matrix organization, and laminin interactions led the way, forming the most significantly enriched group (*p*-value = 3.46 × 10^−16^, Fig 14A). These gene sets were dominated by integrins (ITGA3, ITGA6, ITGB4, ITGB6, ITGB8) and laminin chains (LAMA3, LAMB3, LAMC2), which were highly expressed by myoepithelial cells (Fig 14B and 14C). Notably, these 3 laminin genes encode the a3, b3, and g2 chains that form the heterotrimeric laminin 332 molecule—an essential epithelial basement-membrane-specific variant that reinforces the cellular adhesion to the basal lamina [30].

Representative pathways from the other significantly enriched groups included: cell junction organization, keratinization and skin development, prostate glandular acinus development, morphogenesis of a branching epithelium, epithelial tube formation, actin filament bundle assembly, smooth muscle contraction, intermediate filament organization, regulation of Wnt signaling pathway, and others (Fig 14A–14E and S4 Data: PC4 Myoepithelial tab). Among the many integrin, laminin, keratin, and collagen genes represented in these enriched pathways were cadherins CDH1 and CDH3, desmosome-related desmocollin 3 (DSC3) and desmogleins DSG2 and DSG3, stratafin (SFN, 14-3-3 sigma); wnt-modulators “secreted frizzled-related protein 1” (SFPR1) and “Dickkopf wnt signaling pathway inhibitor 3” (DKK3); epidermal growth factor receptor (EGFR), fibroblast growth factor 1 (FGF1), and others (S4 Data: PC4 Myoepithelial tab).

Myoepithelial cells have historically been the most studied breast cell type, likely because of their prevalence in the tissue and ease of culture. Our results are consistent with prior knowledge of myoepithelial function: that these cells are basally located, help shape the basement membrane to which they attach via hemidesmosomes and are rich with smooth muscle actin and myosin that provide them with contractile ability. Furthermore, the mammary gland is formed through invagination of the skin, and the myoepithelial gene list reflects this connection, as it contains genes associated with epidermal development. This list includes basal keratins (KRT-14, -17, -5, -6b, -6c, and -7), the laminin 332 chains, desmosomal genes, vitamin D receptor (VDR), and transcription factors SOX9 and p63. These attributes distinguish myoepithelial cells from all others in the breast. Our results pinpoint the specific factors produced by myoepithelial cells, affirming their pivotal role in ECM-mediated cellular functions.

This section highlights the broad characteristics of cell lineages composing the breast. These characteristics emerged from pathway enrichment analyses using gene lists defined by the principal components aligning and separating each cell lineage. Hundreds of pathways were revealed. Some we anticipated, whereas many others were not. Although we have here highlighted the most statistically significant pathways emerging from this analysis (Figs 10–14), many more exist and are provided in the tabulated results of the pathway enrichment analyses (S4 Data). A full review of these results will undoubtedly provide a more comprehensive perspective.

### Gene families contributing to cellular identity

Above, we explored the distinguishing qualities of different cell lineages by determining the pathways enriched in PCA-defined gene lists. These analyses relied on curated gene sets from the Gene Ontology (GO) and Reactome databases—but not every human gene is represented in these archives. For example, the Reactome knowledgebase currently contains 11,291 human proteins (including known isoforms) that, by our count, are encoded by just over 9,300 genes. Because we measured over 20,000 genes in each cell type, many are not captured in the above analysis. Therefore, we have complemented the prior results by shifting to a more “gene-centric” approach. This method entailed identifying all differentially expressed (DE) genes in the breast cell data set (DESeq2 [12], adj. *p*-value ≤0.1, S5 Data). We then used this information to identify gene families with the most variably expressed genes. Due to their differential expression, these gene types must contribute to cell-specific processes and the unique features of each cell type. Their identification and patterns of expression provided countless insights.

The HUGO Gene Nomenclature Committee has organized 25,320 human genes into 1,519 manually curated gene families (HGNC gene group index [31,32]). When we explored the breast cells’ transcriptomes, we identified 1,421 gene families in our data set (S6 Data). These included vacuolar-type ATPases, matrix metallopeptidases, mucins, collagens, glutathione transferases, erb-B2 tyrosine kinases, histones, interleukins, and more (S6 Data). The sizes of the families varied widely, from 1 to 831 genes ($\bar{x}=14,sd=42.2$) with a median family size of 6 genes per family (Fig M in S1 Text).

**Fig 15:**
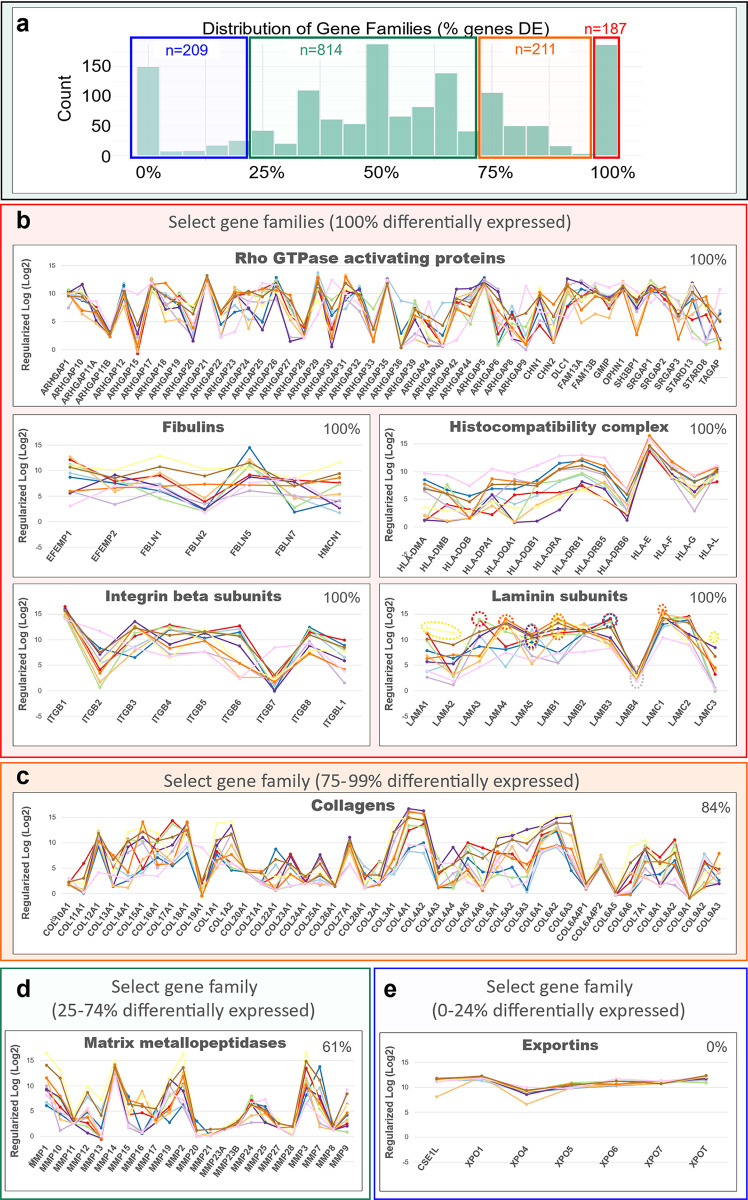
Gene types contributing to cell-specific features. Of the 1,519 types of genes (gene families) defined by HGNC, we found 1,421 gene families (of all sizes) expressed within the breast cells transcriptomes. **(a)** Distribution of the proportions of DE genes in each gene family (BH Adj. *p-*value ≤0.1). The proportion of DE genes in these families ranged between 0% and 100%, which we grouped into different bins: 0%–24% (blue, *n* = 209), 25%–74% (green, *n* = 814), 75%–99% (orange, *n* = 211), and 100% (red, *n* = 187). **(b–e)** Median transcript levels (rlog, log2 scale) across all cell types of genes composing select families with **(b)** 100% DE genes, **(c)** 75%–99% DE genes, **(d)** 25%–74% DE genes, and **(e)** 0%–24% DE genes. (The values for each graph can be found in Supplementary Data File 13 at 10.17605/OSF.IO/GFHC3.) DE, differentially expressed.

To determine the families with the most variably expressed genes, we examined the expression of each gene, from each family, within every breast cell type—and focused on families that had 6 or more genes (≥median size). We discovered 187 gene families composed entirely of differentially expressed genes (Fig 15A). Twenty of these families remained once we excluded those containing fewer than 6 genes. These included Rho GTPase activating proteins, fibulins, histocompatibility complex genes, laminin subunits, integrin beta subunits, and more (Fig 15B and S6 Data). The cellular processes affected by genes in these 20 families are wide-ranging, as they are involved in Rho signaling, cAMP regulation, interactions with the immune system, and cellular adhesion—between other cells, the extracellular matrix, and basement membranes. Below, we highlight nine families that we believe are most intriguing, but we have also provided a full accounting of each gene family in a table supplement—indicating the number and percentage of genes differentially expressed among them (S6 Data)

#### Laminins

Each gene family told a story. The family of laminins, for example, is composed of 12 genes that code for laminin a, b, and g chains. These chains aggregate to form an array of heterotrimeric laminin molecules with distinct properties that polymerize in the extracellular matrix and help shape basement membranes. Above, we found that myoepithelial cells highly expressed the chains composing laminin 332 (Fig 14). Inspection of the entire laminin family revealed that fibroblasts highly express 4 laminins (LAMA1, LAMA2, LAMB1, and LAMC3); endothelial cells express the highest levels of 3 (LAMA4, LAMB1, and LAMC1), whereas ER^Pos^ luminal, vascular endothelial, and pericytes significantly expressed LAMA5 (Fig 15B—laminin subunits). There were also several intriguing instances where closely related cell types expressed widely disparate levels of a particular laminin chain. Notable examples were the differential expression of LAMA1 between ER^Pos^ and ER^Neg^ luminal cells, LAMB1 between the 2 endothelial cell types, and LAMC3 between pericytes and vSMCs. Furthermore, no cell type meaningfully expressed LAMB4, consistent with its consideration as a pseudogene [33].

The relationship between cells and their basement membranes is dynamic and reciprocal. Each cell type contributes to the basal lamina’s structural diversity by secreting basement membrane components like the laminins and other extracellular molecules. These integral basement membrane proteins anchor the cells via membrane-tethered receptors and transmit signals to the cells, where they become integrated and influence multiple cellular processes.

#### Fibulins

One such related and intriguing ECM-related gene family was the fibulins. Fibulins are RGD-containing extracellular matrix glycoproteins that bind basement membrane proteins, such as tropoelastin, fibrillin, and fibronectin [34]. Whereas we found that fibroblasts highly expressed most fibulin family members, fibulin 5 (FBLN5) levels were highest in the ER^Neg^ luminal epithelial cells (Fig 15B—fibulins). FBLN5 was also notably expressed by lymphatic endothelial cells at substantially higher levels than their vascular counterparts. Fibulin 5 is one of the smallest fibulins, is secreted, has unique calcium and integrin binding properties, and is essential for elastic fiber formation [34]—and thus likely contributes to the elasticity of the lymphatic endothelium. We also found fibulin 3 (EFEMP1) was also differentially expressed by lymphatic endothelial cells, presumably providing similar contributions.

The fibulins and laminins are just 2 examples of the 187 gene families whose members were entirely differentially expressed. Of the remaining families (having fewer than 100% DE genes), we identified 211 containing between 75% and 99% DE genes (Fig 15A). Over half of these families were composed of 6 or more genes. These 126 families included integrin alpha subunits, toll-like receptors, kruppel like factors, adenylate cyclases, low-density lipoprotein receptors, glypicans, interleukin receptors, ephrins, and others (S6 Data). One of the larger and more familiar families was the collagens.

#### Collagens

The family of collagens consists of 45 individual genes, and, as expected, we found mesenchymal and myoepithelial cell types highly expressed most of them. There were some notable exceptions, however. For example, the vascular endothelial cells highly expressed COL15A1 and COL9A3, whereas COL22A1 was expressed uniquely by luminal epithelial cells and adipocytes (Fig 15C). Particularly enlightening were the cell types expressing the collagen IV genes.

Collagen IV is a sheet forming collagen and the principal constituent of basement membranes. It provides cellular integrin and non-integrin attachment sites through its triple-helical and non-collagenous domains [35]. There are 6 collagen IV isoforms, which assemble to form one of 3 triple-helical heterotrimers, α1α1α2, α3α4α5, or α5α5α6 [35,36]. We found the expression of collagen IV chains in the breast cells quite revealing, as the most robust levels occurred in cells associated with the basal lamina, but patterns of isoform expression differed by cell type. For example, pericytes and vSMCs, which are embedded within, and help maintain the vascular basement membrane, expressed the highest levels of COL4A1, COL4A2, and COL4A4 (Fig 15C and Fig N, panels A–C, in S1 Text). In contrast, myoepithelial cells, which contribute significantly to epithelial basement membranes, exhibited the highest levels of COL4A5 and COL4A6. From these results, we would predict that the dominant collagen IV heterotrimer in epithelial basement membranes to be α5α5α6—whereas our data suggests it would be α1α1α2 within the basal lamina of the vasculature.

#### Integrins

The family of integrins was highly differentially expressed across the breast cell types. Integrins connect the ECM to the cytoskeleton and act as receptors that integrate extracellular signals, relaying information to the cell’s interior. Each integrin heterodimer forms from one α and one β subunit, from families of 18 α and 8 β isoforms, generating the 24 observed αβ combinations. When we investigated the family of integrins in the breast cell types, we found they were all differentially expressed—except for ITGAD, which was essentially absent in every cell type (Fig 15B—integrin beta subunits and Fig N, panel D, in S1 Text). Due to their diversity and cell-surface expression, 16 integrins serve as “cluster of differentiation” (CD) antigens. Above, we introduced that a critical marker in our FACS was CD49f, which is integrin α6 (ITGA6)—and that myoepithelial and endothelial cell types highly expressed it (Fig 14B and Fig A in S1 Text). Integrin a6 binds laminins through heterodimers formed with either b1 or b4 subunits. The a6b1 and a6b4 integrins, along with a3b1 and a7b1, are the 4 main laminin-binding integrins. Thus, along with α6 integrin, the main laminin-binding integrin heterodimers use a3 and a7 integrins. Investigating these transcripts’ expression in breast cells, we found that myoepithelial cells and ER^Pos^ luminal epithelial cells expressed the highest levels of ITGA3. In contrast, pericytes and vSMCs expressed the highest levels of ITGA7, and fairly specifically, which is consistent with prior reports of this subunit’s expression in vascular smooth muscle cells [37] (Fig M, panels D and E, in S1 Text). These data suggest that myoepithelial and perivascular cells use distinct integrin heterodimers to bind laminins within their respective basement membranes.

Another CD antigen is ITGAM (CD11b), which is frequently used to identify myeloid cells. We indeed found that within the breast cell populations, ITGAM was highly expressed by leukocytes—although it was also strongly expressed by adipocytes and luminal epithelial cells (Fig M, panel D, in S1 Text). Moreover, leukocytes also highly expressed 6 other integrins: ITGAE, ITGAL, ITGAX, ITGA4, ITGB7, and ITGB2 (Fig M, panel D, in S1 Text). ITGB7 and ITGB2 were exclusively expressed by leukocytes, consistent with their recognized role in trafficking leukocytes to sites of injury and infection [38]. Our data reflect this cell type specificity, and transcript levels within the different breast cell types provide a detailed accounting of these and other expressed integrin isoforms, offering valuable insights into potential dimer combinations used by the different cell types.

Along with the collagens, alpha integrins, and 206 additional gene families, families containing between 75% and 99% DE genes included the *plakins* (88% genes DE), *spectrins* (86%), and *receptor tyrosine kinases* (80%). By comparison, we identified 209 families whose members were expressed relatively equally across all breast cell types (0% to 24%), such as the *exportins* (0%, Fig 15E). Furthermore, we also investigated families formed by a more moderate makeup of DE genes, i.e., between 25% to 74% (S6 Data). Among these 814 families were the *semaphorins* (70%), *proteoglycans* (71%), *mucins* (71%), *prostaglandin receptors* (67%), *nuclear hormone receptors* (66%), and *claudins* (64%, S6 Data). Also included were the *matrix metalloproteinases* (61% DE), which are a family of proteinases that degrade all extracellular matrix components—and thus help regulate the homeostatic balance of ECM molecules in the breast microenvironment (Fig 15D).

#### Matrix metalloproteinases (MMPs)

Of the 23 MMP isoforms encoded within the human genome, we identified 14 differentially expressed among the breast cell types. Fibroblasts highly expressed over half of these MMPs, which included gelatinase A (MMP2), stromelysin 1 and 2 (MMP3, MMP10), macrophage elastase (MMP12), membrane type 1, 3, and 4 (MMP14, MMP16, MMP17), and MMP19. Predominant substrates for these MMPs include fibronectin, laminins, type III, IV, and V collagens, elastin, fibrin, pro-MMP2, pro-gelatinase A, aggrecan, and cartilage oligomeric matrix protein [39]. In addition, we found leukocytes (and adipocytes) expressed gelatinase B (MMP9) at its highest levels, consistent with reports of it being absent in fibroblasts and playing an essential role in ECM proteolysis and leukocyte migration [40,41]. Arguably the most notable deviation from the near-universal fibroblast expression of MMPs was the differential expression of matrilysin (MMP7) by ER^Neg^ luminal epithelial cells, where we found levels to be 18-fold higher than in their ER^Pos^ counterparts—and 237-fold higher than in fibroblasts (Fig 15D).

#### Tight junctions

Essential to multicellular life are the mechanisms in which cells join to form the fabric of tissues and guide their shape and functions. Above, we introduced the family of integrins that serve as central receptors binding cells to the ECM. Different cell lineages have distinct requirements. For example, breast fibroblasts are embedded within a collagen-rich matrix, whereas perivascular cells, which are also mesenchymal in nature, encircle vessels, help generate the endothelial basement membrane, and collaborate with endothelial cells to form the vasculature. The endothelial cells, in turn, must create tight junctions to generate a patent and sealed network for transporting liquid blood—especially important for forming the blood–brain barrier of the central nervous system. Epithelial cells are also unique because their glandular role requires them to develop functional sheets while maintaining a semi-permeable barrier to the luminal space, where they may secrete and collect milk. The lumen is also essentially connected to the outside environment through the ductal network and nipple opening, so epithelial cells must also establish a barrier to protect against bacteria and other pathogens—working in concert with adjacent leukocytes that closely associate with the epithelium. Thus, the formation of tight junctions is essential to the gland.

**Fig 16:**
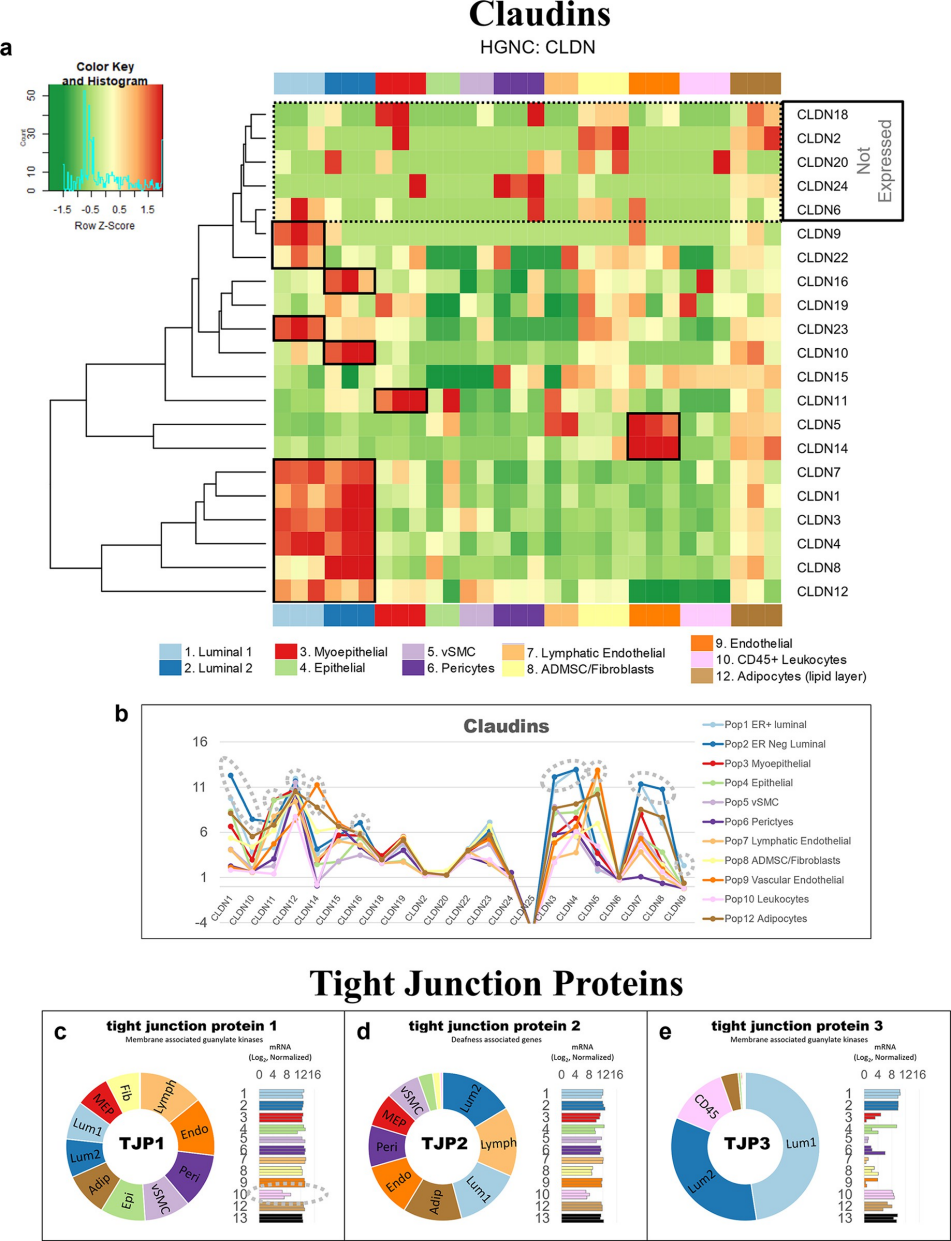
Tight junctions. **(a)** Heatmap displaying transcript levels of genes belonging to the claudin gene family (VST, normalized by row). Dashed box indicates claudins that were not appreciably expressed by any cell type. Dark boxes highlight genes differentially expressed by luminal epithelial cells, endothelial cells, and myoepithelial cell types. **(b)** Median rlog levels of the claudin family genes. Dashed circles identify genes outlined in the heat map. **(c–e)** Transcript levels for the zona occludens tight junction proteins: **(c)** TJP1, **(d)** TGP2, and **(e)** TJP3 (rlog values on log2 (bar graph) and linear (donut graph) scales). (The values for each graph can be found in Supplementary Data File 13 at 10.17605/OSF.IO/GFHC3).

Tight junctions are the prominent cell-to-cell junctions of epithelia and endothelia, establishing the selectively permeable barrier between neighboring cells [42]. Claudin and occludin proteins are vital components of this cell adhesion complex. Investigating their expression in the breast cells, we found the vast majority of claudins (11 of the 19 expressed) were predominantly limited to the 2 luminal epithelial cell types, although there were some notable exceptions (Fig 16A and 16B). One was the specific expression of claudins 5 and 14 (CLDN5, CLDN14) by endothelial cells—and to a lesser extent, by adipocytes. Whereas the vascular and lymphatic endothelial cells both highly expressed CLDN5, CLDN14-expression was limited to the vascular endothelial cells. Another exception was myoepithelial CLDN11 expression. Also, of the 11 claudins expressed by the luminal epithelial cells, 5 were differentially expressed between the 2 luminal types, with CLDN9, 22, and 23 transcripts being more highly expressed by the ER^Pos^ cells and CLDN16 and 10 being more highly expressed by the ER^Neg^ cells (Fig 16A and 16B). Furthermore, claudin 1 was one of the 6 claudins expressed by the luminal epithelial cells, and tissue staining confirmed luminal cell expression, as the staining was confined to the apical surface of the paracellular space, circumscribing the luminal surface (Fig O in S1 Text). These data are consistent with the epithelial and endothelial use of tight junctions, and results pinpoint the differential isoform specificity among the 5 claudin-expressing cell types.

The other critical components of tight junctions are the occludins (tight junction proteins encoded by TJP1, TJP2, and TJP3 genes). When we explored their expression among the breast cell types, we found that TJP1 and TJP2 (encoding zona occludens -1 and -2) shared a similar pattern, as they were both expressed relatively equally among all cell types. The only exceptions were that for TJP1, leukocyte levels were roughly 50-fold lower than the other cell types, and for TJP2, levels were lower in both fibroblasts and leukocytes. In contrast, TJP3 expression (ZO-3) was predominantly limited to the luminal epithelial cells (Fig 16C–16E).

#### Gap junctions

**Fig 17:**
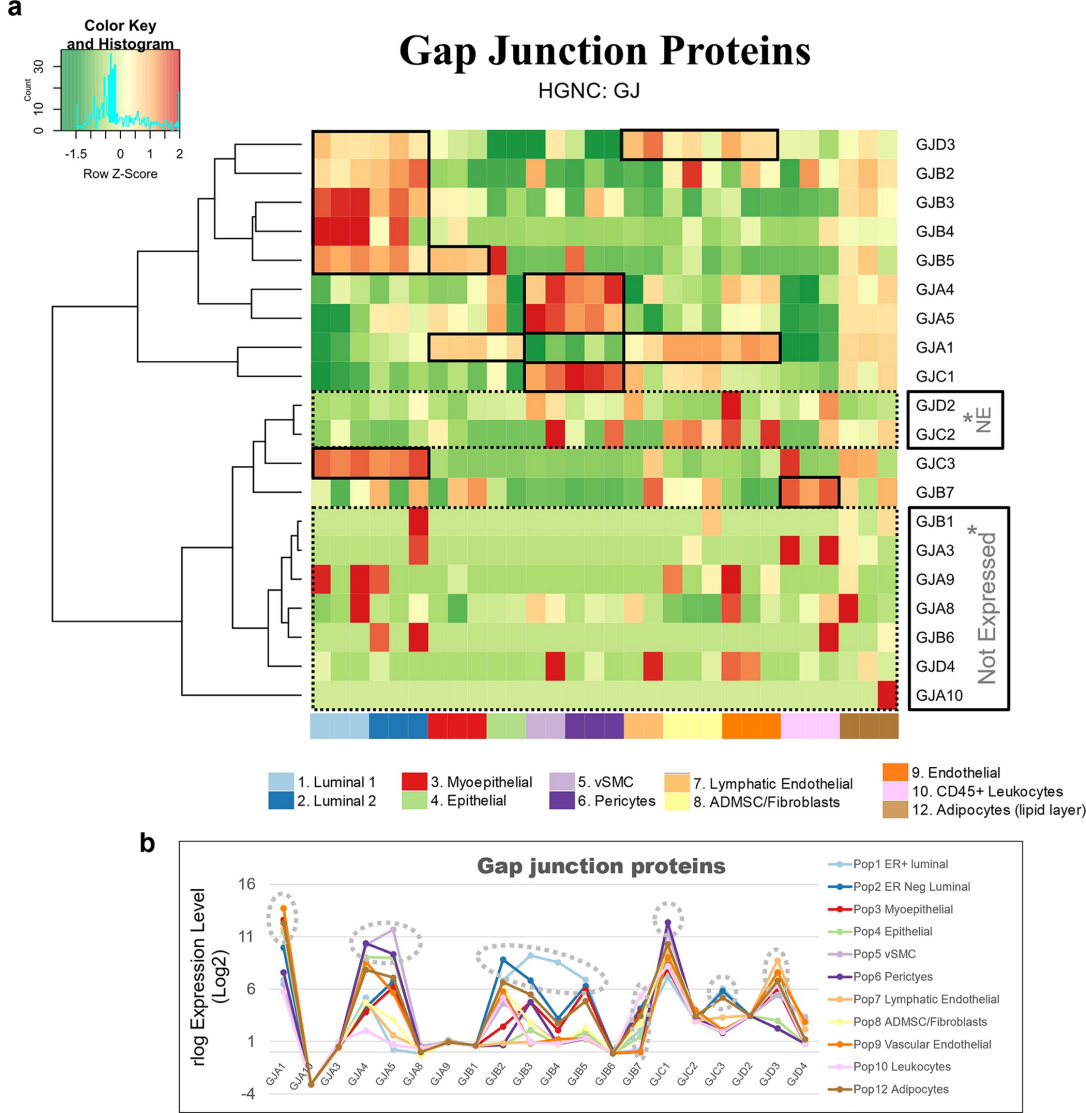
Gap junction proteins. **(a)** Heatmap displaying transcript levels of genes encoding gap junction proteins (VST, normalized by row). Boxed areas highlight the cell types expressing select gap junction proteins. Dashed boxes identify genes not appreciably expressed by any cell type, as evidenced by the rlog values shown in b. **(b)** Median rlog transcript levels of gap junction genes on a log2 scale; differentially expressed genes are encircled. (The values for each graph can be found in Supplementary Data File 13 at 10.17605/OSF.IO/GFHC3).

In contrast to the occluding tight junctions that seal the paracellular space between neighboring cells, gap junctions create channels between them, allowing the exchange of ions and low molecular weight metabolites between neighboring cells [43]. We found that the breast cell types expressed 11 of the 20 genes in the gap junction family (Fig 17A and 17B). Myoepithelial cells expressed two of these genes (GJA1 and GJB5, i.e., connexin 43 and 31.1), whereas luminal epithelial cells expressed 6 (GJB2, B3, B4, B5, C3, and D3), sharing only with myoepithelial cells the expression of GJB5. Furthermore, ER^Pos^ and ER^Neg^ luminal cells differentially expressed 3 gap junction genes, with GJB3 and GJB4 being higher in ER^Pos^ cells—and GJB2 being highest in the ER^Neg^ luminal cells. Another intriguing feature was the fibroblast and endothelial expression of GJA1, which they shared with Pop4 epithelial and myoepithelial cells. We also found that leukocytes uniquely expressed the highest levels of GJB7, although we are uncertain what role it would fulfill in these cells. One final observation was the perivascular expression of GJA4, GJA5, and GJC1; and the differential expression of GJA5 by vSMCs, at a 5.5-fold higher level than pericytes (Fig 17A and 17B). From these data, we can identify the potential homomeric and heteromeric gap junction complexes used by the perivascular and other cell types.

Whereas gap junctions facilitate the direct connection of cytoplasm between cell types and are essential mediators of cell-to-cell communication, other forms of communication occur through autocrine, paracrine, and endocrine signaling mechanisms. Multicellular organisms—and tissues—rely on efficient and responsive communication between different cells, sometimes separated by great distances. One of the most evolutionary-protected mechanisms facilitating cell communication is the production of proteins, peptides, fatty acids, glycans, cholesterol derivatives, gasses, and other metabolites by one cell, which are detected by another and elicit a response [44]. Unfortunately, the vast number of ligand and receptor families preclude us from exploring each of them here. Instead, we chose to inspect 2 different receptor families that exemplify the heterogeneous expression patterns observed among breast cell types: receptor tyrosine kinases and nuclear hormone receptors. The most studied and best-understood family of receptors possessing inherent kinase activity are the receptor tyrosine kinases (RTKs).

#### Receptor tyrosine kinases

**Fig 18:**
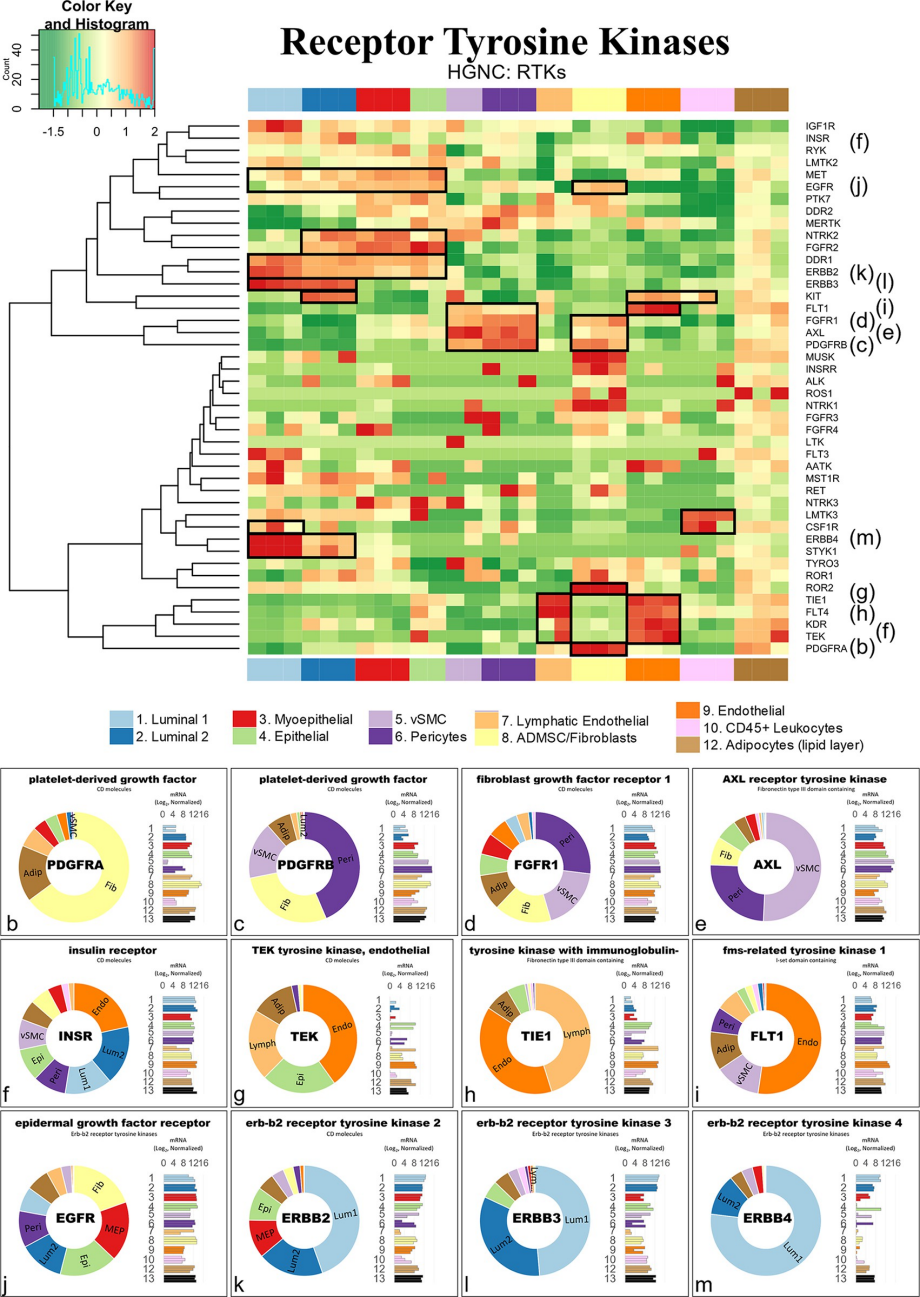
Receptor tyrosine kinases. **(a)** Heatmap displaying transcript levels of genes belonging to the tyrosine kinase receptor gene family (VST, normalize by row). Boxed areas indicate cell types expressing highest levels of select kinases. **(b–m)** Transcript levels of the select RTKs (rlog values on log2 (bar graph) and linear (donut graph) scales). (The values for each graph can be found in Supplementary Data File 13 at 10.17605/OSF.IO/GFHC3).

The human genome encodes 58 different RTKs whose ligands span across 20 subfamilies. These ligands are wide-ranging and include the ephrins, glial-cell derived neurotrophic factor, and: epidermal-, nerve-, hepatocyte-, fibroblast-, vascular endothelial-, and insulin-like growth factors, among others. RTKs are vital regulators of critical cellular processes and are frequently involved in many cancer types. Among the breast cell types, most RTKs displayed a cell type-specific expression pattern (Fig 18A–18M). Notably among these was the robust mesenchymal expression of platelet-derived growth factors alpha and beta (PDGFRA, PDGFRB), fibroblast growth factor receptor 1 (FGFR1), AXL tyrosine kinase receptor (AXL), and orphan receptor 2 (ROR2, Fig 18A–18M). In contrast, most cells equally expressed insulin receptor (INSR), insulin-like growth factor 1 receptor (IGF1R), receptor-like tyrosine kinase (RYK), and lemur tyrosine kinase 2 (LMTK2). As expected, the endothelial cells differentially expressed angiopoietin receptors tie2 and tie1 (TEK and TIE1) and VEGF receptors 1 and 3 (FLT1 and FLT4), which are known to regulate many facets of vascular biology (Fig 18A and 18I). We have previously described, consistent with prior reports [45–47], how stem cell growth factor receptor (KIT) was highly expressed by ER^Neg^ luminal cells[8], but we also found it to be appreciably expressed by vascular endothelial cells and vSMCs (Fig 18A and Fig K in S1 Text). Her2 (ERBB2) is amplified in a subset of breast cancers and has been successfully targeted for therapeutic intervention (Herceptin/trastuzumab). We found that Her2, along with two other members of the ErbB family (ERBB3 and ERBB4), were highly expressed by luminal epithelial cells. In contrast, the remaining family member, EGFR, was expressed across a broader range of cell types, with fibroblasts and myoepithelial cells displaying the highest transcript levels (Fig 18J–18M). Overall, RTKs display a complex pattern of expression. Most, 80% were expressed differentially across breast cell types, sometimes even within the same lineage, e.g., PDGFRB, KIT, ERBB3.

#### Nuclear hormone receptors

Nuclear hormone receptors were another receptor family demonstrating high cell type specificity. These receptors act as ligand-induced transcription factors and are activated upon binding to small lipophilic molecules, such as thyroid or steroid hormones, vitamin D3, retinoic acids (vitamin A), ecdysone, oxysterols, bile acids, fatty acids, leukotrienes, and prostaglandins [48]. The human genome has 49 nuclear hormone receptors, and their regulatory effects are wide ranging. Of this family’s many prominent members, the most notable for the breast field is estrogen receptor (ERα).

**Fig 19:**
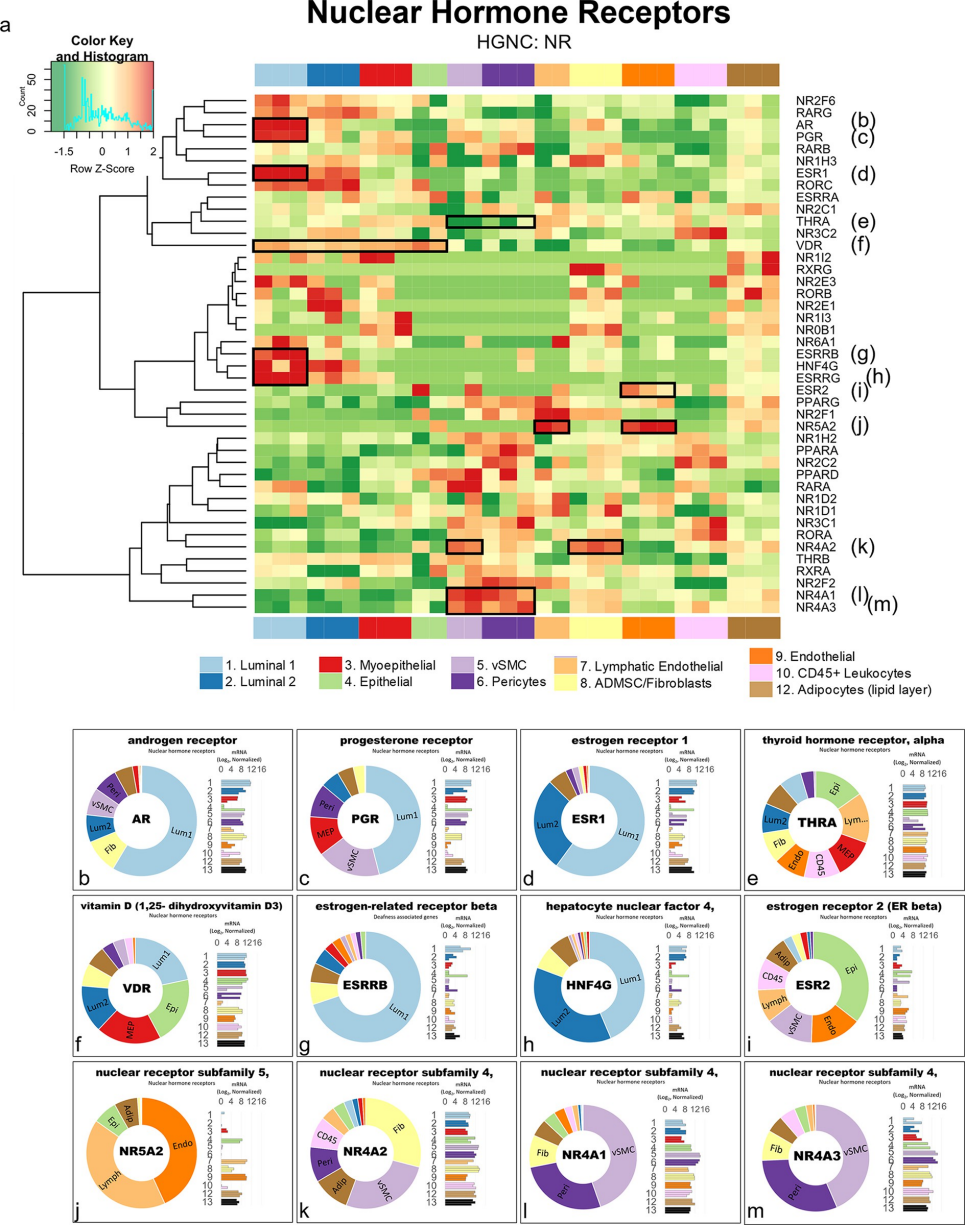
Transcript levels of nuclear hormone receptors. **(a)** Heatmap displaying transcript levels of genes belonging to the nuclear hormone receptor gene family (VST, normalized by row). Boxed areas indicate high/low gene expression in different cell types, which are highlighted below by the donut plots. Select genes include **(b–m)** mRNA values (rlog) are provided on log2 scale (bar graph of each biological replicate) and linear scale (donut graph of median value) which are both color-coded by cell type. (The values for each graph can be found in Supplementary Data File 13 at 10.17605/OSF.IO/GFHC3).

Interestingly, much of what we know about nuclear hormone receptors is rooted in the early studies of estrogen and how it regulates female reproductive organs, including the breast [49]. We found 44 nuclear hormone receptors expressed in breast cells—31 differentially. The androgen, progesterone, and estrogen α receptors were all strongly expressed by luminal epithelial cells—more so in ER^Pos^ cells than their ER^Neg^ counterparts (Fig 19A–19D). Both thyroid hormone receptors were similarly expressed among the cell types, except perivascular cells, which expressed 5.3-fold lower levels of THRA—and endothelial cells and leukocytes, which expressed 8.5-fold lower levels of THRB (Fig 19E). The cell type-specific expression extended to many other nuclear hormone receptors, such as vitamin D receptor (epithelial cell types), estrogen-related receptor beta (ER^Pos^ luminal cells), hepatocyte nuclear factor 4 gamma (both luminal cell types), estrogen receptor beta (Pop4 epithelial and vascular endothelial cells), and nuclear receptor subfamily members –5A2 (both endothelial cell types), –4A1(mesenchymal cells), –4A2 and –4A3 (perivascular cells, Fig 19F–19M). Nuclear hormone receptors possess broad influence over many cell processes. The cell-specific expression patterns detailed here identify the cell types in which these receptors likely play a prominent role.

Analyzing gene families has provided us with an unconventional look at how different types of genes contribute to the cell types in the breast, and the results identified the kinds of genes that vary most across the spectrum of cell types. Above, we meticulously described 9 gene families whose functions extend from the nucleus (hormone receptors) to the exterior cell microenvironment (laminins). However, these represent only a limited percentage of families we identified as having a high proportion of highly variable genes. Many more are listed in the supplementary tables (Table B in S1 Text and S6 Data). To further understand how these different genes collectively contribute to the individuality of each breast cell type, we needed to identify and evaluate the cellular pathways in which these genes are collectively involved—and the higher-order processes they bestow.

### Defining the nature of each breast cell type

To acquire a broad perspective of each breast cell type’s fundamental properties and individual functions, we performed a wholesale assessment of the biological pathways and processes disproportionately employed by these different cell types. Gene set enrichment analysis (GSEA) [50–52] accounted for each of the 20,000+ expressed genes in these cells’ transcriptomes. The algorithm determined whether a particular pathway’s genes were enriched within a bidirectionally ranked list of genes constructed from 2 contrasted cell types. One advantage of GSEA is that it does not require large transcriptional variations to pinpoint enriched pathways, as it can identify situations where many genes in a pathway differ in a small but coordinated fashion.

**Fig 20:**
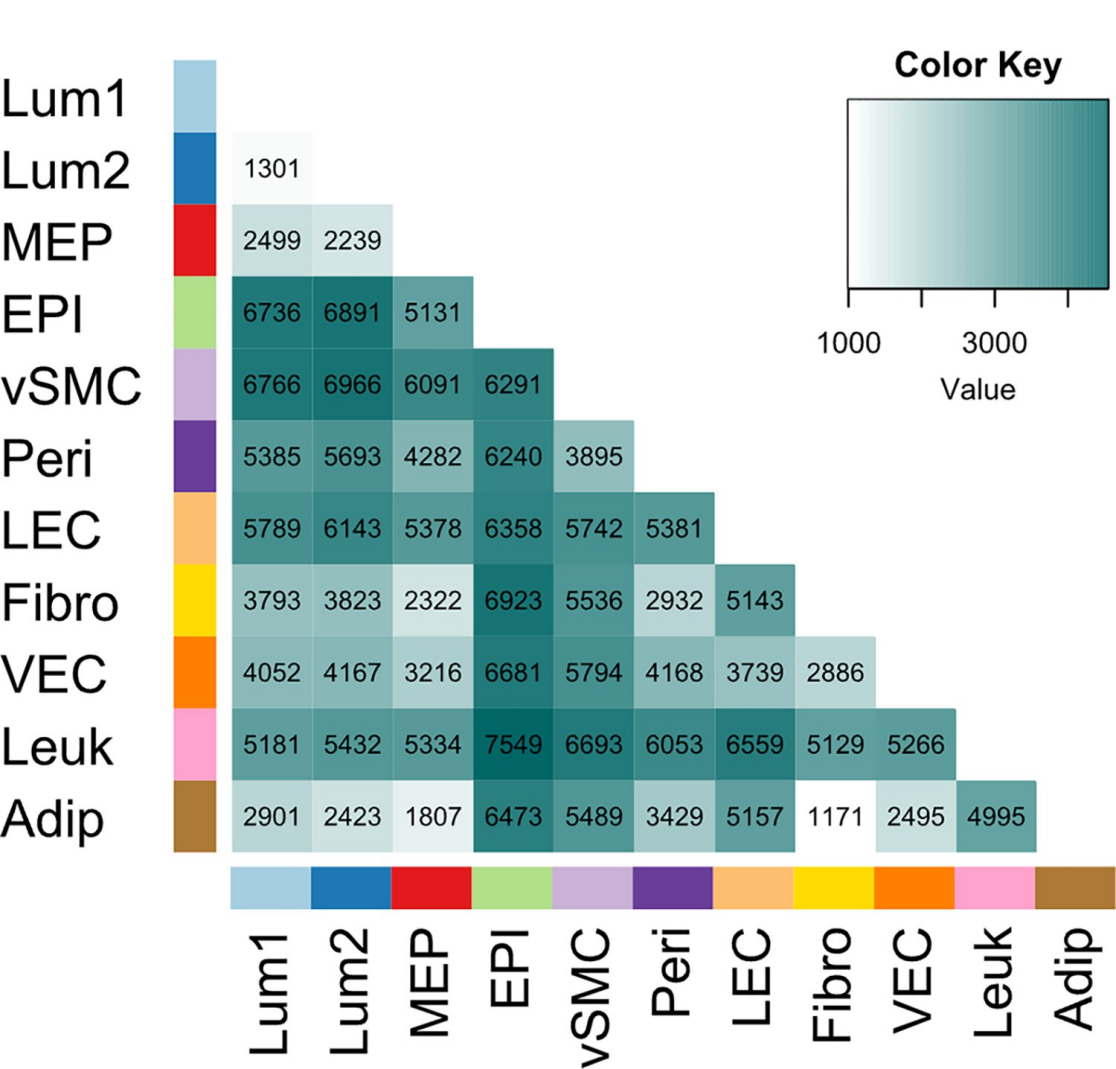
Differentially expressed genes across all cell type pairs. The number of differentially expressed genes between cell-type pairs, as reported by DESeq2; Benjamini–Hochberg adjusted *p*-values ≤0.1.

Several databases organize functionally related genes into distinct pathways. Here, we present our GSEA results that used gene sets defined by the Reactome Knowledgebase [17]. This database contains a manually curated list of 2,585 pathways cross-referenced to other scholarly resources, such as NCBI [53], ENSEMBL [54], KEGG [55], and GO [16,19]. Each Reactome pathway represents a collection of genes whose products coordinate to perform a specialized biological process. To determine which of these pathways are potentially active—or differentially enhanced—in a particular cell type relative to another, we analyzed every transcript, in each breast cell type, across every cell–cell comparison, which required 55 separate differential gene expression contrasts (Fig 20 and Supplemental Data File 7, 1v2 at 10.17605/OSF.IO/GFHC3 [56]). For each pairwise comparison, we calculated: (i) the log fold change for each measured transcript; (ii) the *p*-value for the calculated difference (Wald test); and (iii) the Benjamini–Hochberg adjusted *p*-value. Using these values, we ranked all expressed genes bidirectionally, placing the most statistically significant genes at the extreme ends of the list, with one cell type represented at the top (most positive)—and the other at the bottom (most negative). After ranking the genes, we performed GSEA, which extracted pathways from the Reactome database, identified the position of each pathway’s genes within our ranked list, and used a running sum algorithm to calculate a normalized enrichment score for each pathway. For each pairwise comparison, hundreds of enriched pathways were typically identified, each consisting of 15 to 200 genes.

To help us navigate the overwhelming amount of data generated from these combined analyses, we integrated our GSEA results and gene expression values into a single pathway analysis tool that we programmed within Microsoft Excel (Supplementary Data File 12 at 10.17605/OSF.IO/GFHC3 [9]). This facile tool efficiently summarizes results according to user inputs (e.g., cell types to contrast, FDR cutoffs to use, biological themes to investigate, specific pathways to examine, and genes to explore).

With the aid of the Pathway Analyzer tool, we found that the number of pathways enriched in each cell type varied widely, ranging between 7 to 462 (Supplementary Date File 12 at 10.17605/OSF.IO/GFHC3, $\bar{x}=194,sd=120$). On average, fibroblasts had the fewest enriched pathways across all comparisons ($\bar{x}=85\pm 59$), whereas those having the most were the vSMCs ($\bar{x}=315\pm 86$), pericytes ($\bar{x}=272\pm 121$), vascular endothelial cells ($\bar{x}=287\pm 126$), and lymphatic endothelial cells ($\bar{x}=229\pm 114$). When we compared cell types across different lineages, akin to the PCA analysis performed above, many of the same enriched pathways emerged, e.g., keratinization within the epithelial cell types, angiogenesis by endothelial cells, and more. Unfortunately, the magnitude and comprehensive nature of the GSEA results preclude us from presenting here every identified aspect. Instead, we will focus on the biology of closely related cell types since the broad features of different cell lineages were examined above. However, the results from all analyses are accessible, and one can thoroughly explore any of the 55 possible comparisons using the Pathway Analyzer tool (Supplementary Data File 12 at 10.17605/OSF.IO/GFHC3). Below, we present the most thought-provoking findings from 6 cell-type comparisons; these are: (i) the 2 luminal epithelial cell types; (ii) ER^Neg^ luminal epithelial cells and myoepithelial cells; (iii) ER^Neg^ luminal epithelial cells and vascular endothelial cells; (iv) the 2 endothelial cell types; (v) fibroblasts and adipocytes; and finally (vi) fibroblasts and pericytes. Some enriched pathways we anticipated, such as finding the enrichment of “estrogen signaling” in ER^Pos^ luminal cells. These results validated the analytic approach and helped us establish confidence in the pathway enrichments.

### Estrogen signaling in luminal epithelial cells: A validating example

**Fig 21:**
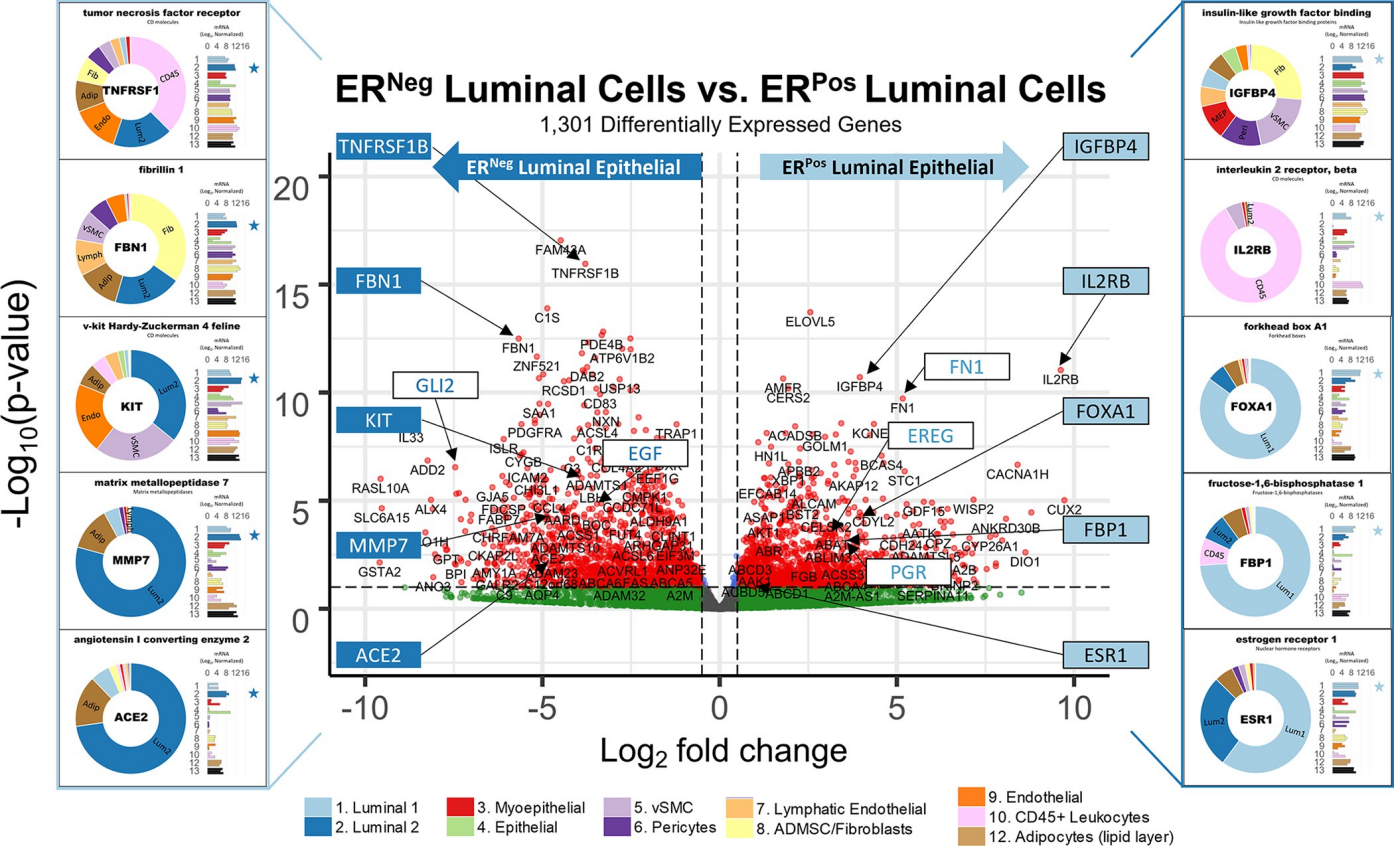
Differences between ER^Pos^ and ER^Neg^ luminal epithelial cell types. A contrast of expressed transcripts between the 2 luminal epithelial cell types revealed 1,301 differentially expressed genes (DeSeq2/Benjamini–Hochberg adjusted *p*-value ≤0.1; Supplemental Data File 7, 1v2 at 10.17605/OSF.IO/GFHC3). All expressed genes are displayed. The horizontal dashed lines (separating red and green dots) represents an Adj. *p*-value of 0.1. Established cell type markers (of each cell type) are denoted. mRNA expression values for 10 genes (indicated by filled, color-coordinated boxes) are shown at left and right of the volcano plot. Color-coded stars in the bar graphs indicate the cell type being contrasted that had the higher level of that gene; i.e., ER^Pos^(light blue) and ER^Neg^ luminal cells (dark blue).

Luminal epithelial cells help shape the breast’s glandular structure by forming the lumen of mammary ducts. The breast’s 2 major luminal cell types are commonly classified by their divergent staining for estrogen receptor alpha (ERα), and we were able to isolate each of these cell types based on their mutually exclusive staining for α6 integrin (ITGA6/CD49f; Fig 1 and Fig A in S1 Text). To determine the distinguishing characteristics of these 2 cell types, we contrasted their expressed transcripts, exposing 1,301 differentially expressed genes (BH/FDR adj. *p*-value < 0.1, Fig 21). ER^Pos^ luminal cells expressed higher levels of 546 of these, including progesterone receptor (PGR), epiregulin (EREG), fibronectin (FN1), and FOXA1. In contrast, transcripts differentially expressed by the ER^Neg^ cells included EGF, matrix metallopeptidase 7 (MMP7), KIT, and 752 other genes (Fig 21 and Supplementary Data File 7, 1v2 at 10.17605/OSF.IO/GFHC3 [56]). We ranked all expressed genes by their differential expression (based on their fold-change and statistical significance) and performed pathway enrichment analysis to identify the cellular functions impacted by these cell types’ transcriptional differences.

**Fig 22:**
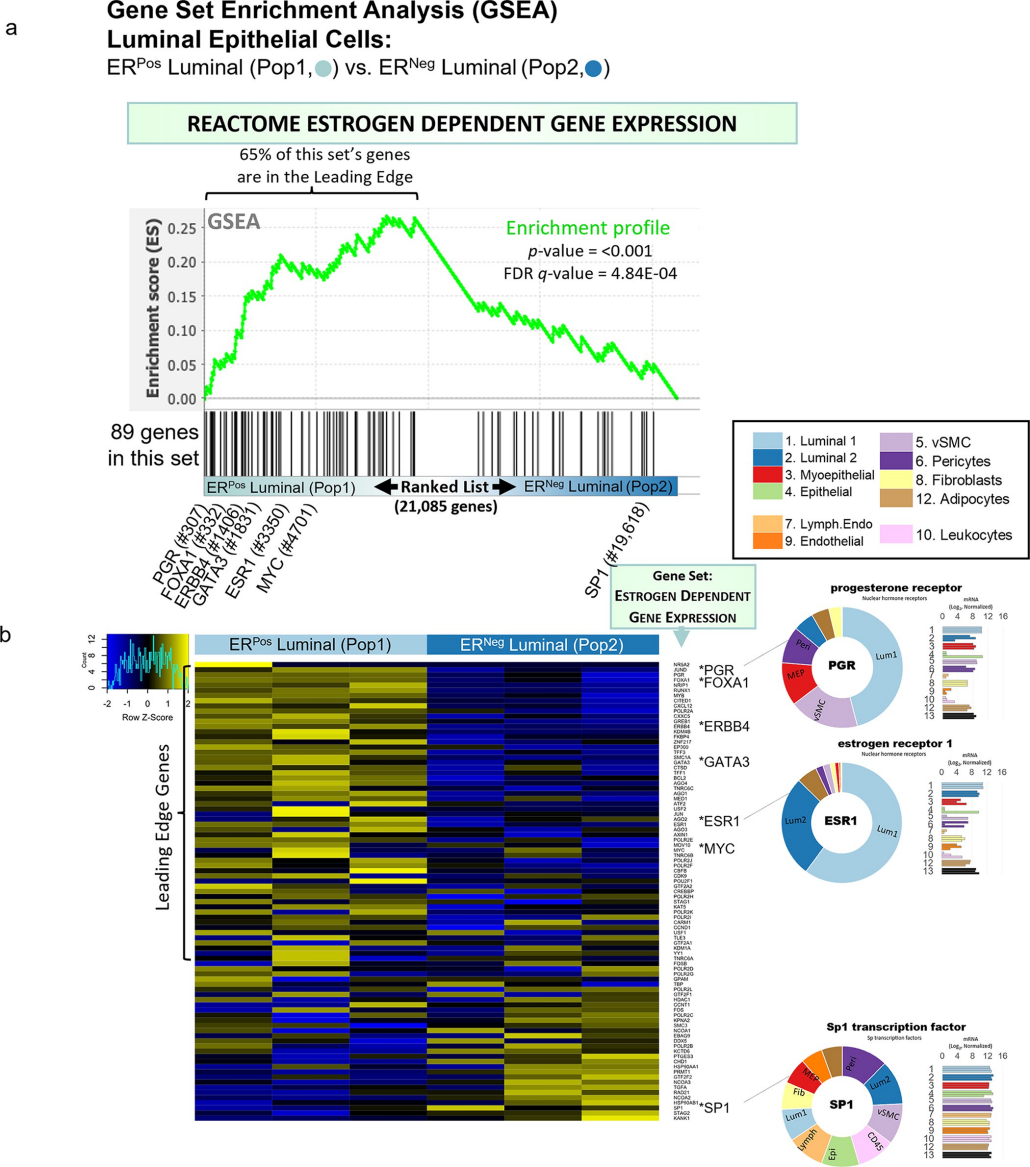
Inspection of GSEA results and the validating example of the estrogen-dependent gene expression pathway. **(a)** GSEA was performed using a list of genes expressed by ER^Pos^ and ER^Neg^ luminal epithelial cells, ranked by the statistical significance of their differential expression, *-log*_*10*_ {p-value} * sign([Fold Change]). The ranked list, depicted by the shaded bar, has the most statistically significant genes located at its extreme ends, with the ER^Pos^ cells’ most differentially expressed genes represented at the left end and the ER^Neg^ cells’ most differentially expressed genes on the right. GSEA identified the Reactome pathway estrogen-dependent gene expression as one of the most statistically significant enriched pathways identified (FDR <0.001; Supplementary Data File 12 at 10.17605/OSF.IO/GFHC3, select 1v2, 00_DS). The position of the 89 genes composing the estrogen-dependent gene expression pathway within the ranked list of expressed genes are depicted by vertical black lines. Names and positions of several familiar genes in the pathway are indicated, e.g., progesterone receptor (PGR) is found at position 337 (out of 21,085 expressed genes). The GSEA algorithm assessed the distribution of the pathway’s 89 genes within the ranked list using a running sum score (green trace), calculating statistics by comparing results to analysis of a randomized ranked list (1,000 permutations). **(b)** Heat map depicting transcript levels within ER^Pos^ and ER^Neg^ luminal epithelial cells of the 89 genes belonging to estrogen-dependent gene expression pathway, ordered by their relative position in the ranked list (VST values normalized by row; Supplementary Data File 12 at 10.17605/OSF.IO/GFHC3: select 1v2, Estrogen-Dependent Gene Expression). Positions of the familiar genes are indicated, including SP1 (which was near the bottom of the list and was not differentially expressed). Transcript levels of PGR, ESR1, and SP1 in each cell type (rlog values on log2 (bar graph) and linear (donut graph) scales). FDR, false discovery rate; GSEA, gene set enrichment analysis.

GSEA assessed where each pathway’s genes fell along our ranked gene list, and the algorithm calculated a normalized enrichment score, nominal *p*-value, and false discovery rate (FDR) for each Reactome pathway. The estrogen-dependent gene expression set was among the top pathways enriched in the ER^Pos^ luminal cells (*p*-value <0.001, *q-*value = 4.84 × 10^−4^). Identifying this estrogen signaling pathway was validating and served as a fine example of the enrichment algorithm (Supplementary Data File 12, 1v2 at 10.17605/OSF.IO/GFHC3). Differentially expressed genes contributing to this pathway’s enrichment included progesterone receptor (PGR), FOXA1, erb-b2 receptor tyrosine kinase 4 (ERBB4), GATA binding protein 3 (GATA3), estrogen receptor a (ESR1), MYC, and others (Fig 22A). These genes were among the 65% in the pathway that fell within the leading edge of the analysis (the leading-edge genes are the core genes that contributed most to the GSEA enrichment score, Fig 22A). Furthermore, when we analyzed transcripts from this estrogen-related pathway, we confirmed that the leading-edge genes were indeed differentially expressed between the ER^Pos^ and ER^Neg^ luminal epithelial cells (Fig 22B and Supplementary Data File 12 at 10.17605/OSF.IO/GFHC3).

**Fig 23:**
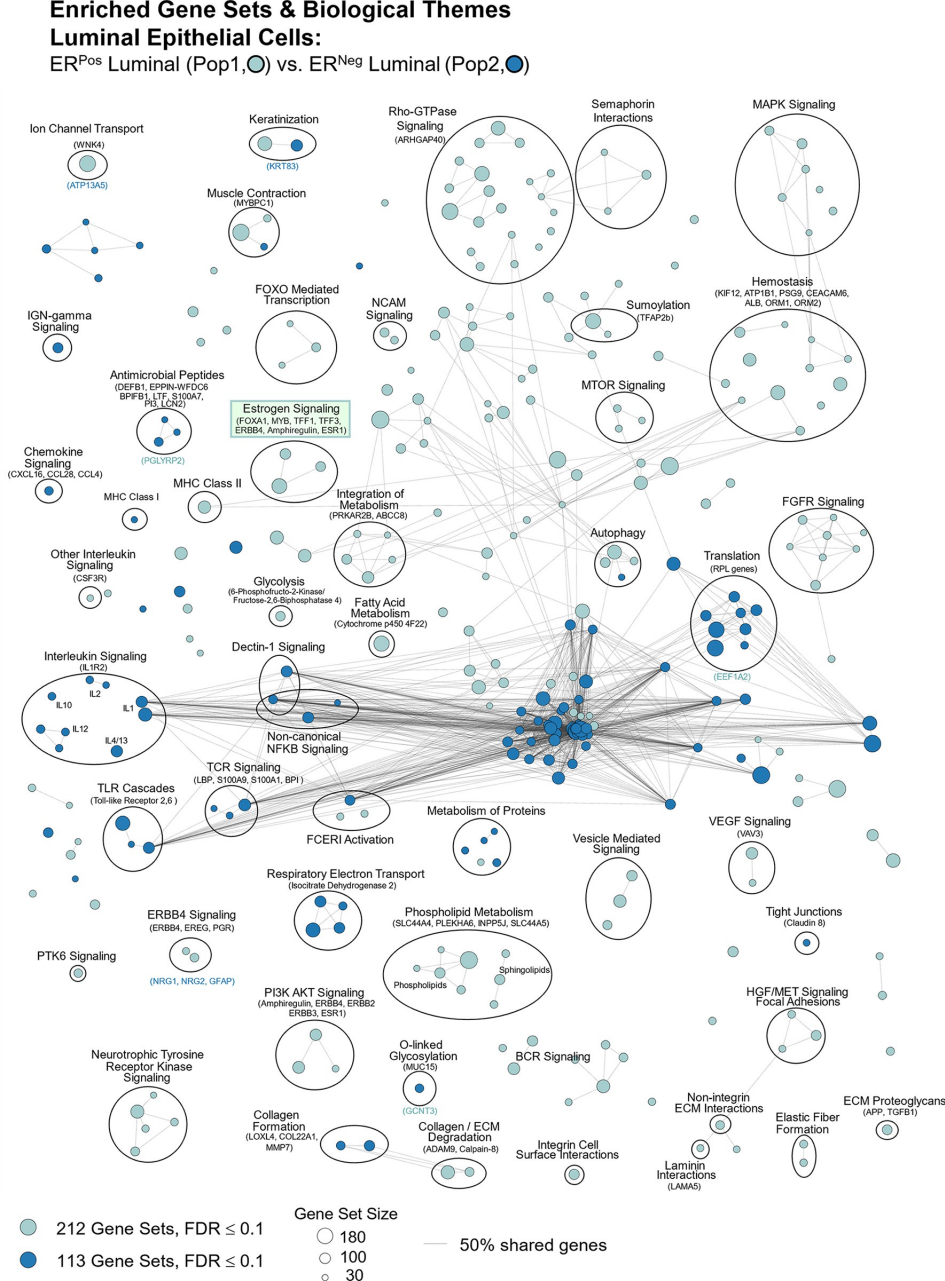
ER^Pos^ and ER^Neg^ luminal cells’ enriched pathways. Visualization of pathways enriched among ER^Pos^ and ER^Neg^ luminal cells (Enrichment Map/Cytoscape). All enriched pathways with an FDR ≤ 0.1 appear on the map (nodes, color coded by cell type). Pathways (nodes) are connected when they share 50% of their genes (or greater). Node size corresponds to the number of genes in the pathway, as indicated (bottom left). Biologically related pathways sharing genes formed clusters in the network (biological themes), and different themes with a common biological function (e.g., metabolism-, immune -, and ECM-related) were typically placed adjacent to each other (when possible). Select themes were encircled and annotated. Highly differentially expressed genes are indicated (above the theme when it coincided with the attributed cell type—and below if it was expressed by the contrasted cell type). The names of each pathway are available in the supplemental Cytoscape file (Supplementary Data File 8 at 10.17605/OSF.IO/GFHC3) and Pathway Analyzer tool (Supplementary Data File 12 at 10.17605/OSF.IO/GFHC3). FDR, false discovery rate.

Apart from estrogen-dependent gene expression, we identified an additional 324 enriched pathways between the 2 luminal cell types, 211 within the ER^Pos^ cells and 113 within the ER^Neg^ cells (FDR ≤ 10%, Supplementary Data File 12 at 10.17605/OSF.IO/GFHC3). We used a pathway enrichment and visualization method to extract information rooted within these GSEA results (Enrichment Map [57]). This tool analyzed the pathway’s gene sets and, based on the proportion of genes they shared, formed a complex network of interconnected pathways, placing redundant and biologically related pathways close to each other. We connected pathways when they shared 50% or more genes, and this caused distinct clusters, or “biological themes,” to emerge in the network (Fig 23 and Supplemental Data 8 at 10.17605/OSF.IO/GFHC3). We circled these grouped pathways and named their overarching theme using Reactome’s hierarchy nomenclature as a guide. For example, the estrogen-dependent gene expression gene set clustered with the 2 other sets related to estrogen signaling: estrogen-receptor mediated signaling and extra-nuclear estrogen signaling. We encircled all 3 on the map and labeled this overall theme “Estrogen Signaling” (Fig 23). We adhered to this strategy for each theme in the network and often arranged related themes together. For example, we placed the enriched “FOXO mediated transcription” theme adjacent to “Estrogen Signaling” on the enrichment map because FOXO3 is an established ERα regulator [58,59].

To evaluate the GSEA results and biological rationale for each pathway’s enrichment, we studied the genes from each pathway—and their expression levels in the different breast cell types. These details often provided valuable insights (Supplementary Data File 12 at 10.17605/OSF.IO/GFHC3). For example, high-scoring genes within the ER mediated signaling pathway included: HSBP1, which binds to ERα and promotes membrane localization; JUND, which interacts with ERα and occupies estrogen response element sites within the promoters of target genes; progesterone receptor, which is a direct downstream target of ERα; and others, like FOXA1, AKT1, epiregulin (EREG), amphiregulin (AREG), and SRC proto-oncogene. These genes were all highly expressed by ER^Pos^ luminal cells, and their transcript levels in these cells stood in stark contrast to those we observed within ER^Neg^ luminal cells—and the same was observed for FOXO mediated transcription (Fig P, panels A and B, in S1 Text). In some cases, a pathway’s genes were uniquely expressed (or nearly so) by one of the contrasted cell types. We indicated these genes on the enrichment map under each theme’s name. For example, the genes encoding estrogen receptor a (ESR1), amphiregulin growth factor (AREG), transcriptional factors FOXA1 and MYB; tyrosine kinase receptor ErbB4, and the trefoil factor mucosal proteins TFF1 and TFF3 were all highly and differentially expressed by ER^Pos^ luminal cells (Figs 22B and 23). Collectively, these data provided strong evidence that estrogen signaling is active in this cell type—as one would anticipate in cells expressing estrogen receptor.

Identifying the association between estrogen signaling pathways and ER^Pos^ cells was validating—as was later finding additional anticipated themes in other cell type contrasts. Moreover, these correlations assured us that the pathway analysis had indeed identified processes used disproportionately between cell types. Below, we move on from the detailed example of estrogen signaling in these luminal cells and sift through the complexity and sheer volume of remaining results.

### ER^Pos^ versus ER^Neg^ luminal epithelial cells (Pop1 versus Pop2)

When we compared transcripts expressed by ER^Pos^ and ER^Neg^ luminal cell types (FACS populations #1 and #2, Fig 1A), we identified 325 enriched pathways at a false discovery rate of 10%. The majority of these pathways were associated with cell signaling (83 pathways), metabolism (55 pathways), and immune-related functions (42 pathways). Others involved extracellular matrix organization, developmental biology, nervous system development, cell signaling, muscle contraction, and vesicle-mediated transport (Fig 23 and Supplementary Data File 12 at 10.17605/OSF.IO/GFHC3). Further exploration of expressed transcripts defined by each pathway showed that the GSEA algorithm functioned well at identifying significant, concordant differences in the gene levels between cell types—even if individual transcript levels only modestly diverged. There were, however, pathways that contained a significant share of highly differentially expressed genes—some of these being principal players in the pathway. For example, fibronectin (FN1) and TGFB1 are members of the ECM proteoglycans gene set and were expressed at over 35- and 10-fold higher levels in ER^Pos^ luminal cells than their ER^Neg^ cell counterparts (Adj. *p*-value = 3.2 × 10^−8^ and 3.8 × 10^−7^, Fig 23 and Supplementary Data File 7, 1v2 at 10.17605/OSF.IO/GFHC3 [56]). Because implications of these stark differences were often patently evident, we primarily focus on pathways that contain these, but details of all enriched pathways can be thoroughly examined with the provided analysis tool (Supplementary Data File 12 at 10.17605/OSF.IO/GFHC3).

#### Cell signaling and signal transduction (1v2)

The PI3K/Akt, ErbB4, VEGF, and HGF/MET pathways are all notable receptor tyrosine kinase pathways that we found enriched within ER^Pos^ cells (Fig 23 and Supplementary Data File 12 at 10.17605/OSF.IO/GFHC3). Along with p53, Phosphatidylinositol 3-kinase (PIK3CA) is the most mutated gene in breast tumors (36% of tumors), and genes in the phosphoinositide 3-kinase (PI3K/AKT) pathway are the most frequently altered in human cancers [60]. Across the span of normal breast cell types, we found PIK3CA to be evenly expressed (Fig P, panel C, in S1 Text). The PI3K/AKT pathway’s enrichment in ER^Pos^ luminal cells was instead primarily driven by the differential expression of the serine-threonine kinase AKT1, the EGFR ligand epiregulin (EREG), and the receptor tyrosine kinases: fibroblast growth factor receptor 1 (FGFR1) and PDGFRB. Other genes contributing to the pathway’s enrichment included the EGFR receptors ERRB2, ERBB3, and ERBB4 (Fig P, panel C, in S1 Text). These data show that the ER^Pos^ luminal cells contain different signaling elements than ER^Neg^ cells and possess the potential for stimulating themselves and neighboring cell types.

Another prominent member of the EGF family of receptors is ERBB4. This receptor dimerizes with ERα and is involved in many facets of downstream ER signaling [61]. Not surprisingly, we found ER^Pos^ luminal cells enriched with the ERBB4 pathways: signaling by ERBB4 and nuclear signaling by ERBB4 (FDR = 3.9% and 2.9%, Fig 23). This enrichment was driven by the cell’s prominent expression of ERBB4 and its ligands betacellulin (BTC) and epiregulin (EREG), although ER^Neg^ luminal cells differentially expressed several pathway members as well, including neuregulins 1 and 2 (NRG1, NRG2). In addition, glial fibrillary acidic protein (GFAP), another pathway member, was also expressed—nearly exclusively—by ER^Neg^ luminal cells. In CNS astrocytes (cells that express high levels of GFAP), GFAP promoter activity is inhibited when bound by an erbb4-containing complex [62]. This inhibition perhaps explains the reduced GFAP levels in ER^Pos^ cells, as these cells have high ERBB4 levels (Fig P, panel D, in S1 Text).

Another theme emerging from the analysis of the 2 luminal cell types was VEGF signaling, as we found the pathways signaling by VEGF and VEGFR2 mediated vascular permeability enriched within ER^Pos^ luminal cells (FDR = 0.17% and 4.2%, respectively). This result is, however, an excellent example of how comprehensive information on all the breast cell types provided substantial perspective. Between the 2 luminal cell types, the VEGF pathways were indeed enriched in the ER^Pos^ cells—predominantly due to their differential expression of the serine/threonine kinase AKT1, amyloid beta precursor like protein 2 (APLP2), neuropilin 1 (NRP1), and other pathway members. However, when we explored these transcript levels across all cell types, we found their expression in ER^Pos^ cells was not dramatically different from most other breast cell types (Fig P, panel E, in S1 Text). It thus became clear that the pathway’s enrichment was not a feature of its overexpression in ER^Pos^ cells but rather its relative repression in ER^Neg^ luminal epithelial cells (Supplementary Data File 12 at 10.17605/OSF.IO/GFHC3). Because of the importance of these comparative insights, we similarly evaluated gene expression across all cell types for each pathway, using heat maps and the Pathway Analyzer tool (Supplementary Data File 12 at 10.17605/OSF.IO/GFHC3).

Another pathway significantly enriched in ER^Pos^ luminal cells was HGF/MET. Hepatocyte growth factor (HGF) was first identified as a fibroblast-secreted factor that caused dramatic epithelial cell migration [63]. Its cognate receptor is MET receptor tyrosine kinase [64]. We found three HGF/MET signaling pathways enriched within ER^Pos^ luminal cells, including signaling by MET (FDR = 0.6%, Fig 23). Enrichment was mainly driven by the differential expression of fibronectin, integrin alpha 3 (ITGA3), and several laminin subunits (LAMA1, -A5, and -B2). These proteins are involved in focal adhesion signaling that acts through focal adhesion kinase (PTK2), which was also modestly elevated in ER^Pos^ cells (Fig P, panel F, in S1 Text). Interestingly, when we examined HGF expression across all cell types, we found, consistent with its initial discovery, that it was indeed highly expressed by breast fibroblasts, although pericytes and adipocytes also expressed it. HGF’s receptor, MET, was expressed by vascular endothelial cells, adipocytes, and the breast’s 4 epithelial cell types (Fig P, panel F, in S1 Text).

#### Metabolism (1v2)

To evaluate metabolism-related pathways, we coded a filter in the Pathway Analyzer tool, allowing us to extract pathways involved in specific biological themes (Supplementary Data File 12 at 10.17605/OSF.IO/GFHC3). At a 10% FDR threshold, we identified 55 enriched pathways related to metabolism—thirty within ER^Pos^ luminal cells and 25 within ER^Neg^ luminal cells. Those related to energy metabolism were enriched in ER^Pos^ luminal cells (Fig 23). Notable among these were: integration of energy metabolism (FDR = 0.550%), glycolysis (FDR = 7.94%), regulation of insulin secretion (FDR = 5.51%), PKA activation in glucagon signaling (FDR = 4.87%), and glucuronidation (FDR = 3.25%, Supplementary Data File 12 at 10.17605/OSF.IO/GFHC3). Genes contributing to these pathways’ enrichment included: the inositol 1,4,5-trisphosphate receptor (ITPR1), which is a PKA-activated calcium channel that acts downstream of glucagon and cAMP signaling; fatty acid synthase (FASN), which catalyzes the synthesis of palmitic acid from acetyl-CoA and malonyl-CoA; and cAMP-dependent protein kinase (PRKAR2B), which transduces cAMP signals (Fig P, panel G, in S1 Text). Elevated transcript levels of several glycolytic and regulatory enzymes within ER^Pos^ cells also led to the enrichment of the core glycolysis pathway. These were: hexokinase-1 (HK1), phosphofructokinase (PFKP), the bifunctional enzyme 6-phosphofructo-2-Kinase/Fructose-2,6-Biphosphatase 1 (PFKFB1), enolase gamma (ENO2), and pyruvate kinase M1/2 (PKM). These enzymes catalyze the first, last, and an intermediate step of glycolysis (HK1, PKM, ENO2)—and regulate other key regulatory metabolites in the pathway (PFKFB1; Fig 23 and Fig P, panel H, in S1 Text). Thus, significant evidence suggests ER^Pos^ cells have increased bioenergetic demands. As such, it was not surprising that we also found enrichment of pathways involved in lipid metabolism.

Metabolism of phospholipids, sphingolipids, and fatty acids were 3 themes that emerged from the comparison of the 2 luminal cell types (Fig 23). The ER^Pos^ cells’ disproportional expression of many of these pathways’ genes led to the enrichment sphingolipid metabolism (FDR = 0.04%), phosphoinositol metabolism (FDR = 0.06%), fatty acid metabolism (FDR = 9.53%), and related pathways (Supplementary Data File 12 at 10.17605/OSF.IO/GFHC3). Analysis of the leading-edge genes from these pathways demonstrated their elevated expression in the ER^Pos^ cells (Fig P, panels I–K, in S1 Text). The most notable genes involved in phospholipid metabolism included the solute carriers SLC44A4 and SLC44A5. These membrane-embedded choline transporters provide a critical metabolic precursor for phosphatidylcholine synthesis. ER^Pos^ cells also highly expressed pleckstrin homology domain protein (PLEKHA6) and phosphatidylinositol 4,5-bisphosphate 5-phosphatase A (INPP5J). PLEKHA6 interacts with phosphatidylinositol 3,4,5-trisphosphate (PIP3), whereas INPP5J dephosphorylates PIP3. Fatty acid elongase (ELOVL5) and fatty acid synthase (FASN) are both involved in synthesizing fatty acids, were highly ranked, and contributed to the pathways’ enrichment.

The set of pathways involving protein metabolism covers the range of reactions that proteins encounter over their lifespan, from synthesis to degradation (including protein folding, posttranslational modifications, and trafficking). We found both luminal cell types enriched with distinct protein metabolic pathways (Fig 23). In ER^Pos^ cells, two of the 11 significant pathways were N-glycan antennae elongation in the medial trans golgi (FDR = 0.79%) and sumoylation of intracellular receptors (FDR = 0.95%, Supplementary Data File 12 at 10.17605/OSF.IO/GFHC3). Differentially expressed genes in the former pathway included carbohydrate (N-acetylgalactosamine 4–0) sulfotransferases (CHST8, CHST10), ST8 alpha-N-acetyl-neuraminide alpha-2,8-sialyltransferases (ST8SIA2, ST8SIA6), and mannosyl (alpha-1,3-)-glycoprotein beta-1,4-N-acetylglucosaminyltransferase isoenzymes (MGAT4A, MGAT4B). These enzymes are active in the medial Golgi, where N-glycan remodeling occurs. When we explored the second pathway, sumoylation of intracellular receptors, we found that the small ubiquitin-like modifiers (SUMO1, 2, 3) were surprisingly expressed evenly across cell types. The pathway’s enrichment was instead driven by elevated expression of the retinoic acid, retinoid X, progesterone, androgen, and estrogen receptors, indicating the receptors that could be modified by sumoylation (Supplementary Data File 12 at 10.17605/OSF.IO/GFHC3).

The protein metabolism pathways enriched in ER^Neg^ cells included amyloid fiber formation (FDR = 5.95%) and O-linked glycosylation of mucins (FDR = 9.28%, Supplementary Data File 12 at 10.17605/OSF.IO/GFHC3). We also observed that many, but not all, large ribosomal protein components were elevated in ER^Neg^ cells, leading to the enrichment of ribosome-involved pathways, eukaryotic translation initiation (FDR ≤ 0.001%) and eukaryotic translation elongation (FDR ≤ 0.001%). Historically, these genes have been considered invariant, but more recent evidence suggests otherwise, that levels may be linked to cell specification [65]—as we observed among the breast cell types here.

#### Extracellular matrix organization (1v2)

The ECM is a dynamic structure consisting of a heterogeneous composition of scaffolding proteins, growth factors, proteoglycans, and remodeling enzymes. It undergoes persistent remodeling, primarily through reciprocal interactions with nearby cells in the immediate microenvironment. We found 11 pathways enriched between the 2 luminal cell types, with ER^NEG^ cells overexpressing genes related to collagen synthesis and assembly (FDR = 1.58%), whereas the ER^Pos^ cells were enriched in degradative pathways degradation of the extracellular matrix and collagen degradation (FDR 4.08% and 5.55%, Fig 23 and Supplementary Data File 12 at 10.17605/OSF.IO/GFHC3). Driving enrichment of the synthetic pathway in ER^Neg^ cells were collagens (COL4A2, 6A2, 6A1, 22A1), lysyl oxidases (LOX, LOXL4), and matrix metallopeptidases (MMP3, MMP7). In contrast, the degradative pathway in ER^Pos^ cells was driven by differential expression of tissue inhibitor of metalloproteinase 2 (TIMP2), calpains (CAPN8, CAPN2, CAPN13), ADAM metallopeptidase 10 (ADAM10), cathepsin L (CTSL), elastin (ELN), matrix metalloproteinases (MMP12, MMP13), and collagens (COL7A1, 12A1, 4A5, 4A6). These data suggest that these 2 related—but distinct—luminal cell types have contrasting roles in the synthesis and degradation of specific ECM proteins (Fig P, panels L and M, in S1 Text).

Additional enriched pathways in ER^Pos^ cell relating to the ECM included: laminin interactions (FDR = 4.1%), non-integrin ECM interactions (FDR = 0.18%), integrin cell-surface interactions (FDR = 0.47%), syndecan interactions (FDR = 6.47%), and ECM proteoglycans (FDR = 0.31%; Fig P, panel N–R, in S1 Text). Differentially expressed genes within these pathways included collagen IV isoforms A5 and A6 (COL4A5, COL4A6), laminin chains (LAMA5, LAMB2), fibronectin (FN1), transforming growth factor-beta -1 and -2 (TGFB1, TGFB2), platelet-derived growth factor beta (PDGFB), agrin (AGRN), synedecan 3 (SDC3), and integrins α5 and β3 (ITGA5, ITGB3), all of which had higher transcript levels in ER^Pos^ luminal cells compared to their ER^Neg^ counterparts.

#### Tight junctions (1v2)

Claudins are a family of genes we explored in the previous section, where we found, with few exceptions, that their transcription was mainly limited to endothelial and epithelial cells—predominantly the 2 luminal cell types (Fig 16A and 16B). Here, using GSEA, we identified significant differences between the ER^Pos^ and ER^Neg^ luminal cells (FDR = 9.1%). Specifically, the ER^Neg^ cells expressed significantly higher levels of CLDN10, CLDN16, CLDN11, CLDN8, and CLDN1, which led to enrichment of the tight junction interactions pathway (Figs 23 and 16A). This was notably the only Reactome “Cell-cell Communication” pathway enriched in either cell type (other pathways, for example, involve adherens junctions, cell-ECM interactions, and hemidesmosome assembly, etc.). Despite the elevated levels of 5 claudins in ER^Neg^ cells, CLDNs 1, 7, 3, and 12 were expressed at roughly the same level in both cell types, whereas ER^Pos^ cells had higher levels of CLDNs 23, 6, and 9. Thus, the enrichment indicated that the 2 luminal cell types possess unique tight junction components to support their individual physiologies. These tight junctions are critical to the cells’ ability to form semi-permeable barriers, which helps retain fluid and protect against microorganisms in the outside environment—to which the ductal system is connected via the nipple opening.

#### Immune system (1v2)

Using GSEA, we identified 42 enriched pathways related to immune function. The vast majority of these were in the ER^Neg^ luminal cells (33 pathways), which overlapped with the 2 innate immune system pathways previously identified by the PCA/overrepresentation analysis (Fig 10A). One of the larger themes was interleukin signaling, where we found the enrichment of 9 pathways related to: interleukin-4 and interleukin-13 signaling, interleukin-10 signaling, interleukin-1 family signaling, interleukin-2 family signaling, and interleukin-12 family signaling (FDR = 0.181%, 0.792%, 2.33%, 4.79%, 1.096%, respectively; Fig 23 and Supplementary Data File 12 at 10.17605/OSF.IO/GFHC3). Enrichment of these pathways was driven by the ER^Neg^ cell’s differential expression of the interleukins 33, 1a, and 18 (IL33, IL1A, IL18), chemokine ligands 4 and 5 (CCL4, CCL5), tumor necrosis factor receptor TNFRSF1B, serum amyloid-A1 (SAA1), interleukin-2 receptor gamma (IL2RG), interleukin-1 receptor type II (IL1R2), interleukin-27 receptor alpha (IL27RA), colony-stimulated factor 2RB (CSF2RB), and lipocalin 2 (LCN2, Fig P, panel S and T and Fig Q, panels A–C, in S1 Text). We notably found breast macrophages concentrated near the epithelial structures and within the ducts and epithelial compartment [8], and thus were not surprised to find luminal cell enrichment of interleukin pathways that involve macrophage activation. This indicates that the ER^Neg^ cells are disproportionately responsible for regulating macrophage physiology—a crosstalk that is subverted in breast tumors [66].

Among the other enriched immune-related pathways, we discovered ER^Neg^ luminal cells expressed higher levels of genes involved in toll-like receptor cascades and antimicrobial peptides (FDR = 8.208%, 4.1067%, respectively). The ER^Neg^ cells differentially expressed toll-like receptors 1 and 2 (TLR1 and TLR2), defensins B4A and B1 (DEFB4A and DEFB1), peptidase inhibitor 3 (PI3), and clusterin (CLU, Fig Q, panels D and E, in S1 Text). Furthermore, all 10 toll-like receptors were highly expressed by luminal cells, with half being the highest in these cells compared to all other cell types (TLR1, TLR2, TLR2, TLR5, and TLR6, Fig Q, panel D and Fig R, panels A–F, in S1 Text). Toll-like receptors play a vital role in the innate immune system by triggering an immune response to microbial agents. For example, TLR1 and TLR2 coordinate to mediate the response to bacterial lipoproteins. Although the toll-like receptors are well studied in the colonic epithelium, their contributions to the homeostasis of the breast epithelium are not as well characterized. Because luminal epithelial cells have the potential for elevated microorganism exposure, it is reasonable to expect TLR and antimicrobial peptide expression, as they are needed to provide innate immune protection.

#### Milk proteins (1v2)

Although not part of the Reactome gene sets, we noticed several genes encoding prominent milk proteins were highly differentially expressed by ER^Neg^ luminal cells, such as lactotransferrin (LTF) and casein kappa (CSN3). Lactotransferrin is the major iron-binding protein in milk, whereas casein kappa is critical for stabilizing casein micelle formation in milk. To explore the cellular expression of other milk constituents, we created a list of genes from prior reports of human milk composition [67–69]. This list comprised 48 genes and included lactotransferrin, caseins, lactalbumin, and other milk proteins (Fig S, panel A, in S1 Text). Most of these were expressed by at least 1 breast cell type (41/48), and nearly all were differentially expressed (37/41; BH/FDR adj. *p*-value <0.05), with one of the 2 luminal cells most often being the cell type with the highest transcript level (25/37). After comparing expression between the luminal cell types, we found 12 genes whose expression significantly differed between the 2 (adj.*p-*val <0.05), with ER^Neg^ cells most often having the higher transcript levels (8/12). These 8 genes were: lactotransferrin (LTF), secreted phosphoprotein 1 (SPP1), polymeric immunoglobulin receptor (PIGR), xanthine dehydrogenase (XDH), clusterin (CLU), complement component 3 (C3), haptoglobin (HP), and milk fat globule-EGF factor 8 protein (MFGE8, Fig S, panels B–J, in S1 Text). These results demonstrate that the luminal cells are primed for milk production despite being derived from the resting breast tissues of non-lactating women and, at rest, transcribe the genes encoding major milk proteins—with the highest levels often being in the ER^Neg^ luminal cells.

### ER^Neg^ luminal epithelial versus myoepithelial cells (Pop2 versus Pop3)

Whereas the luminal cells shape the lumen of the gland’s ductal structure, myoepithelial cells form the basal layer, anchoring the epithelium to the basal lamina (Fig 9A, 9D, 9E, 9G and 9H). The ER^Neg^ luminal and myoepithelial cells (FACS populations #2 and #3) are two of the most abundant cell types in the breast (Fig 1); they share the same epithelial lineage and spatial proximity—and physically interact. Most clinical breast tumors have a luminal phenotype and presumably originate from luminal cells, with the ER^Neg^ luminal cells being the most-likely cell of origin for basal-like breast tumors [47,70], despite these tumors’ basal-like features. For these reasons, we were interested in—and explored—the differences between ER^Neg^ luminal and myoepithelial cells, focusing on the biochemical and physiological processes distinguishing these 2 cell types.

**Fig 24:**
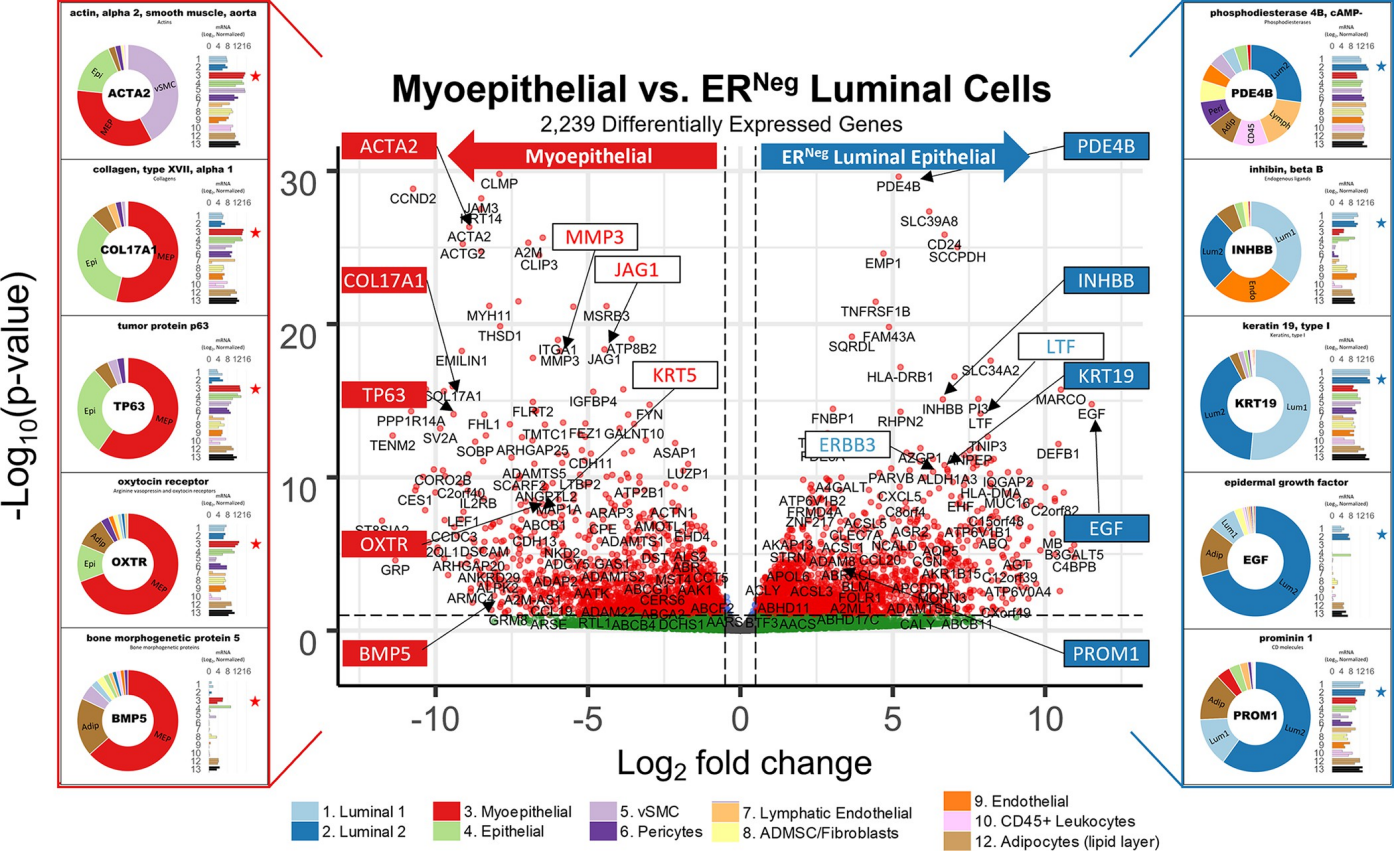
Differences between ER^Neg^ luminal and myoepithelial cell types. A contrast of expressed transcripts between ER^Neg^ luminal and myoepithelial cells revealed 2,239 differentially expressed genes (DeSeq2/Benjamini–Hochberg adjusted *p*-value ≤0.1; Supplementary Data File 7, 2v9 at 10.17605/OSF.IO/GFHC3). All expressed genes are displayed. The horizontal dashed lines (separating red and green dots) represents an Adj. *p*-value of 0.1. Established cell type markers (of each cell type) are denoted. mRNA expression values for 10 genes (indicated by filled, color-coordinated boxes) are shown at left and right of the volcano plot. Color-coded stars in the bar graphs indicate the cell type being contrasted that had the higher transcript level, i.e., ER^Neg^(blue) and myoepithelial cells (red).

**Fig 25:**
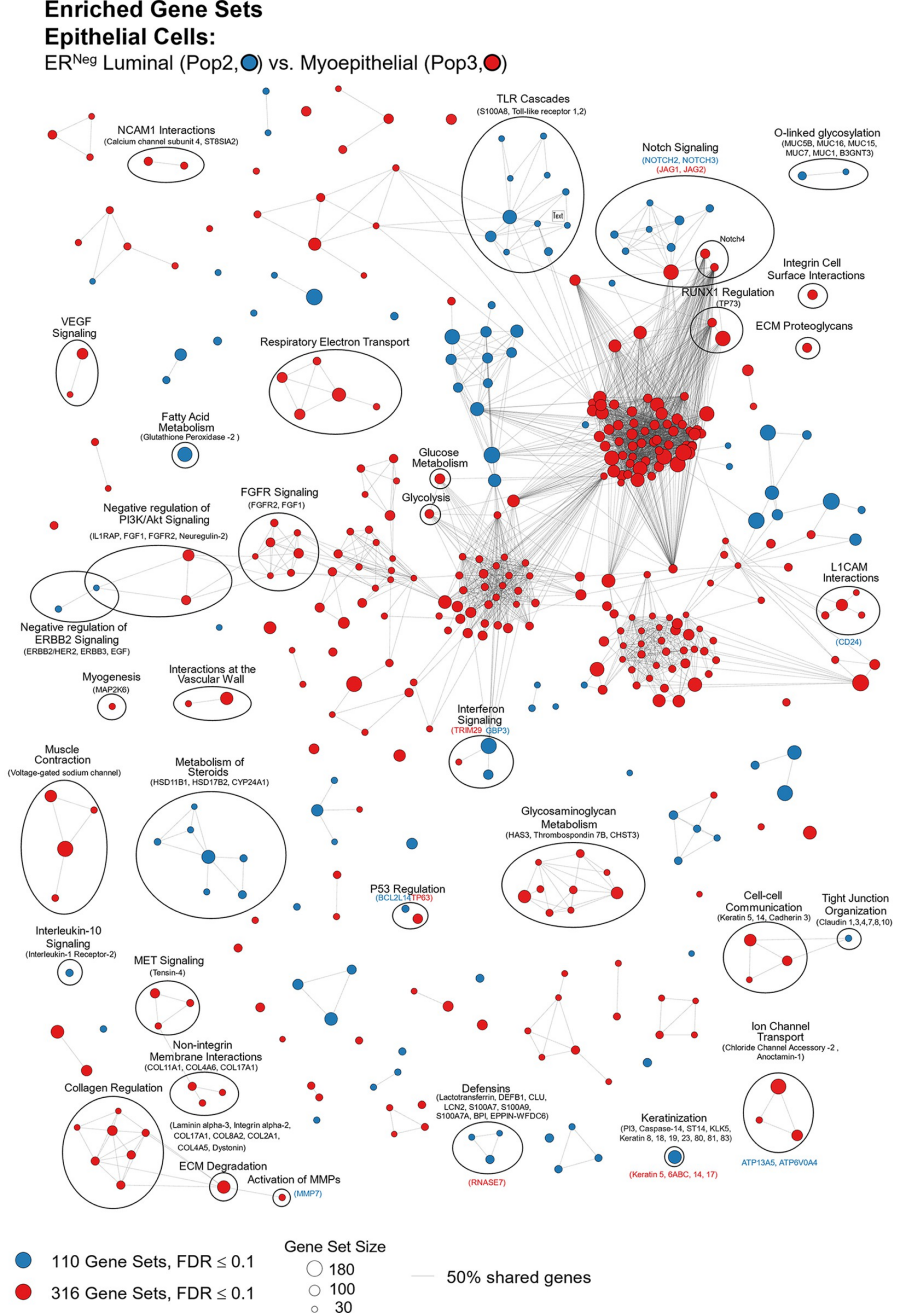
The ER^Neg^ luminal and myoepithelial cells’ enriched pathways. Visualization of pathways enriched among ER^Pos^ and myoepithelial cell types (Enrichment Map/Cytoscape). All enriched pathways with a FDR ≤ 0.1 appear on the map (nodes, color coded by cell type). Pathways (nodes) are connected when they share 50% of their genes (or greater). Node size corresponds to the number of genes in the pathway, as indicated (bottom left). Select biological themes were encircled and annotated. Highly differentially expressed genes are indicated (above the theme when it coincided with the attributed cell type—and below if it was expressed by the contrasted cell type). The names of each pathway are available in the supplemental Cytoscape file and Pathway Analyzer tool (Supplementary Data Files 9 and 12 at 10.17605/OSF.IO/GFHC3). FDR, false discovery rate.

Analysis of transcripts expressed by the myoepithelial and ER^Neg^ luminal cells revealed 2,239 differentially expressed genes (BH/FDR adj. *p*-value <0.1, Fig 24 and Supplementary Data File 7, 2v3 at 10.17605/OSF.IO/GFHC3 [56]). ER^Neg^ luminal cells expressed the higher level of roughly half of these genes (51.6%), which included CD24, keratin 19, prominin 1 (PROM1), EGF, and peptidase inhibitor 3 (PI3). Myoepithelial cells, in contrast, differentially expressed 1,083 genes; among these were genes previously associated with this cell type, including smooth muscle actin (ACTA2), keratin 5 (KRT5), p63 (TP63), and oxytocin receptor (OXTR, Fig 24). Using the *p-*values from this differential expression analysis, we created a ranked list of all expressed genes and used this list to perform pathway enrichment analysis. GSEA revealed enrichment of 426 Reactome pathways (FDR ≤ 10%), which clustered into distinct biological themes (Fig 25 and Supplementary Data File 9 at 10.17605/OSF.IO/GFHC3). The most significant of these involved cell–cell communication, extracellular matrix organization, and metabolism (Supplementary Data File 12 at 10.17605/OSF.IO/GFHC3).

#### Cell–cell communication (2v3)

Proper intercellular communication is essential for maintaining homeostatic balance and normal tissue function. When we contrasted the ER^Neg^ luminal and myoepithelial cells, we found enrichment of 4 cell–cell communication pathways: 1 in the ER^Neg^ luminal cells (tight junctions) and 3 in the myoepithelial cells (cell-junctions, cell communication, and ECM interactions). Given the previous identification of claudin expression in the luminal epithelial cells, it was not surprising to find the tight junction interactions pathway enriched in the ER^Neg^ luminal cells, as they—in comparison to myoepithelial cells—differentially expressed most epithelial claudins, such as CLDN1, 3, 4, 7, 8, 10, 12, and 16 (FDR = 5.57%). The only significant exception was the myoepithelial expression of CLDN 11 (Fig 16A and 16B). These results show that luminal cells express significantly more claudins than myoepithelial cells and thus rely on the tight junction features that these different claudins provide.

Although luminal cells differentially expressed claudins and were enriched with the tight junction interactions pathway, we found other cell communication pathways enriched in myoepithelial cells. These were the cell–cell communication pathway (FDR = 0.0919%) and its underlying pathways: cell junction organization (FDR = 0.0076%) and cell-extracellular matrix interactions (FDR = 0.1682%). Notable genes shared among these 3 pathways include actinin and filamin proteins that promote the branching of actin filaments and anchor the filaments to proteins in the cell’s membrane. Shared in the enriched pathways were also the cell adhesion molecule 3 (CADM3), keratins 5 and 14 (KRT5, KRT14), and cadherins 3, 4, and 11 (CDH3, CDH4, CDH11, Fig T, panel A, in S1 Text). As we found above with the PCA-derived gene list for myoepithelial cells (myoepithelial and Pop4 epithelial cells), a general feature of myoepithelial cells is their expression of ECM and basement membrane components and formation of desmosomes involved in cell junction organization (Fig 14A–14C). Thus, we were not surprised to find myoepithelial enrichment of the cell-extracellular matrix interactions pathway because these cells form and help shape the epithelial basement membrane. For the same reason, we anticipated other pathways involved in extracellular matrix organization would be enriched within myoepithelial cells. We found this was indeed the case.

#### ECM organization (2v3)

As the non-cellular component of tissues, the extracellular matrix is formed by a network of proteins, proteoglycans, growth factors, and minerals that provides structural support, elasticity, and tensile and compressive strength to the tissue [71]. The ECM and basement membrane composition is dynamic and is constantly remodeled by cells in their immediate vicinity. Notably, we found pathways associated with extracellular matrix organization as some of the most significantly enriched pathways in myoepithelial cells, as 9 of the 10 most statistically significant pathways in the myoepithelial cells involved ECM and basement membrane organization (Supplementary Data File 12 at 10.17605/OSF.IO/GFHC3). Myoepithelial cells were enriched in nearly every aspect of ECM organization, including Elastic fiber formation (FDR = 0.01%), Laminin interactions (FDR < 0.001%), non-integrin membrane ECM interactions (FDR < 0.001%), ECM Proteoglycans (FDR < 0.001%); activation of matrix metalloproteinases (FDR = 4.38%), collagen formation and collagen degradation pathways (FDR < 0.001%). These cells differentially expressed many collagen and laminin encoding genes, including COL2A1, 4A5, 4A6, 8A2, 11A1, and 17A1; and laminin chains LAMA1, LAMA3, and LAMB1). Other genes contributing to these pathways’ enrichment included elastin microfibril interfacer 1 (EMILIN1), metalloproteinase 3 (MMP3), tissue inhibitor of metalloproteinases 2 (TIMP2), actinin alpha 1(ACTN1), thrombospondin 1 (THBS1), fibromodulin (FMOD), heparan sulfate proteoglycan 2 (HSPG2), and syndecan 3 (SDC3), among others (Fig T, panels B–E, in S1 Text). The myoepithelial cells—which reside on the basement membrane at the edge of the epithelial compartment—are thus a major contributor and remodeler of the basement membrane and ECM. This is a significant role that diverges from that of the luminal epithelial cells. However, the luminal cells were a major producer of matrilysin (MMP7) that, in addition to several ECM proteins, cleaves the milk protein casein, which we also found differentially expressed by ER^Neg^ luminal cells.

#### Muscle contraction (2v3)

A defining feature of myoepithelial cells has long been their expression of smooth muscle actin [72], which allows them to contract when stimulated by oxytocin, e.g., during lactation. It was thus reassuring to find muscle contraction (FDR = 0.0085%) and associated pathways enriched in myoepithelial cells, including smooth muscle contraction, phase 0 rapid depolarization, and cardiac conduction—the latter sharing similar genes despite being a pathway in the heart (FDR = 0.0707%, 2.011%, 0.148%, respectively, Fig 25 and Supplementary Data File 12 at 10.17605/OSF.IO/GFHC3). Myoepithelial cells highly expressed smooth muscle actins alpha-1 and gamma-2 (ACTA1, ACTG2), the voltage-gated sodium channel alpha subunit 3 (SCN3A), the calcium voltage-gated channel subunit L (CACNA1C), myosin heavy chain 11 (MYH11), myosin light chain kinase (MYLK), and leiomodin 1 (LMOD1, Fig T, panel F, in S1 Text) that contribute to the contractile ability of this cell type.

#### Ion channel transport (2v3)

Calcium is essential for myoepithelial contractility. Its intracellular availability is regulated by the transport of ions between the extracellular and intracellular spaces. This process was significantly enriched in myoepithelial cells, as we found ion channel transport and associated pathways enriched in myoepithelial cells (FDR = 5.99%, Fig 25). The enrichment was driven predominantly by these cell’s differential expression of anoctamin-1 (ANO1), chloride channel accessory-2 (CLCA2), and the intracellular calcium channels ryanodine receptors 1 and 3 (RYR1, RYR3, Fig T, panel G, in S1 Text). Furthermore, myoepithelial cells differentially expressed the transient receptor potential (TRP) cation channels TRPV6, TRPC1, TRPC4, TRPM3, TRPC6, and TRPV2 (TRP channels, FDR = 1.59%). TRP channels are calcium permeable and convert external stimuli into electrical or chemical signals [73] (Fig T, panel G, in S1 Text). These pathways likely contribute to regulating myoepithelial cells’ intracellular hyperpolarization/depolarization and contractility.

#### Cell signaling (2v3)

We identified the enrichment of 81 cell signaling pathways between the ER^Neg^ and myoepithelial cells. Most of these (62) were enriched within the myoepithelial cells. The bulk of these pathways involved receptor tyrosine kinases (23/62), G-protein coupled receptors (13/62), Notch (9/62), Wnt (8/62), and Hedgehog pathways (6/62). Although the ER^Neg^ luminal cells differentially expressed KIT and were enriched with both signaling by SCF Kit (FDR = 5.7%) and ERBB2 signaling pathways, the myoepithelial cells were enriched with 20 receptor tyrosine kinase pathways, most at a conservative false discovery rate of less than 1% (13/20, Supplementary Data File 12 at 10.17605/OSF.IO/GFHC3). These pathways involved MET, neurotrophic tyrosine receptor kinases, platelet-derived growth factor, vascular endothelial growth factor, fibroblast growth factor, and insulin-like growth factor pathways (Fig 25). For example, the enrichment of the FGFR signaling pathways signaling by FGFR, signaling by FGFR1, and signaling by FGFR2 (FDR = 0.328%, 0.822%, and 4.00%) was driven by their differential expression of fibroblast growth factors 1, 2, and 7 (FGF1, FGF2, FGF7); FGF receptors 1, 2, and 4 (FGFR1, FGFR2, FGFR4); and the ras proto-oncogene (HRAS, Fig T, panel H, in S1 Text).

The enrichment of Notch signaling pathways split between the luminal and myoepithelial cells. Signaling by notch and signaling by notch4 were enriched in myoepithelial cells (FDR = 0.0268%, 0.059%), as these cells differentially expressed smooth muscle actin (ACTA2) and notch ligands, JAG1 and JAG2 (Fig T, panel H, in S1 Text). They also had slightly elevated levels of many proteosome subunits that help compose this pathway. In contrast, ER^Neg^ luminal cells were enriched with Notch 1, 2, and 3 signaling pathways (FDR = 3.38%, 7.75%, 1.99%) due to their differential expression of the Notch receptors, NOTCH2 and NOTCH3; the downstream effector HEY2, delta/notch-like EGF repeat containing (DNER) and EGF, and other pathway members (Fig T, panel J, in S1 Text).

Other myoepithelial enriched pathways involved G-protein coupled receptor-, Wnt-, and Hedgehog-signaling. Differentially expressed genes from the 12 enriched G-protein coupled receptor pathways included G-protein coupled receptor 176 (GPR176), dopamine receptor (DR), and adenylate cyclase 5 (ADCY5). Enrichment of the Wnt and Hedgehog signaling pathways was driven by elevated transcript levels of these pathways’ downstream effectors, which included transcription factor 7 (TCF7), lymphoid enhancer-binding factor-1 (LEF1), GLI1, and GLI3 (Supplementary Data File 12 at 10.17605/OSF.IO/GFHC3).

#### Metabolism (2v3)

Within the 87 metabolism-related pathways enriched between luminal and myoepithelial cells, one of the larger emergent themes involved the metabolism of glycosaminoglycans (Fig 25). These pathways were enriched in myoepithelial cells and included glycosaminoglycan metabolism, chondroitin sulfate dermatan sulfate metabolism, and heparan sulfate heparin HS-GAG metabolism pathways (FDR = 3.76%, 5.50%, and 3.04%, Fig 25). Among the genes contributing to their enrichment included key enzymes in GAG metabolism, e.g., (i) hyaluronan synthase 3 (HAS3), which catalyzes the addition of acetylglucosamine and glucuronic acid monosaccharides to hyaluronan polymers during hyaluronic acid synthesis; (ii) chondroitin-6 sulfotransferase-3 (CHST3), which catalyzes the sulfation of chondroitin; and (iii) the heparan sulfate proteoglycan glypican-3 (GPC3), an ECM glycoprotein that binds to, and modulates, several signaling pathways (Fig T, panel K, in S1 Text).

A characteristic feature of epithelial cells is their secretion and release of mucins from their apical surfaces [74]. Not surprisingly, we found the O-linked glycosylation of mucins pathway enriched in ER^Neg^ luminal epithelial cells (Fig 25). Luminal cells notably expressed mucin-1 (MUC1), which is a marker in our FACS panel and was used to sort the luminal cells. We found many other mucins expressed by the luminal cells, including MUC15 and MUC16 (Fig U, panels A–E, in S1 Text). Mucins are highly glycosylated proteins and a component of milk, where they provide antimicrobial resistance in the GI tract of breastfed infants [75]. These mucins are derived from the luminal epithelial cells.

The metabolism of steroids was another large theme in the network, which was enriched within ER^Neg^ luminal cells (FDR = 0.998%, Fig 25 and Supplementary Data File 12 at 10.17605/OSF.IO/GFHC3). These cells differentially expressed hydroxysteroid dehydrogenases 17B2 and 17B4 (HSD17B2 and HSD17B4)—essential enzymes involved in the production of the androgen and estrogen sex hormones [76]. Additionally, luminal cells highly expressed other steroid hormone regulators, HSD11B1 and steroid 5-alpha-reductase (SRD5A3), as well as the cytochrome p450s (CYP24A1 and CYP24B1), which function in Vitamin D metabolism (Fig T, panel L, in S1 Text). We found these genes were highly expressed by both luminal cell types, indicating steroid metabolism is a general feature of the luminal lineage.

The ER^Neg^ luminal cells were also enriched with other metabolic pathways, such as fatty acid metabolism (FDR = 6.04%, Fig 25 and Supplementary Data File 12 at 10.17605/OSF.IO/GFHC3), as they differentially expressed acyl synthetase long-chain family members 1, 3, 5, and 6 (ACSL1, ACSL3, ACSL5, ACSL6), aldo-keto reductase family member C3 (AKR1C3), glutathione peroxidase 2 (GPX2), and carnitine O-acetyltransferase (CRAT, Fig T, panel M, in S1 Text). The acyl synthetase enzymes catalyze long chain fatty acids into active fatty acyl-CoA esters, which is a critical step in beta-oxidation and lipid biosynthesis. The roughly 10-fold difference in acyl synthetase transcripts between luminal and myoepithelial cells indicates that luminal cells have different metabolic demands requiring the processing of long-chain fatty acids.

In our analysis of the citric acid cycle pathway, we found most genes in this pathway were uniformly expressed across the entire spectrum of cell types. However, there were 3 notable exceptions. The first, which occurred in both luminal cell types, was their pronounced differential expression of both isoforms of isocitrate dehydrogenase (IDH1, IDH2), which operate in the mitochondria to regulate 2-hydroxyglutarate, alpha-ketoglutarate, and isocitrate (adj. *p*-values = 5.2e-5 and 2.1e-13). Another differentially expressed TCA cycle gene was pyruvate decarboxylase (PC), which was repressed in both endothelial cell types. Finally, we found phosphoenolpyruvate carboxykinase-1 (PCK1) expression was limited to pericytes and adipocytes (Fig U, panel F, in S1 Text).

### ER^Neg^ luminal epithelial versus Vascular endothelial cells (Pop2 versus Pop9)

Histologists consider endothelial cells specialized epithelial cells because they exhibit classical epithelial features [15]. For example, vascular endothelial cells and epithelial cells both form the lumen of their respective structures (e.g., vessels, ducts), use tight junctions for cell–cell adhesion, and rest on a basal lamina. However, when we analyzed the breast cell transcriptomes by PCA (Fig 7A), we found that endothelial cells projected more closely to mesenchymal cell types than to the epithelial cells, which may reflect their mesodermal origin. We found this curious and decided to compare the features of vascular endothelial cells to the abundant ER^Neg^ luminal epithelial cell type (FACS populations #9 and #2).

**Fig 26:**
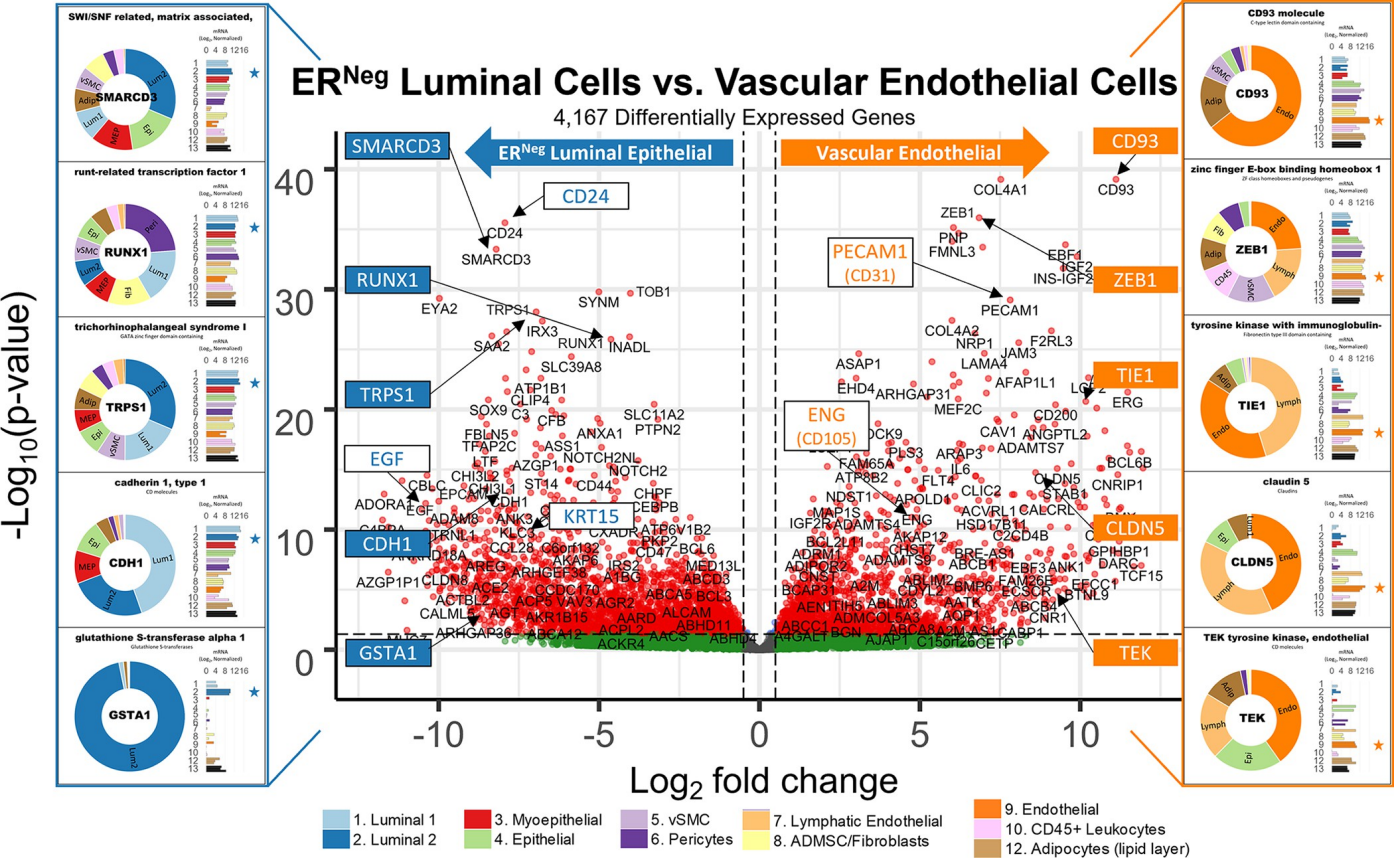
Differences between ER^Neg^ luminal and vascular endothelial cell types. A contrast of expressed transcripts between ER^Neg^ luminal and vascular endothelial cells revealed 4,167 differentially expressed genes (DeSeq2/Benjamini–Hochberg adjusted *p*-value ≤ 0.1; Supplementary Data File 7, 2v9 at 10.17605/OSF.IO/GFHC3). All expressed genes are displayed. The horizontal dashed lines (separating red and green dots) represent an adj. *p*-value of 0.1. Established cell type markers (of each cell type) are denoted. mRNA expression values for ten genes (indicated by filled, color-coordinated boxes) are shown at the left and right of the volcano plot. Color-coded stars in the bar graphs indicate the cell type being contrasted that had the higher transcript level, i.e., ER^Neg^(blue) and vascular endothelial cells (orange).

Comparing transcripts between vascular endothelial cells and ER^Neg^ luminal cells, we identified 4,167 differentially expressed genes (Fig 26). Of those, 1,652 were differentially expressed by the endothelial cells, which included endothelial markers: platelet/endothelial cell adhesion molecule (PECAM1/CD31), CD93, TIE1, and TEK tyrosine kinase. In contrast, luminal cells differentially expressed the transcriptional regulator, SMARCD3, keratin 15 (KRT15), CD24, cadherin 1 (CDH1), and transcriptional repressor GATA binding-1 (TRPS1, Fig 26 and Supplementary Data File 7, 2v9 at 10.17605/OSF.IO/GFHC3 [56]). We ranked all expressed genes by their degree of differential expression and used this list for pathway analysis. GSEA revealed 500 pathways enriched between ER^Neg^ luminal and vascular endothelial cells (FDR ≤ 10%), which provided insights into the different biological pathways and systems used by each cell type (Supplementary Data File 12 at 10.17605/OSF.IO/GFHC3).

#### Hemostasis (2v9)

**Fig 27:**
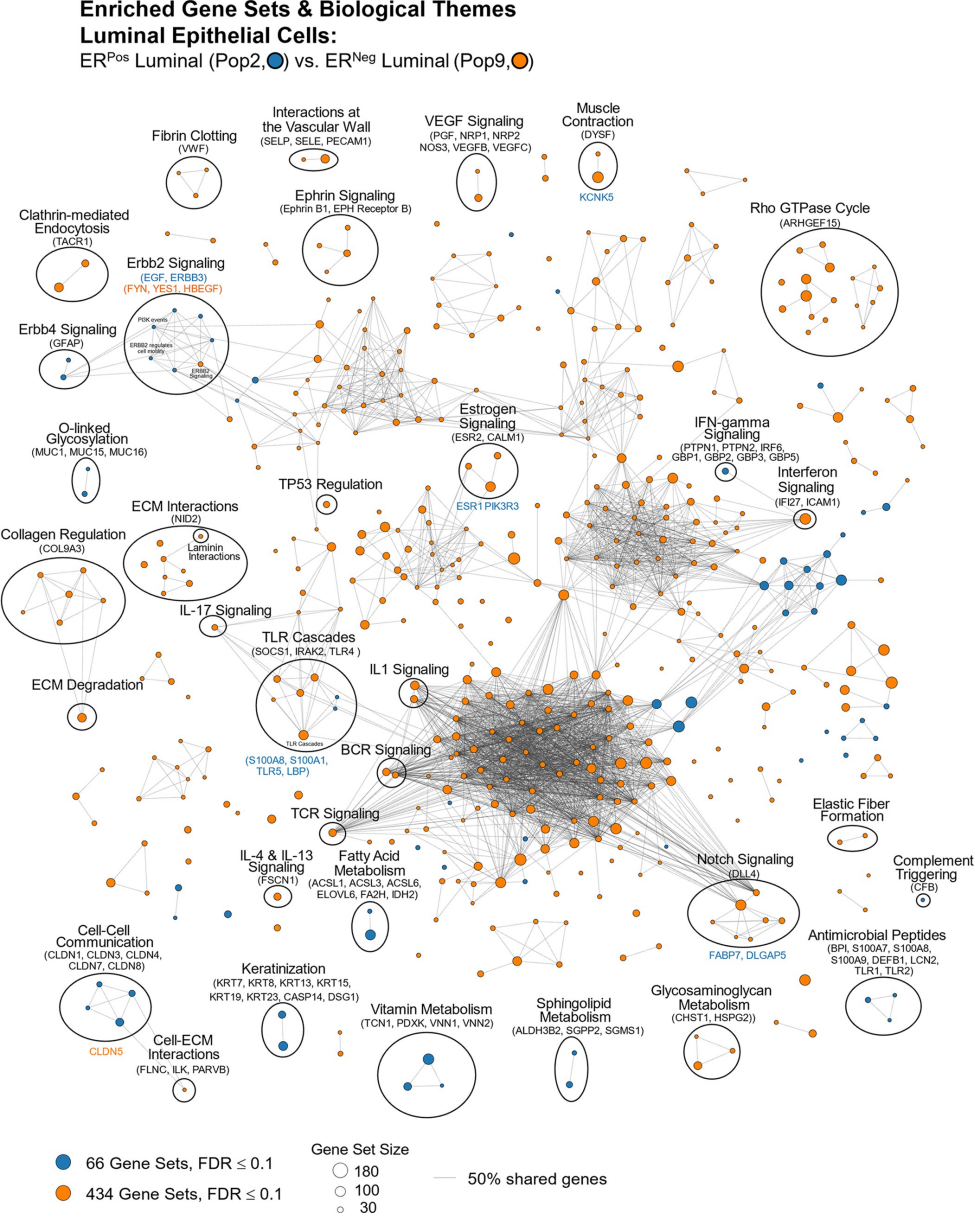
The ER^Neg^ luminal and vascular endothelial cells’ enriched pathways. Visualization of pathways enriched among ER^Pos^ luminal and vascular endothelial cell types (Enrichment Map/Cytoscape). All enriched pathways with an FDR ≤ 0.1 appear on the map (nodes, color-coded by cell type). Pathways (nodes) are connected when they share 50% of their genes (or greater). Node size corresponds to the number of genes in the pathway, as indicated (bottom left). Select biological themes were encircled and annotated. Highly differentially expressed genes are indicated (above the theme when it coincided with the attributed cell type—and below if it was expressed by the contrasted cell type). Samples are color-coded. Due to space constraints and a large number of presented genes, their names (rows of the heatmap) must be inspected digitally (magnified in the PDF). The names of each pathway are also available in the supplemental Cytoscape file and Pathway Analyzer tool (Supplementary Data Files 10 and 12 at 10.17605/OSF.IO/GFHC3). FDR, false discovery rate.

One of the most significantly enriched pathways, cell surface interactions at the vascular wall (FDR < 0.001%), was found in vascular endothelial cells—cells that indeed form the vascular wall (Fig 27 and Supplementary Data File 10 at 10.17605/OSF.IO/GFHC3). Endothelial cells differentially expressed selectins E, L, and P (SELE, SELL, SELP)—which mediate cell-to-cell adhesion; endothelial cell adhesion molecule (ESAM); junctional adhesion molecule 2 (JAM3); platelet/endothelial cell adhesion molecule (PECAM1); and the angiopoietin receptor TEK tyrosine kinase (TEK, Fig V, panel A, in S1 Text). These gene products regulate leukocyte adhesion to vessel walls during an inflammatory response and facilitate leukocyte migration. In addition, endothelial cells differentially expressed VWF—a historical marker of endothelial cells, thrombomodulin (THBD), and coagulation factors F2R and F3 (Fig V, panel B, in S1 Text). These contributed to the enrichment of formation of fibrin clot (clotting cascade, FDR = 3.04%). This is an essential step for blood clotting after injury, indicating that endothelial cells supply critical factors for this process, thus distinguishing them from other epithelial cell types. Notably, several of these pathway’s genes are shared by mesenchymal perivascular cells (e.g., ITGA5, ESAM, ANGPT); this shared expression contributes to the mesenchymal alignment of endothelial cells in the PCA projection (Fig 7A).

#### Keratinization (2v9)

Another divergent feature of endothelial and epithelial cells was the keratinization that occurred in ER^Neg^ luminal cells (keratinization, FDR < 0.001%, Fig 27 and Supplementary Data File 12 at 10.17605/OSF.IO/GFHC3). Keratin filaments are heteropolymers of different Type I and II keratins that confer distinct biophysical properties to cells, connecting the cells’ cytoskeleton to other cells and the basement membrane via desmosomes and hemidesmosomes [77]. We found ER^Neg^ luminal cells differentially expressed 15 keratins, including keratins 7, 8, 19, 6b, 23, 81, 15, 16, 17, and 18. In addition, they also differentially expressed caspase-14 (CASP14)—which is involved in the cornification of epithelia—and desmoglein-1 (DSG1), a component of desmosomes (Fig V, panel C, in S1 Text).

#### Cell communication (2v9)

Among the pathways associated with cell communication, we found that ER^Neg^ luminal cells were, compared to the vascular endothelial cells, enriched with the broad cell–cell communication pathway (FDR = 0.954%) and others involving tight junctions (Fig V, panel D, in S1 Text). These were cell junction organization (FDR = 1.8%) and tight junction interactions pathways (FDR = 0.835%, Fig V, panel E, in S1 Text). The enrichment resulted from ER^Neg^ luminal cells’ differential expression of laminin beta 3 (LAMB3), cadherins 1 and 3 (CDH1, CDH3), as well as the crumbs-cell-polarity complex component 3 (CRB3), which helps form tight junctions. Although the vascular endothelial cell types expressed claudins 5 and 14 rather exclusively, the ER^Neg^ luminal cells differentially expressed an overwhelming number of claudins (CLDN1, -3, -4, -7, -8, -10, -11, and -12), leading to the pathway’s luminal cell enrichment (Fig 16 and Fig V, panel E, in S1 Text). Another cell-communication pathway is the cell-extracellular matrix interactions pathway, which we found enriched in vascular endothelial cells (FDR < 0.001%). This pathway includes integrins that link ECM proteins to the cell’s cytoskeleton—with the help of scaffolding and focal adhesion proteins. Genes in this pathway differentially expressed by the vascular endothelial cells included filamin C (FLNC)—which was notably repressed by both luminal cell types (compared to all others), integrin-linked kinase (ILK), and parvin beta (PARVB, Fig V, panel F, in S1 Text and Supplementary Data File 12, 2v9 at 10.17605/OSF.IO/GFHC3).

#### Metabolism (2v9)

We identified 82 enriched pathways that involve the metabolism of nucleic acids, lipids, proteins, and other metabolites. Some of the more statistically significant pathways within ER^Neg^ luminal cells included the metabolism of vitamins and cofactors (FDR = 0.618%), metabolism of water-soluble vitamins and cofactors (FDR = 0.71%), and nicotinamide salvaging (FDR = 4.63%, Fig 27 and Supplementary Data File 12 at 10.17605/OSF.IO/GFHC3). Genes contributing to their enrichment included (i) vanins 1 and 2 (VNN1 and VNN2), which are involved in vitamin B5 metabolism; (ii) transcobalamin 1 (TCN1)—a vitamin 12 binding protein; and (iii) pyridoxal kinase (PDXK), which phosphorylates vitamin B6 (Fig V, panel G, in S1 Text). We also found sphingolipid metabolism enriched in luminal cells (FDR = 4.37%), as they differentially expressed sphingosine-1-phosphate phosphatase 2 (SGPP2), sphingomyelin synthase 1 (SGMS1), and aldehyde dehydrogenase 3 family member B2 (ALDH3B2, Fig V, panel H, in S1 Text). Additionally, fatty acid metabolism was enriched in ER^Neg^ luminal cells (FDR = 0.636). Enrichment resulted from the luminal cells’ differential expression of epoxide hydrolase 2 (EPHX2), ELOVL fatty acid elongase 6 (ELOVL6), acyl-CoA synthetase long-chain family members 1, 3, and 6 (ACSL1, ACSL3, ACSL6); fatty acid-2 hydrolase (FA2H); and the mitochondrial isocitrate dehydrogenase IDH2 (Fig V, panel I, in S1 Text). Glycosaminoglycan metabolism was another enriched pathway that contained substantial transcriptional differences (FDR = 0.95%, Fig 27 and Supplementary Data File 12 at 10.17605/OSF.IO/GFHC3). Leading to its enrichment in vascular endothelial cells was their differential expression of carbohydrate sulfotransferase 1 (CHST1), versican (VCAN), biglycan (BGN), decorin (DCN), and heparan sulfate proteoglycan 2 (HSPG2/perlecan, Fig V, panel J, in S1 Text). This is notable because endothelial surfaces and their surrounding ECM contain large quantities of heparan sulfate proteoglycans [78] that modulate vascular functions, such as sequestering growth factors (FGF, VEGF), regulating lipoprotein metabolism, and inhibiting clotting [79].

#### Immune system (2v9)

Pathways associated with the immune system were enriched in both luminal and endothelial cells (Fig 27). Nine were enriched in luminal cells and 39 in endothelial cells. Antimicrobial peptides (FDR = 0.058%), defensins (FDR = 0.349%), and beta-defensins (FDR = 1.19%) were enriched in luminal cells, which differentially expressed defensin beta 1 (DEFB1), S100 calcium-binding proteins A7A, A7, A8, and A9 (S100A7A, S100A7, S100A8, and S100A9), toll-like receptors 1 and 2 (TLR1 and TLR2), lipocalin 2 (LCN2), and bactericidal/permeability-increasing protein (BPI, Fig V, panel K, in S1 Text). Luminal cells also differentially expressed protein tyrosine phosphatase 2 and 6 (PTPN1 and PTPN6), interferon-inducible guanylate binding protein 1, 2, 3, and 5 (GBP1, GBP2, GBP3, and GBP5), and interferon regulatory factor 6 (IRF6)—which define the enrichment of interferon-gamma signaling in luminal cells (Fig 27 and Fig V, panel L, in S1 Text).

Interferon signaling was enriched in vascular endothelial cells, which differentially expressed intercellular adhesion molecule 1 (ICAM1) and interferon alpha-inducible protein 27 (IFI27, FDR = 0.512%, Fig 27 and Fig V, panel M, in S1 Text and Supplementary Data File 12 at 10.17605/OSF.IO/GFHC3). ICAM1 encodes a receptor for leukocyte adhesion protein LFA-1 (ITGAL) and is essential for leukocyte trans-endothelial migration [80]. Additionally, toll-like receptor cascades (FDR = 1.36%) were enriched in vascular endothelial cells, that differentially expressed suppressor of cytokine signaling (SOCS1), interleukin receptor-associated kinase 2 (IRAK2), and TLR4 (Fig 27 and Fig V, panel N, in S1 Text). Two pathways associated with TLR cascades were enriched in luminal cells: IRAK4 deficiency TLR2/4 and regulation of TLR by endogenous ligand (FDR = 0.926% and 4.94%, respectively, Fig 27). Notably, we discovered that enrichment of many pathways associated immune system was due to the expression of proteasome subunits. For example, enrichment of interferon signaling, TCR signaling, BCR signaling, and Interleukin-1 signaling were all affected by overexpression of the proteosome subunits PSMD2, PSMB6, and PSMB5, among others (FDRs < 0.001%, Fig 27, Fig V, panels O–Q, in S1 Text). Proteosomes function in peptide processing for many pathways, including those involved in antigenic peptide presentation during immune responses. However, protein modification is required for many other pathways like cell cycle progression, apoptosis, and DNA repair.

#### Cell signaling (2v9)

As expected, signaling by VEGF and VEGFR2 mediated vascular permeability were enriched in vascular endothelial cells (FDR = 0.005% and 6.81%, respectively), with differential expression of placental growth factor (PGF), neuropilin 1 and 2 (NRP1 and NRP2), nitric oxide synthase 3 (NOS3), and vascular endothelial growth factors B and C (VEGFB and VEGFC)—all of which are involved in VEGF-regulated angiogenesis (Fig 27, Fig V, panel R, in S1 Text)

Interestingly, signaling by ERBB2 was enriched in endothelial cells (FDR = 8.00%, Fig 27). The differentially expressed genes contributing to the enrichment of this pathway include FYN proto-oncogene (FYN), YES proto-oncogene (YES1), and heparin-binding EGF-like growth factor (HBEGF, Fig 27, Fig V, panel A, in S1 Text). However, the luminal cells differentially expressed ERBB2, ERBB3, ERBB4, phosphoinositide-3-kinase regulatory subunit 1 (PIK3R1), EGF, and EGFR, which define the enrichment of other pathways associated with ERBB2 signaling, like PI3K events in ERBB2 signaling and ERBB2 regulates cell motility (Fig 27).

One other surprising finding was, despite the higher levels of estrogen receptor α mRNA in ER^Neg^ luminal cells (so named because of their undetectable protein expression and lower mRNA levels compared to the ER^Pos^ luminal cells), we found estrogen receptor-mediated signaling and associated pathways enriched in vascular endothelial cells (FDR = 0.008%). After investigating other members of this pathway, we found vascular endothelial cells differentially expressed estrogen receptor β (ESR2), calmodulin-1 (CALM1), and the G protein subunits GNAI2, GNG2, GNG5, GNG11, GNB1, and GNB2, and endothelial nitric oxide synthetase (NOS3) that compose this pathway. (Fig 27 and Fig V, panel T, in S1 Text). This is noteworthy because endothelial cells respond to estrogen for nitric oxide and prostacyclin production [81]. Our data thus indicate that the bulk of estrogen’s effects on breast endothelium likely results through ERβ signaling.

### Lymphatic endothelial versus vascular endothelial cells (Pop7 versus Pop9)

Whereas the blood’s vascular system circulates blood throughout the body, the lymphatic system collects and filters interstitial fluid, ultimately returning it to the bloodstream—a process that helps maintain fluid homeostasis. Much like capillaries of the blood system, lymphatic vessels are also formed by endothelial cells, although the 2 vascular architectures are distinct (Fig 2D and 2F). We found lymphatic and vascular endothelial cells expressed similar markers, like CD31 (PECAM1) and VWF (Fig 8B) but diverge and expressed other genes that contribute to their individual functions. To explore the biological processes shaping these 2 endothelial cell types, we investigated their transcriptional differences.

Lymphatic endothelial cells are a rare cell type in the breast. Because of this, acquiring enough cells required for mRNA-sequencing proved challenging. However, by scaling up the number of cells stained—and through sheer brute force (approximately 15 h of continuous FACS sorting for each sample), we succeeded in purifying a sufficient number of cells from 2 samples (24,713 cells from N228 and 29,111 cells from N239, S1 Data). We detected roughly 21 million and 43 million paired end reads, respectively, from these purified lymphatic endothelial cells (N228 and N239). During quality control assessment of the RNA-sequencing data, we however found outliers and conflicts between the lymphatic cell replicates (Fig 4C). Nonetheless, the pair of lymphatic endothelial cell types clustered together well—in the hierarchical clustering dendrogram (Fig 6) and projection of principal components (Fig 7A). We thus retained the lymphatic endothelial samples for further analyses, yet, we did expect to find discordance between replicates for some genes.

**Fig 28:**
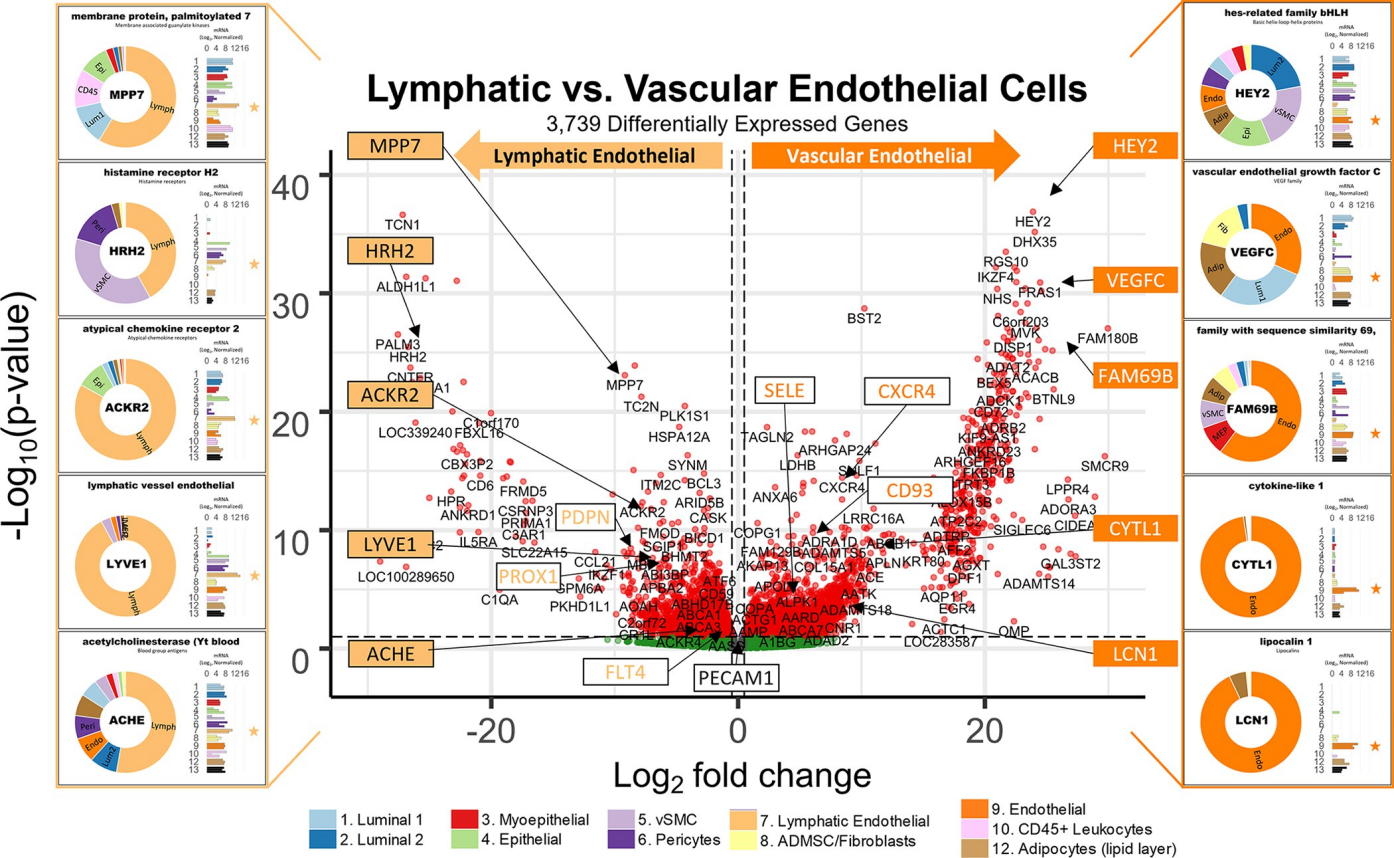
Differences between lymphatic and vascular endothelial cell types. A contrast of expressed transcripts between lymphatic and vascular endothelial cells revealed 3,739 differentially expressed genes (DeSeq2/Benjamini–Hochberg adjusted *p*-value ≤ 0.1, Supplementary Data File 7, 7v9 at 10.17605/OSF.IO/GFHC3). All expressed genes are displayed. The horizontal dashed lines (separating red and green dots) represent an adj. *p*-value of 0.1. Established cell type markers (of each cell type) are denoted. mRNA expression values for ten genes (indicated by filled, color-coordinated boxes) are shown at left and right of the volcano plot. Color-coded stars in the bar graphs indicate the cell type being contrasted that had the higher transcript level, i.e., lymphatic (light orange) and vascular (dark orange) endothelial cells.

When we compared transcript levels between lymphatic and vascular endothelial cell types, we identified 3,739 differentially expressed genes (Fig 28 and Supplementary Data File 7, 7v9 at 10.17605/OSF.IO/GFHC3 [56]). Lymphatic endothelial cells expressed the higher level of 1,462 of these, including podoplanin (PDPN), the marker we used to differentiate lymphatic cells in our FACS strategy (adj.*p-*value of 1.14E-7). We also found other lymphatic markers differentially expressed, such as “lymphatic vessel endothelial hyaluronan receptor1” (LYVE1), “vascular endothelial growth factor receptor-3” (FLT4), and prospero homeobox 1 (PROX1, Fig 28). In contrast, the vascular endothelial cells differentially expressed 2,277 genes. Among these were the vascular endothelial growth factor C (VEGFC), cytokine-like 1 (CYTL1), lipocalin 1 (LCN1), endothelial selectin (SELE), and the endothelial marker CD93 (Fig 28). After ranking the expressed genes by degree of differential expression, we performed GSEA, and discovered 240 pathways enriched between the lymphatic and endothelial cell types (FDR ≤ 10%, Supplementary Data File 12 at 10.17605/OSF.IO/GFHC3). Of these enriched pathways, we have concentrated only on those established from genes where there was concordance between lymphatic endothelial cell replicates.

#### Immune system (7v9)

**Fig 29:**
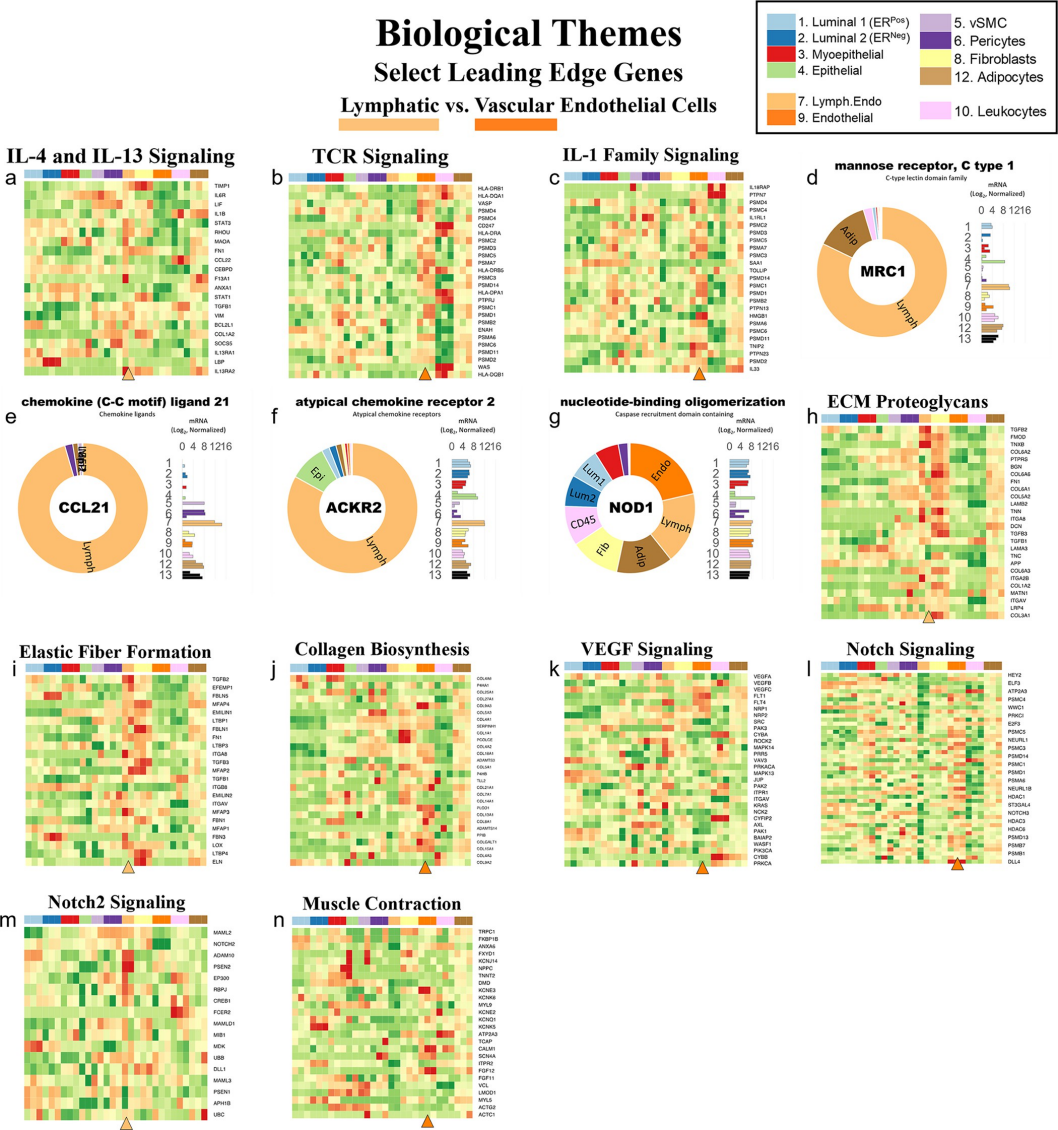
Transcript levels from enriched pathways. **(a-n)** Transcript levels of leading-edge genes from select pathways enriched in lymphatic and vascular endothelial cell (from 240 enriched pathways with an FDR of ≤ 10%, GSEA). VST gene levels are relative (normalized by row). Arrowheads under the heatmaps identify the cell type, either over- or under-expressing the pathway’s genes. (Heatmap data values are found in Supplementary Data File 12 at 10.17605/OSF.IO/GFHC3, select 7v9, 00_DS; donut data values are found in Supplementary Data File 13 at 10.17605/OSF.IO/GFHC3.) FDR, false discovery rate; GSEA, gene set enrichment analysis.

Both lymphatic and blood vascular systems are essential and active components of immune responses. Lymphatic vessels serve as conduits for lymphocyte migration from peripheral tissues to the lymph nodes, making them crucial to adaptive immune response. In contrast, vascular vessels are first responders to pathogen detection and danger-signal-sensing mechanisms in the bloodstream. Indeed, when we inspected the enrichment results, both cell types were enriched with immunity-related pathways. For instance, we found interleukin-4 and interleukin-13 signaling pathways enriched in lymphatic endothelial cells (FDR = 1.54%). Contributing to this enrichment was the lymphatic cells’ differential expression of transforming growth factor-beta 1 (TGFB1) and IL13 receptors A1 and A2 (IL13RA1 and IL13RA2, Fig 29A). In contrast, the vascular endothelial cells were enriched with the TCR signaling and interleukin-1 family signaling pathways (FDR = 0.009% and 0.394%, Supplementary Data File 12 at 10.17605/OSF.IO/GFHC3). The TCR signaling pathway’s most highly differentially expressed genes included major histocompatibility complexes HLA-DRB1 and HLA-DQA1, vasodilatory-stimulated phosphoprotein (VASP), and the TCR subunit CD247 (Fig 29B), whereas differentially expressed genes of the IL-1 signaling pathway included HMGB1, which encodes the “high mobility group box 1” protein that promotes inflammatory responses to infectious signals, and protein tyrosine phosphatase 7 (PTPN7) which regulates TCR signaling (Fig 29C).

Other immune-related genes that we identified as being differentially expressed by lymphatic endothelial cells but were not associated with the above pathways included MRC1, TLR4, CCL21, and ACKR2 (Fig 23 and Fig R, panel G, in S1 Text). MRC1 encodes mannose receptor 1, a cognate binding partner of L-selectin—that is expressed by lymphocytes and facilitates their binding to endothelial cells. Toll-like receptor 4 (TLR4) plays a crucial role in mediating the innate immune response to bacterial lipopolysaccharide (LPS), where it engages other levers of the immune response upon binding to bacterial LPS [82]; chemokine ligand 21 (CCL21) mediates homing of lymphocytes to lymph organs, whereas the atypical chemokine receptor 2 (ACKR2) regulates cellular interactions with inflammatory leukocytes [83]. Interestingly, we found lymphatic and vascular endothelial cells both expressed the pattern recognition receptor NOD1, which detects bacterial peptidoglycan fragments and initiates immune responses to gram-negative bacteria [84] (Fig 29G). There is thus ample evidence showing the respective endothelial cell types are interacting with cells of the immune system and actively participating in immune surveillance—in distinct ways.

#### ECM organization (7v9)

While vascular endothelial cells are surrounded by a continuous basement membrane, the basement membrane surrounding lymphatic endothelial cells is thin and discontinuous (Fig 2D and Fig C in S1 Text) [8]. We thus expected to find differences in their respective expression of extracellular matrix components. After performing pathway analysis, we indeed found enrichment of ECM proteoglycans in the lymphatic cells (FDR = 1.09%). Contributing to the pathway’s enrichment was the strong lymphatic expression of all 3 TGFβ ligands (TGFB1, -B2 and -B3), fibromodulin (FMOD), biglycan (BGN), fibronectin (FN1), tenascin XB (TNXB), integrin alpha 8 (ITGA8), collagen 6A2 (COL6A2), and laminin beta 2 (LAMB2, Fig 29H). Elastic fibers appear to be strongly associated with lymphatics, as we found the elastic fiber formation pathway highly enriched in this cell type (FDR = 0.5%). Twenty-four genes in the elastic fiber pathway were elevated in lymphatic endothelial cells, including “elastin microfibril interfacer 1” (EMILIN1) and latent transforming growth factor beta (LTBP1). In a prior section, we introduced the fibulin gene family, where we discussed their importance to elastic fibers and identified several family members (EFEMP1, FBLN1, FBLN5) differentially expressed by lymphatic endothelial cells—these genes are also members of the elastic fiber formation pathway (Fig 29I).

The only ECM-related pathway enriched in vascular endothelial cells was collagen biosynthesis and modifying enzymes (FDR = 6.65%). Differentially expressed genes contributing to its enrichment included the collagens COL9A3, COL4A1, COL4A2, COL18A1, COL21A1, COL13A1, COL8A1, COL15A1; procollagen-lysine 2-oxoglutarate 5-dioxygenase 1 (PLOD1); and collagen beta (1-O) galactosyltransferase 1 (COLGALT1, Fig 29J). The type IV collagens are a major structural component of basement membranes, indicating that endothelial cells collaborate with pericytes to produce the vascular basement membrane.

#### Cell signaling (7v9)

VEGF signaling is a critical regulator of vascular development. Interestingly, we found signaling by VEGF enriched in lymphatic endothelial cells (FDR = 0.563%) due to their elevated expression of 55 of the 105 genes in the pathway, despite the vascular endothelial cells’ differential expression of several key pathway members, like vascular endothelial growth factor c (VEGFC), placental growth factor (PGF), and VEGF Receptor-1 (VEGFR1/FLT1). The lymphatic endothelial cells, in contrast, expressed higher levels of other pathway members, like: “p21 (Cdc42/Rac)-activating protein 3” (PAK3), cytochrome b245 (CYBA), the mTORC2 target PRR5, and VEGFR3 (FLT4, Fig 29K).

Distinct Notch signaling pathways were enriched in both endothelial cell types. We found the broader Signaling by notch pathway enriched in vascular endothelial cells (FDR = 0.434%), whereas the signaling by notch2 pathway was enriched in lymphatic endothelial cells—at a false discovery rate of 9.97% (near the 10% cut-off). Of the 174 genes in the signaling by notch pathway, 103 were differentially expressed by vascular endothelial cells, including genes encoding the Notch ligands jagged 1 (JAG1), jagged 2 (JAG2), and delta-like 4 (DLL4, Fig 29L). Notch2 transcript levels were higher in the lymphatic cells, which, compared to all other breast cell types, appeared to stem from its repression within vascular endothelial cells (Fig 29M). This contributed to signaling by notch2 pathway’s enrichment in lymphatics, along with robust lymphatic expression of other pathway members, like ADAM metallopeptidase domain 10 (ADAM10) and mastermind-like transcription coactivator (MAML2).

#### Muscle contraction (7v9)

We found the muscle contraction pathway enriched in vascular endothelial cells (FDR = 4.30%, Supplementary Data File 12 at 10.17605/OSF.IO/GFHC3). However, both endothelial cell types did not appreciably express skeletal actin (ACTA1) or smooth muscle actins (ACTA2 or ACTG2), so they do not appear to be contractile. Of course, this is consistent with contractile smooth muscle cells being the primary regulator of vessel diameter. Instead, we found the vascular endothelial cells’ enrichment of the muscle contraction pathway primarily resulted from the differential expression of (i) the calcium-permeable transient receptor potential cation channel (TRPC1); (ii) the calcium-dependent membrane protein annexin A6 (ANXA6); and (iii) several potassium channels, including KCNE3, KCNK5, and KCNK6. These intermediate and small conductance channels play an essential role by regulating the release of endothelial cell autacoids, like nitric oxide [85], and were noticeably absent in lymphatic endothelial cells (Fig 29N).

### Fibroblasts (ADMSCs) versus adipocytes (Pop8 versus Pop12)

As epithelial cells form the parenchyma of the breast, the bulk of the tissue stroma is formed by the mesenchymal cell types (adipocytes, fibroblasts, vascular smooth muscle cells, and pericytes), with endothelial cells and a spectrum of leukocytes providing key elements [8]. We did not quantify adipocytes by FACS, but we could deduce from the RNA-sequencing data that adipocytes are one of, if not the most, abundant breast cell type. This was inferred from the covariance analysis and hierarchical clustering that grouped adipocytes with the whole tissue control samples, indicating they were disproportionately comprised of adipocytes (Figs 4B and 6). The remaining cell types were assessed by FACS, and it came as no surprise that fibroblasts were also abundant (Fig 1B).

**Fig 30:**
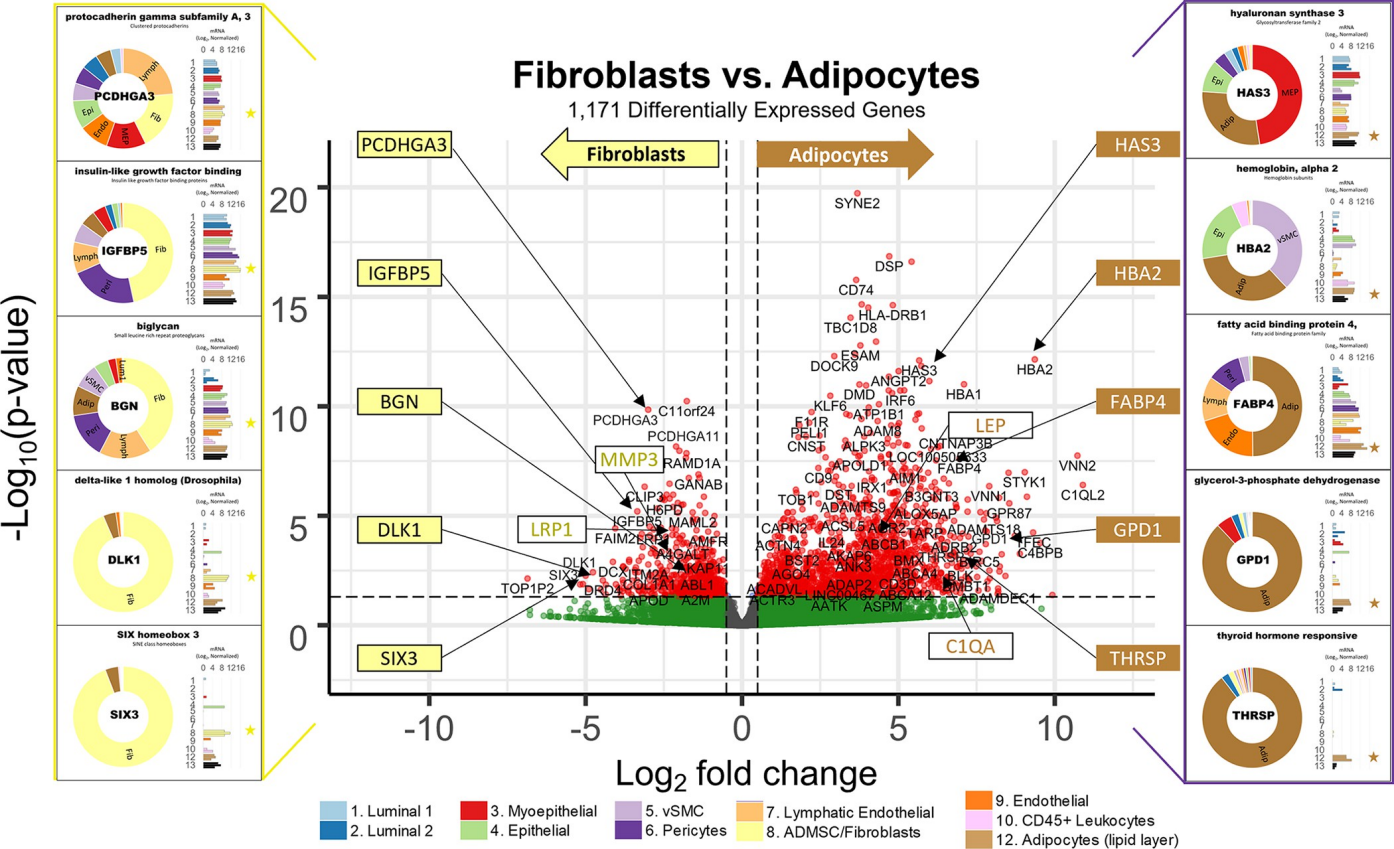
Differences between fibroblasts and adipocytes. A contrast of expressed transcripts between fibroblasts and adipocytes revealed 1,171 differentially expressed genes (DeSeq2/Benjamini–Hochberg adjusted *p*-value ≤0.1; Supplementary Data File 7, 8v12 at 10.17605/OSF.IO/GFHC3). All expressed genes are displayed. The horizontal dashed lines (separating red and green dots) represent an adj. *p*-value of 0.1. Established cell type markers (of each cell type) are denoted. mRNA expression values for 10 genes (indicated by filled, color-coordinated boxes) are shown at left and right of the volcano plot. Color-coded stars in the bar graphs identify the position of the fibroblasts (yellow) and adipocytes (brown).

**Fig 31:**
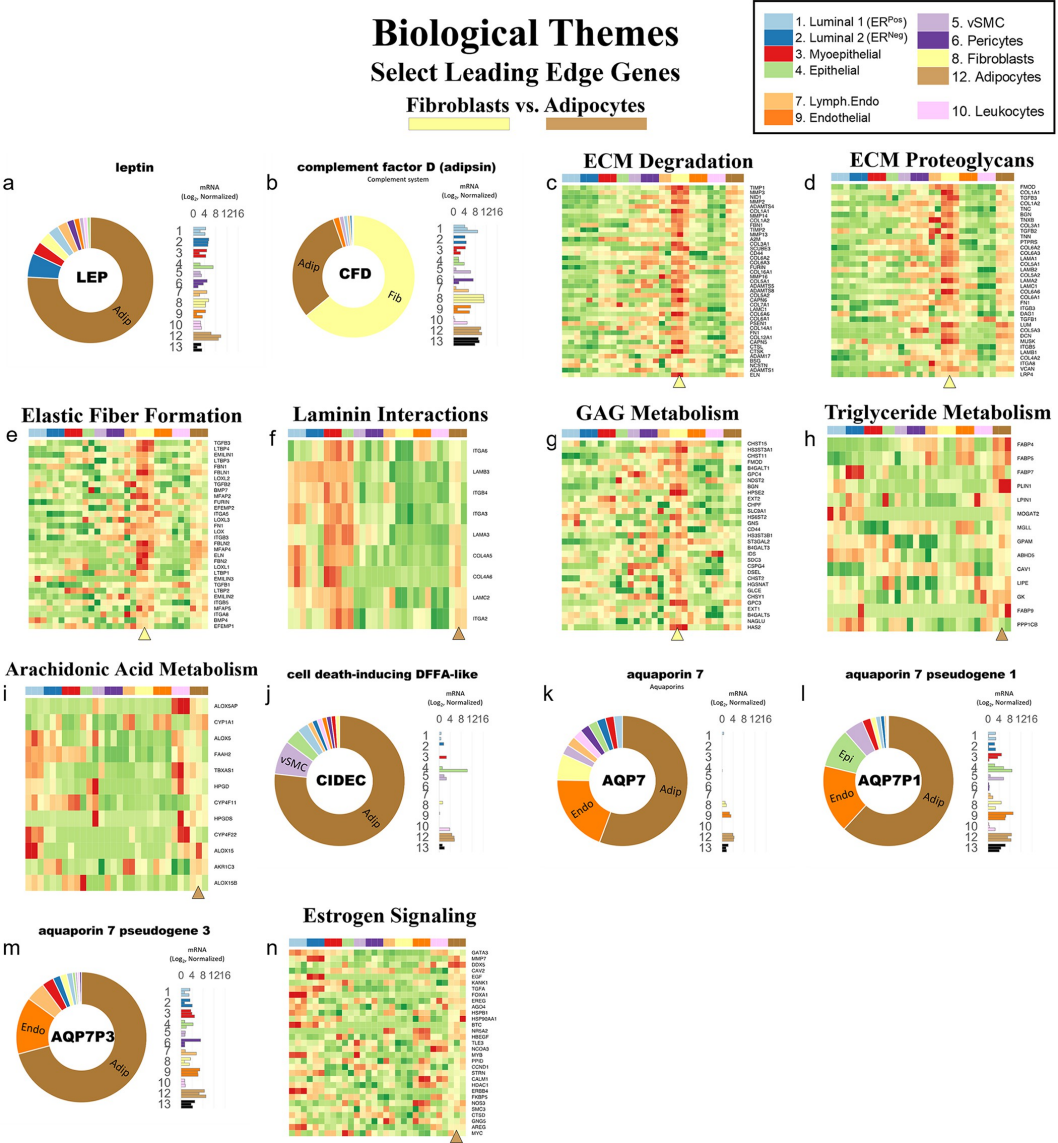
Transcript levels from enriched pathways. **(a-n)** Transcript levels of leading-edge genes from select pathways enriched in fibroblasts and adipocytes (from 387 enriched pathways with a FDR of ≤ 10%, GSEA). VST gene levels are relative (normalized by row). Arrowheads under the heatmaps identify the cell type, either over- or under-expressing the pathway’s genes. (Heatmap data values are found in Supplementary Data File 12 at 10.17605/OSF.IO/GFHC3, select 8v12, 00_DS; donut data values are found in Supplementary Data File 13 at 10.17605/OSF.IO/GFHC3.) FDR, false discovery rate; GSEA, gene set enrichment analysis.

Fibroblasts are spindle-shaped cells that predominantly provide the tissue’s foundation—a collagenous-rich matrix of fibrillar extracellular proteins, which fibroblasts shape through the secretion of growth factors, proteoglycans, and matrix-bound proteases. Fibroblasts are the archetypical mesenchymal cell type, existing within a developmental lineage that is shared with adipocytes. Previously, we described how breast fibroblasts express mesenchymal stem cell markers (CD10, CD34, CD73, Thy1, CD44, CD105) and displayed a functional multi-lineage potential when cultured under osteogenic or adipogenic conditions [8]. For these reasons, we designated Pop8 fibroblasts as adipocyte-derived mesenchymal stem cells (ADMSCs) but commonly refer to them as fibroblasts. In our cultures, and especially within the tissue, adipocytes appeared as large, spherical cells containing a single large lipid droplet (unilocular adipocytes) that stained for neutral lipids [8] (Fig 2A and 2C, Fig B, panel A, and Fig C, panel A, in S1 Text). Adipocytes notably serve as the body’s chief storage depot of triglycerides and other acylglycerols and regulate energy metabolism through secretion of adipokines [86], e.g., leptin hormone (LEP) and adipsin (CFD), which we found highly expressed in the pop12 lipid (adipocyte) samples (Figs 30, 31A, and 31B). To establish the different cellular processes shaping adipocytes and fibroblasts, we examined their expressed transcripts.

In contrast to the other breast cell types, freshly isolated adipocytes were not FACS purified prior to RNA-sequencing, and therefore results should be interpreted differently. Adipocytes were instead isolated from the phase-separated lipid layer of the collagenase tissues digests, which we had visually inspected for contaminating cellular content (Hoechst staining). The number of paired-end mRNA fragments measured from the RNA isolated from the lipid layers was excellent (34 to 45 million fragments), and the replicates nicely clustered together (Figs 4B amd 6, Fig I panel A and 8A). However, one sample did stray in the projection of PC1 and PC2 (Fig 7A), indicating some discordance between samples. We also noticed, in all 3 adipocyte replicates, low transcript levels for many genes (e.g., this can be observed in the many heatmaps presented herein, such as those containing the claudins and gap junction proteins; Figs 16 and 17). These adipocyte samples thus have a higher noise level than that observed in the FACS-purified samples, which is to be expected given the purity and excellent quality of those samples. The noise is likely derived from a fraction of contaminating cells in the samples, such as leukocytes. Therefore, we have prioritized genes and pathways in our analyses that are not heavily associated with immune response or where there is no evidence of robust adipocyte mRNA levels. With the caveat that the adipocyte samples should be interpreted differently, we explored the nature of adipocytes by comparing them with fibroblasts.

Pairwise comparison of RNA transcripts from adipocytes and fibroblasts revealed 1,171 differentially expressed genes. This was notably the lowest number of differentially expressed genes between any cell type comparison, similar to the number differentially expressed between the 2 luminal epithelial cell types (1,301, Fig 20). We anticipated fewer DE genes given the similarity and shared lineage of adipocytes and fibroblasts. Of the 1,171 differentially expressed genes we identified, 207 were more highly expressed by fibroblasts, and 964 were more highly expressed by adipocytes (Fig 30 and Supplementary Data File 7 Data, 8v12 at 10.17605/OSF.IO/GFHC3 [56]).

When we investigated the identity of the differentially expressed genes, we found fibroblasts expressed the ECM protein biglycan, delta-like non-canonical Notch ligand 1 (DLK1), matrix metalloproteinase 3 (MMP3), and others. In contrast, adipocytes differentially expressed leptin (LEP), thyroid hormone-responsive protein (THRSP), and glycerol-3-phosphate dehydrogenase (GPD1), along with 961 other genes (Fig 30). After performing GSEA, we identified 387 pathways enriched between the 2 cell types (FDR ≤ 20%), with 69 pathways in fibroblasts and 318 in adipocytes (Supplementary Data File 12 at 10.17605/OSF.IO/GFHC3). Due to the large number of immune-related genes and pathways in adipocyte samples, we increased the false discovery rate cutoff to 20% for this comparison, which resulted in the enrichment of more pathways related to fat storage and metabolism.

#### Extracellular matrix organization (8v12)

Twelve pathways associated with ECM organization were enriched in fibroblasts, including degradation of the extracellular matrix, ECM proteoglycans, and elastic fiber formation (FDR < 0.001%, < 0.001%, and = 0.090%, respectively, Supplementary Data File 12 at 10.17605/OSF.IO/GFHC3). Fibroblasts differentially expressed genes contributing to these pathways, including TIMP metallopeptidase inhibitor 1 and 2 (TIMP1 and TIMP2); metallopeptidases 2, 3, 13, 14, and 16 (MMP2, MMP3, MMP13, MMP14, and MMP16); fibulin-1 (FN1); fibrillin-1 (FBN1); and elastin (ELN, Fig 31C–31E). There were 2 pathways associated with ECM organization enriched in adipocytes, including laminin interactions and anchoring fibril formation (FDR = 3.23% and 9.97%, Fig 31F and Supplementary Data File 12). Adipocytes differentially expressed integrins A6, B4, A3, and A2; laminins B3, A3, and C3; and collagens 4A5 and 4A6, which contributed to the enrichment of these pathways (Fig 31F).

#### Metabolism (8v12)

Included in the enriched metabolic pathways was Glycosaminoglycan metabolism that was associated with fibroblasts (FDR < 0.001%). This pathway’s genes included heparanase-2 (HPSE2), hyaluronan synthase-2 (HAS2), and glypican-3 (GPC3, Fig 31G). In contrast, we found adipocytes enriched in pathways associated with lipid metabolism, that included triglyceride metabolism, triglyceride catabolism, and arachidonic acid metabolism (FDR = 15.5%, 15.5%, and 4.13%, Supplementary Data File 12 at 10.17605/OSF.IO/GFHC3 and Fig 31H). Differential expression of fatty acid binding proteins (FABP4, FABP5, FABP7, and FABP9) were among those genes contributing to enrichment of pathways associated with triglyceride metabolism and catabolism (Fig 31H). These intracellular fatty acid-binding proteins (FABPs) exhibit high affinity for a single long-chain fatty acids and are essential for triglyceride storage; they mediate intracellular fatty acid transport and interact with PPAR nuclear receptors [87]. Enrichment of arachidonic acid metabolism in adipocytes was defined by differential expression of arachidonate 5-lipoxygenase (ALOX5) and its activating protein (ALOX5AP, Fig 31I), which catalyzes the first 2 steps of the biosynthesis of leukotrienes, which are potent mediators of inflammation.

We also found adipocytes uniquely expressed cell death inducing DFFA like effector C (CIDEC, Fig 31J), which binds to lipid droplets and regulates their enlargement, thereby restricting lipolysis and favoring storage [88]. The adipocytes also uniquely expressed aquaporin AQP7 and the aquaporin pseudogenes AQP7P1 and AQP7P3 (Fig 31K–31M), which control water and glycerol transport and promote the catabolism of lipids in adipocytes [89].

#### Cell signaling (8v12)

Accumulation of adipose tissue is regulated in part by estrogen signaling [90]. Therefore, it is unsurprising that ESR-mediated signaling and its associated pathways were enriched in adipocytes (FDR = 0.110%), which differentially express the contributing genes GATA binding protein 3 (GATA3), caveolin 2 (CAV2), EGF, and epiregulin (EREG, Fig 31N). Adipocytes also expressed estrogen receptors 1 and 2 (ESR1 and ESR2), albeit at 10× lower levels lower than ER^Pos^ luminal epithelial cells but at a 4.3× higher level than fibroblasts (Fig 31N and Supplementary Data File 12 at 10.17605/OSF.IO/GFHC3).

### Pericytes versus fibroblasts (ADMSCs, Pop6 versus Pop8)

Whereas mesenchymal adipocytes contribute to the storage and mobilization of energy-rich metabolites, and mesenchymal fibroblasts secrete ECM proteins and enzymes that shape the extracellular stromal environment, the mesenchymal perivascular cells influence and modulate blood vessels. In a companion article [8], we described how we identified vascular smooth muscle cells and pericytes while devising methods to isolate and culture all breast cell types, finding these perivascular cells to be an elusive set of cell types. Pericytes have long been overlooked [91,92], largely stemming from their heterogeneity that, in turn, required a combinatoric assessment of markers to identify and purify them. After discovering how to reliably isolate and propagate breast pericytes, we found their microscopic appearance fascinating, as they had a stellate astrocyte-like morphology that was entirely distinct from fibroblasts and adipocytes [8]. However, it was difficult to tell the pericytes and fibroblasts apart at higher confluency. We were thus interested in exploring the biological processes defining these 2 similar mesenchymal cell types (i.e., FACS populations #6-pericytes and #8-ADMSC/fibroblasts).

**Fig 32:**
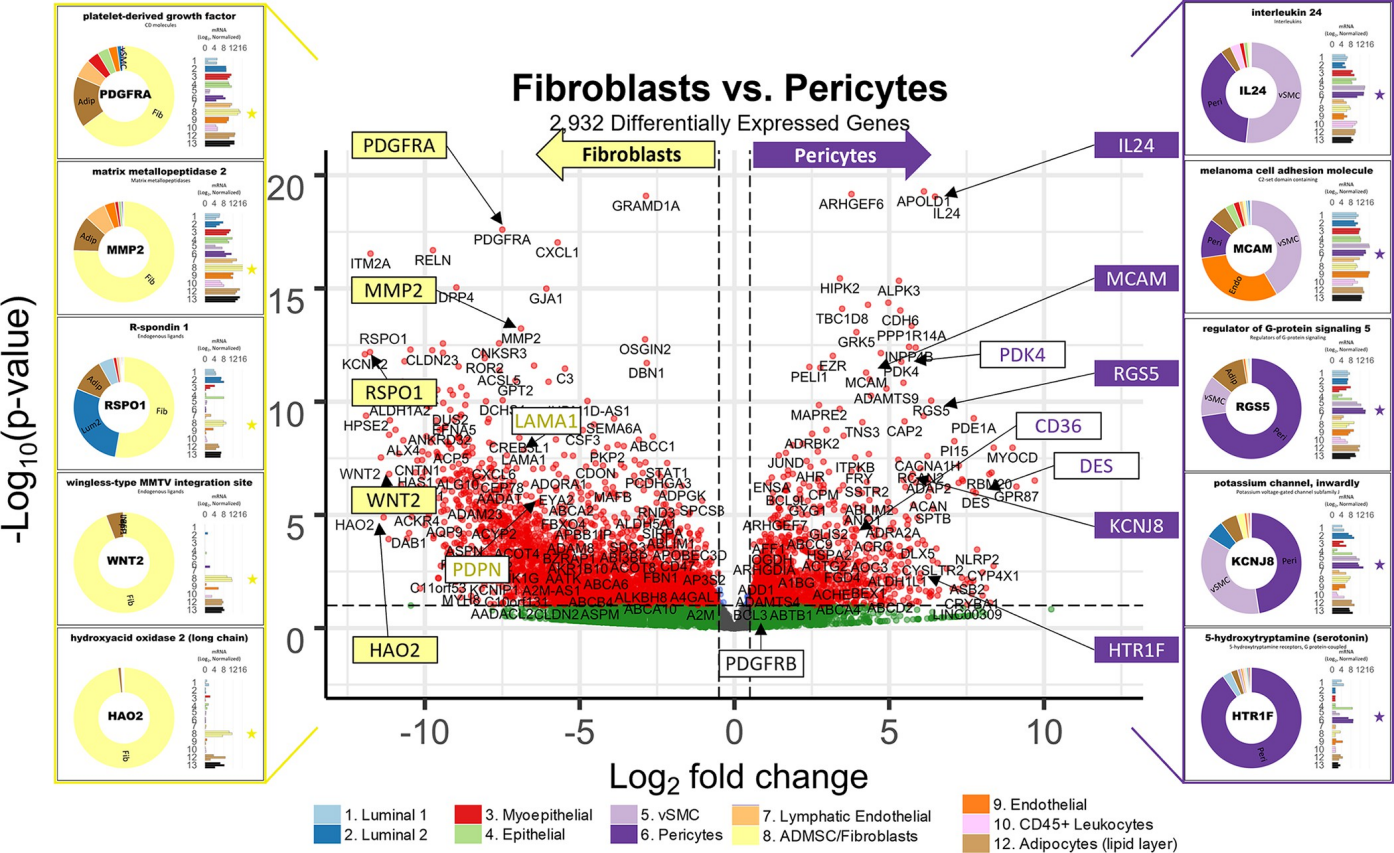
Differences between pericytes and fibroblasts. A contrast of expressed transcripts between pericytes and fibroblasts revealed 2,932 differentially expressed genes (DeSeq2/Benjamini–Hochberg adjusted *p*-value ≤0.1; Supplementary Data File 7, 6v8 at 10.17605/OSF.IO/GFHC3). All expressed genes are displayed. The horizontal dashed lines (separating red and green dots) represent an adj. *p*-value of 0.1. Established cell type markers (of each cell type) are denoted. mRNA expression values for 10 genes (indicated by filled, color-coordinated boxes) are shown at the left and right of the volcano plot. Color-coded stars in the bar graphs identify the position of the pericytes (purple) and fibroblasts (yellow).

Analysis of transcripts expressed by freshly isolated pericytes and fibroblasts revealed 2,932 differentially expressed genes (BH/FDR adj. *p*-value <0.1, Fig 32). Interestingly, these were not split evenly between the 2 cell types, as fibroblasts had roughly three times the number of DE genes than pericytes. Among these 2,187 genes, were platelet-derived growth factor receptor alpha (PDGFRA), matrix metallopeptidase 2 (MMP2), and hydroxyacid oxidase 2 (HAO2). In contrast, pericytes differentially expressed 748 genes, including pyruvate dehydrogenase kinase 4 (PDK4), CD36, and desmin (DES, Fig 32). Along with desmin, we found the pericytes differentially expressed other genes previously associated with pericytes, such as melanoma cell adhesion molecule (MCAM), G protein receptor RGS5, and the potassium channel KCNJ8. We also found pericytes uniquely expressed the serotonin receptor isoform HTR1F, which was distinct from the HTR1B isoform we discovered uniquely expressed by vascular smooth muscle cells that, coincidently, helped us identify the vSMC FACS population [8] (serotonin is a vasoactive peptide [93]). We ranked all expressed genes by their degree of differential expression and used this list for pathway analysis.

We discovered 477 pathways enriched between pericytes and fibroblasts at a false discovery rate of 10%. We found the enrichment of many pathways resulted from small concordant expression differences between the cell types, such as those involved in transcriptional regulation, cell cycle, and immunity. Here, we focus on pathways containing significantly differentially expressed genes.

#### Muscle contraction (6v8)

**Fig 33:**
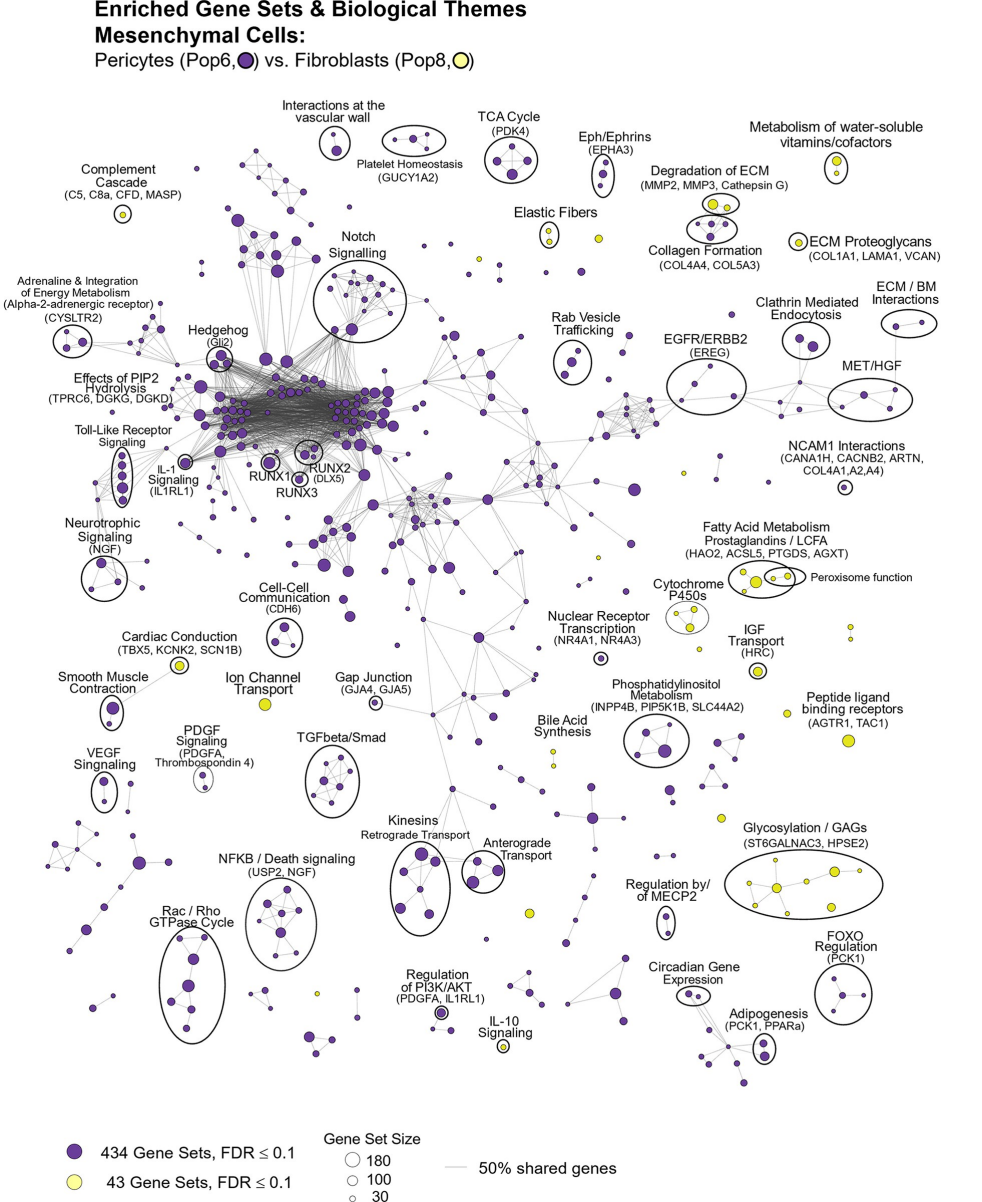
The pericytes and fibroblasts’ enriched pathways. Visualization of pathways enriched among pericytes and fibroblasts (Enrichment Map/Cytoscape). All enriched pathways with an FDR ≤ 0.1 appear on the map (nodes, color-coded by cell type). Pathways (nodes) are connected when they share 50% of their genes (or greater). Node size corresponds to the number of genes in the pathway, as indicated (bottom left). Select biological themes were encircled and annotated. Highly differentially expressed genes are indicated (above the theme when it coincided with the attributed cell type—and below if it was expressed by the contrasted cell type). Samples are color-coded. Due to space constraints and a large number of presented genes, their names (rows of the heatmap) must be inspected digitally (magnified in the PDF). The names of each pathway are also available in the supplemental Cytoscape file and Pathway Analyzer tool (Supplementary data files 11 and 12 at 10.17605/OSF.IO/GFHC3).

As one would predict, we found perivascular pericytes enriched with pathways involved with muscle contraction and smooth muscle contraction (FDR = 0.361% and 0.026%, respectively, Fig 33 and Supplementary Data File 11 at 10.17605/OSF.IO/GFHC3). Pericytes differentially expressed myosin light chain kinase (MYLK), myosin heavy chain 11 (MYH11), actin gamma 2 (ACTG2), and the voltage-gated sodium channel type IV (SCN4A). Also differentially expressed by pericytes was dystrophin (DMD), which anchors ECM proteins to cytoplasmic filamentous actin (and whose defects lead to Duchenne and Becker muscular dystrophies). Tropomodulin 1 (TMOD1) was also highly expressed by pericytes, which is a protein that regulates tropomyosin via blocking the elongation and depolymerization of actin filaments (Fig W, panel A, in S1 Text). Interestingly, cardiac conduction was enriched in fibroblasts (FDR = 5.64%), which differentially expressed T-box 5 (TBX5) transcription factor; potassium channels KCNK1, KCNK2, KCNIP1, and KCNIP4; and the sodium channel SCN1B (Fig 33, Fig W, panel B, in S1 Text). Fibroblasts share similar expression of voltage-gated channels to cardiac myocytes, which are responsible for cardiac conduction in the heart. Additionally, the dramatic difference in gene expression could be due to the low expression of many of these channels in pericytes, which may instead rely on endothelial cells for depolarization signals (as we found similar channels were differentially expressed by vascular endothelial cells when compared to their lymphatic counterparts, Fig 29N).

#### Hemostasis (6v8)

We found 14 pathways associated with hemostasis enriched in pericytes, including platelet homeostasis (FDR = 0.732%, Fig 33). Its enrichment was defined by differential expression of phosphodiesterases 1A, 1B, and 5A (PDE1A, PDE1B, and PDE5A), transient receptor cation channel C6 (TRPC6), cGMP-dependent protein kinase 1 (PRKG1), and the prostaglandin receptor PTGIR (Fig W, panel C, in S1 Text). The phosphodiesterases are responsible for hydrolyzing cGMP and modulating the effects of nitric oxide signaling. Also related to the vasculature was the enrichment, in pericytes, of cell surface interactions at the vascular wall (FDR = 1.14%), resulting from their differential expression of MER proto-oncogene (MER), thrombomodulin (THBD), and angiopoietin 1 (ANGTP1, Fig 33 and Fig W, panel D, in S1 Text).

#### Intracellular transport (6v8)

Pericytes differentially expressed kinesins (KIF4A, KIF2A, and KIF26B) and tubulins (TUBB2A, 3, and 4B), leading to their enrichment of *kinesins* pathway (FDR < 0.001%, Fig 33 and Fig W, panel E, in S1 Text). Clathrin-mediated endocytosis was also enriched in pericytes (FDR < 0.001%), due to their differential expression of the endocytic adapter protein SGIP1, WNT5A ligand, the wnt receptor “frizzled class receptor 4” (FZD4), angiotensin II receptor (AGTR1), and the endocytosis inhibitor synaptotagmin XI (SYT11, Fig 33 and Fig W, panel F, in S1 Text). This pathway regulates the cell surface expression and signaling of RTKs and GPCRs [94,95], and we found that most of the enriched pathways associated with RTKs or GPCRs were enriched in pericytes (Supplementary Data File 12 at 10.17605/OSF.IO/GFHC3).

#### Cell signaling (6v8)

We identified thirty enriched pathways associated with RTK signaling in pericytes, including signaling by NTRKs, signaling by VEGF, and signaling by PDGF (FDR = 0.011%, 0.365%, and 6.67%, respectively, Fig 33). Neurotropic tyrosine kinase receptors are activated by neurotrophins like NGF and NTF3—which were highly and differentially expressed by pericytes (Fig W, panel G, in S1 Text). Pericytes also differentially express AXL receptor tyrosine kinase (AXL), caveolin-1 (CAV1), and integrin beta 3 (ITGB3), which define the enrichment of signaling by VEGF (Fig W, panel H, in S1 Text). Signaling by PDGF was enriched in pericytes due to the differential expression of collagens COL4A1, COL4A2, COL4A4, COL5A3; platelet-derived growth factor alpha (PDGFA), and thrombospondin 4 (THBS4, Fig W, panel I, in S1 Text).

The enrichment of 13 Notch signaling pathways in pericytes indicates their importance to pericyte function. The broader signaling by notch pathway (FDR < 0.001%) was enriched largely because of the differential expression of NOTCH receptors 1 and 3 (NOTCH1, NOTCH3), the downstream transcription factors HEYL and HEY2, and SMAD family member 3 (SMAD3, Fig W, panel S, in S1 Text).

#### Cell–cell communication (6v8)

Communication between pericytes and endothelial cells is critical to vessel development and function. We found pericytes enriched with the broad cell–cell communication and cell junction pathways (FDR = 0.10% and 0.06%, respectively, Fig 33). These pathways involve tight junctions, adherins junctions, gap junctions, and signal regulatory proteins, and pericytes differentially expressed (i) cadherins 3 and 6 (CDH3 and CDH6), classical calcium adhesion molecules; (ii) plectin (PLEC), which interacts and organizes connections to intermediate filaments and microtubules and are essential components of desmosomes and hemidesmosomes; and (iii) laminin subunit alpha-3 (LAMA3), which assembles into laminin heterotrimers that are essential components of the vascular basement membrane. (Fig J, panel A, in S1 Text).

#### Extracellular matrix organization (6v8)

Pericytes and fibroblasts have different functional roles requiring distinct interactions with different ECM components. This was reflected in the different ECM organization pathways we found in either cell type. For example, acting in opposition, the collagen formation pathway was enriched in pericytes, whereas the collagen degradation and degradation of the extracellular matrix pathways were enriched in fibroblasts. Enrichment of the Collagen formation pathway in pericytes (FDR = 4.28%) resulted from their differential expression of COL4A1, COL4A2, COL4A3, COL4A4, COL4A5, COL5A3, COL17A1, COL23A1, COL25A1, and COL27A1 (Fig 33 and Fig W, panel K, in S1 Text). As introduced in the discussion of the collagen family above, the perivascular cells expressed the highest levels of type IV collagens (basement membrane collagens, S14A–S14C Fig in S1 Text), consistent with their spatial location in the tissues—embedded within the vascular basement membranes—and role of maintaining these basement membranes. In contrast, fibroblasts exist in the extracellular matrix, which they are responsible for forming and shaping. We found fibroblasts differentially expressed collagens COL1A1, COL3A1, COL6A6, COL8A1, COL8A2, COL14A1, and COL16A1, contributing to the degradation of the extracellular matrix pathway’s enrichment (FDR = 0.705%, Fig 33 and Fig W, panel L, in S1 Text). Additional differentially expressed genes included matrix metalloproteinases MMP2, MMP3, MMP10, MMP12, MMP13, and MMP16; TIMP metallopeptidase inhibitor 1 (TIMP1); calpains 5 and 6 (CAPN 5 and CAPN6); and the bone remodeling protein cathepsin K—which cleaves collagen I fibers (Fig W, panel L, in S1 Text). We also found enrichment of ECM proteoglycans in fibroblasts (FDR = 4.68%), as they differentially expressed versican (VCAN), asporin (ASPN), lumican (LUM), heparan sulfate proteoglycan 2 (HSPG2), decorin (DCN), and biglycan (BGN) proteoglycans (Fig 33 and S23M). Other genes in this pathway highly expressed by fibroblasts were tenascins N and C (TNN, TNC), TGFB3, and laminin alpha 1 (LAMA1, Fig W, panel M, in S1 Text).

#### Elastic fibers (6v8)

Elastic fibers are essential components of connective tissue and contribute to tissue elasticity. We discovered pathways related to elastic fibers were among the most significantly enriched pathways in fibroblasts, i.e., molecules associated with elastic fibers (FDR = 0.29%) and elastic fiber formation (FDR = 0.90%). Leading to these pathways’ enrichment were the fibroblasts’ robust expression of elastin (ELN), elastic microfibril interfacer 1 (EMILIN1), fibrillins 1 and 2 (FBN1 and FBN2), bone morphogenic protein 7 (BMP7), fibronectin (FN1), and lysyl oxidase 1 (LOXL1, Fig 33 and Fig W, panel N, in S1 Text).

#### Metabolism (6v8)

Pericytes and fibroblasts were enriched with different metabolic pathways. There were 15 pathways enriched in pericytes and 13 in fibroblasts (Supplementary Data File 12 at 10.17605/OSF.IO/GFHC3). One of the most significant pathways enriched in pericytes was Integration of energy metabolism (FDR = 0.016%), stemming from the pericytes’ differential expression of adrenoreceptor alpha 2A and 2C (ADRA2A and ADRA2C), thrombospondin receptor (CD36), adenylate cyclases 6 and 9 (ADCY6 and ADCY9), cysteinyl leukotriene receptor 2 (CYSLTR2), and the voltage-dependent calcium channel CACNB2 (Fig 33 and Fig W, panel O, in S1 Text). In contrast, fibroblasts were enriched in fatty acid metabolism (FDR = 0.889%), owing to their differential expression of prostaglandin synthases PTGES, PTGS2, PTGIS, and PTGDS; the C2 domain-containing phospholipase PLA2G4A; and their unique expression of hydroxyacid oxidase 2 (HAO2, Fig 33 and Fig W, panel O, in S1 Text).

#### White adipocyte differentiation (6v8)

We found 45 of the 84 genes composing the transcriptional regulation of white adipocyte differentiation pathway were elevated in pericytes, leading to its enrichment (FDR = 0.007% Fig 33, Fig W, panel Q, in S1 Text). Genes differentially expressed by pericytes included NR2F2, which regulates apolipoprotein A-I transcription; CD36 (fatty acid translocase), which regulates the transport and uptake of long-chain fatty acids, and fatty acid binding protein-4 (FABP4), which mediates intracellular fatty acid transport (Fig W, panel P, in S1 Text). One of the most notable genes in this pathway is phosphoenolpyruvate carboxykinase 1 (PCK1), which catalyzes the production of phosphoenol pyruvate from oxaloacetate. The activity of PCK1, which is regulated at the transcriptional level, commits mitochondrial pyruvate to gluconeogenesis or glyceroneogenesis [96]. This step is irreversible under physiological conditions and is the rate-limiting step controlling gluconeogenesis flux. We found PCK1 expression in breast cells was limited to the pericytes and adipocytes (Fig W, panel R, in S1 Text), indicating, at the very least, that pericytes have an increased demand for cytoplasmic pyruvate.

### Summary

Resolving the types and properties of cells in a tissue is vital to understanding the underlying symphony of interactions that make life possible. It is essential to overcome diseases such as cancer. Here, we explored the fabric of the breast through a cytometric and molecular dissection of the tissue, which has provided us with insights into its inner workings. From the outset, we had questions regarding the number and types of cells composing the breast, their spatial location, their functions, and how they contributed to the tissue as a whole.

To address these questions, we revisited breast tissues to explore their architecture and cellular composition through an exhaustive cytometric dissection of the tissue, which led to our development of a FACS procedure that reconciled every cell in the tissue (Fig 1). We isolated 12 cell populations from 4 lineages and developed techniques to culture nearly every one (Fig 2) [8]. Our system of defining breast cell types provides a comprehensive resource that can be used to explore interactions in more complex and physiologically relevant culture systems aimed at mimicking the tissue microenvironment.

Here, we explored the nature of each breast cell type by dissecting their transcriptomes. These were acquired by scaling up our FACS procedure and painstakingly sorting each cell type to purity. RNA was then isolated and sequenced, leading to the acquisition of transcriptomes that were of high depth and quality. Although the rare cell types yielded fewer cells and transcripts, concordance of transcriptomic profiles between all cell replicates illustrated the reproducibility of our methods (Fig 4). Analysis of transcripts identified uniquely expressed genes that have already helped us decipher spatially resolved transcripts from breast tumors. Bioinformatic analyses of transcriptional profiles further helped validate the identity of several cell types by providing information on expressed markers and revealing their functional commonalities to related cell types (Fig 6).

Over 20,000 genes were measured in each cell type, providing a wealth of information. As we explored the transcriptomes from every major cell type in the breast, there were so many exciting features that we had to come to terms with the fact that we could not describe all that we encountered. Our solution was to build resource-sharing tools that aid exploration of our data [9,10]. To demonstrate the utility of these resources, we addressed 3 pressing questions: (i) What features distinguish each cell lineage; (ii) what gene types confer cell-specific functions; and (iii) what biological processes define each cell type?

To identify the cell lineages’ distinguishing features, we investigated the cellular pathways impacted by lineage-specific genes, which we defined through PCA. The pathway enrichment analyses affirmed established attributes of each cell lineage while identifying others we had not previously associated with these lineages (Figs 10–14).

Focusing on the genes families that varied most among the different cell types provided us with an unconventional look at the types of genes contributing to the essential qualities of the breast cell types. We examined the expression of each gene, from each gene family, within every breast cell type, revealing the gene types that vary across cell types. We provide a full accounting of each gene family in a supplemental table and have meticulously described 9 gene families whose functions extend from the nucleus (e.g., hormone receptors) to the exterior cell microenvironment (e.g., laminins, Figs 15–19).

To address our last question and develop a broad perspective of each breast cell type’s fundamental properties and individual functions, we performed a comprehensive assessment of the biological pathways and processes that are differentially enriched in each cell population. We performed 55 independent analyses identifying differentially expressed genes and enriched pathways between every possible cell type comparison. These results are available as a downloadable interactive application (Supplementary Data File 12 at 10.17605/OSF.IO/GFHC3 [9]). We comprehensively analyzed 6 comparisons we believed were of the highest interest, as they revealed features of every major cell type and identified those distinguishing closely related cell types (Figs 21–33).

As the field moves forward with single-cell and spatial transcriptomic approaches to explore and define the significance of cellular heterogeneity within tissues and tumors [97–100], we predict that our analyses will complement these studies. Additionally, our FACS method provides the ability to focus on any of the 12 major cell types, allowing them to be purified prior to single-cell sequencing (increasing statistical power and reducing research costs). By FACS-purifying each cell type and sequencing each individually, we achieve a depth of transcripts that provides a strong foundation for interpreting results and identifying features of cell subpopulations that likely exist for each cell type. Our analysis includes samples obtained from 4 women, and further analysis of the hormonal, physiological, and contraceptive effects on gene expression changes in breast cell types will be of significant interest in future studies.

A broad wealth of information touching on many scientific disciplines is presented herein, and we recognize that developing deeper insights requires engagement and a focused exploration of the associated data sets. We offer novel tools to aid this exploration [9,10] and look forward to creating and sharing others. This body of work presents a systematic approach for identifying, isolating, and culturing nearly every major breast cell type of epithelial, endothelial, and mesenchymal origin. We spent many years refining our FACS strategy, used multiple rounds of sorting to ensure purity, exhaustively stained tissues and primary cultures to validate and characterize cell types [8], and comprehensively and painstakingly analyzed each transcriptome. This rich resource, which we submit here, has provided countless insights and has already changed our perception of the cells composing this elaborate and beautiful tissue.

### Experimental procedures

#### Ethics statement

Breast tissues were collected with patient consent and were reported negative for proliferative breast disease by board-certified pathologists. The University of New Mexico Human Research Protections Office granted the use of anonymous samples through exemption status, according to the Code of Federal Regulations 45 CFR 46.101.

#### Tissue acquisition and processing

Breast tissues from reduction mammoplasties were obtained from the Cooperative Human Tissue Network (CHTN), a program funded by the National Cancer Institute. All specimens were collected with patient consent and were reported negative for proliferative breast disease by board-certified pathologists. Upon receipt, several fragments (roughly 2 cm^2^) were embedded in optimal cutting temperature (OCT) compound in tissue cassettes, flash-frozen in nitrogen, and archived at −80°C for later cryosectioning and staining. The remaining tissue, typically 20 to 100 g, was thoroughly rinsed with phosphate-buffered saline and processed to organoids as previously described (gentle agitation method) [101]. Briefly, this included manually mincing and scoring tissues with a scalpel and incubating them overnight (12 to 18 h, 37°C) with 0.1% collagenase I (Gibco/Invitrogen) in Dulbecco’s Modified Eagle Medium containing 100 U/ml penicillin, 100 mg/ml streptomycin, and 100 mg/ml Normocin (Invivogen, San Diego, California, USA). The resulting divested tissue fragments (organoids) were collected by centrifugation (100 g × 2 min) and either archived in liquid nitrogen (90%FBS+10% DMSO) or immediately processed to cell suspensions for FACS analysis. Tissues used for RNA sequencing were immediately processed upon receipt and kept at 4°C during processing and FACS sorting, except for the overnight collagenase digestion at 37°C. RNA-sequenced cells were from fresh specimens and were not previously archived in nitrogen.

#### Antibodies

A list of antibodies and reagents used in this study is provided in Table A in S1 Text. The table includes antibody clone designations, conjugations, isotype, supplier product numbers, and dilution factors used.

#### Cryosectioning and immunostaining

Immunofluorescence was performed on 10 to 80 mm cryosectioned tissue sections, as previously described [8]. Briefly, specimens were fixed with 4% paraformaldehyde for 5 min at 23°C, which was followed by 4% formaldehyde/0.1% saponin for 5 min at 23°C. Tissues were rinsed for 20 min in wash buffer (0.1% saponin/10% goat serum in PBS) and then incubated with primary antibodies, typically overnight at 4°C. Following the primary incubation period, samples were washed and incubated with secondary antibodies conjugated with AlexaFluor 488, 568, 594, or 647 (Thermo). After 1 h incubation at 23°C, samples were rinsed in PBS and their nuclei counterstained with 300 nM DAPI (4′,6-Diamidino-2-Phenylindole, Dihydrochloride, Thermo). Coverslips were mounted with Fluoromount G (Southern Biotech). Images were captured using a Zeiss LSM710 confocal microscope, Zeiss Axioscope, or EVOS FL Auto imaging station. If needed, image contrast was applied to the entire image using Photoshop and was annotated with the antibodies used to stain the tissue.

#### Cell preparation for flow cytometry and FACS analysis

To prepare cell suspensions for FACS and flow cytometry analysis, we thoroughly rinsed the organoids in PBS, then pelleted them via centrifugation at 100 g × 2 min. After removing PBS by aspiration, the organoids were suspended in 1 ml of “Cell Dissociation Reagent” (Sigma# C5914; or Thermo# 13150016), incubated for 2 min at room temperature, upon which 3 ml trypsin (used for RNA-sequenced samples) was added (0.25%, Thermo 25200072; note that we have more recently found TrypLE better preserves CD49f staining, although it dampens CD34 resolution). Samples were incubated by hand/body temperature, thus permitting visual inspection and brief (1 to 3 s) pulse vortexing every 30 to 60 s. After the mixture became cloudy from the dissociating cells (about 8 to 10 min), the organoids/cells were gently and repeatedly pipetted through a 16-gauge needle until clumps dissipated. Afterward, we filtered cell suspensions through a 100 mm cell strainer and added 3 ml 0.1% w/v soybean trypsin inhibitor to stop the digest (Sigma# T9128). The suspensions were then filtered through a 40 mm cell strainer, rinsed in 10 to 20 ml PBS, and pelleted by centrifugation at high centrifugal force (400 g × 5 min—or longer, until all cells had pelleted, which we confirmed by microscopic examination of the supernatant). The cells were then rinsed in 10 ml Hanks balanced salt solution/1% BSA (w/v), counted, and again pelleted by centrifugation. After centrifugation, nearly all the Hanks/BSA was aspirated, leaving the cell pellet with roughly 60 ml of Hanks/BSA. The pellet was resuspended in this small volume, and then we added FACS antibodies to the samples and incubated the samples on ice, covered, for 30 min (see Table A in S1 Text for antibody information). Following the incubation, the cells were rinsed in Hanks/1% BSA, centrifuged (400g × 5 min), and resuspended in Hanks/BSA with To-Pro-3 viability marker (diluted 1:4,000, Thermo T3605), then filtered again through a 40 mm cell strainer cap (into the FACS tube). We did not use DNase enzyme or hypotonic RBC lysis solutions on the cell preparations because we found they were unnecessary. We also wanted to avoid any potential deleterious effect these treatments may have on the cells, such as reducing cell viability or altering gene expression levels. We, again, also conscientiously chilled samples during cell preparation and the sorting procedure for similar concerns.

#### Flow cytometry

The FACS panel and gating strategy outlined herein was developed using a BD FACS Vantage 8-channel cytometer (FACSDIVA software) and has since been adapted and reproduced using a 14-channel SONY SY3200. PMT voltages were optimized on both instruments by analyzing individually stained compensation beads (AbC Anti-Mouse Bead Kit, Thermo # A10344) at increasing voltage intervals for each detector. Because the FACS Vantage has only 8 available channels, it was necessary to use 2 markers (podoplanin and CD45) in a single channel (A488), which we experimentally validated. To-Pro-3 was used as the viability marker, so the corresponding 405 nm channel (455/50) could be used for BV421. This channel was dedicated to CD49f to provide a maximum resolution of cell populations, which is essential to this sorting strategy. The considerable compensation typically required between the heavily overlapping dyes PE/Cy5 and To-Pro-3 was not needed, as the To-Pro-3 negative (viable) cells were used for downstream analyses, allowing us to use the PE/Cy5 channel for Thy1. Negative controls consisted of unlabeled beads and cells incubated with isotype control antibodies conjugated to each fluorophore. Sort times typically ranged 14 to 18 h (50 to 120 million cells stained—needed to yield enough cells for RNA-sequencing). Cells were chilled at the sample intake and collection chambers, and all samples were chilled on ice during the entire experiment. All media and PBS were filtered through a 0.2 mm filters to prevent the FACS machine from triggering events from crystals and debris. Staining profiles of cells collected at the beginning and end of the sorts (sometimes separated by as many as 18 h) were identical and did not show any signal loss or appreciable decrease in viability. Because our machines could sort cells into a maximum of 4 tubes, we first sorted cells into 4 groups, containing 2 to 4 populations each. These mixtures were: Tube1 (CD34Pos) and contained Pops 7, 8, 9; Tube 2 (Thy1^Pos^) contained Pops 3, 6; Tube 3 (Luminal) contained Pops 1&2; and Tube 4 (Null gate) contained Pops 4, 5, 10, 11. During the first round of sorting, cells were typically sorted at approximately 8,000 cell/s. We followed this initial enrichment with purity sorts for each cell type, using stringent sort masks, sorting each population into individual tubes at a rate of approximately 2,000 cells/s. FACS data were analyzed using Flowjo software (version v7.6.3 –v10.8), Tree Star/Becton, Dickinson & Company.

#### RNA isolation and sequencing

Following the overnight digestion of tissues with collagenase, we centrifuged the samples at 100g × 2 min. This produced aqueous and lipid phases that separated under centrifugal force. The top lipid phase was collected, visualized microscopically for cellular content, and then processed immediately for RNA extraction using Qiagen RNA Lipid Tissue Mini Kit (Qiagen 74804), according to the manufacturer’s instructions. Total RNA from the FACS-purified cells was isolated immediately after FACS sorting using Qiagen RNA Mini (>100,000 cells, Qiagen 74104) or RNA Micro kits (<100,000 cells, Qiagen 74004) according to the manufacturer’s instructions. Total RNA was treated with DNAse I for 30 min at 37°C (Ambion DNA-*free* DNA Removal kit, Thermo AM1906*)*, then treated with DNAse Inactivation beads. RNA was then concentrated with RNA Clean & Concentrator-5 columns (Zymo R1015), quantified by Nanodrop and Agilent Bioanalyzer assays, and stored at −80°C.

#### RNA library construction and sequencing

mRNA was captured using poly-T coated dishes (Clontech) and used for library construction using SMARTer technology (SMARTer Stranded RNA-seq Kit, Takara Biotech) following the manufacturer’s guidelines. For low abundance samples, mRNA was captured on biotinylated LNA coated beads, magnetically captured and rinsed 3× in binding and wash buffer (10 mM Tris-HCl (pH 7.5), 1 mM EDTA, 2 M NaCl). mRNA was eluted from Dynabeads in 10 ml volume of elution buffer, following by a 5 min incubation at 68°C. It was then used for first-strand cDNA synthesis (SMARTer Stranded RNA-seq Kit). Sample clean-up and removal of adapter-dimers was performed using Ampure XP beads (Beckman Coulter), according to the manufacturer’s protocol. Specific attention was given to the samples to prevent under- or over-drying the beads, which we found to reduce PCR efficiency. PCR amplification of the RNA-Seq library was performed using SeqAmp DNA Polymerase (Takara Bio, cat# 638509). Each library was then purified prior to sequencing using SPRI AMPure Beads (Beckman Coulter). Sequencing was performed using Illumina Trueseq v.2 technology, targeting approximately 30 million paired-end reads for each cell population (Illumina HiSeq 2 × 100 bp).

#### RNA-Seq preprocessing and quality control assessment

We processed raw count data from aligned fragments (S2 Data) using R programming language (RStudio) and associated BioConductor packages. Quality control analyses (Cook’s distance box plot, covariance matrix, sample correlation matrix) were performed using the DESeq2 package [12]. For data visualization (donut and heatmaps), the count matrix data was transformed by 2 methods used in the DESeq2 package: (i) regularized log transformation (rlog); and (ii) variance-stabilizing transformation (vst), both normalized to library size. Quality control assessment of expressed transcripts across sorted cell samples was conducted, and one sample was removed from statistical analyses as it was an extreme outlier (Epithelial Population 4 Sample N239). This was determined by: (i) distance matrix; (ii) PCA; and (iii) Kolmogorov–Smirnov test that compares each sample to the reference probability distribution of the pooled data. We included this sample in downstream visualizations for reference purposes, for example, the bar and donut graphs used throughout the manuscript, which display rlog transformed values on both log2 (bar graphs) and linear (donut graph) scales.

#### Differential gene expression analysis

All differential expression analyses were performed using DESeq2 [12]. The DESEQ2 likelihood ratio test (LRT) was used to identify genes differentially expressed genes across all cell types (S5 Data). These genes were then used to calculate the proportion of differentially expressed genes within each HGNC families/group in our expression data set (https://www.genenames.org/data/genegroup). Differential expression *p*-values were corrected for multiple hypothesis testing using Benjamini–Hochberg method across all genes and all 55 possible pairwise contrasts. Volcano plots of the DESeq2 contrasted cell types were generated using the EnhancedVolcano R/Bioconductor package [102].

#### Principal component analysis

PCA was performed on VST transformed data (subsetting without tissue and bulk cell controls) using the pcaExplorer R/Bioconductor package [103]. Loading factors from the ends of principal components 1, 2, 3, and 4 were further analyzed using the ClueGo [18] Cytoscape package, referencing the Gene Ontology biological processes (GOBP) and Reactome pathway databases [17,19]. Subsequent enriched pathways were grouped automatically by likeness to obtain a condensed list of defining features for each cell class.

#### Gene set enrichment and pathway analysis

Pathway enrichment analysis was performed using Broad Institute’s GSEA software (version 4.3.2) [50]. We pre-ranked our individual gene lists using the DESeq2 calculated Fold changes and Benjamini–Hochberg adjusted *p*-values from all 55 possible pairwise contrasts (Supplementary Data File 7 at 10.17605/OSF.IO/GFHC3 [56]). The rank metric was calculated by: (sign[Fold Change]* -log_10_{*p*-value}). GSEA analysis was performed using a classic, unweighted enrichment statistic (*p* = 0) [50] and was used to evaluate the enrichment of Reactome pathways [17]. Pathways were obtained via the Molecular Signatures Database [52], and we included pathways with sizes between 15 and 200 genes. The resulting analysis produced a collection of enriched pathways and calculated statistics (1,000 permutations to correct for multiple hypotheses testing) for each of the 55 pairwise comparisons. These data became the foundation for the Excel-based Pathway Analyzer tool (Supplementary Data File 12 at 10.17605/OSF.IO/GFHC3).

#### Enrichment Map/Cytoscape

Visualization of enriched gene sets was performed using the Enrichment Map Cytoscape plugin [50,57], with applied edge cut-offs of 0.5 and FDR cutoffs of 0.1 (10%) for most comparisons, with the exception of the 8v12 comparison between fibroblasts and adipocytes, which had an FDR of 0.2 (20%). The produced nodes (biological pathways) were manually adjusted/grouped into themes for presentation purposes using the Reactome hierarchy as a reference. Annotations of select biological themes and notable genes were added manually.

### Supplemental resources

#### Pathway analyzer (Supplementary Data File 12)

This supplemental tool integrates regularized log transformed transcript levels (mRNA-sequencing values) and enriched biological pathways identified by GSEA. Data from every possible pairwise comparison of cell types is embedded in the tool, which is supplied as an MS Excel file. The file is approximately 300 Mb and contains computationally intensive algorithms (e.g., we run the file on both Windows and MAC-based computers (Surface Laptop Studio [Core i7/3.3 GHz with 32 Gb RAM], Surface Book 2 [Corei7/4.2 GHz with 16 Gb RAM], Dell Inspiron Desktop [Corei5/3.2 GHz with 8 Gb RAM], MacBook Pro [Corei7/2.1 GHz with 16 Gb RAM]), and we recommend using it on computers with ≥16 Gb RAM). This file can be found at 10.17605/OSF.IO/GFHC3.

#### Breast Cell Atlas: Transcript Finder

http://breastcancerlab.com/breast-atlas/. This online tool facilitates exploration of mRNAs expressed by the breast cells. When provided individual gene names (official gene symbol), the tool will display the transcript levels measured in each cell type. Regularized log-transformed values are provided on a log2 and linear scale (bar and donut graph, respectively), similar to those presented in the many figure elements contained herein. This application was coded using the R/Shiny package [104].

## Supporting information

S1 Text **Fig A. FACS strategy designed for an 8 channel FACS machine (BD FACS Vantage)**. **(A)** FACS gating scheme used to isolate the RNA-sequenced cell types (antibody panel designed for an 8-channel FACS machine- BD FACS Vantage; FCS files are available at https://osf.io/gfhc3/). Representative FACS data are of cells derived from disease-free reduction mammoplasty tissue surgically resected from a 22-year-old female (sample #N239). **Fig B. (A)** High-resolution image of the immunostained breast tissues provided in Fig 2A. **(B)** Magnified area indicated by white box in a (on left) containing several breast lobules (a.k.a, terminal ductal lobular units). **(C)** Magnified area indicated by white box in a (on right) containing the cross section of a large lactiferous duct. The duct is surrounded by numerous blood vessels running parallel to the duct and is embedded in a collagenous-rich matrix. **Fig C. (A, B)** High-resolution (label-free) images of the immunostained breast tissues provided in Fig 2C and 2D. **Fig D. (A, B)** High-resolution (label-free) images of the immunostained breast tissues provided in Fig 2E and 2F. **Fig E. Project workflow.** Diagram of the sequential processes and analyses in the body of work dissecting breast tissues and defining each cell type. In a previous article (in green), we describe the development and validation of a FACS isolation strategy for separating all 12 major breast cell types, culminating with the development of primary cell models for 9 major breast cell types. The current article (in blue) explores the nature of cell type through bulk-mRNA-sequencing and analysis of transcript levels measured in each FACS-purified population. **Table A. Antibodies.** Antibody clones used for immunostaining and FACS. **Fig F. mRNA levels correspond to FACS staining. (A)** Normalized mRNA values for genes encoding FACS markers used to purify cell types from tissues (and CD36, which was later used to purify cultured adipocytes). Transcript levels are provided on (a) a log2 scale (bar graphs containing each biological replicate) and (b) a linear scale (donut graphs of the replicate’s median value), which are both color-coded by cell type. **(B)** Schematic indicating relative FACS staining levels of each marker in the 12 breast cell types. “Neg” indicates relative negative staining (i.e., compared to other populations, not absolute negativity), “-/+” indicates near background level staining, and the increasing number of “+” indicates greater staining intensity. ^**†**^ CD36 is a marker used to purify cultured adipocytes but was not used to purify adipocytes prior to RNA-sequencing in this analysis. **(C)** Normalized transcript levels for the commonly used internal control gene GAPDH (rlog, DEseq2) are presented on a log2 scale (bar graph) and linear scale (donut graph of median value) (data values for donut graphs can be found in Supplementary Data File 13). **Fig G. CD antigen genes.** Median mRNA expression levels of genes encoding CD antigens (rlog, log2 scale; Supplementary Data File 13). The genes are arranged by the cell type that expressed them at their highest level, as indicated by the background shading specific to each cell type. Genes differentially expressed across cell populations are annotated with (*). CD antigen genes in the uncolored panel were not expressed by any breast cell type. **Fig H. Variance captured by principal components.** A scree plot of sample variances captured by each principal component, and 71% of the total variance is captured by the first 4 components (Supplementary Data File 13). **Fig I. PCA with endothelial cells removed. (A)** PCA projection of breast cell types (after removal of the lymphatic and vascular endothelial cells; S3 Data) resolves the mesenchymal cell types (on PC1^NoEndo^). **(B)** Median mRNA expression of the 50 loading factors (genes) contributing to the positive end of PC2 (top end of the list; rlog, log2 scale; Supplementary Data File 13). Stars (★) denote common markers of pericytes (purple) and fibroblasts (yellow). **Fig J. Pathway analysis of leukocyte genes (PC2). (A)** Grouped GO/Reactome terms associated with the 200 genes contributing to the positive end (bottom of list) of PC1 ordered by adjusted *p*-value. Median mRNA expression (rlog transformed) for PC2 genes associated with select pathways, including **(B)** adaptive immune response, **(C)** immune response activation, and **(D)** leukocyte homeostasis (data values for line graphs are found in Supplementary Data File 13). **Fig K. Transcript levels of genes composing the signaling by *SCF-KIT* pathway.** Transcript levels for each gene in the Reactome pathway signaling by *SCF-KIT*. VST gene levels are relative (normalized within each row of the heatmap; Supplementary Data File 13). Boxed areas highlight expression of select genes in lymphatic and vascular endothelial, and ER^Neg^ luminal epithelial cell types. **Fig L. Transcript levels of genes composing the *Semaphorins* gene family. (A)** Transcript levels of genes in Semaphorin gene family. VST gene levels are relative (normalized within each row of the heatmap; Supplementary Data File 13). Boxed areas highlight select genes highly expressed by the lymphatic and vascular endothelial cell types. Normalized mRNA values of **(B)** NRP1 and **(C)** NRP2 (rlog, DEseq2) are provided on log2 scale (bar graph of each biological replicate) and linear scale (donut graph of median value), which are both color-coded by cell type (data values for donuts are found in Supplementary Data File 13). **Table B. Variably expressed gene types.** Genes composing each gene family (HGNC) were assessed by DESeq2 (Adj.*p*-value ≤ 1, across all cell types). The number of genes in each family, along with the number of those found to be differentially expressed, is provided (for gene families ≥ 6 genes). **Fig M. Distribution of gene family size.** The sizes of the HGNC families range from 1 to 831 genes. The distribution of these family sizes is provided, along with a box and whisker plot (top). The median family size is 6 genes per family (Supplementary Data File 13). **Fig N. Transcript levels of DE genes.** Transcript levels for select Type IV collagen chains: (**A)** COL4A1, **(B)** COL4A2, and **(C)** COL4A4. **(D)** Median mRNA expression (rlog, log2 scale) for integrin alpha subunits. The dashed circles identify notable differentially expressed genes, ITGA6, ITGAE, and ITGAM. **(E)** Transcript levels of the laminin-binding integrins. The predominant laminin-binding integrins are αβ heterodimers of: α3β1, α7β1, α7β1, and α6β4. These are integrins are encoded by ITGB1, ITGA3, ITGA7, ITGA6, and ITGB4. Collagen-binding integrins that also show laminin binding properties include heterodimers of: α1β1, α2β1, and α10β1 (encoded by ITGA1, ITGA2, ITGA10, and ITGB1). ITGA7 is predominantly limited to the perivascular cell types, whereas ITGA3, ITGA6, and ITGB4 are largely expressed by the epithelial cell types. (All data values are found in Supplementary Data File 13.) **Fig O. IHC staining. (A)** Claudin-1 staining of normal breast tissue (reduction mammoplasty of 28-year-old female). (θ) Claudin-1 staining is found confined to the apical surface of the paracellular space of luminal epithelial cells, circumscribing the luminal surface of the cells. Scale = 50 μm. **Fig P. Transcript levels from enriched pathways. (A–T)** Transcript levels of leading-edge genes from select pathways enriched in ER^Pos^ or ER^Neg^ luminal cells (from 325 enriched pathways with an FDR of ≤10%, GSEA; Supplementary Data File 13). VST gene levels are relative (normalized by row). Arrowheads under the heatmaps identify the cell type either over- or under-expressing the pathway’s genes. Samples are color-coded. Due to space constraints and the large number of presented genes, their names (rows of the heatmap) must be inspected digitally (magnified in the PDF). **Fig Q. Transcript levels from enriched pathways. (A–E)** Transcript levels of leading-edge genes from select pathways enriched in ER^Pos^ or ER^Neg^ luminal cells (from 325 enriched pathways with an FDR of ≤10%, GSEA; Supplementary Data File 13). VST gene levels are relative (normalized by row). Arrowheads under the heatmaps identify the cell type either over- or under-expressing the pathway’s genes. Samples are color-coded. Due to space constraints and the large number of presented genes, their names (rows of the heatmap) must be inspected digitally (magnified in the PDF). **Fig R. Transcript levels of toll-like receptors. (A)** Transcript levels of TLR gene family. **(B–F)** Normalized mRNA values (rlog, DEseq2) provided on log2 scale (bar graph of each biological replicate) and linear scale (donut graph of median value), which are both color-coded by cell type. (All data values are found in Supplementary Data File 13.) **Fig S. Transcript levels of milk proteins. (A)** Transcript levels of milk proteins. VST gene values are relative (normalized by row; Supplementary Data File 13). **(B–J)** Normalized mRNA values (rlog, DEseq2) provided on log2 scale (bar graph of each biological replicate) and linear scale (donut graph of median value; Supplementary Data File 13), which are both color-coded by cell type. **Fig T. Transcript levels from enriched pathways**. **(A–M)** Transcript levels of leading-edge genes from select pathways enriched in ER^Pos^ or myoepithelial cells (from 426 enriched pathways with an FDR of ≤10%, GSEA; Supplementary Data File 13). VST gene levels are relative (normalized by row). Arrowheads under the heatmaps identify the cell type, either over- or under-expressing the pathway’s genes. Samples are color-coded. Due to space constraints and the large number of presented genes, their names (rows of the heatmap) must be inspected digitally (magnified in the PDF). **Fig U. Transcript levels of mucins and TCA cycle genes. (A)** Transcript levels of mucins. VST gene values are relative (normalized by row; Supplementary Data File 13). **(B–E)** Normalized mRNA values (rlog, DEseq2) provided on log2 scale (bar graph of each biological replicate) and linear scale (donut graph of median value; Supplementary Data File 13), which are both color-coded by cell type. **(F)** Transcript levels of TCA cycle genes. VST gene values are relative (normalized by row; Supplementary Data File 13). **Fig V. Transcript levels from enriched pathways. (A–T)** Transcript levels of leading-edge genes from select pathways enriched in ER^Pos^ luminal and vascular endothelial cells (from 500 enriched pathways with an FDR of ≤10%, GSEA; Supplementary Data File 13). VST gene levels are relative (normalized by row). Arrowheads under the heatmaps identify the cell type, either over- or under-expressing the pathway’s genes. **Fig W. Transcript levels from enriched pathways. (A–S)** Transcript levels of leading-edge genes from select pathways enriched in pericytes and fibroblasts (from 477 enriched pathways with an FDR of ≤10%, GSEA; Supplementary Data File 13). VST gene levels are relative (normalized by row). Arrowheads under the heatmaps identify the cell type, either over- or under-expressing the pathway’s genes.
(PDF)

S1 Data Sample information/Meta Data. Table of sample information, containing age/sex/race information supplied with the acquired tissues (B = black; W = white), Patient number, FACS gate, cell type, cell yields, RNA-isolation strategy/kit, elution volumes, nanodrop concentrations, DNase treatment used, Bioanalyzer results (concentration and integrity number), RNA-sequencing batch, and calculated reads in each sample.
(XLSX)

S2 Data RNA-sequencing count matrix. (XLSX)

S3 Data PCA loading factors. Sheet 1, “All Pops,” contains loading factors calculated from the analysis of all cell types (Pops 1–12). Sheet 2, “Endothelial Removed,” contains loading factors calculated from the analysis of non-endothelial cell types (Pops 1–7, 8, 10–12).
(XLSX)

S4 Data Tabulated pathway enrichment results (ClueGo/Reactome/Gene Ontology Biological Processes) that used the 200 loading factors associated with the 4 cell lineages: (a) Epithelial end of PC1, (b) Leukocyte end of PC2, (c) Mesenchymal end of PC1^No Endo^, (d) Endothelial end of PC3, and the perivascular and myoepithelial lineage subtypes (e) Perivascular cell end of PC3, and (f) myoepithelial end of PC4.
(XLSX)

S5 Data RNA-Seq DESeq2 likelihood ratio test (LRT) statistics, *p*-values, and Benjamini–Hochberg adjusted *p-*values for differentially expressed genes with LRT adj.*p-*val < 0.05. These results were used to calculate the proportion of differentially expressed genes in each HGNC gene family.
(XLSX)

S6 Data Table of the 1,421 HGNC Gene families, number of genes in each family, and the calculated number of differentially expressed genes in each family (using the DESeq2 Benjamini–Hochberg Adj. *p*-value threshold of ≤0.1). (XLSX)

## Abbreviations

AU: approximately unbiased
CHTN: Cooperative Human Tissue Network
DE: differentially expressed
DSP: desmosome-associated desmoplakin
ECM: extracellular matrix
EGF: epidermal growth factor
EGFR: epidermal growth factor receptor
EPCAM: epithelial cell adhesion molecule
FABP: fatty acid-binding protein
FACS: fluorescence-activated cell sorting
FDR: false discovery rate
GFAP: glial fibrillary acidic protein
GO: Gene Ontology
OBP: Gene Ontology biological processes
GSEA: gene set enrichment analysis
HGF: hepatocyte growth factor
LPS: lipopolysaccharide
LRT: likelihood ratio test
MMP: matrix metalloproteinase
OCT: optimal cutting temperature
PC: principal component
PCA: principal component analysis
PDGFRB: platelet-derived growth factor receptor beta
PGF: placental growth factor
RTK: receptor tyrosine kinase
TRP: transient receptor potential
VDR: vitamin D receptor
vSMC: vascular smooth muscle cell
VWF: von Willebrand factor
