# Supplementary material for: Transcriptomic analysis of the 12 major human breast cell types reveals mechanisms of cell and tissue function
Source: PLoS Biol. 2024 Nov 5;22(11):e3002820. doi: 10.1371/journal.pbio.3002820 (PMC11537416; doi:10.1371/journal.pbio.3002820)
Supplement: S1 Text — Fig A. FACS strategy designed for an 8 channel FACS machine (BD FACS Vantage). (A) FACS gating scheme used to isolate the RNA-sequenced cell types (antibody panel designed for an 8-channel FACS machine- BD FACS Vantage; FCS files are available at https://osf.io/gfhc3/). Representative FACS data are of cells derived from disease-free reduction mammoplasty tissue surgically resected from a 22-year-old female (sample #N239). Fig B. (A) High-resolution image of the immunostained breast tissues provided in Fig 2A. (B) Magnified area indicated by white box in a (on left) containing several breast lobules (a.k.a, terminal ductal lobular units). (C) Magnified area indicated by white box in a (on right) containing the cross section of a large lactiferous duct. The duct is surrounded by numerous blood vessels running parallel to the duct and is embedded in a collagenous-rich matrix. Fig C. (A, B) High-resolution (label-free) images of the immunostained breast tissues provided in Fig 2C and 2D. Fig D. (A, B) High-resolution (label-free) images of the immunostained breast tissues provided in Fig 2E and 2F. Fig E. Project workflow. Diagram of the sequential processes and analyses in the body of work dissecting breast tissues and defining each cell type. In a previous article (in green), we describe the development and validation of a FACS isolation strategy for separating all 12 major breast cell types, culminating with the development of primary cell models for 9 major breast cell types. The current article (in blue) explores the nature of cell type through bulk-mRNA-sequencing and analysis of transcript levels measured in each FACS-purified population. Table A. Antibodies. Antibody clones used for immunostaining and FACS. Fig F. mRNA levels correspond to FACS staining. (A) Normalized mRNA values for genes encoding FACS markers used to purify cell types from tissues (and CD36, which was later used to purify cultured adipocytes). Transcript levels are provided on (a) a log2 scal [file pbio.3002820.s001.pdf]

# Transcriptomic analysis of the twelve major human breast cell types reveals mechanisms of cell and tissue function

Katelyn Del Toro, Rosalyn Sayaman, Kate Thi, Yamhilette Licon-Munoz, and William C. Hines

# Supporting Information

# Supporting Information

| | |
|---|---|
| Figure S1 | FACS strategy designed for an 8 channel FACS machine |
| Figure S2 | High Res. images of immunstaining from 2a |
| Figure S3 | High Res. images of immunstaining from 2c,d |
| Figure S4 | High Res. images of immunstaining from 2e,f |
| Figure S5 | Project Workflow |
| S1 Data* | Sample Treatment |
| S2 Data* | Count Matrix (w/ rlog and VST) |
| S3 Data* | PCA Loading Factors |
| S4 Data* | Tabulated Pathway Enrichment Results |
| S5 Data* | DESeq2 Likelihood Ratio Test Statistics |
| S6 Data* | Gene Families |
| S7 Data* | DESeq2 of 55 pairwise comparisons |
| S8 Data* | Enrichment Map Pop1v2 ($ER^{Pos}$ luminal cells vs. $ER^{Neg}$ luminal cells) |
| S9 Data* | Enrichment Map Pop2v3 ($ER^{Neg}$ luminal cells vs. myoepithelial cells) |
| S10 Data* | Enrichment Map Pop2v9 ($ER^{Neg}$ luminal cells vs. vascular endothelial cells) |
| S11 Data* | Enrichment Map Pop6v8 (pericytes vs. fibroblasts) |
| S12 Data* | Pathway Analyzer (Excel based application for analyzing all cell type comparisons) |
| S13 Data* | Comprehensive file containing the underlying data values for all graphs and figures |
| Table S1 | Antibodies |
| Figure S6 | mRNA levels correspond to FACS markers |
| Figure S7 | CD antigen genes |
| Figure S8 | Variance captured by principal components |
| Figure S9 | PCA with endothelial cells removed |
| Figure S10 | Pathway analysis of leukocyte genes |
| Figure S11 | Transcript levels of genes composing the signaling by SCF-KIT pathway |
| Figure S12 | Transcript levels of genes composing the Semaphorins gene family |
| Table S2 | Variably expressed gene types |
| Figure S13 | Distribution of gene family size |
| Figure S14 | Transcript levels of DE genes |
| Figure S15 | IHC staining |
| Figure S16 | Transcript levels from enriched pathways (1v2) |
| Figure S17 | Transcript levels from enriched pathways (1v2) |
| Figure S18 | Transcript levels of toll-like receptors |
| Figure S19 | Transcript levels of milk proteins |
| Figure S20 | Transcript levels from enriched pathways (2v3) |
| Figure S21 | Trancsript levels of mucins and TCA cycle genes |
| Figure S22 | Transcript levels from enriched pathways (2v9) |
| Figure S23 | Transcript levels from enriched pathways (6v8) |

*located online (Open Science Framework Data Repository: https://osf.io/gfhc3/ )

Figure S1

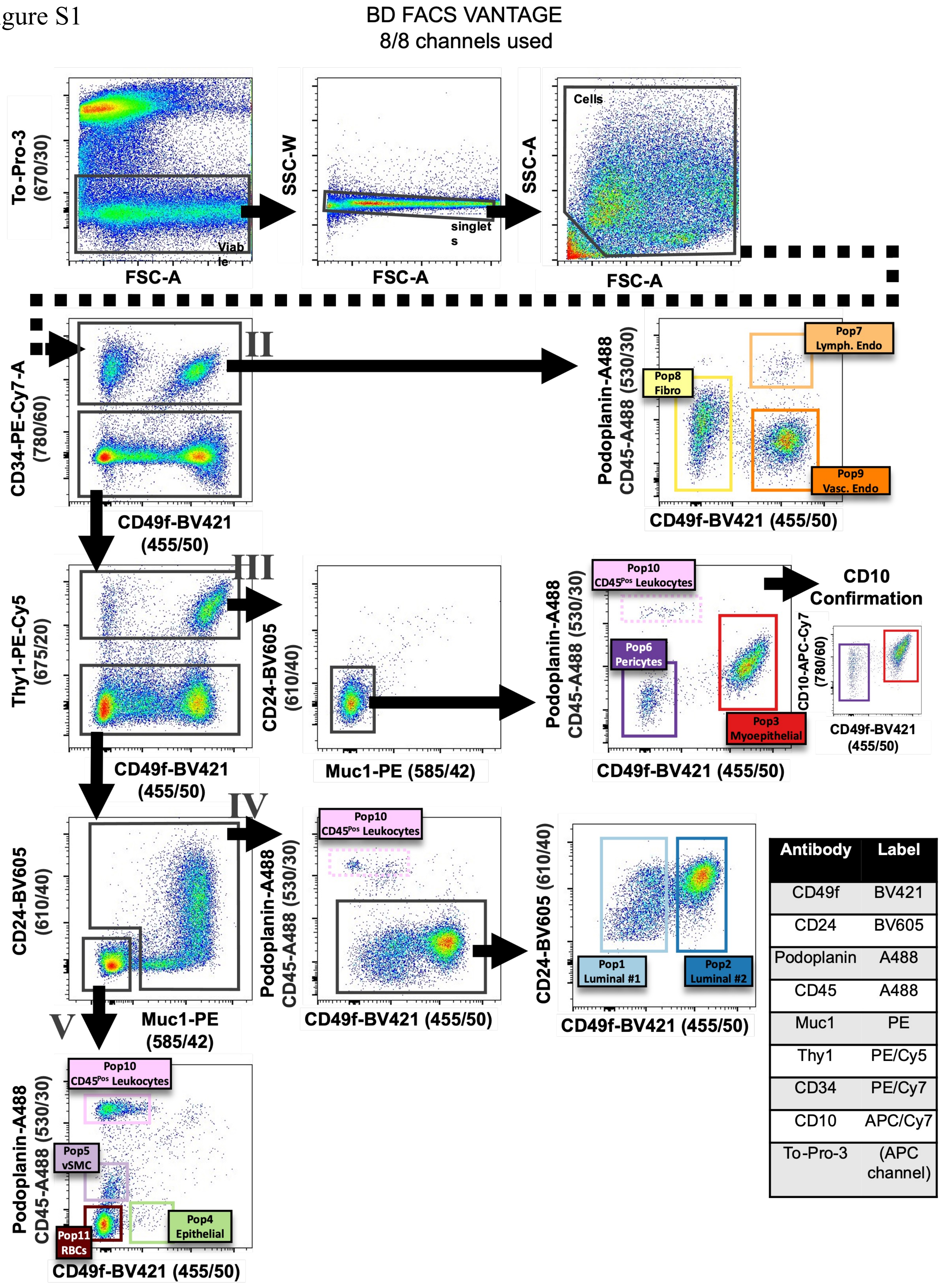


| Antibody | Label |
|---|---|
| CD49f | BV421 |
| CD24 | BV605 |
| Podoplanin | A488 |
| CD45 | A488 |
| Muc1 | PE |
| Thy1 | PE/Cy5 |
| CD34 | PE/Cy7 |
| CD10 | APC/Cy7 |
| To-Pro-3 | (APC channel) |

**Figure S1. FACS strategy designed for an 8 channel FACS machine (BD FACS Vantage). A,** FACS gating scheme used to isolate the RNA-sequenced cell types (antibody panel designed for an 8-channel FACS machine- BD FACS Vantage; FCS files are available at https://osf.io/gfhc3/). Representative FACS data are of cells derived from disease-free reduction mammoplasty tissue surgically resected from a 22-year-old female (sample #N239).

Figure S2

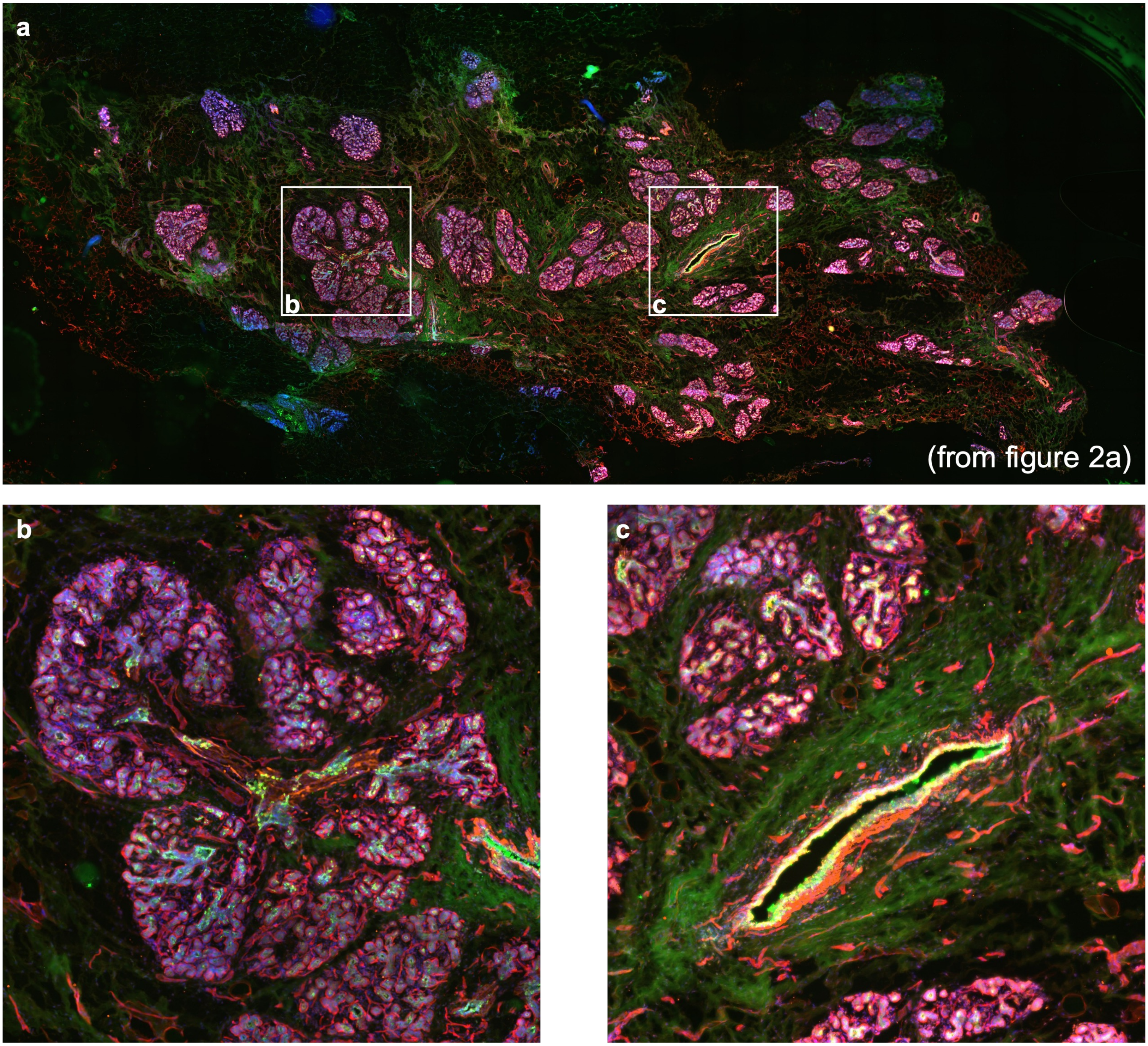


**Figure S2. A,** High-resolution image of the immunostained breast tissues provided in Fig. 2a. **B,** magnified area indicated by white box in a (on left) containing several breast lobules (a.k.a, terminal ductal lobular units). **C,** magnified area indicated by white box in a (on right) containing the cross section of a large lactiferous duct. The duct is surrounded by numerous blood vessels running parallel to the duct and is embedded in a collagenous-rich matrix.

Figure S3

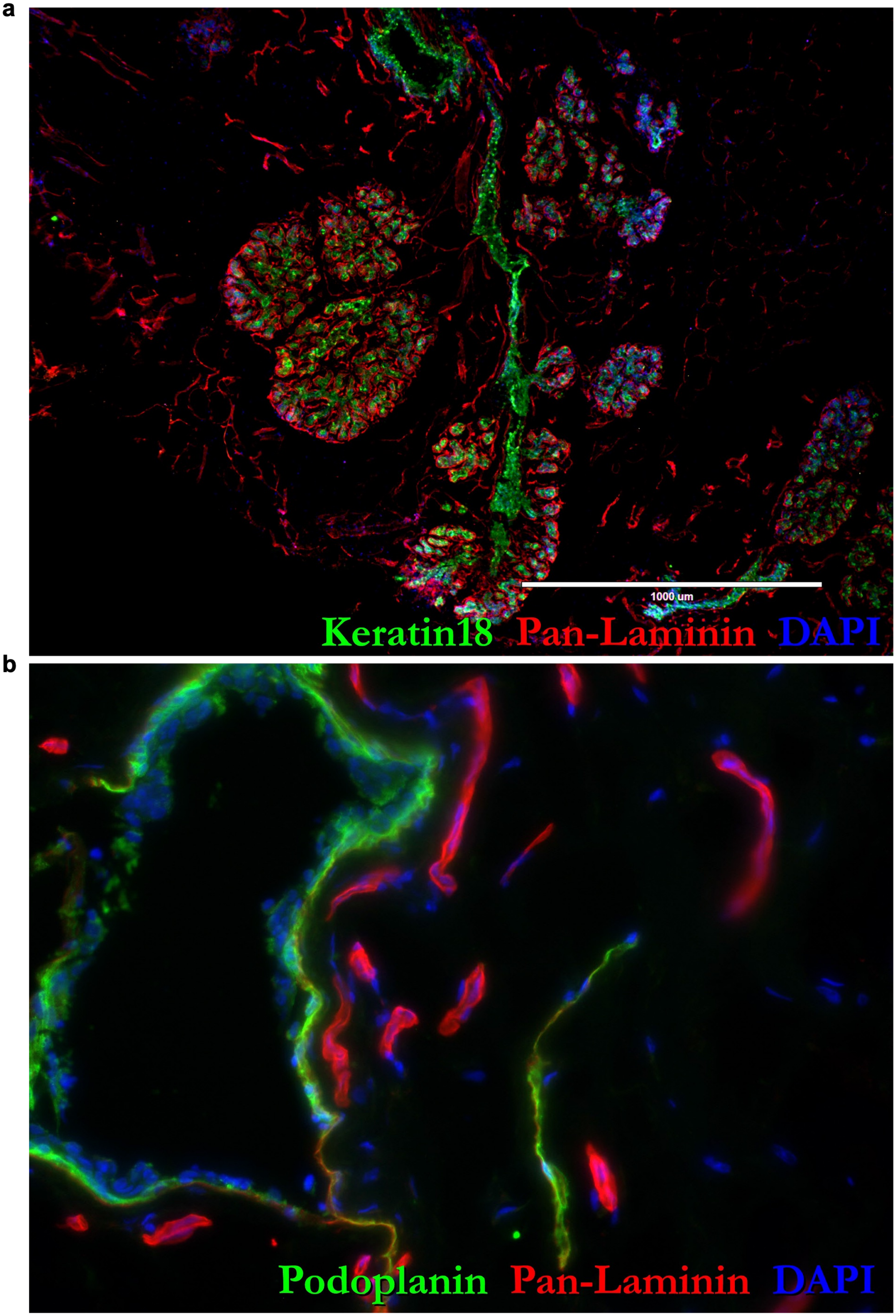


**Figure S3. A&B,** High-resolution (label-free) images of the immunostained breast tissues provided in Figs 2C,D.

Figure S4

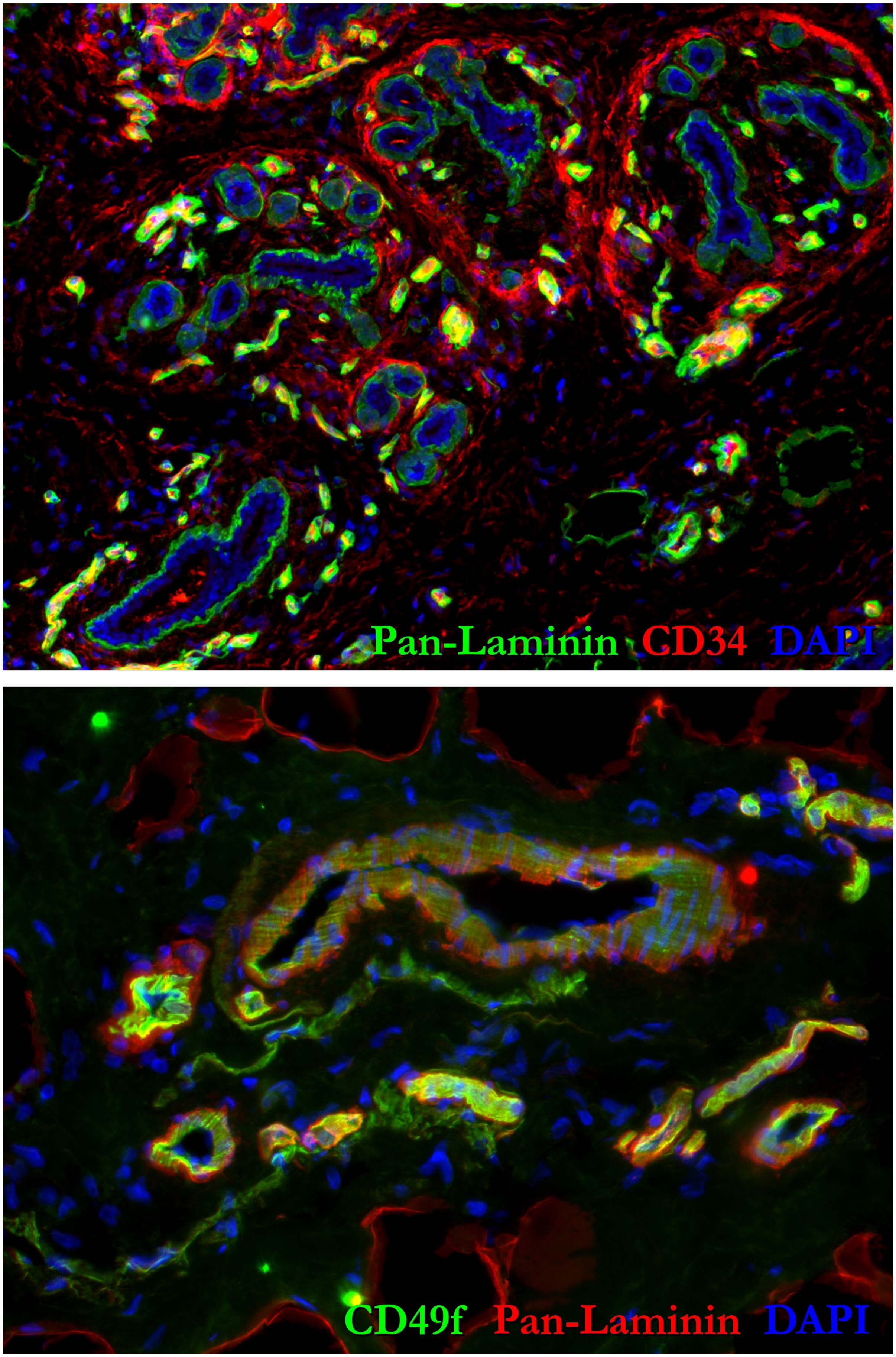


**Figure S4. A&B,** High-resolution (label-free) images of the immunostained breast tissues provided in Figs 2E,F.

# Figure S5

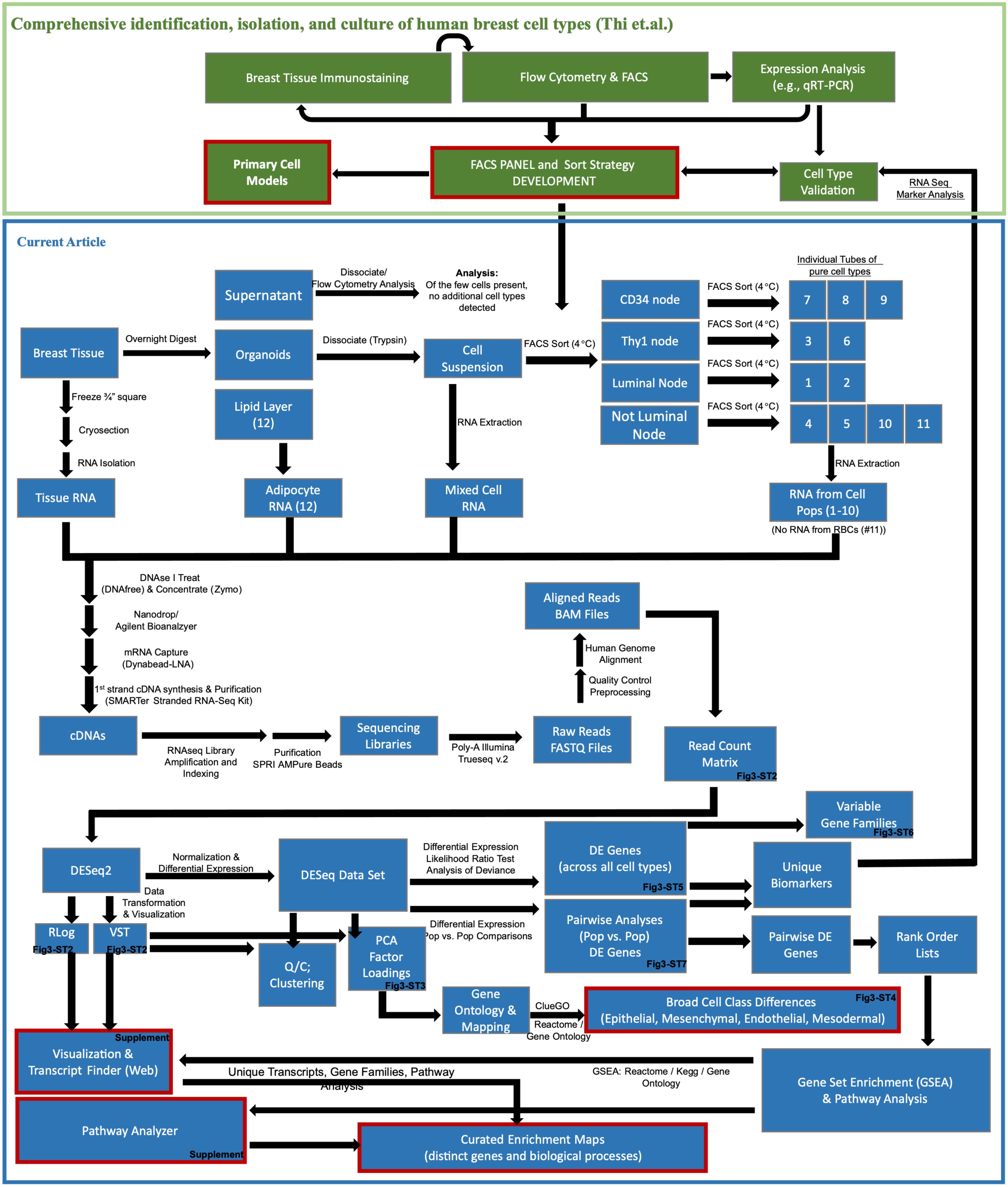

Comprehensive identification, isolation, and culture of human breast cell types (Thi et.al.)
Breast Tissue Immunostaining
Flow Cytometry & FACS
Expression Analysis (e.g., qRT-PCR)
Primary Cell Models
FACS PANEL and Sort Strategy DEVELOPMENT
Cell Type Validation
RNA Seq Marker Analysis
Current Article
Supernatant
Dissociate/ Flow Cytometry Analysis
Analysis: Of the few cells present, no additional cell types detected
Individual Tubes of pure cell types
CD34 node
FACS Sort (4 °C)
7
8
9
Breast Tissue
Overnight Digest
Organoids
Dissociate (Trypsin)
Cell Suspension
FACS Sort (4 °C)
Thy1 node
3
6
Luminal Node
1
2
Freeze ¾" square
Lipid Layer (12)
Not Luminal Node
4
5
10
11
Cryosection
RNA Extraction
RNA Isolation
Tissue RNA
Adipocyte RNA (12)
Mixed Cell RNA
RNA from Cell Pops (1-10)
(No RNA from RBCs (#11))
DNAse I Treat (DNAfree) & Concentrate (Zymo)
Nanodrop/ Agilent Bioanalzyer
mRNA Capture (Dynabead-LNA)
1st strand cDNA synthesis & Purification (SMARTer Stranded RNA-Seq Kit)
Aligned Reads BAM Files
Human Genome Alignment
Quality Control Preprocessing
cDNAs
RNAseq Library Amplification and Indexing
Purification SPRI AMPure Beads
Sequencing Libraries
Poly-A Illumina Trueseq v.2
Raw Reads FASTQ Files
Read Count Matrix
Fig3-ST2
Variable Gene Families
Fig3-ST6
DESeq2
Normalization & Differential Expression
DESeq Data Set
Differential Expression Likelihood Ratio Test Analysis of Deviance
DE Genes (across all cell types)
Fig3-ST5
Unique Biomarkers
Data Transformation & Visualization
RLog
Fig3-ST2
VST
Fig3-ST2
PCA Factor Loadings
Fig3-ST3
Differential Expression Pop vs. Pop Comparisons
Pairwise Analyses (Pop vs. Pop) DE Genes
Fig3-ST7
Pairwise DE Genes
Rank Order Lists
Q/C; Clustering
Gene Ontology & Mapping
ClueGO
Reactome / Gene Ontology
Broad Cell Class Differences (Epithelial, Mesenchymal, Endothelial, Mesodermal)
Fig3-ST4
Supplement
Visualization & Transcript Finder (Web)
Unique Transcripts, Gene Families, Pathway Analysis
GSEA: Reactome / Kegg / Gene Ontology
Gene Set Enrichment (GSEA) & Pathway Analysis
Pathway Analyzer
Supplement
Curated Enrichment Maps (distinct genes and biological processes)

**Figure S5. Project workflow.** Diagram of the sequential processes and analyses in the body of work dissecting breast tissues and defining each cell type. In a previous article (in green), we describe the development and validation of a FACS isolation strategy for separating all twelve major breast cell types, culminating with the development of primary cell models for nine major breast cell types. The current article (in blue) explores the nature of cell type through bulk-mRNA-sequencing and analysis of transcript levels measured in each FACS-purified population.

**(separate file upload)**

**S1 Data. Sample information/Meta Data.** Table of sample information, containing age/sex/race information supplied with the acquired tissues (B=black; W=white), Patient number, FACS gate, cell type, cell yields, RNA-isolation strategy/kit, elution volumes, nanodrop concentrations, DNase treatment used, Bioanalyzer results (concentration and integrity number), RNA-sequencing batch, and calculated reads in each sample.

**(separate file upload)**

**S2 Data.** RNA-sequencing count matrix.

**(separate file upload)**

**S3 Data.** PCA loading factors. Sheet 1, ‘All Pops,’ contains loading factors calculated from the analysis of all cell types (Pops 1-12). Sheet 2, ‘Endothelial Removed,’ contains loading factors calculated from the analysis of non-endothelial cell types (Pops 1-7,8,10-12).

**(separate file upload)**

**S4 Data.** Tabulated Pathway Enrichment Results (ClueGo/Reactome/Gene Ontology Biological Processes) that used the 200 loading factors associated with the four cell lineages: a) Epithelial end of PC1, b) Leukocyte end of PC2, c) Mesenchymal end of $PC1^{No\ Endo}$, d) Endothelial end of PC3, and the perivascular and myoepithelial lineage subtypes e) Perivascular cell end of PC3, and f) myoepithelial end of PC4.

**(separate file upload)**

**S5 Data.** RNA-Seq DESeq2 Likelihood Ratio Test (LRT) statistics, p-values, and Benjamini Hochberg adjusted *p*-values for differentially expressed genes with LRT adj.*p*-val<0.05. These results were used to calculate the proportion of differentially expressed genes in each HGNC gene family.

**(separate file upload)**

**S6 Data.** Table of the 1,421 HGNC Gene families, number of genes in each family, and the calculated number of differentially expressed genes in each family (using the DESeq2 Benjamini Hochberg Adj. *p*-value threshold of $\leq 0.1$).

**(separate file upload)**

**S7 Data.** Excel workbook containing 55 DESeq results (on separate tabulated worksheets; https://osf.io/gfhc3/) from each possible pairwise contrast of the 11 seqenced breast cell types; e.g. Pop1v2 tab contains results from the contrast of Pop1 ($ER^{Pos}$ luminal epithelial cells) and Pop2 ($ER^{Neg}$ luminal epithelial cells). Tab 1v2 also contains the calculated rank metric used for GSEA analysis; i.e., $sign([Fold\ Change] * -log_{10} \{p\text{-}value\})$.

**(separate file upload)**

**S8 Data.** Cytoscape Enrichment Map for $ER^{Pos}$ luminal cells vs. $ER^{Neg}$ luminal cells (1v2)

**(separate file upload)**

**S9 Data.** Cytoscape Enrichment Map for $ER^{Neg}$ luminal cells vs. myoepithelial cells (2v3)

**(separate file upload)**

**S10 Data.** Cytoscape Enrichment Map for $ER^{Neg}$ luminal cells vs. vascular endothelial cells (2v9)

**(separate file upload)**

**S11 Data.** Cytoscape Enrichment Map for pericytes vs. fibroblasts (6v8)

**(separate file upload)**

**S12 Data.** Pathway Analyzer: Excel-based application for analyzing GSEA results for all cell type comparisons

**(separate file upload)**

**S13 Data.** Excel file containing all underlying data for all graphs and figures.

Table S1

| Tissue Staining (Immunofluorescence) | | | | |
|---|---|---|---|---|
| **Antibody specificity** | Clone | Conjugated | Source | Dilution |
| **Alpha-Smooth Muscle Actin** | 1A4 | No | Mouse IgG2a, κ, Lab Vision/Neomarkers MS-113-P0[‖] | 1:100 |
| **CD29** | TS2/16 | A488 | Mouse IgG1, κ; Biolegend 303015 | ½ assay[¶] |
| **CD49f** | GoH3 | No | Rat IgG2a, κ; Biolegend 313602 | ½ assay[¶] |
| **E-Cadherin (CD324)** | 24E10 | No | Rabbit mAb; Cell Signaling 3195S | 1:100 |
| **E-Cadherin (CD324)** | 67A4 | A488 | Mouse IgG1, κ; BioLegend 324110 | ½ assay[¶] |
| **erbB2/Her2 (CD340)** | 24D2 | PE | Mouse IgG1, κ; Biolegend 324406 | ½ assay[¶] |
| **Keratin 14** | Polyclonal | No | Rabbit Polyclonal; Thermo/Lab Vision RB-9020-P1 | 1:400 |
| **Keratin 14** | Polyclonal | No | Chicken Polyclonal; Biolegend 906001 | 1:400 |
| **Keratin 18** | DC10 | No | Mouse IgG1; Thermo/Lab Vision MS142P0 | 1:400 |
| **Keratin 19** | A53-B/A2.26; same as Ks19.1 | No | IgG2a/λ; Lab Vision/Neomarkers MS-198-PABX | 1:400 |
| **Keratin 8** | | No | Mouse IgG2a κ | 1:400 |
| **P63** | 4A4 | None | Abcam ab32353 | 1:100 |
| **Pan-Laminin** | Polyclonal | No | Rabbit Polyclonal; L9393 | 1:400 |
| **Pan-Laminin** | Polyclonal | No | Rabbit Polyclonal; L9393 | 1:400 |
| **Podoplanin** | NC-08 | No | Rat IgG2a, λ | ½ assay[¶] |
| **Facs Panel Antibodies / Viability Dyes** | | | | |
| **CD49f (alpha-6 integrin)** | GoH3 | BV421* | BioLegend 313624, 313612, 313607 | 2.5ml[§] |
| **CD24** | ML5 | BV605* | BioLegend 311124, 311106, 311108 | 2.5ml[§] |
| **CD227 (Muc1)** | 16A | PE | BioLegend 355604, | 4ml[§] |
| **Podoplanin** | NC-08 | A488[†] | BioLegend 337006, 337004 | 2.5ml[§] |
| **CD45** | HI30 | A488[†] | BioLegend 304052, 304032 | 3ml[§] |
| **CD90 (Thy1)** | 5E10 | PE-Cy5 | BioLegend 328112, 328134 | 3ml[§] |
| **CD34** | 581 | PE-Cy7 | BioLegend 343516 | 1-2ml[§] |
| **CD10** | HI10a | APC-Cy7 | BioLegend 312212, 312204 | 3ml[§] |
| **TO-PRO™-3 Iodide** | N/A | (642/661) | Thermo (T3605) | 1:4000 |

** Brilliant Violet; † Alexa Fluor; (Biolegend);* § 1x volume per $6x10^6$ cells stained in FACS experiments (up to $36x10^6$, after which cells received 6x volumes; max stained cells $=120x10^6$); titration required and must be matched to cytometer. ¶ Percentage of manufacturer's suggested assay volume per 100ul volume (antibody is prediluted).

**Table S1. Antibodies.** Antibody clones used for immunostaining and FACS.

Figure S6

a

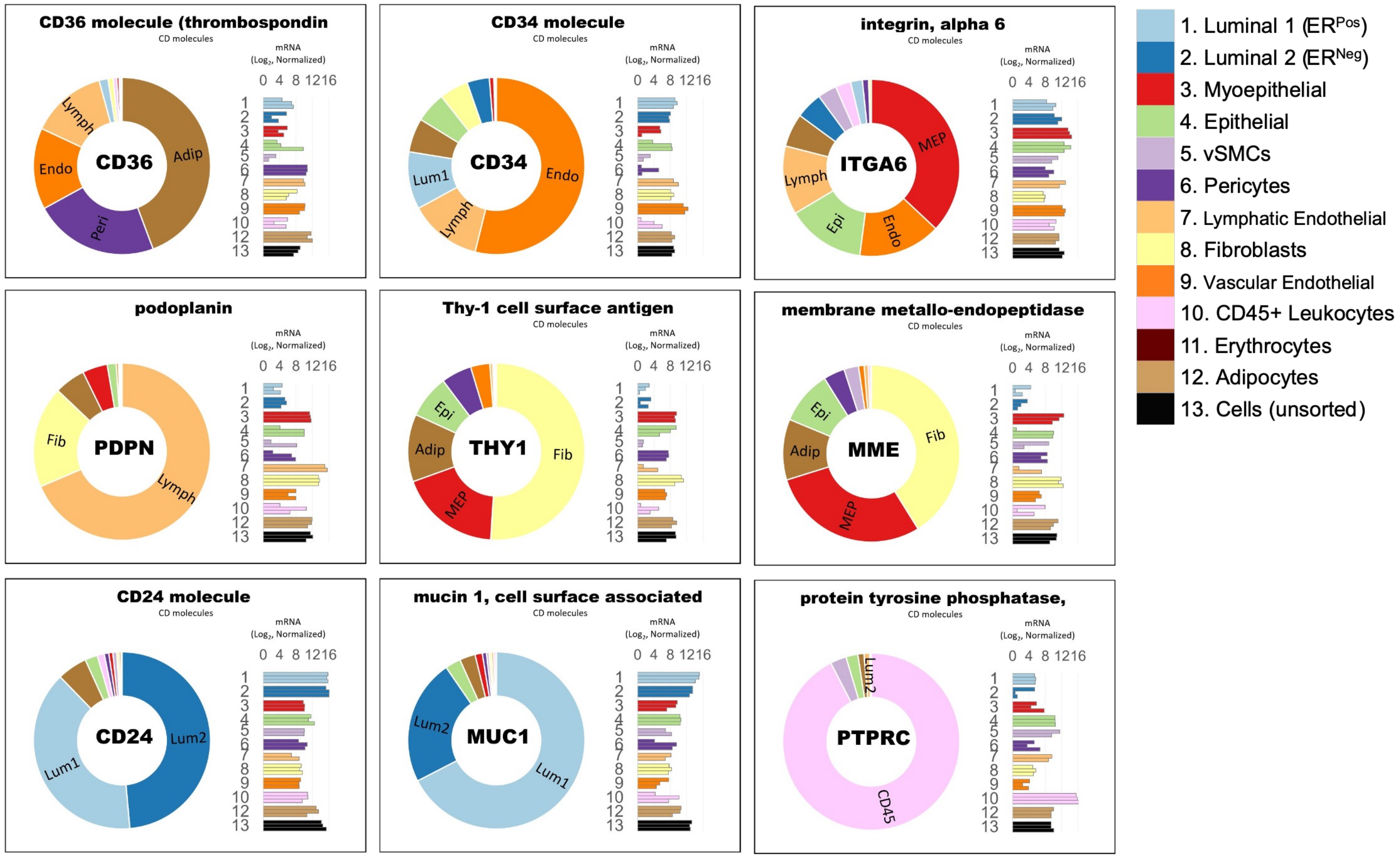


b

| Pop ID | Cell Type | CD36† | CD34 | CD49f (ITGA6) | PDPN | CD90 (Thy1) | CD10 (MME) | CD24 | CD227 (MUC1) | CD45 (PTPRC) |
|---|---|---|---|---|---|---|---|---|---|---|
| | | ANTIBODY STAINING IN SORTING SCHEME | | | | | | | | |
| 1 | $ER^{Pos}$ Luminal Epithelial | | Neg | -/+ | Neg | Neg | Neg | +++ | +++ | Neg |
| 2 | $ER^{Neg}$ Luminal Epithelial | | Neg | ++ | Neg | Neg | Neg | +++ | +++ | Neg |
| 3 | Myoepithelial | | Neg | ++++ | ++ | +++ | +++ | Neg | Neg | Neg |
| 4 | Pop4 Epithelial | | Neg | ++ | + | Neg | -/+ | Neg | Neg | Neg |
| 5 | Vascular Smooth Muscle Cells | | Neg | Neg | -/+ | Neg | Neg | Neg | Neg | Neg |
| 6 | Pericytes | | Neg | Neg | Neg | +++ | Neg | Neg | Neg | Neg |
| 7 | Lymphatic Endothelial Cells | | +++ | ++ | +++ | -/+ | Neg | Neg | Neg | Neg |
| 8 | Adipocyte Derived Mesenchymal Stem Cells (Fibroblasts) | | +++ | Neg | + | ++ | + | Neg | Neg | Neg |
| 9 | Vascular Endothelial Cells | | +++ | ++ | Neg | -/+ | Neg | Neg | Neg | Neg |
| 10 | Leukocytes ($CD45^{Pos}$) | | Neg | Neg | Neg | Neg | -/+ | Neg | Neg | +++ |
| 11 | Red Blood Cells | | Neg | Neg | Neg | Neg | Neg | Neg | Neg | Neg |
| 12 | Adipocytes | +++ | | | | | | | | |

c

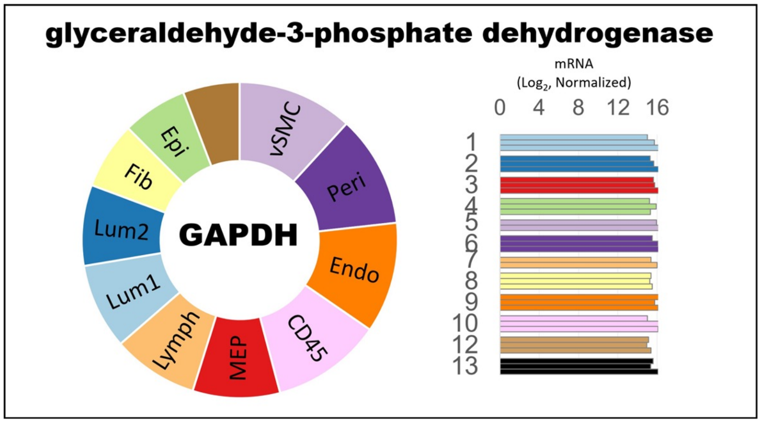

**Figure S6. mRNA levels correspond to FACS staining. A,** Normalized mRNA values for genes encoding FACS markers used to purify cell types from tissues (and CD36, which was later used to purify cultured adipocytes). Transcript levels are provided on a) a log2 scale (bar graphs containing each biological replicate) and b) a linear scale (donut graphs of the replicate's median value), which are both color-coded by cell type. **B,** Schematic indicating relative FACS staining levels of each marker in the twelve breast cell types. "Neg" indicates relative negative staining (i.e., compared to other populations, not absolute negativity), "-/+" indicates near background level staining, and the increasing number of "+" indicates greater staining intensity. † CD36 is a marker used to purify cultured adipocytes but was not used to purify adipocytes prior to RNA-sequencing in this analysis. **C,** Normalized transcript levels for the commonly used internal control gene GAPDH (rlog, DEseq2) are presented on a log2 scale (bar graph) and linear scale (donut graph of median value). (Data values for donut graphs can be found in S13 Data)

Figure S7

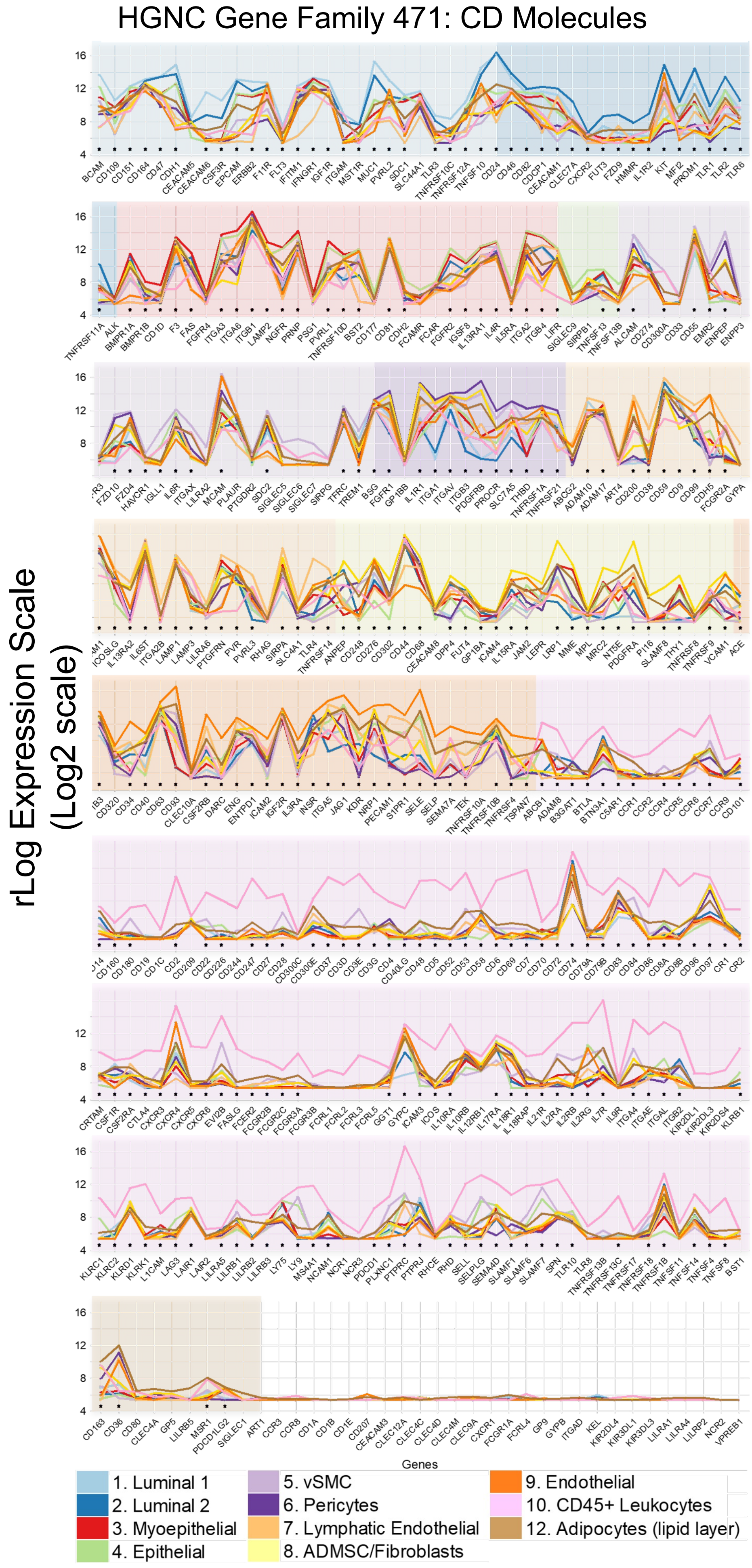
HGNC Gene Family 471: CD Molecules
rLog Expression Scale
(Log2 scale)
Genes
1. Luminal 1
2. Luminal 2
3. Myoepithelial
4. Epithelial
5. vSMC
6. Pericytes
7. Lymphatic Endothelial
8. ADMSC/Fibroblasts
9. Endothelial
10. CD45+ Leukocytes
12. Adipocytes (lipid layer)

**Figure S7. CD antigen genes.** Median mRNA expression levels of genes encoding CD antigens (rlog, log2 scale; S13 Data)**.** The genes are arranged by the cell type that expressed them at their highest level, as indicated by the background shading specific to each cell type. Genes differentially expressed across cell populations are annotated with (*). CD antigen genes in the uncolored panel were not expressed by any breast cell type.

Figure S8

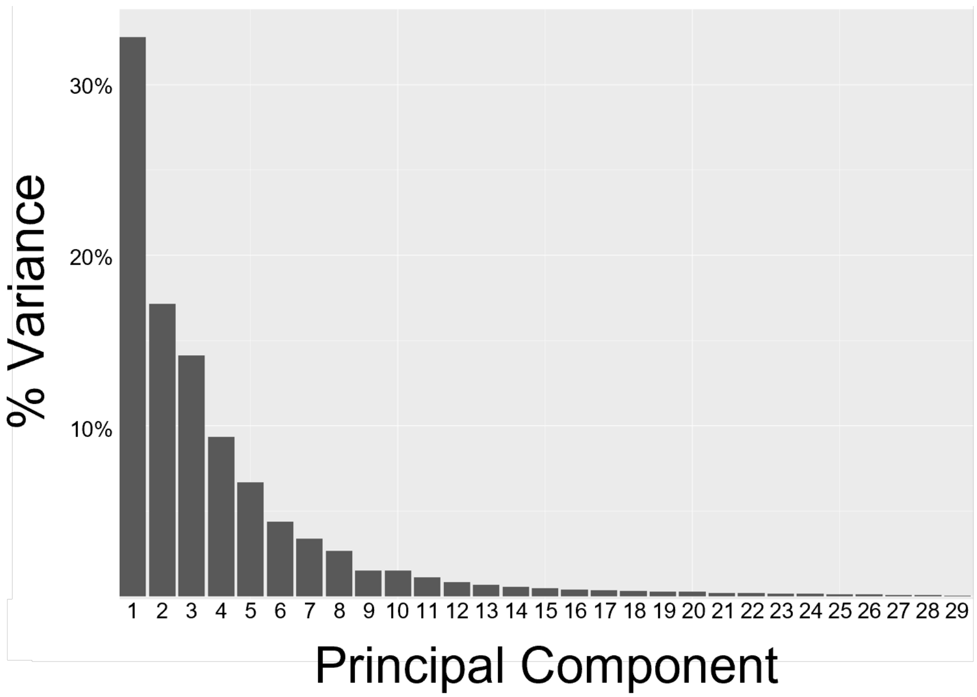


**Figure S8. Variance captured by principal components.** A screeplot of sample variances captured by each principal component. 71% of the total variance is captured by the first four components (S13 Data).

Figure S9

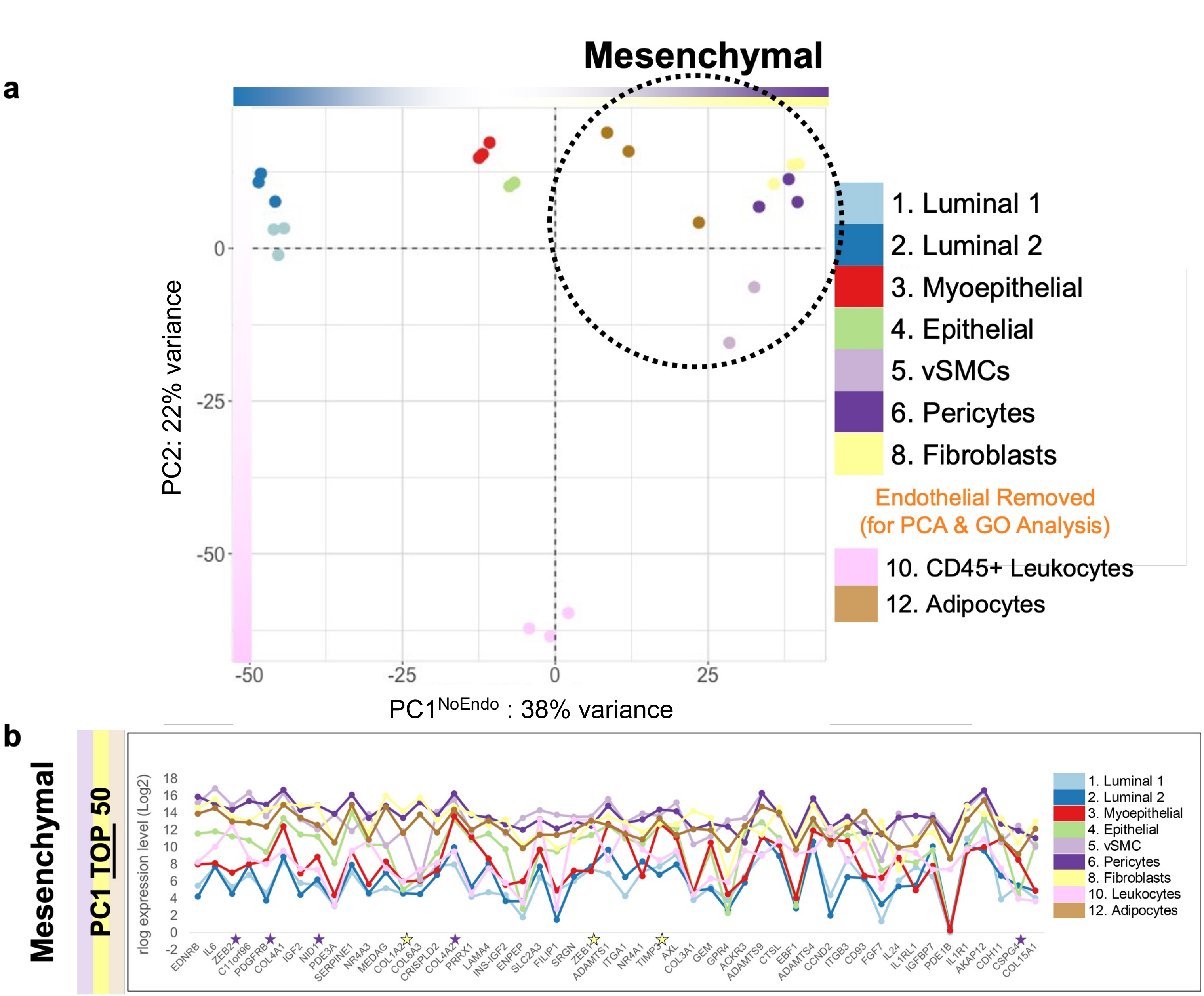


**Figure S9. PCA with endothelial cells removed. A,** PCA projection of breast cell types (after removal of the lymphatic and vascular endothelial cells; S3 Data) resolves the mesenchymal cell types (on PC1$^{NoEndo}$). **B,** Median mRNA expression of the 50 loading factors (genes) contributing to the positive end of PC2 (top end of the list; rlog, log2 scale; S13 Data). Stars (★) denote common markers of pericytes (purple) and fibroblasts (yellow).

Figure S10

# Principal Component 2: Leukocytes (Mesodermal)

## Leukocytes

**a**

| (# pathways in group) | Representative Pathways | *Adj. p*-value | Associated Genes (from **PC2** **BOTTOM** 200) Leukocytes |
|---|---|---|---|
| 6 | adaptive immune response, lymphocyte activation, regulation of immune response … | 6.07E-46 | [BTK, CAMK4, CD247, CD28, CD3D, CD3E, CD48, CD6, CD7, CD74, CD79A, CD8A, CD8B, GPR183, HLA-DRA, HLA-DRB1, HLA-DRB5, IGLL5, IL7R, INPP5D, IRF4, ITK, JCHAIN, KLRK1, LY9, MYO1G, PIK3CG, PRF1, PRKCB, PRKCQ, PTK2B, PTPRC, SAMSN1, SASH3, SKAP1, SLA2, SLAMF6, SLAMF7, SPN, TARP, THEMIS, TNFRSF13C, WAS, ZAP70] |
| 15 | activation of immune response, T cell receptor signaling pathway, B cell receptor signaling pathway… | 1.78E-41 | [CARD11, CCR7, CD247, CD28, CD3D, CD3E, FYB1, HLA-DRA, HLA-DRB1, HLA-DRB5, INPP5D, ITK, LCK, LCP2, PRKCQ, PTPN22, PTPRC, SKAP1, SLA2, TARP, TESPA1, THEMIS, WAS, ZAP70] ; [BLK, BTK, CD22, CD79A, IGLL5, ITK, LCK, LPXN, MS4A1, NCKAP1L, PAX5, PRKCB, PTPN22, PTPRC] |
| 141 | leukocyte homeostasis, cytokine production, hemopoiesis, interleukin-10, -4, -2, -8 production, cell-cell adhesion… | 2.42E-41 | [CD74, CORO1A, DOCK10, DOCK11, GPR183, IL2RA, NCKAP1L, TNFRSF13C, TSC22D3] |
| 25 | T cell activation involved in immune response, Interleukin-17 production, T-helper 17 type immune response… | 1.94E-26 | [ADAM8, APBB1IP, ARHGAP45, ARHGAP9, ATP8A1, BIN2, BTK, CD28, CD53, CD74, CORO1A, DOCK10, DOCK11, DOCK2, GPR183, IQGAP2, IRF4, ITGAL, ITGB2, LCP1, LRMP, LY9, NCKAP1L, PIK3CG, PTGER4, PTK2B, PTPRC, RAC2, RASGRP1, SELL, SLAMF6, SPN] |
| 53 | leukocyte chemotaxis and migration, T cell chemotaxis, cellular extravasation… | 5.88E-23 | [ADAM8, CCL5, CCR5, CCR7, CD74, CORO1A, CXCR4, GPR183, IL16, ITGB2, JAML, KLRK1, NCKAP1L, PIK3CG, PREX1, PTK2B, RAC2, RIPOR2 STK4, VAV1] |
| 6 | phagocytosis, Fc-gamma receptor signaling pathway involved in phagocytosis… | 8.85E-22 | [BIN2, CD247, CORO1A, CYFIP2, DOCK2, IGLL5, IL2RB, IL2RG, ITGAL, ITGB2, MYO1G, NCKAP1L, PTPRC, RAC2, TARP, VAV1, WAS, WIPF1] |
| 36 | Interleukin-6 production, interleukin-1 secretion… | 5.64E-17 | [BANK1, CD74, IL16, INPP5D, LCP1, NCKAP1L, PTPN22] |
| 10 | acute inflammatory response… | 3.69E-14 | [ADAM8, ALOX5AP, BTK, CCR7, CD6, PIK3CG] |
| 9 | regulation of GTPase activity, Ras- and Rho- guanyl-nucleotide exchange factor activity… | 1.29E-11 | [ACAP1, ARAP2, ARHGAP15, ARHGAP30, ARHGAP45, ARHGAP9, ARHGDIB, CCL5, CCR7, DOCK10, DOCK11, DOCK2, DOCK8, FGD3, IQGAP2, KLRK1, NCKAP1L, PREX1, PTK2B, RASAL3, RASGRP1, RASGRP2, RGS1, RGS2, RHOH, SMAP2, TAGAP, VAV1] |
| 19 | mast cell degranulation, inflammatory response to antigenic stimulus… | 3.44E-11 | [BTK, CCR7, CD28, CD6, HLA-DRB1, IL2RA, RASGRP1] |
| 6 | monocyte differentiation… | 9.53E-11 | [EVI2B, HCLS1, INPP5D] |

**b**

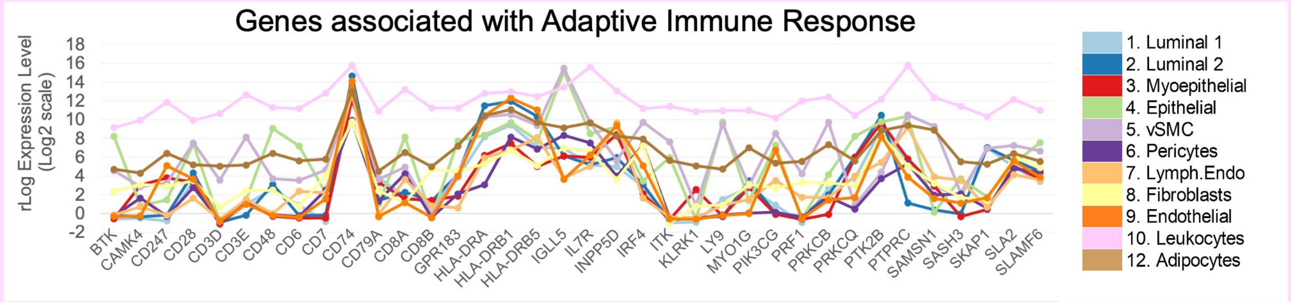


**c**

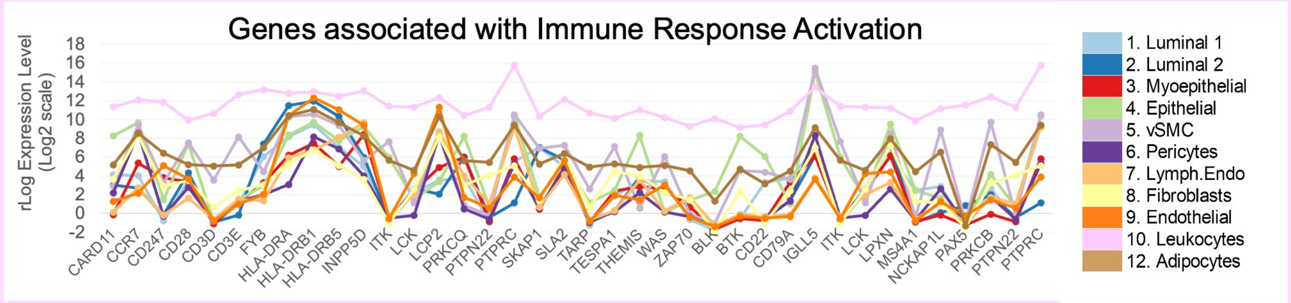


**d**

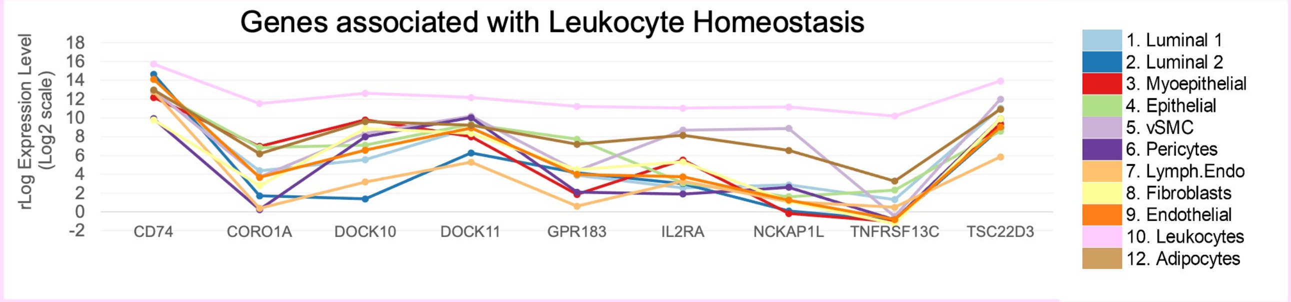

**Figure S10. Pathway analysis of leukocyte genes (PC2). A,** Grouped GO/Reactome terms associated with the 200 genes contributing to the positive end (bottom of list) of PC1 ordered by adjusted p-value. Median mRNA expression (rlog transformed) for PC2 genes associated with select pathways, including **B,** a*daptive immune response*, **C,** *immune response activation*, and **D,** *leukocyte homeostasis*. (Data values for line graphs are found in S13 Data)

Figure S11

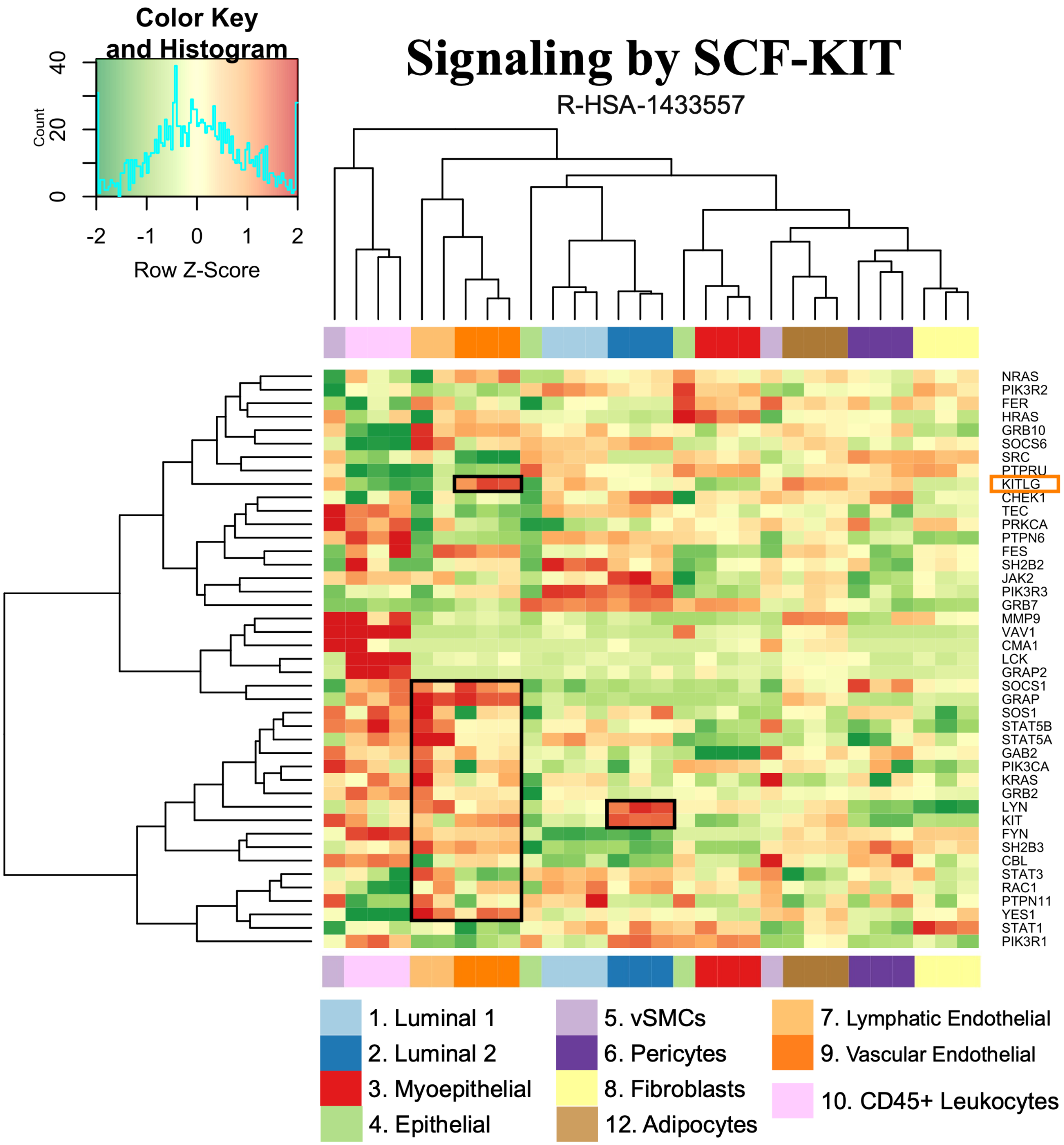


**Figure S11. Transcript levels of genes composing the *signaling by SCF-KIT* pathway.** Transcript levels for each gene in the Reactome pathway *signaling by SCF-KIT*. VST gene levels are relative (normalized within each row of the heatmap; S13 Data). Boxed areas highlight expression of select genes in lymphatic and vascular endothelial, and $ER^{Neg}$ luminal epithelial cell types.

Figure S12

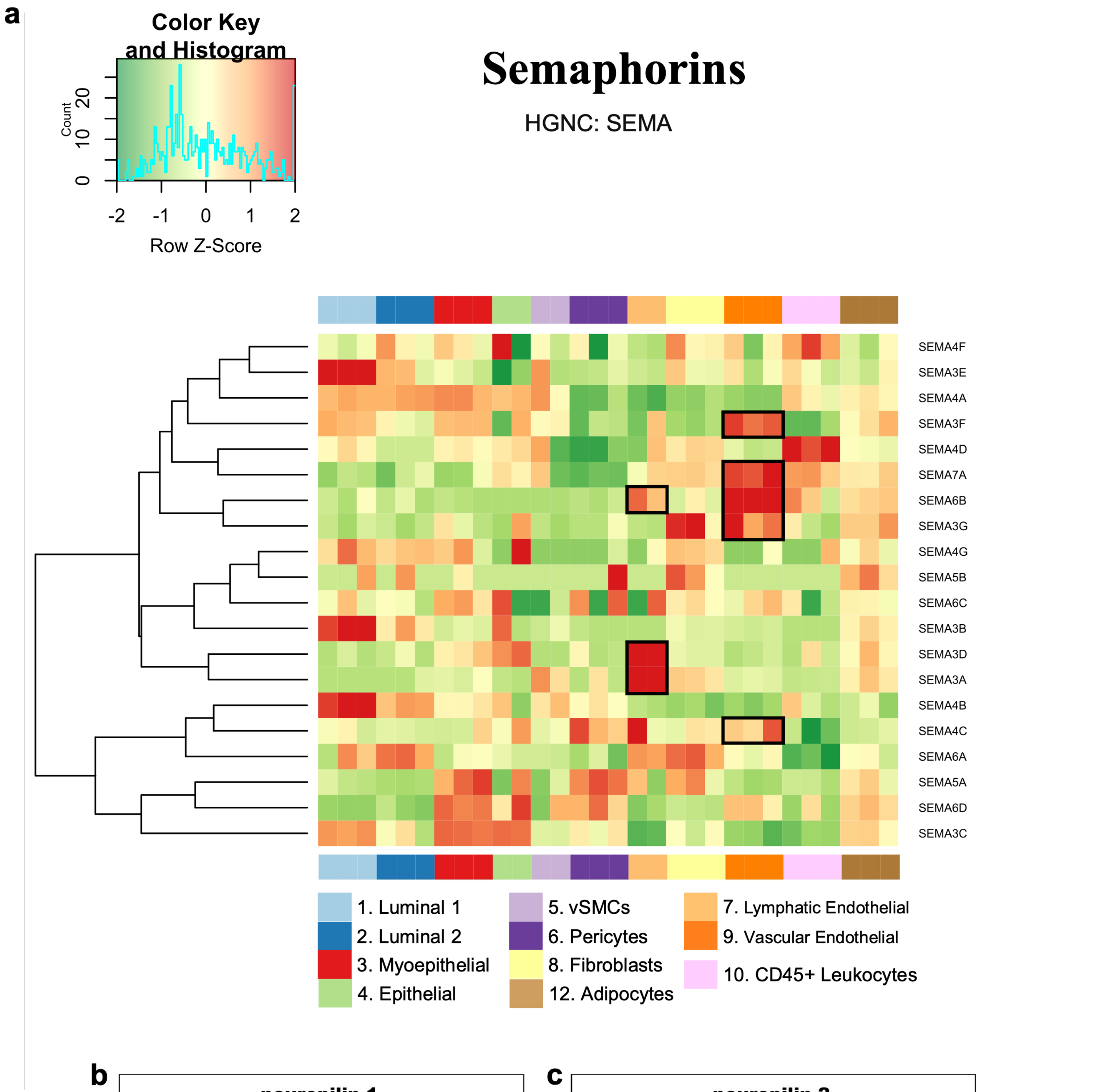
a
Color Key
and Histogram
Count
0
10
20
-2
-1
0
1
2
Row Z-Score
Semaphorins
HGNC: SEMA
SEMA4F
SEMA3E
SEMA4A
SEMA3F
SEMA4D
SEMA7A
SEMA6B
SEMA3G
SEMA4G
SEMA5B
SEMA6C
SEMA3B
SEMA3D
SEMA3A
SEMA4B
SEMA4C
SEMA6A
SEMA5A
SEMA6D
SEMA3C
1. Luminal 1
2. Luminal 2
3. Myoepithelial
4. Epithelial
5. vSMCs
6. Pericytes
8. Fibroblasts
12. Adipocytes
7. Lymphatic Endothelial
9. Vascular Endothelial
10. CD45+ Leukocytes

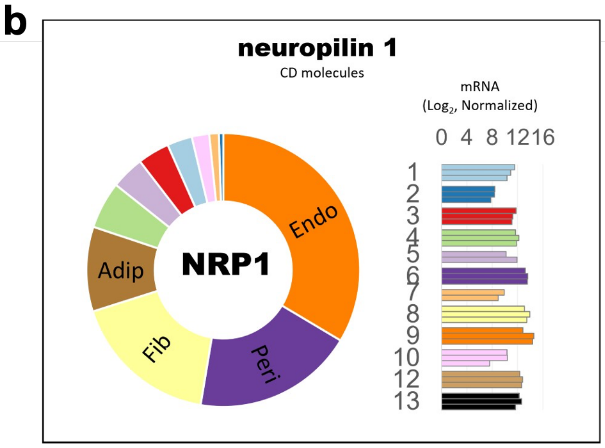
b
neuropilin 1
CD molecules
mRNA
(Log2, Normalized)
0 4 8 12 16
NRP1
Endo
Peri
Fib
Adip
1
2
3
4
5
6
7
8
9
10
12
13

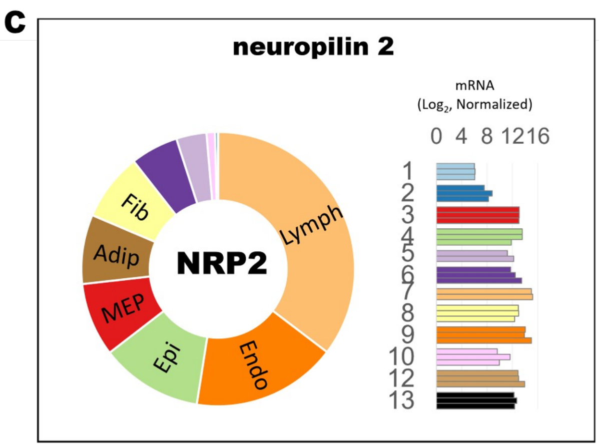
c
neuropilin 2
mRNA
(Log2, Normalized)
0 4 8 12 16
NRP2
Lymph
Endo
Epi
MEP
Adip
Fib
1
2
3
4
5
6
7
8
9
10
12
13

**Figure S12. Transcript levels of genes composing the *Semaphorins* gene family. A,** Transcript levels of genes in Semaphorin gene family. VST gene levels are relative (normalized within each row of the heatmap; S13 Data). Boxed areas highlight select genes highly expressed by the lymphatic and vascular endothelial cell types. Normalized mRNA values of **B,** NRP1 and **C,** NRP2 (rlog, DEseq2) are provided on log2 scale (bar graph of each biological replicate) and linear scale (donut graph of median value), which are both color-coded by cell type. (Data values for donuts are found in S13 Data).

Table S2

| Gene Family | DE Genes | Genes in Family |
|---|---|---|
| Adenylate cyclases | 9 | 10 |
| Aldehyde dehydrogenases | 16 | 19 |
| Collagens | 38 | 45 |
| Death inducing signaling complex | 6 | 6 |
| DOCK family Rho GEFs | 11 | 11 |
| Ephrins | 6 | 8 |
| Erb-b2 receptor tyrosine kinases | 4 | 4 |
| Fibulins | 7 | 7 |
| Glutathione S-transferases | 15 | 23 |
| GTPases, IMAP | 7 | 7 |
| Histocompatibility complex | 14 | 14 |
| Histones | 37 | 89 |
| Integrin alpha subunits | 17 | 18 |
| Integrin beta subunits | 9 | 9 |
| Interleukins | 18 | 37 |
| Interleukin receptors | 34 | 42 |
| Keratins, Type I | 7 | 26 |
| Keratins, Type II | 10 | 26 |
| Kruppel like factors | 14 | 17 |
| Laminin subunits | 12 | 12 |
| Lysophosphatidic acid receptors | 6 | 6 |
| MAP kinase phosphatases | 10 | 11 |
| Matrix metallopeptidases | 14 | 23 |
| Mucins | 10 | 14 |
| Plexins | 9 | 9 |
| Ras association domain family | 10 | 10 |
| Rho GTPase activating proteins | 46 | 48 |
| Suppressors of cytokine signaling | 8 | 8 |
| Tetraspanins | 22 | 33 |
| Toll like receptors | 9 | 10 |
| V-type ATPases | 10 | 23 |

**Table S2. Variably expressed gene types.** Genes composing each gene family (HGNC) were assessed by DESeq2 (Adj.*p*-value $\leq 1$, across all cell types). The number of genes in each family, along with the numer of those found to be differentially expressed are provided (for gene families $\geq 6$ genes).

Figure S13

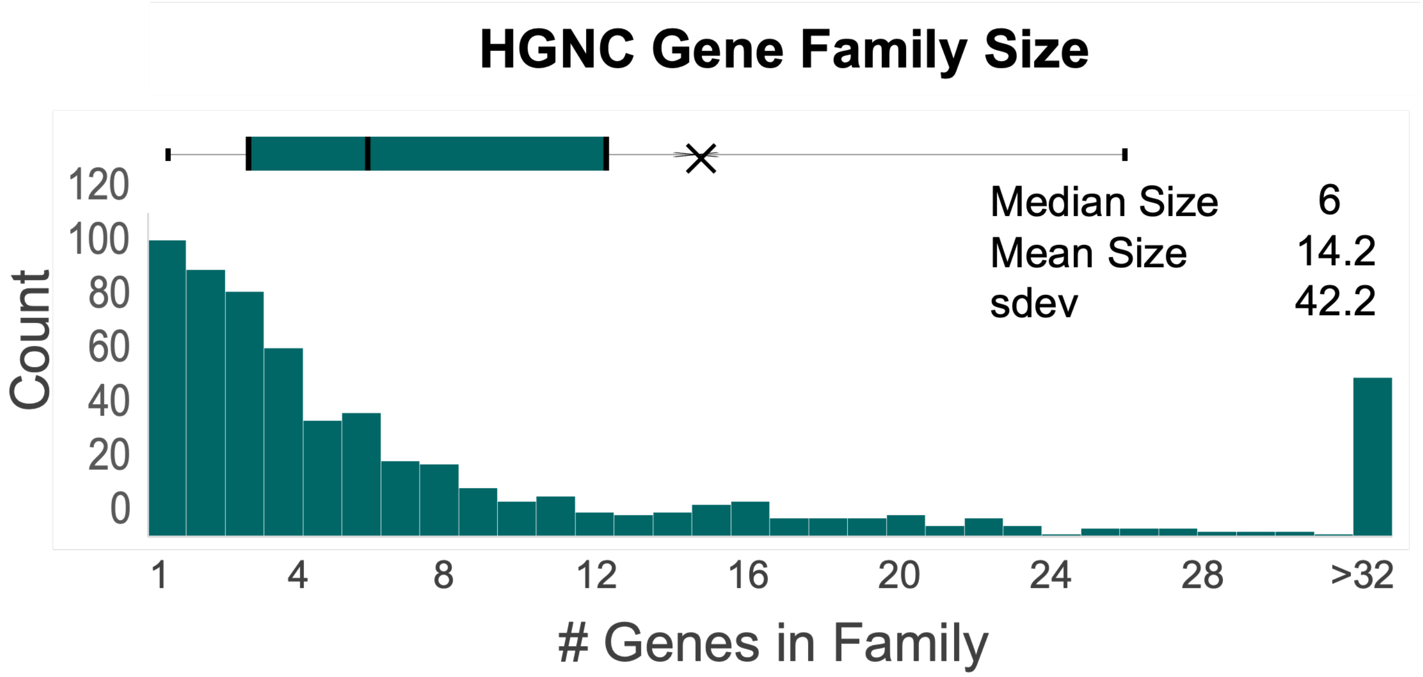


**Figure S13. Distribution of gene family size.** The sizes of the HGNC families range from 1 to 831 genes. The distribution of these family sizes is provided, along with a box & whisker plot (top). The median family size is six genes per family. (S13 Data)

Figure S14

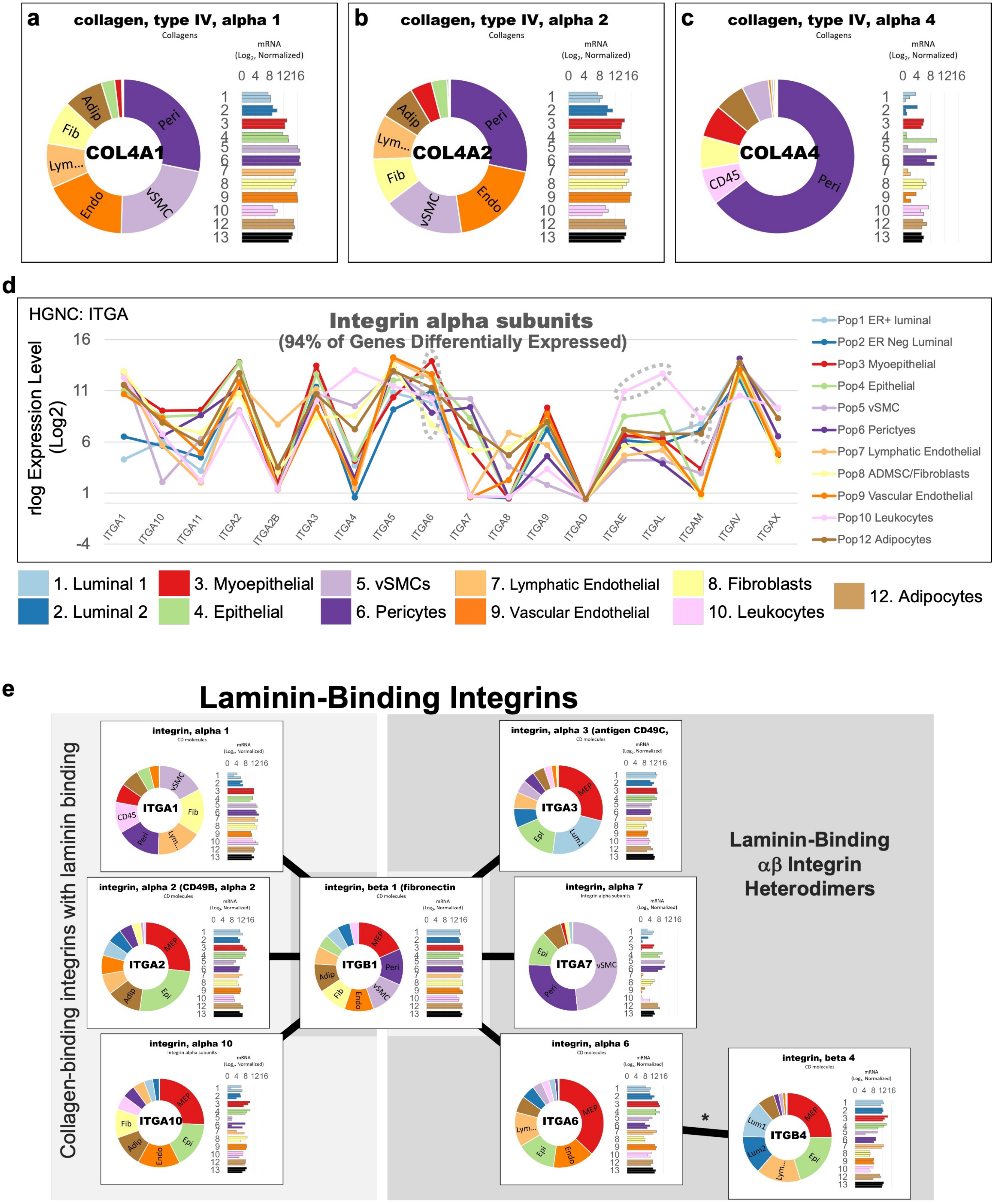
a
collagen, type IV, alpha 1
Collagens
mRNA (Log2, Normalized)
COL4A1
b
collagen, type IV, alpha 2
Collagens
COL4A2
c
collagen, type IV, alpha 4
Collagens
COL4A4
d
HGNC: ITGA
Integrin alpha subunits
(94% of Genes Differentially Expressed)
rlog Expression Level (Log2)
Pop1 ER+ luminal
Pop2 ER Neg Luminal
Pop3 Myoepithelial
Pop4 Epithelial
Pop5 vSMC
Pop6 Pericytes
Pop7 Lymphatic Endothelial
Pop8 ADMSC/Fibroblasts
Pop9 Vascular Endothelial
Pop10 Leukocytes
Pop12 Adipocytes
1. Luminal 1
2. Luminal 2
3. Myoepithelial
4. Epithelial
5. vSMCs
6. Pericytes
7. Lymphatic Endothelial
9. Vascular Endothelial
8. Fibroblasts
10. Leukocytes
12. Adipocytes
e
Laminin-Binding Integrins
Collagen-binding integrins with laminin binding
Laminin-Binding αβ Integrin Heterodimers
integrin, alpha 1
ITGA1
integrin, alpha 3 (antigen CD49C,
ITGA3
integrin, alpha 2 (CD49B, alpha 2
ITGA2
integrin, beta 1 (fibronectin
ITGB1
integrin, alpha 7
ITGA7
integrin, alpha 10
ITGA10
integrin, alpha 6
ITGA6
integrin, beta 4
ITGB4

**Figure S14. Transcript levels of DE genes.** Transcript levels for select Type IV collagen chains: **A,** COL4A1, **B,** COL4A2, and **C,** COL4A4. **D,** Median mRNA expression (rlog, log2 scale) for integrin alpha subunits. The dashed circles identify notable differentially expressed genes, ITGA6, ITGAE, and ITGAM. **E,** Transcript levels of the laminin binding integrins. The predominant laminin-binding integrins are αβ heterodimers of: α3β1, α7β1, α7β1 and α6β4. These are integrins are encoded by ITGB1, ITGA3, ITGA7, ITGA6, and ITGB4. Collagen-binding integrins that also show laminin binding properties include heterodimers of: α1β1, α2β1, and α10β1 (encoded by ITGA1, ITGA2, ITGA10, and ITGB1). ITGA7 is predominantly limited to the perivascular cell types, whereas ITGA3, ITGA6, and ITGB4 are largely expressed by the epithelial cell types. (All data values are found in S13 Data).

Figure S15

**a**

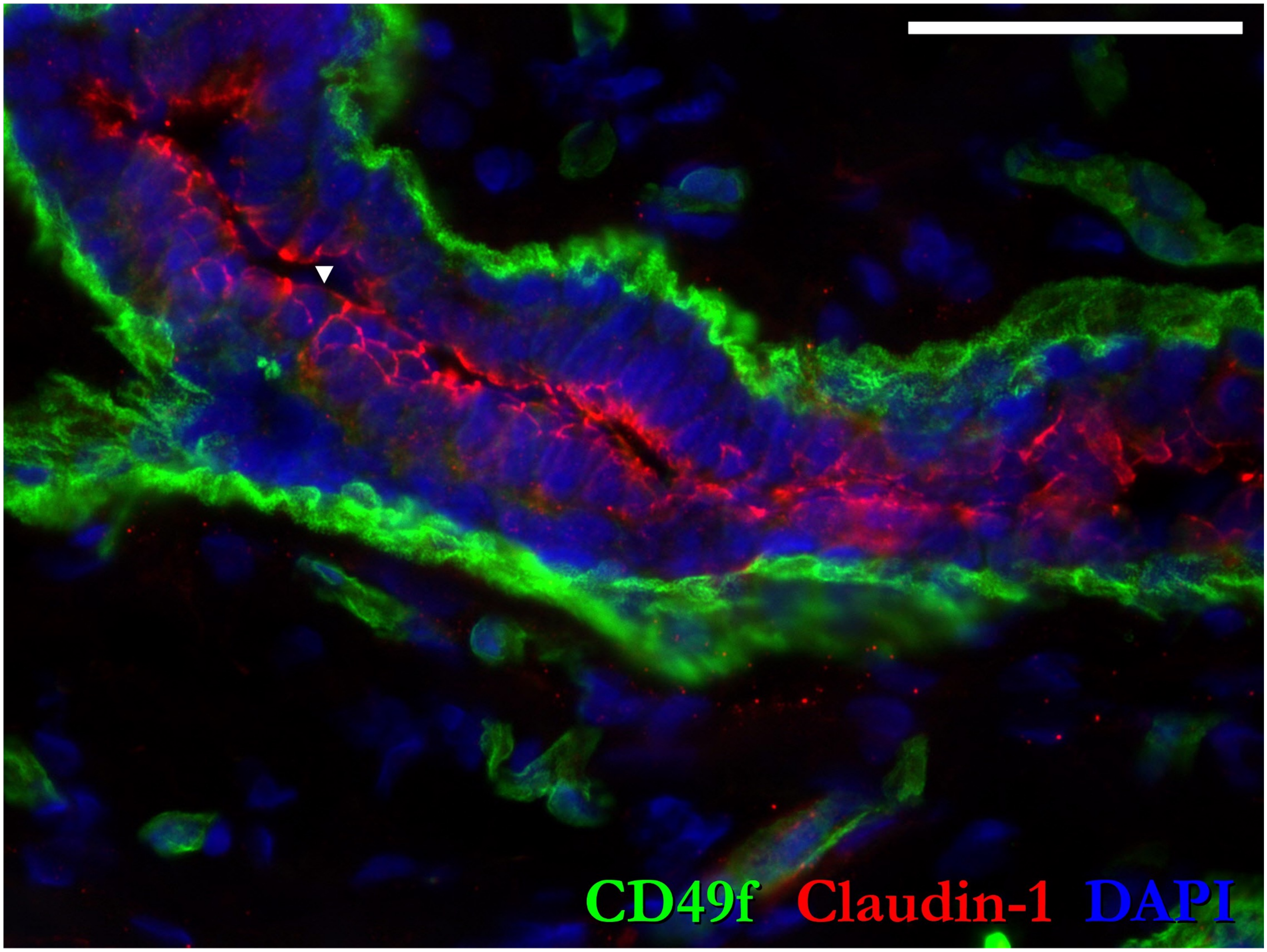


**Figure S15. IHC staining. A,** Claudin-1 staining of normal breast tissue (reduction mammoplasty of 28-year-old female). (▼) Claudin-1 staining is found confined to the apical surface of the paracellular space of luminal epithelial cells, circumscribing the luminal surface of the cells. Scale = 50 μm.

Figure S16

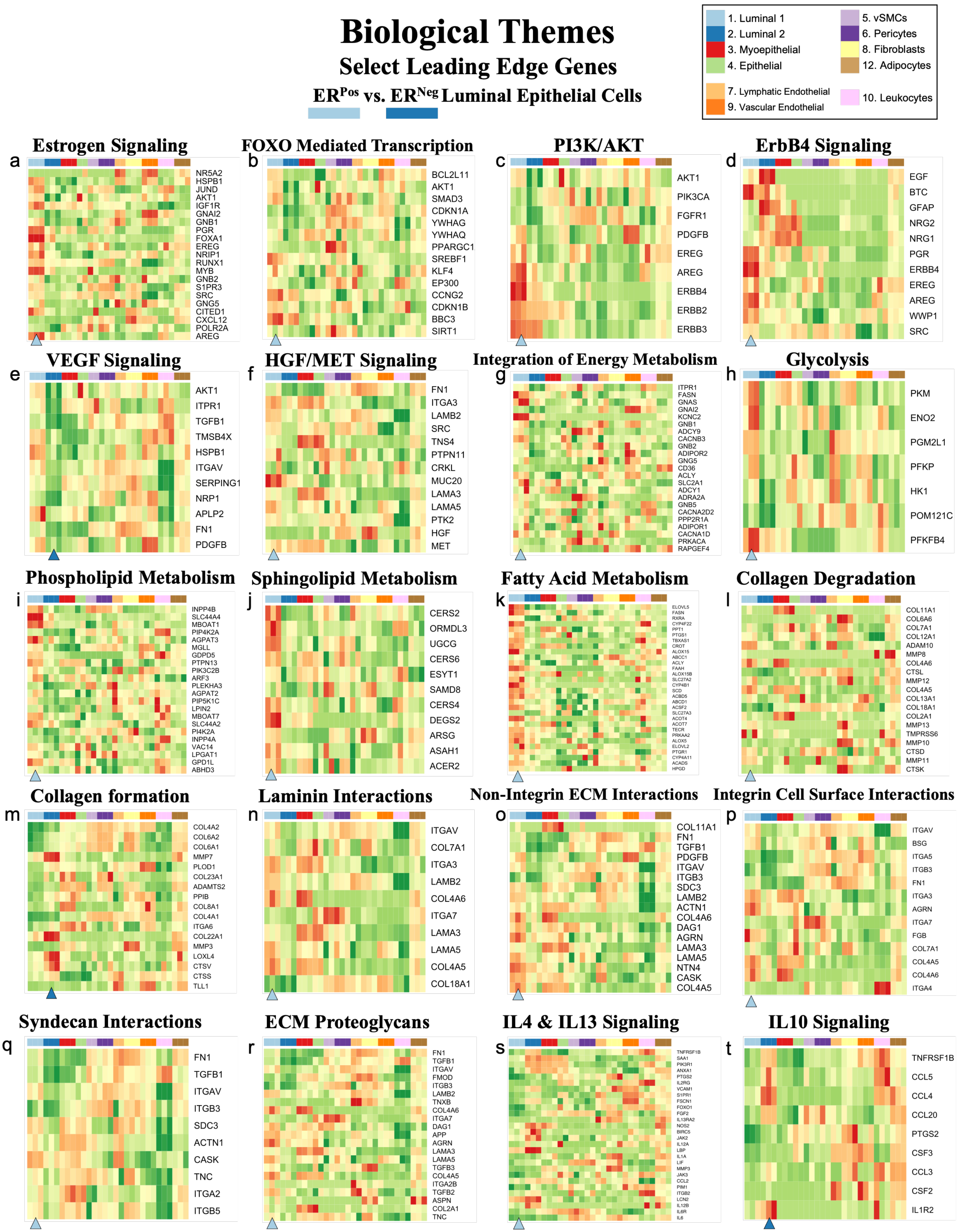

Biological Themes
Select Leading Edge Genes
ER^Pos vs. ER^Neg Luminal Epithelial Cells
1. Luminal 1
2. Luminal 2
3. Myoepithelial
4. Epithelial
5. vSMCs
6. Pericytes
8. Fibroblasts
12. Adipocytes
7. Lymphatic Endothelial
9. Vascular Endothelial
10. Leukocytes
a Estrogen Signaling
NR5A2 HSPB1 JUND AKT1 IGF1R GNAI2 GNB1 PGR FOXA1 EREG NRIP1 RUNX1 MYB GNB2 S1PR3 SRC GNG5 CITED1 CXCL12 POLR2A AREG
b FOXO Mediated Transcription
BCL2L11 AKT1 SMAD3 CDKN1A YWHAG YWHAQ PPARGC1 SREBF1 KLF4 EP300 CCNG2 CDKN1B BBC3 SIRT1
c PI3K/AKT
AKT1 PIK3CA FGFR1 PDGFB EREG AREG ERBB4 ERBB2 ERBB3
d ErbB4 Signaling
EGF BTC GFAP NRG2 NRG1 PGR ERBB4 EREG AREG WWP1 SRC
e VEGF Signaling
AKT1 ITPR1 TGFB1 TMSB4X HSPB1 ITGAV SERPING1 NRP1 APLP2 FN1 PDGFB
f HGF/MET Signaling
FN1 ITGA3 LAMB2 SRC TNS4 PTPN11 CRKL MUC20 LAMA3 LAMA5 PTK2 HGF MET
g Integration of Energy Metabolism
ITPR1 FASN GNAS GNAI2 KCNC2 GNB1 ADCY9 CACNB3 GNB2 ADIPOR2 GNG5 CD36 ACLY SLC2A1 ADCY1 ADRA2A GNB5 CACNA2D2 PPP2R1A ADIPOR1 CACNA1D PRKACA RAPGEF4
h Glycolysis
PKM ENO2 PGM2L1 PFKP HK1 POM121C PFKFB4
i Phospholipid Metabolism
INPP4B SLC44A4 MBOAT1 PIP4K2A AGPAT3 MGLL GDPD5 PTPN13 PIK3C2B ARF3 PLEKHA3 AGPAT2 PIP5K1C LPIN2 MBOAT7 SLC44A2 PI4K2A INPP4A VAC14 LPGAT1 GPD1L ABHD3
j Sphingolipid Metabolism
CERS2 ORMDL3 UGCG CERS6 ESYT1 SAMD8 CERS4 DEGS2 ARSG ASAH1 ACER2
k Fatty Acid Metabolism
l Collagen Degradation
COL11A1 COL6A6 COL7A1 COL12A1 ADAM10 MMP8 COL4A6 CTSL MMP12 COL4A5 COL13A1 COL18A1 COL2A1 MMP13 TMPRSS6 MMP10 CTSD MMP11 CTSK
m Collagen formation
COL4A2 COL6A2 COL6A1 MMP7 PLOD1 COL23A1 ADAMTS2 PPIB COL8A1 COL4A1 ITGA6 COL22A1 MMP3 LOXL4 CTSV CTSS TLL1
n Laminin Interactions
ITGAV COL7A1 ITGA3 LAMB2 COL4A6 ITGA7 LAMA3 LAMA5 COL4A5 COL18A1
o Non-Integrin ECM Interactions
COL11A1 FN1 TGFB1 PDGFB ITGAV ITGB3 SDC3 LAMB2 ACTN1 COL4A6 DAG1 AGRN LAMA3 LAMA5 NTN4 CASK COL4A5
p Integrin Cell Surface Interactions
ITGAV BSG ITGA5 ITGB3 FN1 ITGA3 AGRN ITGA7 FGB COL7A1 COL4A5 COL4A6 ITGA4
q Syndecan Interactions
FN1 TGFB1 ITGAV ITGB3 SDC3 ACTN1 CASK TNC ITGA2 ITGB5
r ECM Proteoglycans
FN1 TGFB1 ITGAV FMOD ITGB3 LAMB2 TNXB COL4A6 ITGA7 DAG1 APP AGRN LAMA3 LAMA5 TGFB3 COL4A5 ITGA2B TGFB2 ASPN COL2A1 TNC
s IL4 & IL13 Signaling
t IL10 Signaling
TNFRSF1B CCL5 CCL4 CCL20 PTGS2 CSF3 CCL3 CSF2 IL1R2

**Figure S16. Transcript levels from enriched pathways. A-T,** Transcript levels of leading-edge genes from select pathways enriched in $ER^{Pos}$ or $ER^{Neg}$ luminal cells (from 325 enriched pathways with a FDR of ≤ 10%, GSEA; S13 Data). VST gene levels are relative (normalized by row). Arrowheads under the heatmaps identify the cell type either over- or under-expressing the pathway's genes. Samples are color-coded. Due to space constraints and the large number of presented genes, their names (rows of the heatmap) must be inspected digitally (magnified in the PDF).

Figure S17

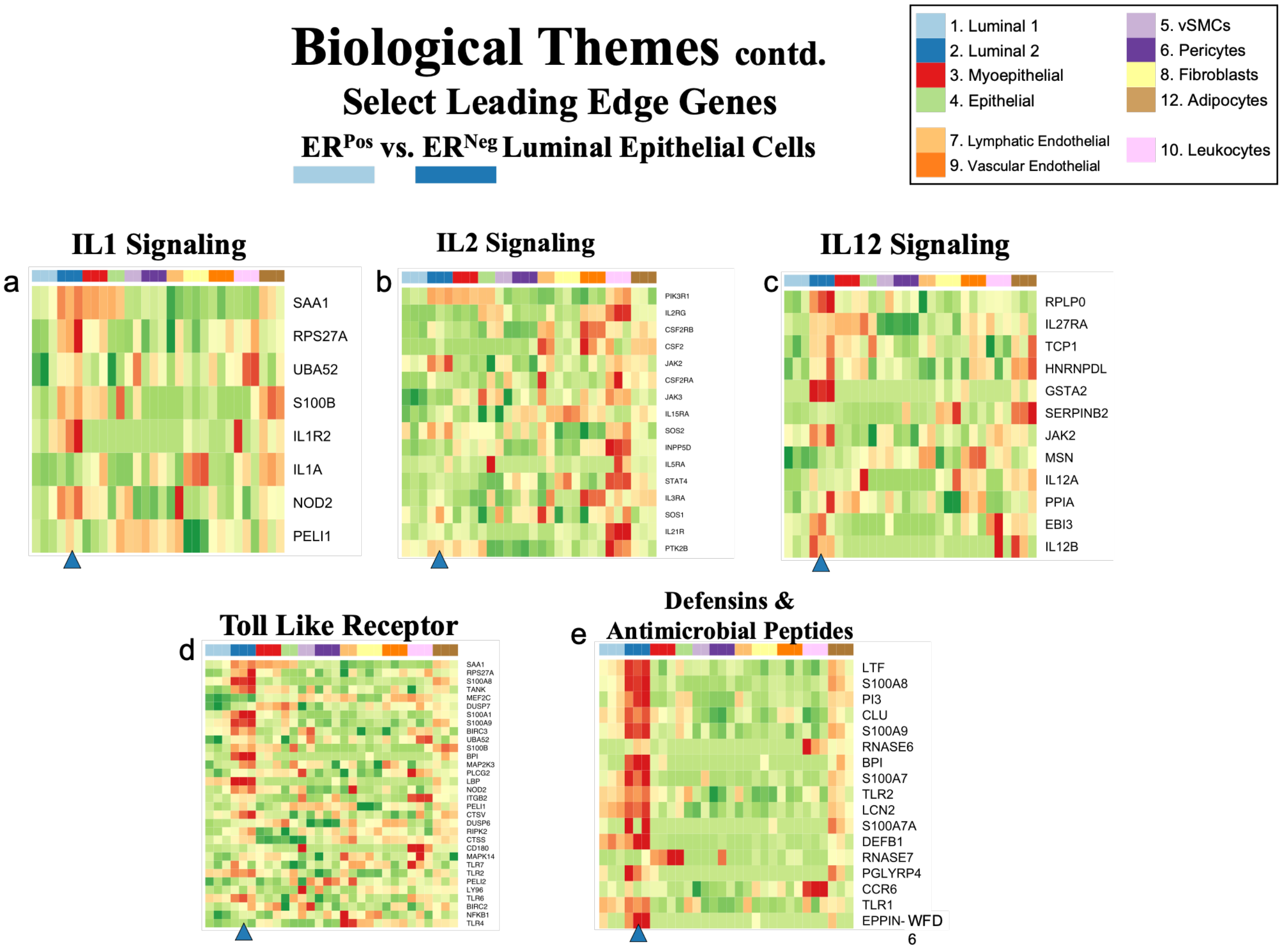


**Figure S17. Transcript levels from enriched pathways. A-E,** Transcript levels of leading-edge genes from select pathways enriched in $ER^{Pos}$ or $ER^{Neg}$ luminal cells (from 325 enriched pathways with a FDR of ≤ 10%, GSEA; S13 Data). VST gene levels are relative (normalized by row). Arrowheads under the heatmaps identify the cell type either over- or under-expressing the pathway's genes. Samples are color-coded. Due to space constraints and the large number of presented genes, their names (rows of the heatmap) must be inspected digitally (magnified in the PDF).

Figure S18

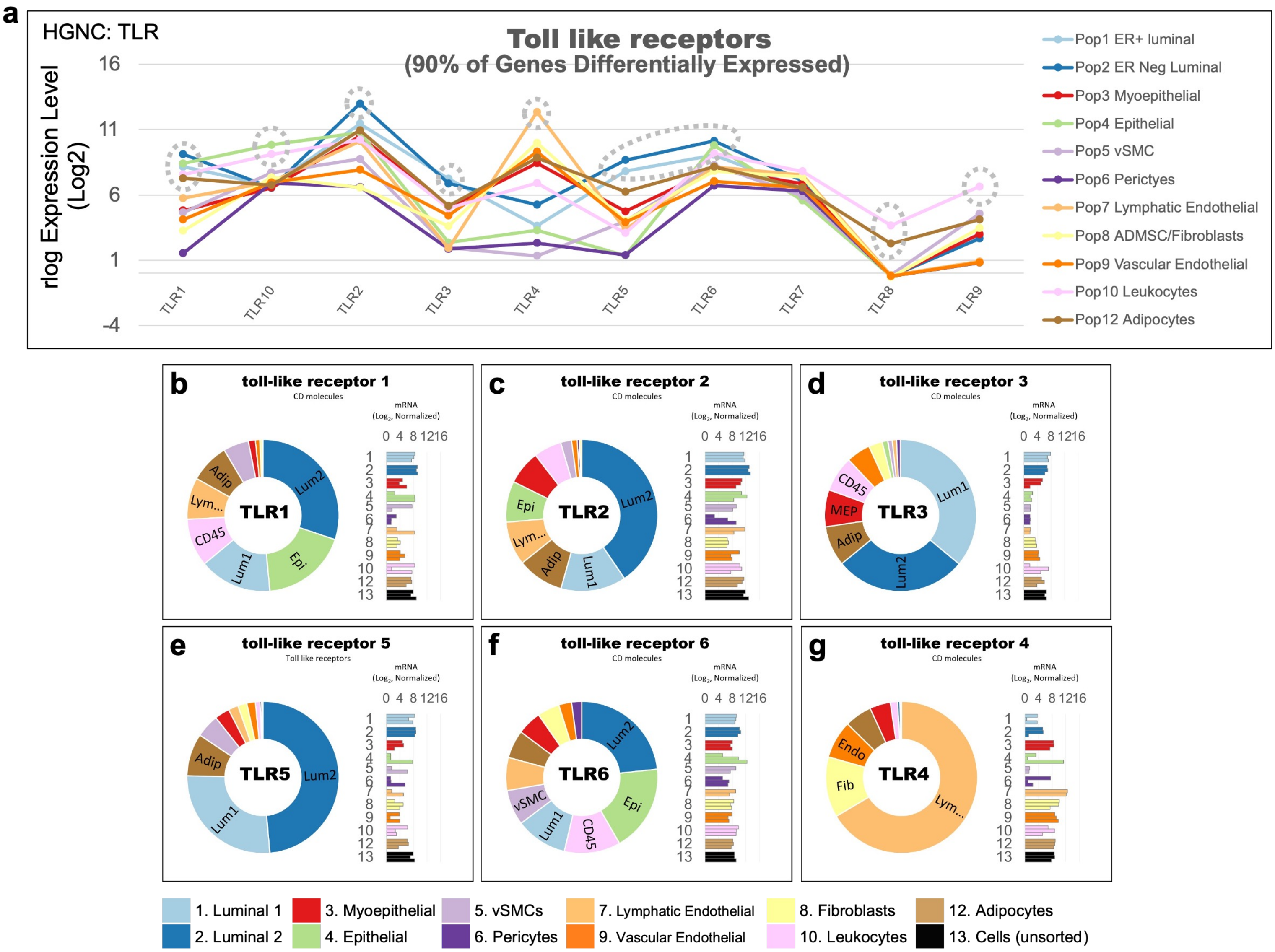


**Figure S18. Transcript levels of toll-like receptors A,** Transcript levels of TLR gene family. **B-F,** Normalized mRNA values (rlog, DEseq2) provided on log2 scale (bar graph of each biological replicate) and linear scale (donut graph of median value) which are both color-coded by cell type. (All data values are found in S13 Data).

Figure S19

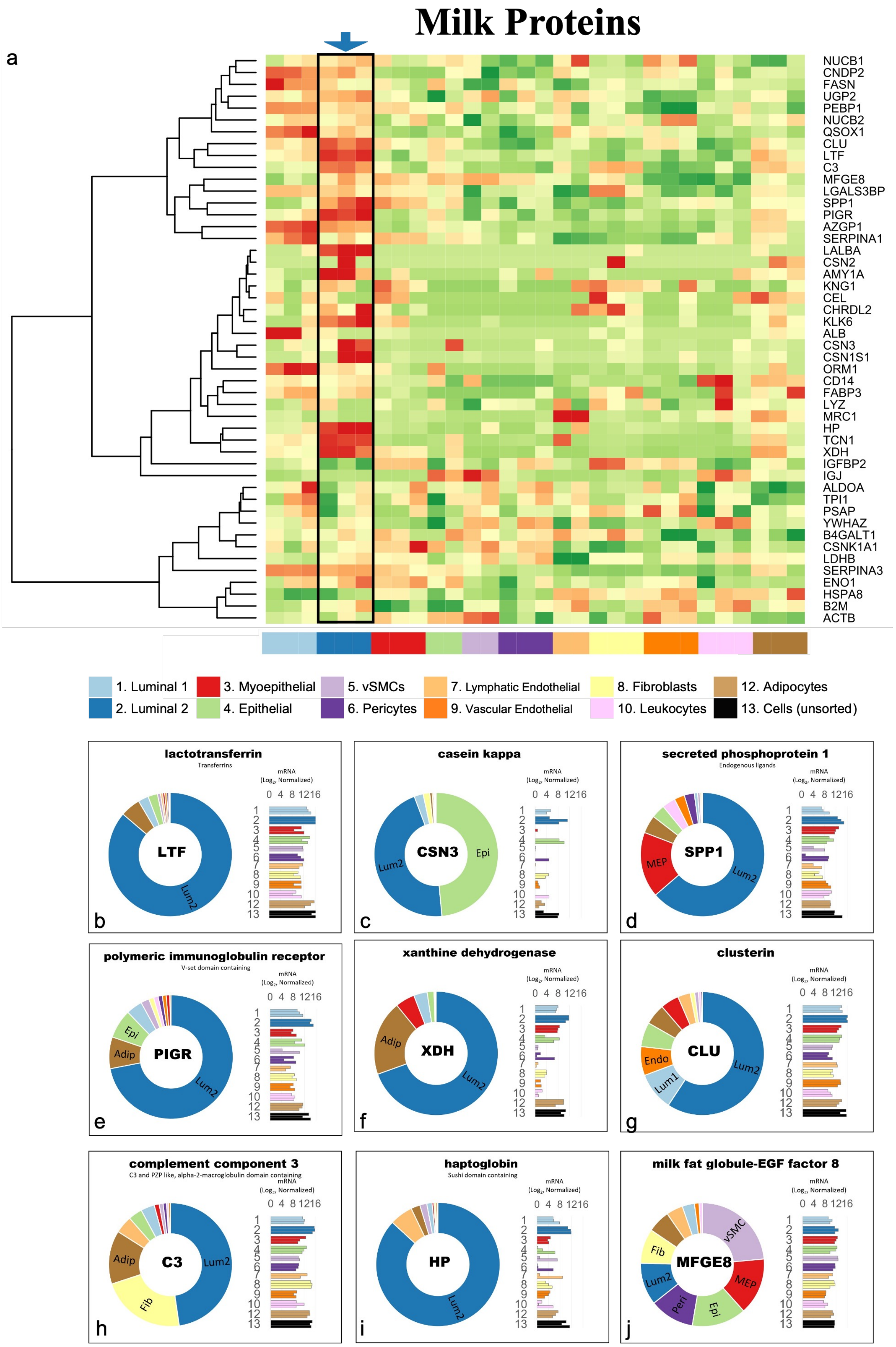
Milk Proteins
a
NUCB1
CNDP2
FASN
UGP2
PEBP1
NUCB2
QSOX1
CLU
LTF
C3
MFGE8
LGALS3BP
SPP1
PIGR
AZGP1
SERPINA1
LALBA
CSN2
AMY1A
KNG1
CEL
CHRDL2
KLK6
ALB
CSN3
CSN1S1
ORM1
CD14
FABP3
LYZ
MRC1
HP
TCN1
XDH
IGFBP2
IGJ
ALDOA
TPI1
PSAP
YWHAZ
B4GALT1
CSNK1A1
LDHB
SERPINA3
ENO1
HSPA8
B2M
ACTB
1. Luminal 1
2. Luminal 2
3. Myoepithelial
4. Epithelial
5. vSMCs
6. Pericytes
7. Lymphatic Endothelial
9. Vascular Endothelial
8. Fibroblasts
10. Leukocytes
12. Adipocytes
13. Cells (unsorted)
lactotransferrin
Transferrins
LTF
Lum2
casein kappa
CSN3
Lum2
Epi
secreted phosphoprotein 1
Endogenous ligands
SPP1
MEP
Lum2
polymeric immunoglobulin receptor
V-set domain containing
PIGR
Epi
Adip
Lum2
xanthine dehydrogenase
XDH
Adip
Lum2
clusterin
CLU
Endo
Lum1
Lum2
complement component 3
C3 and PZP like, alpha-2-macroglobulin domain containing
C3
Adip
Fib
Lum2
haptoglobin
Sushi domain containing
HP
Lum2
milk fat globule-EGF factor 8
MFGE8
vSMC
MEP
Epi
Peri
Lum2
Fib
mRNA
(Log2, Normalized)
0 4 8 1216
b
c
d
e
f
g
h
i
j

**Figure S19. Transcript levels of milk proteins. A,** Transcript levels of milk proteins. VST gene values are relative (normalized by row; S13 Data). **B-J,** Normalized mRNA values (rlog, DEseq2) provided on log2 scale (bar graph of each biological replicate) and linear scale (donut graph of median value; S13 Data), which are both color-coded by cell type.

Figure S20

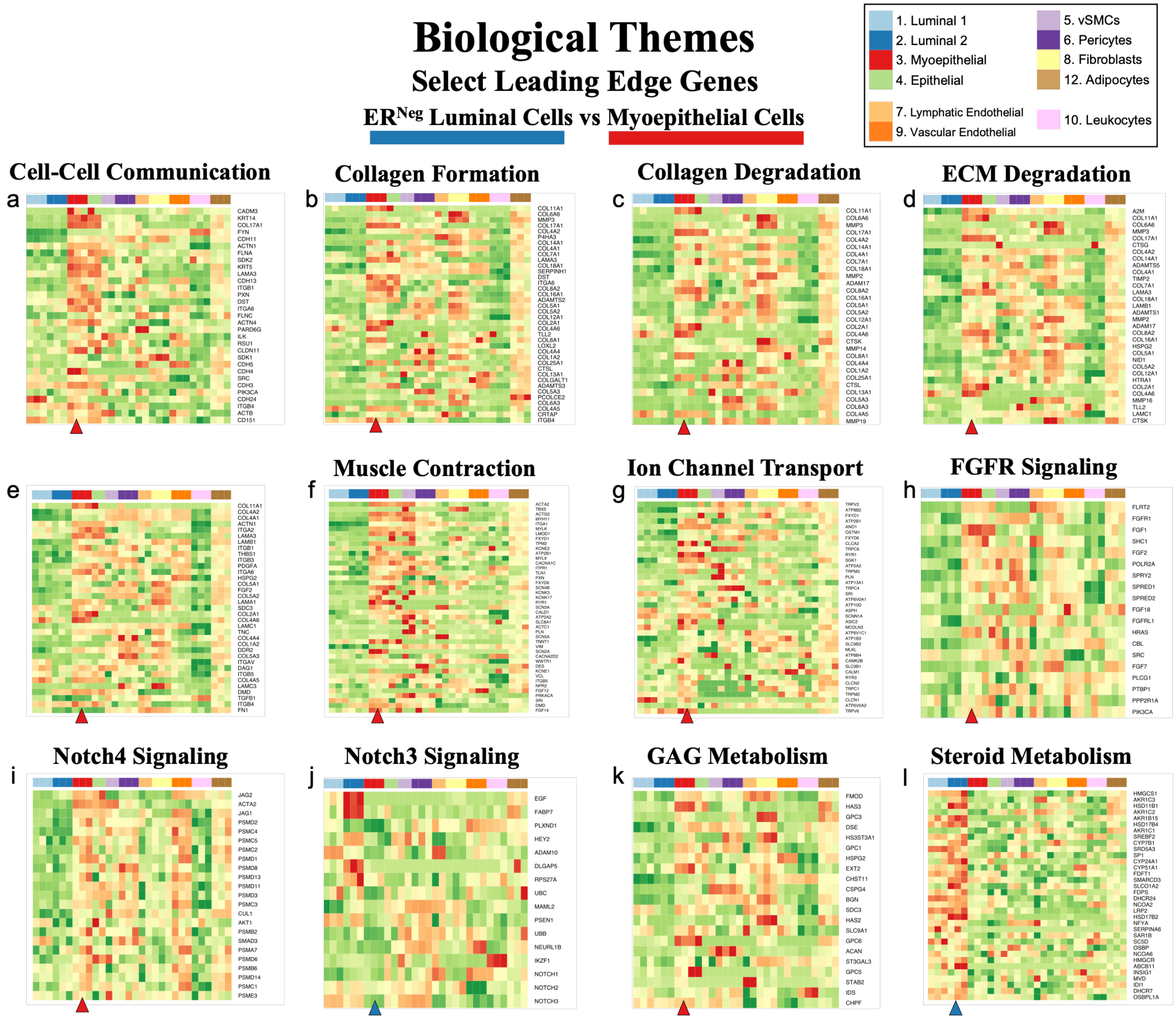


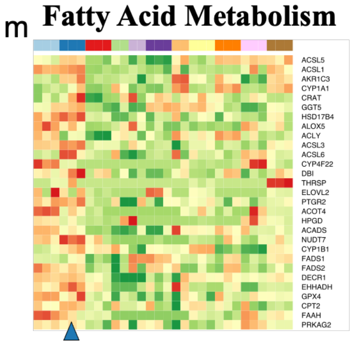


**Figure S20. Transcript levels from enriched pathways**. **A-M,** Transcript levels of leading-edge genes from select pathways enriched in ER$^{Pos}$ or myoepithelial cells (from 426 enriched pathways with a FDR of ≤ 10%, GSEA; S13 Data). VST gene levels are relative (normalized by row). Arrowheads under the heatmaps identify the cell type, either over- or under-expressing the pathway's genes. Samples are color-coded. Due to space constraints and the large number of presented genes, their names (rows of the heatmap) must be inspected digitally (magnified in the PDF).

Figure S21

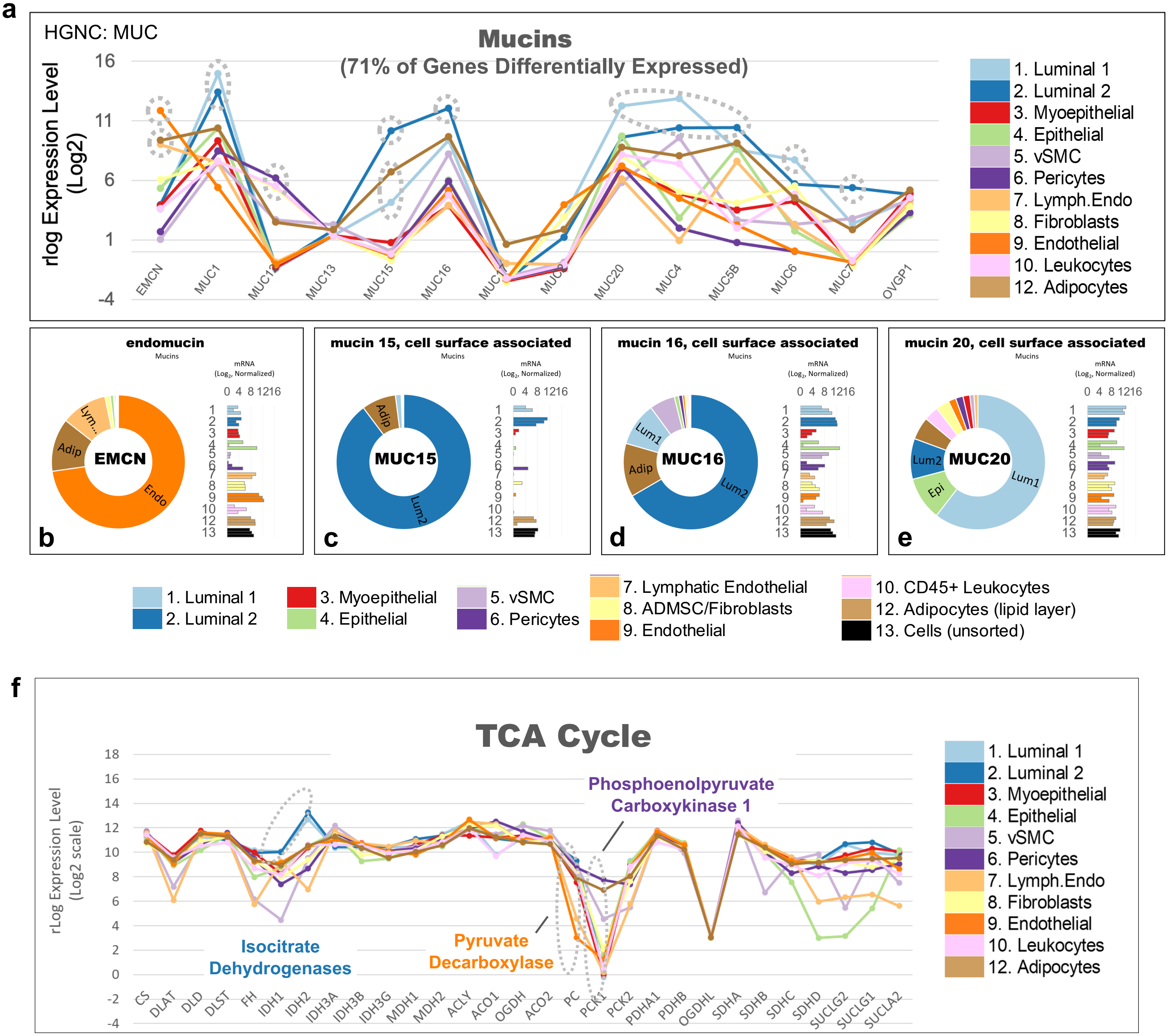


**Figure S21. Transcript levels of mucins and TCA Cycle genes. A,** Transcript levels of mucins. VST gene values are relative (normalized by row; S13 Data). **B-E,** Normalized mRNA values (rlog, DEseq2) provided on log2 scale (bar graph of each biological replicate) and linear scale (donut graph of median value; S13 Data), which are both color-coded by cell type. **F,** Transcript levels of TCA Cycle genes. VST gene values are relative (normalized by row; S13 Data).

Figure S22

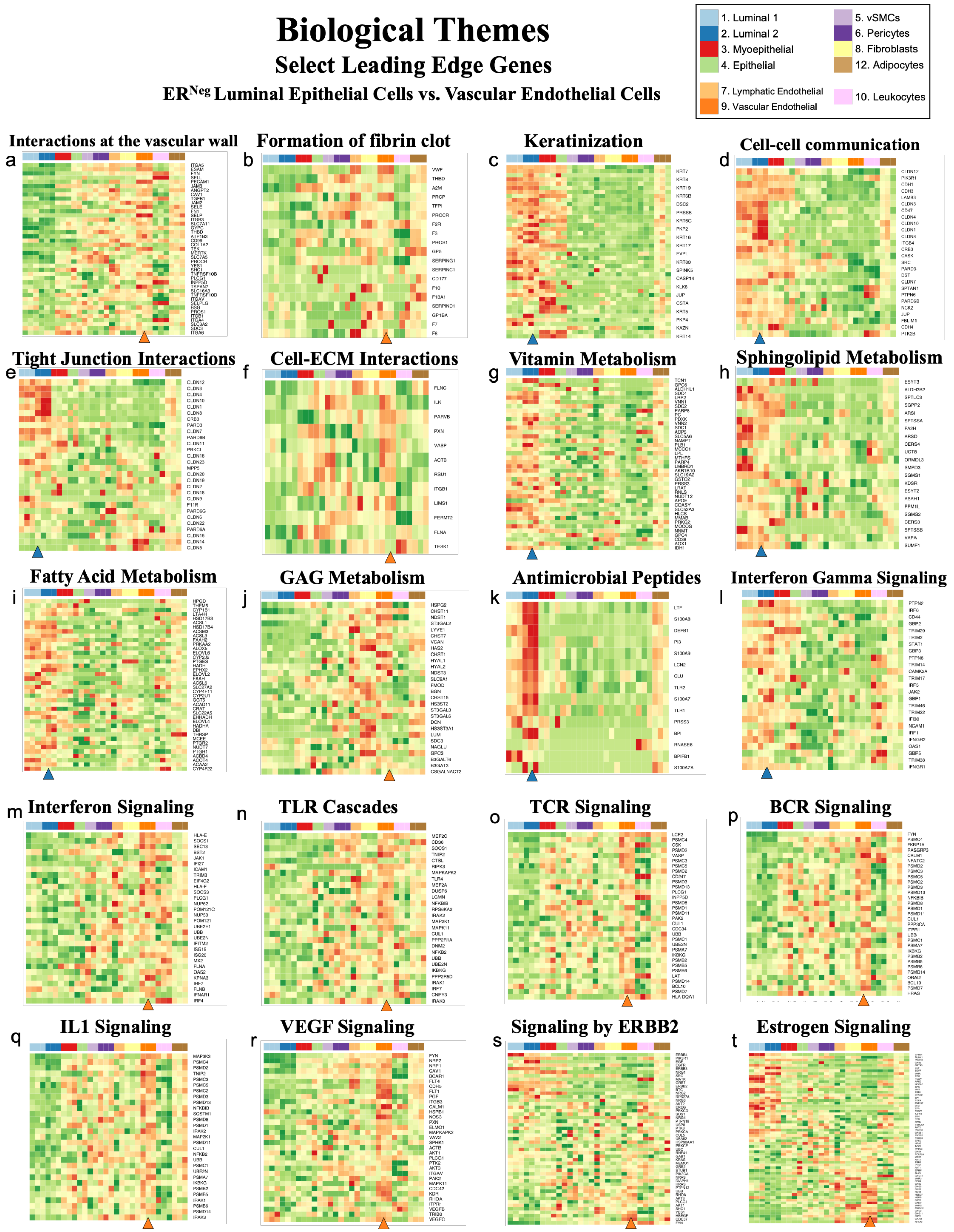
Biological Themes
Select Leading Edge Genes
ER^Neg Luminal Epithelial Cells vs. Vascular Endothelial Cells
1. Luminal 1
2. Luminal 2
3. Myoepithelial
4. Epithelial
5. vSMCs
6. Pericytes
8. Fibroblasts
12. Adipocytes
7. Lymphatic Endothelial
9. Vascular Endothelial
10. Leukocytes
a Interactions at the vascular wall
b Formation of fibrin clot
c Keratinization
d Cell-cell communication
e Tight Junction Interactions
f Cell-ECM Interactions
g Vitamin Metabolism
h Sphingolipid Metabolism
i Fatty Acid Metabolism
j GAG Metabolism
k Antimicrobial Peptides
l Interferon Gamma Signaling
m Interferon Signaling
n TLR Cascades
o TCR Signaling
p BCR Signaling
q IL1 Signaling
r VEGF Signaling
s Signaling by ERBB2
t Estrogen Signaling

**Figure S22. Transcript levels from enriched pathways. A-T,** Transcript levels of leading-edge genes from select pathways enriched in $ER^{Pos}$ luminal and vascular endothelial cells (from 500 enriched pathways with a FDR of ≤ 10%, GSEA; S13 Data). VST gene levels are relative (normalized by row). Arrowheads under the heatmaps identify the cell type, either over- or under-expressing the pathway's genes.

Figure S23

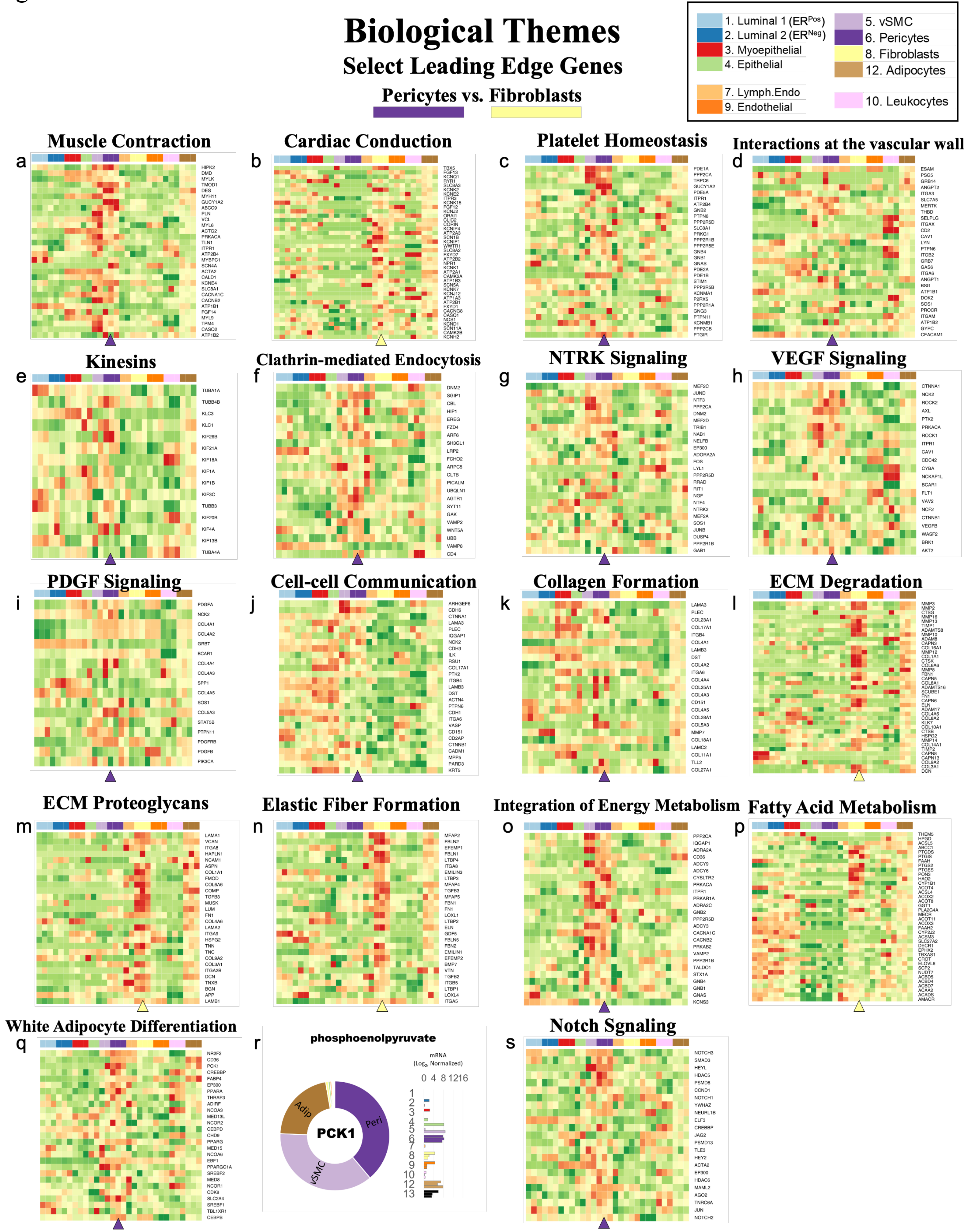

Biological Themes
Select Leading Edge Genes
Pericytes vs. Fibroblasts
1. Luminal 1 (ER^Pos)
2. Luminal 2 (ER^Neg)
3. Myoepithelial
4. Epithelial
7. Lymph.Endo
9. Endothelial
5. vSMC
6. Pericytes
8. Fibroblasts
12. Adipocytes
10. Leukocytes
a Muscle Contraction
b Cardiac Conduction
c Platelet Homeostasis
d Interactions at the vascular wall
e Kinesins
f Clathrin-mediated Endocytosis
g NTRK Signaling
h VEGF Signaling
i PDGF Signaling
j Cell-cell Communication
k Collagen Formation
l ECM Degradation
m ECM Proteoglycans
n Elastic Fiber Formation
o Integration of Energy Metabolism
p Fatty Acid Metabolism
q White Adipocyte Differentiation
r phosphoenolpyruvate
mRNA
(Log2, Normalized)
0 4 8 12 16
PCK1
Adip
Peri
vSMC
1
2
3
4
5
6
7
8
9
10
12
13
s Notch Sgnaling

**Figure S23. Transcript levels from enriched pathways. A-S,** Transcript levels of leading-edge genes from select pathways enriched in pericytes and fibroblasts (from 477 enriched pathways with a FDR of ≤ 10%, GSEA; S13 Data). VST gene levels are relative (normalized by row). Arrowheads under the heatmaps identify the cell type, either over- or under-expressing the pathway's genes.
